# The RAG key to vertebrate adaptive immunity descended directly from a bacterial ancestor

**DOI:** 10.1093/nsr/nwac073

**Published:** 2022-04-18

**Authors:** Xin Tao, Ziwen Huang, Fan Chen, Xinli Wang, Tingting Zheng, Shaochun Yuan, Anlong Xu

**Affiliations:** State Key Laboratory of Biocontrol, Guangdong Key Laboratory of Pharmaceutical Functional Genes, Southern Marine Science and Engineering Guangdong Laboratory (Zhuhai), School of Life Sciences, Sun Yat-sen University, China; Center for Infection and Immunity, School of Medicine, Sun Yat-sen University, China; State Key Laboratory of Biocontrol, Guangdong Key Laboratory of Pharmaceutical Functional Genes, Southern Marine Science and Engineering Guangdong Laboratory (Zhuhai), School of Life Sciences, Sun Yat-sen University, China; State Key Laboratory of Biocontrol, Guangdong Key Laboratory of Pharmaceutical Functional Genes, Southern Marine Science and Engineering Guangdong Laboratory (Zhuhai), School of Life Sciences, Sun Yat-sen University, China; State Key Laboratory of Biocontrol, Guangdong Key Laboratory of Pharmaceutical Functional Genes, Southern Marine Science and Engineering Guangdong Laboratory (Zhuhai), School of Life Sciences, Sun Yat-sen University, China; Shanghai Institute of Immunology, College of Basic Medical Sciences, Shanghai Jiao Tong University School of Medicine, China; State Key Laboratory of Biocontrol, Guangdong Key Laboratory of Pharmaceutical Functional Genes, Southern Marine Science and Engineering Guangdong Laboratory (Zhuhai), School of Life Sciences, Sun Yat-sen University, China; Laboratory for Marine Biology and Biotechnology, Qingdao National Laboratory for Marine Science and Technology, China; State Key Laboratory of Biocontrol, Guangdong Key Laboratory of Pharmaceutical Functional Genes, Southern Marine Science and Engineering Guangdong Laboratory (Zhuhai), School of Life Sciences, Sun Yat-sen University, China; School of Life Sciences, Beijing University of Chinese Medicine, China

The emergence of RAG recombinase to mediate V(D)J recombination has been considered an important milestone in the evolution of adaptive immunity in jawed vertebrates. In past decades, hypotheses, including the invasion of viral or bacterial genes and the transposition of mobile elements, have been proposed to shed light on the origin and evolution of the *RAG* genes and V(D)J recombination. In 2016, our discovery of the long-sought-after *ProtoRAG* transposon in the lancelet—a ‘living fossil’ of vertebrates—directly supported the hypothesis that the RAG recombinase complex originated from an ancestral *RAG-like* (*RAGL*) transposon [1]. A typical *ProtoRAG* contains a pair of recombination signal sequence (RSS)-like terminal inverted repeats (TIRs) and convergently orientated *RAG1-like* (*RAG1L*) and *RAG2-like* (*RAG2L*) genes [1,2]. After the discovery of *ProtoRAG*, *RAGL* transposons were recently found to be distributed widely in bilaterians [3,4]. However, the existence of the *RAGL* transposon in more primitive organisms and its continuous evolution remain to be further elucidated.

After searching for *RAG1* or *RAG2* homologs in >680 000 assembled genomes (Supplementary Table S1), 786 *RAG1L* and 191 *RAG2L* homologs were found in 173 species distributed broadly across the Eukaryota (Supplementary Fig. S1). Although most of the identified sequences were partial, some complete and potentially active *RAGL* transposons were found in some protostomes and cnidarians, as previously reported [3,4]. In Protostomia, *RAGL* genes are complete and tightly linked in several lophotrochozoans but are fragmented and uncoupled in ecdysozoans. A similar observation was found in the Cnidaria, as *RAGL*s are complete and probably active in several corals such as *Fungia costulata* and *Fungia tenuis* (Fig. 1a), but are fragmented and uncoupled in some other cnidarians. These observations suggest that the *RAGL* transposon emerged earlier than the divergence of bilaterians and non-bilaterians. Importantly, complete and tightly linked RAG1 and RAG2 homologs were found in the unicellular microalgae *Aureococcus anophagefferens* (Class Pelagophyceae, Phylum Ochrophyta, Kingdom Stramenopila). Fragments of RAG1L homologs were also identified in several other primitive eukaryotes (Supplementary Fig. S1), tracing the origin of RAGL homologs back to the early eukaryotes for the first time.

**Figure 1.**
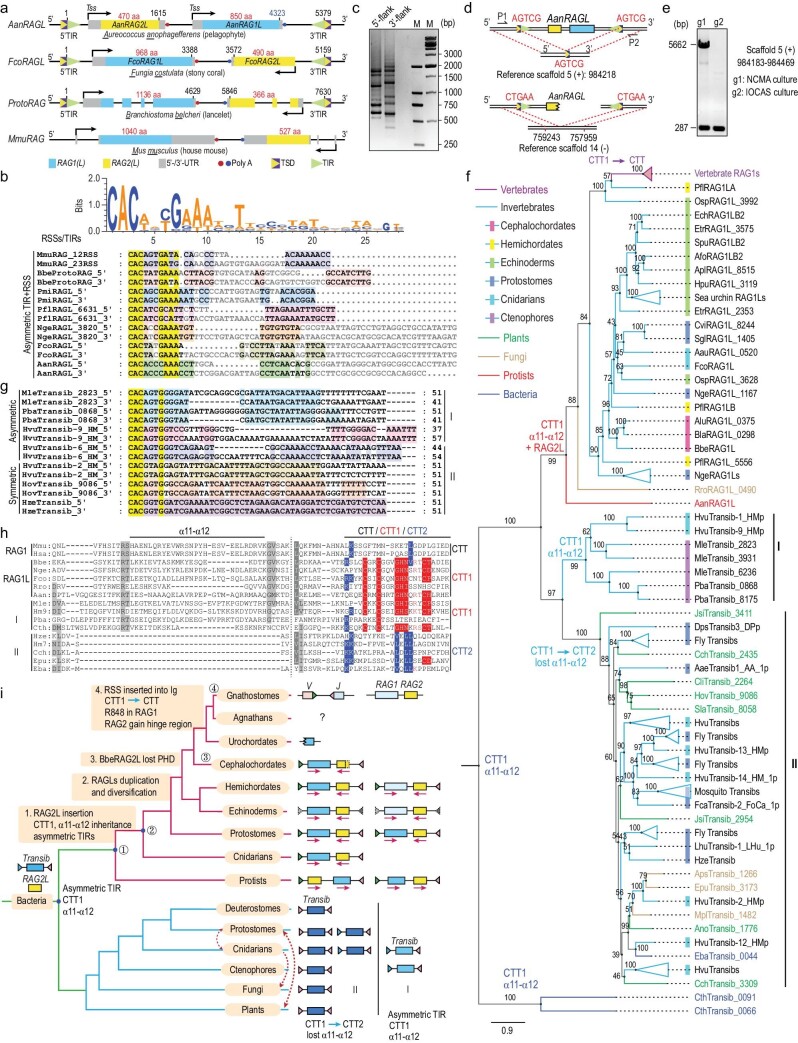
Evolution of the RAG1 and Transib homologs. (a) Schematic diagram showing the genomic organization of RAG-like (RAGL) homologs in *Aureococcus anophagefferens* (pelagophyte), *Fungia costulata* (stony coral), *Branchiostoma belcheri* (lancelet) and *Mus musculus* (mouse). The diagram is not scaled to sequence length. Species names are abbreviated using three letters as shown, which was also applied in the following context. 5’-/3’-untranslated region (UTR), transcription start site (TSS), exons, Poly(A) sites, 5’-/3’-terminal inverted repeat (TIR) and target site duplication (TSD) are shown as indicated. The 5’-/3’-UTRs and coding sequences of *FcoRAGL* were predicted using FGENESH. (b) The consensus sequences of mouse RSSs and *RAGL* TIRs are shown in a weblogo diagram through an unspaced alignment. The spaced sequence alignment of TIRs from *RAGL* transposons and mouse RSSs show a common bipartite conserved structure among them. The conserved elements in TIRs are shaded using different colors. The highly conserved nucleotides are colored yellow. Pmi, *Patiria miniata*; Pfl, *Ptychodera flava*; Nge, *Notospermus geniculatus*. (c) Detection of 5’-terminal and 3’-terminal flanking sequences of *AanRAGL* in the *A. anophagefferens* using agarose gel electrophoresis. The flanking sequences were cloned using the splinkerette-PCR method (primers are listed in Supplementary Table S2) and the original genomic DNA was extracted from the CCMP 1984 strain of *A. anophagefferens* (NCMA cultures). (d) Schematic diagram of two *AanRAGL* loci identified from the splinkerette-PCR assays. The identical regions between the loci and the reference genome are shown by the dash lines. (e) Detection of the *AanRAGL* insertion in Scaffold 5 from two strains of *A. anophagefferens.* g1: CCMP1984 strain from National Center for Marine Algae and Microbiota (NCMA); g2: *A. anophagefferens* from Institute of Oceanology Chinese Academy of Sciences (IOCAS). (f) Phylogenetic analysis of RAG1 and Transib homologs. The maximum-likelihood phylogenetic tree was constructed using IQ-TREE based on the core region of RAG1 and Transib homologs. The optimum LG+F+R6 model was tested and selected, and the ultrafast bootstrap (%) support is shown near the branches. The newly identified Transib proteins are marked using a prefix composed of a three-letter abbreviation of the species name, like those of RAG1 homologs. The primary diversification of protein domains is shown near the branches of the protein clans. The *Transib* family was subdivided into two subgroups according to phylogenetic relationships of Transib proteins. Osp, *Ophiothrix spiculata*; Rro, *Rhizophlyctis rosea*; Mle, *Mnemiopsis leidyi*; Pba, *Pleurobrachia bachei*; Jsi, *Juglans sigillata*; Cch, *Capsicum chinense*; Cli, *Corrigiola litoralis*; Hov, *Hordeum vulgare*; Hvu, *Hydra vulgaris*; Sla, *Silene latifolia*; Aps, *Austropuccinia psidii*; Epu, *Erysiphe pulchra*; Mpl, *Massospora platypediae*; Cth, *Candidatus thioglobus*. The complete phylogenetic tree is shown in Supplementary Fig. S3b. The complete list of species abbreviations is shown in Supplementary Table S3. (g) Sequence alignment of the spaced TIRs from Subgroup I and Subgroup II Transibs. The conserved elements in TIRs are shaded using different colors. The highly conserved nucleotides are colored yellow. Species abbreviation as shown in Fig. 1f. (h) Multiple sequence alignment of representative RAG1 and Transib homologs showing the domain diversification. The Transib subgroups are as defined in Fig. 1g and the conserved amino acids in three types of CTT domains are shaded in color. Mmu, MmuRAG1; Hsa, HsaRAG1; Bbe, BbeRAG1L; Nge, NgeRAG1L_3820; Fco, FcoRAG1L; Rro, RroRAG1L_0490; Aan, AanRAG1L; Mle, MleTransib_3931; Hm9, HvuTransib-9_HMp; Pba, PbaTransib_8175; Cth, CthTransib_0091; Hze, HzeTransib; Hm7, HvuTransib-7_HMp; Cch, CchTransib_3309; Epu, EpuTransib_3173; Eba, EbaTransib_0044. CTT, C-terminal tail; CTT1, type I C-terminal tail; CTT2, type II C-terminal tail. The complete sequence alignment is shown in Supplementary Fig. S5. (i) Model on the origin and evolution of RAG and Transib. The RAG1 and Transib homologs were proposed to have descended from a common bacterial Transib ancestor. After descending from the bacterial Transib ancestor, descendants in Subgroup II experienced complex domain loss and acquisition and spread broadly in different hosts through HGT (horizontal gene transfer) (indicated using red dashed lines). However, descendants in Subgroup I inherited many ancestral characteristics, such as the α11–α12 region, the CTT1 domain and the asymmetric TIRs. At the early times of eukaryotes, one member of the Subgroup I *Transibs* acquired *RAG2L* to generate the ancient *RAGL* transposon. This ancient RAGL then underwent host domestication events in a vertical manner, such as gene duplication [3], transposon fossilization, loss of CTT1, selection of R848 in RAG1 and gain of hinge region in RAG2 [9]. The vertebrate type of RAGLs were generated after duplication and divergence of the primitive RAGLs, which are distinguished using two different blue colors. The fossilized *RAGL* transposons in Echinoderms are indicated by gray TIRs. The evolution of RAG and Transib after splitting from the common bacterial ancestor are independently shown in the upper and lower parts, respectively, but this does not mean that their host species were separately evolved.

After cloning the ancient *AanRAGL* from *A. anophagefferens* (Fig. 1a), typical transposon features of *AanRAGL* elements were found, including a pair of asymmetric TIRs and the 5-bp target site duplications (TSDs). Similar to the TIRs in *ProtoRAG* and the RSSs in V(D)J recombination, the paired TIRs of *AanRAGL* are characterized by two conserved elements, a 13-bp element (CACACCCAAACCT) and a 10-bp element (CCTCAA[C/T]A[C/T]G), which are separated by a pair of 4/13-bp space sequences (Fig. 1b). Bracketed by the pair of TIRs, two single-exon encoded genes similar to RAG1 and RAG2 were identified. However, unlike the other identified *RAGL* transposons, *AanRAG2L* is located upstream of *AanRAG1L* and transcribed toward *AanRAG1L* in the same direction (Fig. 1a). To reveal the transpositional activity of *AanRAGL in vivo*, flanking sequences of *AanRAGL* were cloned from the genome of the NCMA culture (CCMP 1984, source of the reference genome) using the splinkerette-PCR method. *AanRAGL* was found to have been polymorphically inserted into the host genome, which was shown in both the electrophoresis and the alignment of various flanking sequences (Fig. 1c and Supplementary Fig. S2). Among these polymorphic insertions, two paired flanking sequences were seamlessly joined in the reference genome, revealing a recent transposition event in the genome of our NCMA cultures but not in the reference genome (Fig. 1d, upper). This insertion was confirmed by cloning the intact *RAGL* transposon from the NCMA culture but not from another Institute of Oceanology Chinese Academy of Sciences culture (Fig. 1e). Moreover, another pair of flanking sequences without TIRs and TSDs can be well aligned to the reference genome (Fig. 1d, lower), indicating the loss of some *AanRAGL* copies in the reference genome due to transpositional activity. Thus, as early as the unicellular eukaryotes (median origin time 1552 Myr) [5],  active  *RAGL* transposons have appeared and preserved many conserved characteristics of RAG homologs. The divergent gene direction of the *AanRAGL1/2* and some single RAG1L fragments in other primitive eukaryotes imply that the original *RAGL* transposons may have experienced gene insertion or inversion.

Before the discovery of *ProtoRAG* in lancelets, *Transibs* were found to be widely distributed in protostomes and cnidarians, and *RAGL* transposons were thought to be derived from a *Transib* transposon by acquisition of a *RAG2L* gene [6–10]. The recent identification of *RAGL* transposons in protostomes and cnidarians suggests another possibility: that *Transib* arose from a *RAGL* transposon by loss of *RAG2L* [4]. To conclusively clarify the evolutionary relationship between *RAGL* and *Transib* transposons, we searched for Transib homologs as performed in RAGL and identified dozens of potential Transib-like proteins in bacteria (42), fungi (20), plants (39), and ctenophores (6), and hundreds in other metazoans. Analysis of the Transib and RAG1L proteins showed that the average protein identities among RAG1L core, Transib, combined RAG1L core and Transib were 36.75%, 34.28% and 26.22%, respectively (Supplementary Fig. S3a). RAG1L and Transib proteins were phylogenetically clustered into two separate clans after setting the root ahead of the bacterial Transib branch (Fig. 1f and Supplementary Fig. S3b). The pelagophyte AanRAG1L was in the root of the RAG1 clan and the phylogenetic relationship of RAG1 homologs was generally consistent with their host species (Fig. 1f and Supplementary Fig. S4a). In addition, RAG1Ls were expanded into several diverged copies in some protostomes, echinoderms and hemichordates, and appeared to evolve slowly in chordates. Similar observations were obtained from the analyses of RAG2 homologs (Supplementary Fig. S4b). These results suggest that the evolution of the *RAGL* transposons was mainly in a vertical manner and probably accompanied by limited HGT.

For the *Transib* clan, two major subgroups were gathered and defined as Subgroups I and II (Fig. 1f and Supplementary Fig. S3b). The Subgroup I *Transib* transposons all contain a pair of asymmetric TIRs and show a closer relationship with RAG1L than those of Subgroup II (Fig. 1f and g). However, the Subgroup II *Transib* transposons, which include most of the previously identified sequences, contain both asymmetric and symmetric TIRs (Fig. 1f and g). Unlike the vertical evolution of the *RAGL* transposons, the *Transib* transposons may have experienced massive HGT events, as the phylogenetic relationships of Transib transposases were not consistent with that of their host species.

Close to the root of the phylogenetic tree, *RAG1L* and *Transib* superfamilies share a common ancestor that may have descended from bacterial *Transib*. Analyses of the composed domains in these ancient RAG1L and Transib proteins further reveal their conservation and diversification during evolution. First, the RAG2-binding region in the zinc-binding domain (ZnB) of RAG1L (α11–α12 in RAG1 core) was previously found to have been lost in Transibs [10]. Here, we found that the ancient bacterial and Subgroup I Transibs but not Subgroup II Transibs harbored an equivalent α11–α12 region (Fig. 1h), suggesting that the RAG2-binding region in ZnB was a constitutive region in the Transib ancestor but was exclusively lost in Subgroup II Transibs. Second, both RAG1L and Subgroup I Transibs contain the CTT1 domain (previously called CTT^*^, type I C-terminal tail), whereas a structurally different CTT domain (CTT2, type II C-terminal tail) was found in Subgroup II Transibs (Fig. 1h). The CTT1 domain has been shown to be critical for the lancelet RAGL complex to interact with its intact TIRs [2,9], whereas the CTT2 domain may be important for the interaction with the ZnB domain of Transibs [10]. Thus, the CTT1 in RAG1Ls should be inherited from a constitutive region in Transib ancestors, whereas the diverged CTT2 in Subgroup II Transibs may be an adaptation to the loss of the α11–α12 region in their ZnB domain. Third, similar to the diverged NBD^*^ in RAG1L proteins, an equivalent nonamer binding domain (NBD) with several conserved positions (GRP in RAG1 NBD, Supplementary Fig. S5) was found in both Subgroup I and II Transibs, suggesting an equivalent NBD domain in the ancestors of RAG1L and Transib. In addition to these domains, several short regions in RAG1Ls and Transibs also experienced specific gain or loss, such as the gain of the loop region between β1–β3 in the pre-RNase H (PreRNH) domain of RAG1Ls and the loss of partial α1 and α17 in Subgroup II Transibs (Supplementary Fig. S5). Overall, Subgroup I Transibs preserved some ancestral regions shared by RAG1Ls, which were exclusively lost or diverged in Subgroup II Transibs. Subgroup I Transibs may represent intermediates in the early evolution of Transib and RAG1L.

Finally, we proposed an updated evolutionary model to elucidate the origin and evolution of the *RAGL* and *Transib* transposons (Fig. 1i). After descending directly from a bacterial *Transib* ancestor, the vertebrate *RAG* genes and their homologs mostly evolved in a vertical manner beginning in eukaryotes, whereas the *Transibs* experienced massive HGT. As intermediates for the early evolution of *Transib* and *RAG1L*, one of the Subgroup I *Transibs* acquired *RAG2L* early in the eukaryotes to generate the ancient *RAGL* transposon. This ancient *RAGL* then underwent host domestication in a vertical manner, including domain gain and loss, gene duplication, transposon fossilization and key amino acid adaptation, to finally shape the RAG machinery in vertebrates.

## DATA AVAILABILITY

The representative sequences annotated during the current study are provided online in Supplementary Data 2. All other relevant datasets could be provided upon reasonable request.

## Supplementary Material

nwac073_Supplemental_Files
