# Supplementary material for: The RAG key to vertebrate adaptive immunity descended directly from a bacterial ancestor
Source: Natl Sci Rev. 2022 Apr 18;9(8):nwac073. doi: 10.1093/nsr/nwac073 (PMC9435367; doi:10.1093/nsr/nwac073)
Supplement: nwac073_Supplemental_Files [file nwac073_supplemental_files.zip › Supplementary Data2_taoxin-20220516.pdf]

SUPPLEMENTARY DATA 2

RAGL loci 1

Protists 2

Aureococcus anophagefferens 2

Cnidarians 6

Fungia costulata 6

Fungi 10

Rhizophlyctis rosea 10

Transib loci 16

Ascidians 16

Salpa thompsoni 16

Ctenophores 18

Mnemiopsis leidyi *18*

Pleurobrachia bachei *34*

Fungi 42

Massospora platypediae 42

Austropuccinia psidii 47

Erysiphe pulchra 48

Bacteria 52

Candidatus Thioglobus 52

Entomoplasmatales bacterium 57

Plants 62

Capsicum chinense 62

Hordeum vulgare 69

Corrigiola litoralis 73

Juglans sigillata 76

Silene latifolia 83

Arabis nordmanniana 87

Summary of newly identified representative *RAGL* loci

Protists

Aureococcus anophagefferens

1. AanRAGL genome sequence

TSD TIR Exon Annotation

```
    >>>5'TIR
   1 CACACCCAAA CCTTGCACCT CAACACGCCG GATTAGGGCA TGGCGGCCAC
  51 CTTATCGACG TATTAGACGG CGACACCCGC TGTAAAACCG GTCCCGTCAG
 101 CAGCGCTAGC ACCCCATAGG AGCGCGCAGC CGTTCCAACT GCTCGAAACG
 151 AAGACTTTGC GGCCTCGCAC GGCCTGCTGG AGCGTCGCAG CGCCTGCGGC
       >>>AanRAG2L ORF begins
 201 AGATGTCGCG GCGCCGGCAG GCTGCGGGCA CGGCCAAATC CCGCACGCGG
 251 CATCAAGGCC GCCGCCCCGC GCGACCGGAA TCTTCGCCGC TGCCCGAGTT
 301 CGTCAAGCAT CTCGACACGT GCGCCGGGTT CATGGATCGT AAAGACGCGA
 351 GCCGTCTCGC CCGCGCGTCC AAGGGCAACC GCGACGCGCT GCTCGTGACG
 401 GGCTGGGCGC ATGTCGCGCC GGTTTTCGGC AGTCGGGTGC GGTCCGAGAT
 451 CGTACACCTT TCAATCCCTG CCGGAGGCGG CGCAGAGGCC AGGGACGCCG
 501 CGACCGAGCG CCTTGGCGTG GTCGCCTCCG CCATCGTTCC GGTAGGCGAC
 551 GGCACCGCGC TGTTCTACGG CGGCGAGTTG GCGAGGAACA AGCGGCTGCC
 601 GTGCGTGCTG AGCGTCGCGC CGAAGACCGG GGTCGTCTGC GACGCGCCGC
 651 TCTGGACCGA GTCCGACGAG TTGGACGCGA CGTCGTTCTG CGCGGGCGCG
 701 AAGCGCGGGG AGGAGTTCTT GTTCTTCGGC GGTCTCGACC GCTACTTCCG
 751 CGTGACGAGC CTCTTGCGAT CTGCGGTCTC TCTTCGCAAA AAGACCGGCA
 801 CGTCGACGGC GAAGAAGTAC TTGTTCCGGA GCTACAAGGC CTCGGACGAC
 851 ACGCCGTCGC CGCGGTACGG GTGCTCCTTC ACGTATTTCG CGGAGTCGGG
 901 CGAGATGTTC CTCTTCGGCG GCTTCACTGC GTCGCAGGCC GGCTCGCTGG
 951 CCGTCGTCGC GGACGACGCG AAGCTCTACG TCCTCTCTTC GAGAGACGCC
1001 GACGCGGACC CCTGGGAGCG GACGAGCCCC GGCGCGTGGT CTGCGGTCGG
1051 CGGCGCCACC GGGACCGGTC CCGGGCCGCG GGGCCTGCAC GCCTGCGTCG
1101 CCGTCGGGTC CGCGCTCCTG CTCGGGGGCG GGCTGTGCGG CCCGAAAGGC
1151 CCGCTCTGCG CCGAAGAGGT CCACGCCTTT GACGTCGACG CCAAGGTCTG
1201 GAGCACGCTC GCCTTCGACG GGCCCAGCGT CGGCCTCCGG TACTGCCATG
1251 CGATGTTGCA CGACGGGGAC GACGGCGTCA TCGTCTTCGG CGGCAGCTCG
1301 TCCGTCTCCG AGCTTCCGGA GACGAACGCG CTCCAGCACT TCCGTCTCGA
1351 CGGTGGACGC GCGTCCTTGC AGGCCCTCGC CTGGGACGGG CCGTCGTCGC
1401 CCCGGTCCTG CGCGGTGCTC ATCCCCATGT CCGCGCCGAG GCCCTCGAGC
```

1451 ACGGTCACCT TCGCGATCTG CGGCGGCTAT GCCGGCTCTG CTGGACGCAT
1501 CGCACCCCCA GTTTCCGCTT CCAACTTGTT CTACCTCACC ACCGTCGAGG
1551 TTTCGCTGTT CAAGCGCCCG CGCGACCTCG AGTTTGCGCC CGAGCCCGCG
1601 CCCGAGCCCG CGTAAGCTTG CTGCGTGGCT TCACGCGTGG CCGCGTCGAC
<<< AanRAG2L ORF ends
1651 CGCCACGGGC GCGACGCCCA AATTTACCTC AAGCTCGGCT TCAGCGATTC
1701 CAGGCTCCTT GCTCGGCGCC GCTCTCGGAG CCAGCGCGGG CGCGCGGAGT
>>>AanRAG1L ORF begins
1751 CGGGCCAGAG CGCGCGCGCG ATGGAGGTGC CGGACCTGGA CCCGACGGTC
1801 TGGCCGGCGC TCACGAAGTT CCTCACGTTC TGCAAGGCGC GACGGCGCGC
1851 TTCGCGCTCA CGCCGCAGTG GCTCTACCGA CTCTGTCACC TCGCAGACGT
1901 CGCAGACCTC GCAGACGCCG CGGCGATTCG ACGGTGTGTG GGTCGACGTC
1951 CACGTGCTCC AGAAGCAGCT CTGGTGGTGC GACGACGAGC AGGGCCTCGA
2001 CCTCGCGTGG GAGGTGGTGC GGTTGGGGGA GGAGGCGCTT CGGGCGCACG
2051 GATTTACGAT GCGAGCCCTC GGGCGGCACG CGAGCAAGGA CAGCGTCATG
2101 CACCTCATCA ACGCCGTCGA CGTGACGCAC GACGACCGCT ACGCCTTCGA
2151 GGTCGACGAG TACGCGCGGG CGCGGTCCCA GAGCAGCGTC GCGGCCCCGC
2201 CGGAGCAGGC GGCGGCAGAA CCCGAGGGCG CGCCGCCGCT GAAGAAGCCC
2251 CGCAAGGCTC TGACGACGAT CACGCAGCAG TGGCTCGGGC GGCGCCTCGG
2301 CCCCGACCTC ATCAAGGAGA TCGGCGTCGT GGCGGAACGC GAAGGCCTGC
2351 GGGGCATCGA CGTCGCTGCG TCGCTCCTCA AGTACGTCGC GCGCGCGGGC
2401 AACGACCGCA GCCAGTGGCG CATCGGCGAT TGCGTCGAGA CCATGGTCAC
2451 CGGCGGCGCC ACGCCGTTCT GGTCCAAGGA GGAGAGTCTG GCCTACCGCA
2501 TCACGCAGGG CCTCTCCGTG GAGAAGTACA AGTGCCAGCG GAAGCTCGAG
2551 AAGCAGGTCA CGGGCAAGTC CCTGTTGGCT CCGGCTGGTT CCATCGACAT
2601 CTCCTTCCAG AAGACATTTC TACCGGGCTG CGACGACGGC ATCTCTATCA
2651 CGGTCGACTT CGACGGCTAC GCGTCGATGG TGCCCCAGCC CTCGCCGGGC
2701 CTGGTCGGCG CACGGTTTCC AGTCCGAGGT GCTGTCAAAT GCGTCCTAGC
2751 AGCACTGCTG AAACGACATC TGCACCGCTA TTGCATGGAT CACCTCGTCG
2801 TCGACGGCGA CGCCGGGAAT CTGGGCTCAG AGGACGGCGA CTACGACTTC
2851 CGGCCGTTCT TTGGGAAGTC GGCGAGGCTG CTCGTGCGGA TCAAGGAGGG
2901 CTTCGACGGT CTGGGCATGG GCGAGGAGTC GGCTGCCAAG GGCACACACG
2951 TCATCCGCGG CGATTTTGGC GTTTTGGCCA TCTACCACTG CGCGGAGCCG
3001 GAGACGACTT CGAACTCGAC TCGGAAGCGG CGGCGCAACA CGGGCGGCCG
3051 AGGGGACCCG AGCAACTGTG CCTGCGGCCC ACTCGGCGAC GGCTGCGACA
3101 TCTGTGGGAA CAAAATTCTG TTTCTCGAGC CGAAACCCAG CTCGAACCAG
3151 GTCTCGCACC CCTTGGTCTT GGTGTACGCC AATGAGAACA ACGACTCCGA
3201 GACGGGTTTG GCCCTCGTGG CAACCAAGGC CGAGTGGGCC GAGCTCGACG
3251 GCAGCACCAT TGAATTAGAC GTCGGCCATG TCCTCTTTCA CGCGGAGATC
3301 GACGTCTACC CCACCATGTA CGACAAAAAA CTTGAGCGAT CCATCACGGG
3351 ACAGATGGGT ACGACTGCGC ATTACAACTG CACGGACTGC ACGATGACCC
3401 AGGACCAACA GCGCGATCCT ACTCTCGTCG GCCAGGGCGA ATCAATCACT
3451 TTCAAGCGAA CGATCGAGTC GAATGCAGCG GCCCAGGAAT TGGCAGTGCG
3501 GCCCGAACCC GGTACGATGG CGCAGCGACG CATGGCTGCC CAGGGAATGA

3551 AGACCGACGT CATTATGGCC GCACTGGATC TGGTCAGATC AAAGACCGAC
3601 ACTTTACACA CCGACGTGAA CGGTTCTGGC AGCTGGATAG AGTCTATAGT
3651 CATCCACGAA AACGCCCAAA TTTACACGTG GTCGATCACG GCGAGCCATA
3701 AAGAGGCCTA CTCTATTGCA AAGCGCCTAT TTCACGACCA TCTGCGAGTG
3751 TCAATCCGTC AGCATTCGAC CATGATGATT GCTGGCAACT ACGGCCGAGC
3801 CTTGGTCGCC GAGGAGAACG AACGTGTCGT CGTGAGCCTC ATCGCCAATG
3851 TCGAGCGCCG CCAAAAGGTC CTCGAGATCA TCCGCTGCTG GCGTGCCATG
3901 CGGACTGTTT GGCTCTCTGC TACGTCGGAC GCGACCGCCC TGTCAAGCTA
3951 TGATCGCCAC CGCCGGAGAC TTCATGATTT GATCACGAAT CCTGCCCATC
4001 TAGGCGGCTA CATTTCGATA CCATTCTACG TCCACACGCA CATCGAGCAC
4051 ACGCTCAACT CCCTGCCCAT CGCGCCATTC TCTACCGAGT CCCTGGAGGC
4101 CGGGAACAAG GTCCTGCGCC ACGTGCTCAA GCTCCTCAGC CAGAACAATC
4151 GCGAGGGCAT CCGTGGCGCG TTGCACATCC TCTGGCTACG CTCTCATCCG
4201 CTCCTGCGCG AGCTCATGGA GGTTGAGACC CAGGAGCAGG AGTGCCGCAT
4251 GTGCCACCGG ACGGGCCACA ACCAGCGGAC GTGCCACCTG CGCCACGAAG
4301 CCGACGAGGC GGATGAAGAC TAAAATCTTA GCGCGTTTTC GGGCCCCGCG
<<<AanRAG1L ORF ends
4351 TACCCGTAGA GCACCTAGAG GAGCTCTCTT GAGGCCGGGC GTGCGTCGCA
4401 GGTGACCGGG CCACCGCCGG AAAGCCCGCG GCGTCGCAAA GCCATGTACT
4451 TGGAGCGTCG ATAACGAAAA AGTCCGGGGG TACAGACGCG AACGGCGACC
4501 GCGCACCGCG CACGAACGTG TCGGTTTAGG ACCGTCTCCG TCGACGTACG
4551 ACTTCGACGC CGCATAGAAG CACGTCGGGT CCAGCGCTAA TGAGTGCTCC
4601 ACGGCCCTCG CCCATCGATA GGATGAGCTC GGAGCCCATA TCTTCGCACC
4651 GCCGCCCCCA TGGGCGCCGC GTAGCCGTCG CCGAGCGGTT GCGCGCGTGT
4701 GCGCCGAGCA CGACGTTCGC GTCCGCCGAG CGTCGCAGGT CGTCCGCGCC
4751 CGCGAGGAGT CGTTCCCCGT CGCTTCGTGG AGATGTTTGG CCGCGAAATC
4801 GATCAAGTCC GGGGGCACGG ACCGCGGCCC CCGCGCGCCG GCCGCCGCAG
4851 AGCGGCGCCC GAAACCGCCG CCGAAACGGG TTCTGGGGTC ATGAACGGCG
4901 ATGTGGCGTA TTTGACGTGA TCTGTTGGGC CACGGCTCAC CCATAGTGAT
4951 AGACGAGCTG CACGAGCCAC TTCGTCGACG GGAACTCGCG CAGATCTAGC
5001 CGCGGTCCGC GCCTCGAACG TGTCGTCGGG ACCCGTCTTC GGCCGCGCGC
5051 CGCACCACGA CCACGGCCTC GGCCGACGCA GTCGAGCGCG CGCCGCATCT
5101 ATGCGCAACA TGGACGGACC GCCGCGACGG TTTACGGACC CGCGGCGACC
5151 TCCGGACGCG TCCAGCGCCG CCCGCTGCTC GACCGCTCCC GCAGGCGGTT
5201 TCCGAGGCCG TGTGCGTCGT CGCGCGCACG CCGTTTCCAG CGCTCAAAAG
5251 TGGCACGCGA CCGCGCAGGG ACTAGTAGAC GCGGACGGAA GCGGCGCGGG
5301 GGTCACAGGC GGCCTCATAC GGCCTGTGGC GCGGCGCGAA GGCCATATTG
5351 AGGCTAATCG TCCGAGAGGT TTGGGTGTG
<<<3’TIR

AanRAGL_5'TIR : CACACCCAAACCTTGCA---------CCTCAACACGCCGGATTAGGGCATGGCGGCCAC
AanRAGL_3'TIR : CACACCCAAACCTCTCGGACGATTAGCCTCAATATGGCCTTCGCGCCGCGCCACAGGCC

Figure 1 Alignment of 5’-and 3’-TIR of AanRAGL showed their conserved elements.

2. AanRAG2L protein sequence

```
  1 MSRRRQAAGT AKSRTRHQGR RPARPESSPL PEFVKHLDTC AGFMDRKDAS
 51 RLARASKGNR DALLVTGWAH VAPVFGSRVR SEIVHLSIPA GGGAEARDAA
101 TERLGVVASA IVPVGDGTAL FYGGELARNK RLPCVLSVAP KTGVVCDAPL
151 WTESDELDAT SFCAGAKRGE EFLFFGGLDR YFRVTSLLRS AVSLRKKTGT
201 STAKKYLFRS YKASDDTPSP RYGCSFTYFA ESGEMFLFGG FTASQAGSLA
251 VVADDAKLYV LSSRDADADP WERTSPGAWS AVGGATGTGP GPRGLHACVA
301 VGSALLLGGG LCGPKGPLCA EEVHAFDVDA KVWSTLAFDG PSVGLRYCHA
351 MLHDGDDGVI VFGGSSSVSE LPETNALQHF RLDGGRASLQ ALAWDGPSSP
401 RSCAVLIPMS APRPSSTVTF AICGGYAGSA GRIAPPVSAS NLFYLTTVEV
451 SLFKRPRDLE FAPEPAPEPA
```

3. AanRAG1L protein sequence

```
  1 MEVPDLDPTV WPALTKFLTF CKARRRASRS RRSGSTDSVT SQTSQTSQTP
 51 RRFDGVWVDV HVLQKQLWWC DDEQGLDLAW EVVRLGEEAL RAHGFTMRAL
101 GRHASKDSVM HLINAVDVTH DDRYAFEVDE YARARSQSSV AAPPEQAAAE
151 PEGAPPLKKP RKALTTITQQ WLGRRLGPDL IKEIGVVAER EGLRGIDVAA
201 SLLKYVARAG NDRSQWRIGD CVETMVTGGA TPFWSKEESL AYRITQGLSV
251 EKYKCQRKLE KQVTGKSLLA PAGSIDISFQ KTFLPGCDDG ISITVDFDGY
301 ASMVPQPSPG LVGARFPVRG AVKCVLAALL KRHLHRYCMD HLVVDGDAGN
351 LGSEDGDYDF RPFFGKSARL LVRIKEGFDG LGMGEESAAK GTHVIRGDFG
401 VLAIYHCAEP ETTSNSTRKR RRNTGGRGDP SNCACGPLGD GCDICGNKIL
451 FLEPKPSSNQ VSHPLVLVYA NENNDSETGL ALVATKAEWA ELDGSTIELD
501 VGHVLFHAEI DVYPTMYDKK LERSITGQMG TTAHYNCTDC TMTQDQQRDP
551 TLVGQGESIT FKRTIESNAA AQELAVRPEP GTMAQRRMAA QGMKTDVIMA
601 ALDLVRSKTD TLHTDVNGSG SWIESIVIHE NAQIYTWSIT ASHKEAYSIA
651 KRLFHDHLRV SIRQHSTMMI AGNYGRALVA EENERVVVSL IANVERRQKV
701 LEIIRCWRAM RTVWLSATSD ATALSSYDRH RRRLHDLITN PAHLGGYISI
751 PFYVHTHIEH TLNSLPIAPF STESLEAGNK VLRHVLKLLS QNNREGIRGA
801 LHILWLRSHP LLRELMEVET QEQECRMCHR TGHNQRTCHL RHEADEADED
```

Cnidarians

Fungia costulata

4. FcoRAGL transposon genome sequence

```
TSD TIR Exon Annotation
     >>>5’TIR
   1 CACTATGAAA AGTCCTTATA AATATTCATT TATGCAAATT AGCTCGTTTC
  51 AGTCTTCCCT TGTTGTTATT GACGCGCGGC CGAATTTCAT TTTCGCTGCG
 101 TGGGTTGCGA GTCATTCATG CGCGAAGAGA CTGGAAGCGA AATCGAGCCC
 151 TATAAGCATG CGCAATTGTT TCCAATCGAT TCAGCGTGGC TGCTTTCGTG
 201 TAAAAAGTTC CTTCGAGTGT TGATCGGTGT TGTTCGCGGT TTGTTTCAGA
 251 TTTTTTAAGC TTTCGTCAAA TTTTCAGCTG AAGCTAGATC GAAAGATTGT
 301 AAACGGTAAA ATTATCTGTG TTTTAACGAC CTTGCCGGTC CATGCCGGTT
 351 AGGTTTCGAG CTTTTACATT GCTTTTGGAC TACTCTGGAG GAAAGTAAGT
 401 GGTTTCTTTA CTTTGTGCAC ACGCGTGAAC ATAGTTTAAT TTCACGAAAA
                                     >>>FcoRAG1L ORF begins
 451 AGGTTGGTGG TTTAAGAGTG TTCCGTCCAA GATGGACGTC AAGTGTCACA
 501 AGGAAAAGTT GGCTTCGTTT TGTCGGCGAT GCGGGAACTT TTTTGATACA
 551 AATCCAGCAA CAAGTAGACA TAAACCGAAG CTTGCTGTAA CTTTTTCTAA
 601 GGAGCTCCAA GCAGTTGATG GTTTAGATGT GAGCAACGAC AGTGAGGAAA
 651 TGCATCCACC GTCTCTGTGT CGATGCTGTA TTTTAAAGCT TGAACGTTTC
 701 AAGAAATCGA GAAACAGAAA CGATCGTCAA TTAAAGTTTC CAGGTGACAA
 751 AGGTAAATGT TGTGTGTTTT TGACTCATAA TTCCTATGTG AAAGAGGGAA
 801 GTTGCCCAAT TTGCTGTTTT AATTTAAGAC AAAGCCCAAG AAAAAAAAGG
 851 CCCCATGAAC CTGACAACAG CCCCTCTGCA AGTGGAAATG CCAGGGAGAA
 901 TTCAGAGGGA GCTGCAGCTG GGGGTAAAAA GAAGCCTCCT TTTGTTAGAC
 951 AGGTCTTTAC CAGTTCAGCT GAAAGTTCAA CATCAGGAAT GGCAAGCAGC
1001 ACCAGCACTA GTGAAACCCC TACGACAAAA ATTTTCACCG AACAGGAACC
1051 TTCCACTGCA GCTGGTCCAT TTGAAGGTGA TACTTTTGAA CTCACTGGCA
1101 TTCCCCTTGA CAGATTTGTT GAACAGCAGT TTGCTGAAAT ATTTCAATGC
1151 AATATCTGCA TGGATATTCC TACTGATGCT GTAATACTTA GTAACTGTCA
1201 TCATGTATAT TGTGAACATT GTATTCAGCG CTGGCTAGCA TGCAATGGTG
1251 TTTGCCCATC GTGTCGTAAT CTCGTAGAGC TTGACGATGT TCTCCCTCTG
1301 CGTCAGCAGA TGTTGACCAT ATTTGATATG CTGACATTAC ATTGCAAGTA
1351 TTCTGAAAAT GGCTGTGAGG AAAAACTTAC TGTTAATGAA ATTGCTGAGC
1401 ACGAGAAAAG TTGTAGATTT TCAAAAAAGA AACTAAAGAG GGGGGCCTAC
1451 AATAAGACAA AACTATTTGA TATTTCACGA CAATATTCAA AGCGCGGTAG
1501 ACTAAGAAAC ATGTTTGACA ACCTCTCTGA GTTTTGTCAA TCCCACAACG
1551 AGGATGCTGA GGATGTTTTG TTTTCAATGT TGACCACAAC ACTACATGAC
```

1601 AAAGGCAAAA CAGAATTAGC AAAAAAGGTT GAGTATTTGT GGACCGAGCA
1651 GACTGATAAT GATTTAACAG TTGATGAATG TCTGGCAAAT CGAATTAACA
1701 TGCTGCAGAC AAAGAATCAA TACAAGAAAC AGTATGACTT TTTAAATAAC
1751 AAAGGGCAAT TCTTTTTCAA GCCCCTGTAC TCATTGGACA AAGCTGAAGA
1801 CACTTACCTT CCTTCGTACG TAAATTACAA AATTAAAGAC AACATGGACA
1851 ATATCATTTT CAATCATAGC GCATGTAAAT CTGAACCCAT TAATTTGATG
1901 GCTGACTTTA GAGAGGCACT CCCACAGTTT CCTGTACCTA ATGTTGTTGG
1951 TGTTCGATGG AGTTATCGAA ATGCCCTTGC TAAATCTTTA CAAGAGTTAG
2001 GTAGTGAAAT TGATAAGGGC TTGGATAATT GTGGCATTAC AGGCAATCCA
2051 ACACTTCAAG TTCTTATAAA AGATGGTGGT GATGGTCTTG GTGATGTTTC
2101 CATGTACAAA GAGAAAGGAG ACAGATATTT AGAAGACAAG GCTTATAGAT
2151 ATTCATTTTG CGTTCTGAAA ATCACAGTAG ACAGAGAAAA TGAAAGAGTA
2201 GTTATTTGGG AAGAGGATAC CCCAGGCTCT GTACGTACAA ATAAGACTCT
2251 AATTGAGGCA GTGTGTGACG AAAACCAGAC AGCTGCTATG GTTGCATGTG
2301 TTGTACCAGT AGAAAGGGAG CGTGAGCAAA TTGCAAATAA TTTAATTAGT
2351 GTGGAAACTG GAACTTTGTG GAGGAATTTT AAAGTTGAAT TCATAAATTC
2401 TATGGAAGAT GAAAAACGGG CACGCGCTGA TGGTGGACTG CAGGAAGCTG
2451 GATCCAGATT TATGTGTGAC TTGTGCTATG CGACACAAGA TTCAGCAAAA
2501 AGTGATCTTG GAACATTTAA AACCTGTCGC ACCCTTGAAG AGACCAAACA
2551 AATTGCTGAT CTTCTTCATT TTAATCCTAG CAAACTTACT CAGTCACAAC
2601 TTTCTGCTTT AGCAAAGGGG GTTAAAACAC ACCCAGTGCT AAATATTGAA
2651 CATGCGGAGC ATAAAGTGGA TGCCACACAT GCAAGaATTA ATTTGGGAAA
2701 GTTCTGCTAT AAGCTTTTAA TTAGAGAAAT AGCAGGTGTC ACTCAATGGA
2751 ATGAAACTTC TGACATTAAA CATCATATCG AGCAAGCAAC AAACCAGCTC
2801 AATATTCATC TCAAAAAAAC CATTGGAATC AATCCTTGCC TAATGCTGCC
2851 AGGCAACTAT GCCAGAATTT TGTTTTCCCA GAAAAATGAG GGCCACATCC
2901 TTTACCTTCT AAAATCTGTT GAAAAAAGAG AAAAaTTTGC CAAATTATTA
2951 ACACTTGTAA GATTTATGCA CAAGATATTT AGTTCCAGCA AACCAATGGA
3001 AGAATTCCCT GAGGAGTGGT CACACTACAA AAGCAGAGCA GTTGAATTTG
3051 GGAAGCACCC AATTGCAAGT TACCCATATG CAAGGTGGAG CAATTATGTC
3101 CACAAGACCA TTGAGCATGT GCAGGAGGTG ATAGAAAGCC ATGGAACACT
3151 GGGAGGATTT TCTGGAGAAG GAAATGAAGC AGGAAATAAA ATCTTTCGAC
3201 ACCTTCGCAA AAATCATTCA AGAAAAACAG GAACTTTTGA AAGTGTTTCT
3251 GATGTGCTCA AAATGCATTG GCTTTATTGC AGCTTTAAGC TAAAAACACT
3301 CAGTGAAGTT GCAAGAAGAA AATACAAATG CAGTATATGT AAGCAAAATG
3351 GCCATAATTG CAACACTTGC CCATCTGCTC CAAAATAGTC ATCGATATGT

<<<FcoRAG1L ORF ends

3401 ACAAAATTTT TGAATTATTT ATATACCCAA AGTATTTATA CAGCAGCAAT
3451 TGTTGCAGTT ATTTATACTT GCTTACAAAC TATTAATCAT TCTCTACTTT
3501 ATTTATATAC TTATCTGACT ATTTATTTAT TTATTTATTT ATTTACTTAT

>>>FcoRAG2L ORF ends

3551 TGAAATACAA AAGTCTTTTG TTTACTTCTT AGTGACTTTT TTACATTTGT
3601 TGCAAATAAA TTTTCCCTTG GGCATGACTG CTAAGCCAGC ACAAAACTGA
3651 TGAATCCACA TTTTACAGGC ACCCTCACAT TTTACCCATG GCACTGGAAT

```
3701 AATAAGGTCA GCTTGATTAA TTGTGCACTT GTCACCAAGG TCACAGTTAT
3751 CAGCTTGAGG TATAGTTTTT GTAAACAACA GAATTTCCTT TTTAGTCCCC
3801 CCACAAATTA AAAAAGTTGA ACTGTCTAAC AGGAACATAC TGTGACCAGC
3851 AGTGGCATGT TCAGGTTTTG CAGTCTTGCT GAGGTAGACC TGCTTGTCAA
3901 CATCAAGTAC AAACAAACAT GAACTTTGAT TTGGTTTAGT AGAAATTGTT
3951 CTAGCATTAG CTTGATAGCC ACCAAAAATA TGGATGATAT TATCACAAAT
4001 TGTGGCAGCC TGCGAGGACA AATATTGCGG TGCAACCATG GGAAATTGTA
4051 CAGAAGAGGA CAAAATTGTA TTTGTGGCAA AGTTAAATTC TACTGATATA
4101 ACTTCATGAA TGCTGCAATA CTCAACTGCA TGGTCATCAG CCAGTTGAAT
4151 ACCCCCGACA ATGTAAAGAG TGCTTGTTGC TGACCGAAAC ACTGCAGCAT
4201 GATAAGCTCT TGGAGGGATT TCCTCAGACA AGCATGTCCA AGAATTCTCC
4251 ATAATGTTGA AGGCATATAG CCTTCCATCC TTACATGATT GCCTAAAGAT
4301 ATTGTCCACA CCCTCAAGGC GGTTTTCTAA GGTGACCCCA CCAAACAGAA
4351 TGGCTCTTTT ACTTGAAATT GTAGTAAAAC TATGCCCTGT TCTTGGGGAA
4401 GGAACTGTTC CCATTTGAAA AGCTGAACCT TCAGTTGAAG GTGTTTTGTT
4451 CCCAGTTAAA GCAGTCAAGT CTATTTCAAA ATTCAAAGAC TCAGGCAACT
4501 TTTTAGAACC ACTTGCTGTC CTTTTATTTT GCTTGTCTTT AGCAGTAACA
4551 TTTATTTTCA TCAGCTCATT CGTACATGTC ATGGTTCTCG TATCTATCCC
4601 TCCCCACAAA AGCAAAACAT ACTCCTCAGG ATCAGGCGGT AACTTTGACC
4651 GTGCATTGAT GAGCGTACTC CCTTGCAAGG GAAACAAAGA TGATCCTCTC
4701 AATTTATCGG GTCCAAACAC ATTGCAAGAA TTAATTTCAA CATCACTACT
4751 GGATAAAATG AATTCCATTA GAGTAAGGGA ATTCCCTAAC TCCCATGTGG
4801 ACGGCTCATT GTATCTAGCT CCTCCAAGAA ACAATATCCC CACATGTTTC
4851 TCTTCGGATT CCTTCGGACT TTTTTCCCAT AAAATACACG AACAGTGTCC
4901 TTCGGGTAGG AAAAATTGTT CCTGACGGGC ATTCAGTAAG TGCTTTTTCC
4951 GACTAACCTT TCTGTTATCC GCCGAATACT CGAGTAAAGA AATAAATTTG
5001 AAATCTTCTA AGTCTACACT CGACCTTCCC TCTGAGTCCG CCATCTTGTT
                                FcoRAG2L ORF begins<<<
5051 TCTAAAATTT CACACCACAG CGCTTGCGCA GTAGACCGGC CGCGCACTAA
5101 AAAGCCTGGA CCGAGCAATG TGAAATTTTC TAAGGTCAGT GGGCAGCAAT
5151 TTCGTAGTG
      <<<3'TIR
```

```
FcoRAGL_5'TIR1 : CACTATGAAA------------AGTCCTTATAAATATTCATTTATGCAAATTAGCTCGT :  47
FcoRAGL_3'TIR1 : CACTACGAAATTACTGCCCACTGACCTTAGAAAAGTTCATATTGCTCGGTCCAGGCTT :  58
FcoRAGL_5'TIR2 : CACTATGAAA------------AGTCCTTATAAATATTCATTTATGCAAATTAGCTCGT :  47
FcoRAGL_3'TIR2 : CACTACGAAATTGCTGCCCACTGACCTTAGAAAATTTCACATTGCTCGGTCCAGGCTT :  58
                 CACTA GAAA            G CCTTA AAA  TTCAt T        T     C T
```

Figure 2 Alignment of 5'-and 3'-TIR of FcoRAGL showed their conserved elements.

5. FcoRAG1L protein sequence

```
  1 MDVKCHKEKL ASFCRRCGNF FDTNPATSRH KPKLAVTFSK ELQAVDGLDV
 51 SNDSEEMHPP SLCRCCILKL ERFKKSRNRN DRQLKFPGDK GKCCVFLTHN
101 SYVKEGSCPI CCFNLRQSPR KKRPHEPDNS PSASGNAREN SEGAAAGGKK
```

151 KPPFVRQVFT SSAESSTSGM ASSTSTSETP TTKIFTEQEP STAAGPFEGD
201 TFELTGIPLD RFVEQQFAEI FQCNICMDIP TDAVILSNCH HVYCEHCIQR
251 WLACNGVCPS CRNLVELDDV LPLRQQMLTI FDMLTLHCKY SENGCEEKLT
301 VNEIAEHEKS CRFSKKKLKR GAYNKTKLFD ISRQYSKRGR LRNMFDNLSE
351 FCQSHNEDAE DVLFSMLTTT LHDKGKTELA KKVEYLWTEQ TDNDLTVDEC
401 LANRINMLQT KNQYKKQYDF LNNKGQFFFK PLYSLDKAED TYLPSYVNYK
451 IKDNMDNIIF NHSACKSEPI NLMADFREAL PQFPVPNVVG VRWSYRNALA
501 KSLQELGSEI DKGLDNCGIT GNPTLQVLIK DGGDGLGDVS MYKEKGDRYL
551 EDKAYRYSFC VLKITVDREN ERVVIWEEDT PGSVRTNKTL IEAVCDENQT
601 AAMVACVVPV EREREQIANN LISVETGTLW RNFKVEFINS MEDEKRARAD
651 GGLQEAGSRF MCDLCYATQD SAKSDLGTFK TCRTLEETKQ IADLLHFNPS
701 KLTQSQLSAL AKGVKTHPVL NIEHAEHKVD ATHARINLGK FCYKLLIREI
751 AGVTQWNETS DIKHHIEQAT NQLNIHLKKT IGINPCLMLP GNYARILFSQ
801 KNEGHILYLL KSVEKREKFA KLLTLVRFMH KIFSSSKPME EFPEEWSHYK
851 SRAVEFGKHP IASYPYARWS NYVHKTIEHV QEVIESHGTL GGFSGEGNEA
901 GNKIFRHLRK NHSRKTGTFE SVSDVLKMHW LYCSFKLKTL SEVARRKYKC
951 SICKQNGHNC NTCPSAPK

6. FcoRAG2L protein sequence

1 MADSEGRSSV DLEDFKFISL LEYSADNRKV SRKKHLLNAR QEQFFLPEGH
51 CSCILWEKSP KESEEKHVGI LFLGGARYNE PSTWELGNSL TLMEFILSSS
101 DVEINSCNVF GPDKLRGSSL FPLQGSTLIN ARSKLPPDPE EYVLLLWGGI
151 DTRTMTCTNE LMKINVTAKD KQNKRTASGS KKLPESLNFE IDLTALTGNK
201 TPSTEGSAFQ MGTVPSPRTG HSFTTISSKR AILFGGVTLE NRLEGVDNIF
251 RQSCKDGRLY AFNIMENSWT CLSEEIPPRA YHAAVFRSAT STLYIVGGIQ
301 LADDHAVEYC SIHEVISVEF NFATNTILSS SVQFPMVAPQ YLSSQAATIC
351 DNIIHIFGGY QANARTISTK PNQSSCLFVL DVDKQVYLSK TAKPEHATAG
401 HSMFLLDSST FLICGGTKKE ILLFTKTIPQ ADNCDLGDKC TINQADLIIP
451 VPWVKCEGAC KMWIHQFCAG LAVMPKGKFI CNKCKKVTKK

Fungi

Rhizophlyctis rosea

7. RroRAG1L_0490

```
>NJGN01000490.1:9496-17392 Rhizophlyctis rosea strain NBRC 105426
Scaffold491, whole genome shotgun sequence
   1 ACTCACACAT ATCCGTTTTC AAAGGGGATA GGCTTTCTTC TAATGCCATT
  51 ATTAGTCATT AAAGTGAGGT ATGAGGTAAG TATGAACCCC CACTATCACA
 101 CTCATTGTTG CATTGCGACT GAACGGTTTT CGGAAAACAG AGCAACCTAT
 151 ATAATTACCG TTCTATGTTC GGCGCAGCCC ACTCCATATA CAAAGAAGGC
 201 GGCATCCGCT CTTTCTTCTA CGGTTTTGGG CCTACAGCGC TAAGAGATAT
 251 GCCGTATGCA GGACTTTATG TGTTCATATA TGAGAAGTGC AAGAATGTTA
 301 TCAGCCGTAA GTGTTTTCTT CGTCATTCCC CTGCCCATTA TTCTCGCGTC
 351 GAACTTATGT TCAACCGATC CATAGTCGCG ACGCAACATT CGACAATGCC
 401 CCTCGCAACA CCGACAATAA ACATGACGTC GGCGGTCATT AGTAGCATCG
 451 CATCAACATA TATCACTCAA CCGTTTGATA TGGTCCGTGT AGTCAACCTT
 501 ATACCTCGTG ACCCACTGTA TCTCTCGCTC ACCAATTATA GGTCCGAACA
 551 AGAATGCAAC TCCGGCCAGC TGACTACCCT TCAATGGGCA ATACATTTAG
 601 ACTGGTTTAT ACCGTGAGTT TGGGCTTCAT CTGTCTCGCT GATTCTCCTC
 651 CTTGAACTGA CCGACTAAAC GACGAAAATG ATGACAGAAC GAAGGCCTCC
 701 TCGGTTTCTT CTCTGGAATG GTCCCTCGCC TAATGCGGAA AACAATCAGC
 751 TCGGCGATTA CGTGGACGGT GTATGAGGAG ATTGTCCGAG CTTGGCGGTA
 801 GTGCATGCAC TCTAATGTGG GTGTATAAAG TAACTTTTGA CTTGGTTTTG
 851 GTGTTGGGAT TGCATGCCGC TGTGGCGTAT TCTGGTGATT TGAATTTCTT
 901 TGTACATAAC GGCGTAAACA AAATAGAAAT GTATATACAA CGTGTGAAAA
 951 GTTGCGCAGG TGCAGACGAT AGTCATTTTT CAACATGACG GATGGGATAG
1001 ACACAAGGTA CATCGTGGGG GTATTTCCCA GGGGATGTGA CACTCCACTC
1051 CCTTTGATGG GGATCATCGC AGTAGGCGGA GTACCGGCGG AGATTGCATT
1101 CCCTTGAGCA AGGAGAACCG AGTTCGCAGT GGCATACGCC GCCCTCAACT
1151 GCTCATGTCG TAAACCTTGC CAATCGGCGT CGCGGTGGAC CCAAAGCACA
1201 TGAACGAGAT ATGCGTTGTG TTTTCCTCAA GCTCATTTCA AATCGCTGAG
1251 ATAGTCCTTG GTTAGATCTA CATAGCCATT AAAAAAAATG ATGTCAACAC
1301 ATGTGGAAGG TAAGACTGAG ATACCAAAAA TAAATGGAAT TCAATACGTA
1351 CCGATACCCT GCAAATGCGT ATTAGTGATT TTGATCCTCT TATTGTTTCG
1401 TTTCCTCTCT TTAGGTTTTA TTTGCTGTTT GATGGCGGTG CTGAAGACTT
1451 CCATTGATTT TAGGCCGGCT GTAGTGTATT AGATGATTTT TGGATTCAGG
1501 GATGTAATGG GTTGGTGATG AGCGTTGAGG TGGAGATGGG GTTGCAGAGA
1551 AAACCAAGAG CGGGAAAGAA TAGGTGGATG ATTTTTTCTT ACCTCGTTCG
1601 AGCTCTTTCG CCAAATCCAG TTCTGTTGCG GGCATTTTGA GGTTGTGCCA
1651 GTATGTTTTG AACTCTATTT TGTGGTGGGA AACGGTGAGT GAGCATGTAA
```

1701 AGGAAAAAGA GAGTAGACGG GGAAGAAAAA GATTGTTACC GTCGGGCATG
1751 ACGACGTTGA GGGATTTGTC GTTCCAGAAG AGAGATTTGG GGGAGTTGTC
1801 TTTGGAGCGG ATGACGAGGA TTGAGCCTTC TCGTTCGAGT TCCTGCGTGC
1851 AGAGCATTGT TGGATTGAGG TTTCTCCAAT GGTGGGAGCG GCAAAAACAA
1901 CGAACCTGCA CCGCTTGCAA GACCTCTGGC CATCCTTCTT TTAACTCTTT
1951 GTAATCCTGT GCGAGGTTCT TTTCTTTGTT CTCTTTGAGG AGGATGAGGA
2001 GTTCTTCTTT GGTGCGGATT GCGTGGGTGT GCTTTTGGGG GGGGGATGGG
2051 GTTTGTTCAG GTTCTTTTGT GAGTAAGTTA CACTGACGGG TTAGTACCTT
2101 GTACGAATAG GTCCTTCTTT TCTCATCCCA GATGATCTTC TCGTTCTTTT
2151 GCAACGTGGG CCATACATCG GCTGGAGGTG GAGGGCCGGC GATGGCCTTG
2201 CGTATTTGAT CTGCGGTCCA AGGTTTGTGT TCGTTCTGCA AGTTTTTTTT
2251 GTTTGTTTTT CAAAATATGT ATCAATTTTC TGATCCACTG AAAATTGACG
2301 TGATGGACAA ATAGGCTACC TTGAGAAGCT CTAGATAGGC GAAAATGTAA
2351 TTGAAGGTTG TGGGGTTGGG TGGCAGCGTT GGACGGTCCA TTTGAGCTGT
2401 GGGTCATGTG TGCCAATTGA CTTTGAGCAA GCAGTTGGGG TATAAAGAAT
2451 CACCAGAGAT GGAAGCTTAC GGGCTACTCC GGGTGGCATT GTATATTCAA
2501 AAGTTTATGG AGACAGTGGG AACGGAACAG GGCAGACTTT CCAGGTCAGG
2551 ACTGATTGGC ATTGAAAACT CAATTACAAT TGTGCGTTTA GTTGGAACAC
2601 GTTTTTGACG AAACTTACCA GAAAGGAGCT TCAATGATCT TGTGATGGGT
2651 ATGTTGTTAC TTGACCAGTC TAATGGAATA TGATGTGAAC GTGAACGTGA
2701 GTACTAGACG GATGAGAAGA TGTCTCGATG GGCAGGTTGG GAATTGGACG
2751 GCGCACACAC CGCGACTTTC AAATAGAACT TGCCTGCCAC CAATCATTTT
2801 GGGGGACGGC CAAGCCCAAC CTGGCCTGCA CTGGCTCCGA TCTTCACAGG
2851 CACTTCCCCA CGTTCACTTC CTTCCCCCCT ACCATTCCCA AAAACATTCA
2901 CACTCCAAAT ACTATGTCTG AAAATATTCC CCCCAACACT TTTCTTTTTC
2951 AAACCTCCCC GGGCCCCAAA AAAAAGTCCT TGTCCCAATC TTTGAGCCCC
3001 CATCAACAGC GCCGTCGGAC ACGCTCACTC AAAGACATAG TGCAGCAATG
3051 GTGCCAGGAA AATAATGAAG ACTACCGCTC CAGCTTGATA TATCTGTTAT
3101 CATCCGATCT TCGAGCCAAC GGGGAATACA CACAAAGCGA CCTTCTTAAA
3151 TCTATCTTCA CCCACAACAA CTTCAATTTC AAACTTTCTC ATGAACAAAG
3201 CCTGGCCCTA CGCATCGCAT CCAATTTATC TTACCAGGAA TATGAAACTG
3251 TACGTGATGT TTTACACTCT TGCCTTGGAT ATGACATTCT TTCCCGTTAT
3301 TCTGAGACGG TCTCAGCCGG AAGTGTGTTG CTGCCGGGGA AGTTGGAGGA
3351 CGGTATATGG AAACCATCCA TGGTCAATTT GACCGAACAC ATCTACGATG
3401 ATGACGGTGG CATAGCAGCA ATTCGCCATT ATGGATGGAG ATGGGATATA
3451 GAATTGGCGG TCAAGTCACA TATACGGGAA CGACTGGAAT TATTGGAAAG
3501 TGAGGGGAAG TTGCAAACTG TACGGGAATT GGAAGTCGCA ATGAAGGTGG
3551 GGGCGGATGG GATGGGCTCA ACAAGGGAAT CTTTGGATAT GGACTCATCC
3601 GGAAAAGCTC TAAGATTCAA TCAATGTTTG CTTTGGGTGG GAGAAGATGA
3651 CGGGAGTCCA TTGTATCATG GGGATTGGAT ACTTAGTAAA TTAGATAGAG
3701 GATGGAGTCC CATATGGCGG GAAGAATCTC CCAACTCAGA GGCACGTATG
3751 CGACCAATAA TGGTCGTCAA CGGAGACGAG AACGACGATA TTTTTCTCAA
3801 ACATTGTCTC GCTCCCATCG AATTTGCTTA CCATACCCTA CGTTCTACTC
3851 CTCTTCGTTT GTCCATAAAC AATATCGACT TCAACATTAA ACTCCGCATC

3901 ATCAACACAA TGTACGATGA AAAGTTCCGC AGAAAGATCA TCGGCATGCA
3951 GGCAGCAGGC TCTCACTTTG TCTGTTGTTT ATGTCAAGCA TATCGTCCGG
4001 AAATGCGTGA CAGAGTGGGG ACTTACCGTT GGGAACGAGA ACTGGAAAGT
4051 CATAAATCTG TGGCTAAAGG TCAAGTAGTT GCGGGGGTGA AAGATGGGGA
4101 AATGGTATTT CTGTGCGGGG ATTTGTTGAA TTTAGTCATG GATGCCACAC
4151 ATGCTGATAT TAACAATGGA AAATGGTTTG AAAGGGTATG TTTTATTGAT
4201 GTTTGTAACC ATCCAATTAA AATAAAAATT AAGATTATCA GAACAGTTTT
4251 TTTCCAGATT GCTAGCGGGG GTCTTAAAAT GGGAACTTAC AAGTCATGAG
4301 GAAAAGACTG CTGTGGATGC GGCCAACACC ATCATCATTG ACATCATACG
4351 TACAGATCTT AACACACCGG TACCTATGAT GCGGGATGGC AAATTTGGTC
4401 GTTGTATTAT TAGTGGAGAT TTTGAAGCCC TTTTCCTTCC ATTACTACCT
4451 GAGTCCATCC AACGCACCGT CAGTCTCATT TTTCAAGGAT ATCGTGAACT
4501 TCGCACATCT TGGCGAGCGG ATTGGCCATC TCTTGAGCTC TTTAGAGAAG
4551 AAGAAGATGG ATATAGTATA TATAAAAGGA CAGCGGTGGC ATGGGCAGTT
4601 TTGTTACGGG ACAATTTTAT ATGGGCTGAA TGGAATAACT ATTTTCATTA
4651 TATGATAGAA CATGTAGATG AATATATGCG TCAGTGGGGT ACCGTGGGGG
4701 GTCTTTCTGT TGAAGGACAA GAGGGTGGTA ACAAAGTGCA TAGAATGGCG
4751 GAAAAAGGAC ATGCAAGAGC CAATTTAATT GATGGCAATA AGGACATCCT
4801 GGTGTGGATG TGGTATCATA GCAGCGAGAG GTTACAGAGA ATGGCGGTTC
4851 GAGCAAGGAA GATTTATACT TGCTCAACAT GTGGGGGCCG TGGGCATATA
4901 TCTAGCAATC ATAGTTGTCC TGCAAGAAGG ACATAGGGCT TCTTAAATAT
4951 TTTTTTTTTG TATGTGTAAT TATGTATTTC AAATATTTGC TTGTTTTTTT
5001 TGCAAGATTC GCAAAGTTCG TAAGAAGTGG GTGTGTGGGT CAACGGTCCT
5051 TGATGTTGAT GGAATCCAGA GGTGCGTGTC GCACATCAGA GCCACTGCCA
5101 GGTGGTGTCA GATCCACAGC AGAGTGGGTC AGGAGCAAAC AGGGCTGTCA
5151 TAATCAAGCC TCGCCAAGGA GGCGAATATG CTGAAGAGTT GCGTCGTGCG
5201 CGCACGACGA CTGACAACCA GACTGGCACC TGCTGCGGGG GGCGTCACAT
5251 GTGGCTTCTA TCGAGCCCTA CGCCACCAGT TTTGATTACG CTATCAAAGT
5301 ACCAGAAACT GCGCAGCATT GGACCAATCA ACATCGATTC ATTTCTTTTG
5351 ATCGGCAAGC CAAAAAAGTC TATAAATACC CCCTCTCCCC TTCACCCCAC
5401 CAGAAATCAT TCACACGTCA CCTCTTGCAA ATAGCCATCT ACCAGGAAAG
5451 CTACTCAAAG TTGTGGTCCC TCACAGCAGA CATTCTGCCC AAACACCCAA
5501 CCTTCAGCCG CCATTCCTCG TATTTGGCAT ACAGCAGAAA TCACCCTACA
5551 CAAACCGATG CGAGACAAAG TCGCTTGGCA CCGAAGCACA CAGGACCGTC
5601 CTCCCTGAGT TGATTCACTC AACCCACAGG ATTTCCAACC TTCCAGTAGT
5651 GTATATTTCG CCGCCAACGC ATCGAACACC AAACACCCAT GTGCTTTGAG
5701 CTTCCGTCAG GTCGTTCGGT CATGAGGCGT TCCTCTATAT TTCATGTCCT
5751 TTCCGTAGTG TCCCCCTTGT GGGGATTTCA CGCCACTATG TGGTGGTTGC
5801 GGTTTGGAAA AACCATAGAG TTGACATTTC CTTTTTTTAC ATATTGATTT
5851 TTATTTTTAT GATGAGGTGC CAATGGGTTC GATTCCCGTT GGTATCAATG
5901 CTTTTTGCAT TTACCCCACA CGCAACAATC GTTTTTTTAA AGGATGAGAA
5951 ACAATATCAG ACCTGTTCCG AACAATTGCA TGTTGGACAG CCATATATTG
6001 CCTATTTATC TCTGATAATA TTCGATTTGT TGCATTTGAG GGTGACCAGT
6051 GTCACTGGGG TCTTTTTGGT GTTCTCATCA TCAGAACATC CATAAGTGCT

```
6101 CTCACACACA AAGCATATTG CACAATGGTC TCCAACCCTG GCTTTAGTAT
6151 CGTCCCATGC TTTCGCCGTA AAGGAACTGA AGGAATGTCG AGAAAAAGTA
6201 GTAAAAGTAG AAAATCGGAG ACAATAGTTG CAACTTGCAG ATCGCGCGTC
6251 CGTGTTGGTG TTGGGAGGTG AGCGAGTAAA GCAAAAGTCA ACATCACCAT
6301 ATCAAAACGC AGACAGGAAC AACGAAGTCC CCATTTGCAA ATTGTAAAAT
6351 GGTTGCCCCC TGATCCAAGT GATCCATGAC CAATGTCCAG ATCAGATATG
6401 TCAACTGAGA GACTGAGTGG CCACCAAACA ATGTGAAAAA GCCGCGAGGT
6451 CCTTCACAGA TGTCTCGTTC TGCCATCAGA GATGATCAAA CATCACTTGA
6501 GAACCTCATC CTCCCCTTCT GTCAATATCT CTGTTCCTCG CGCAAATTTC
6551 CCCCCAACCC CAACGGTGAC AATCTCAACG TCCGACACTG CCCTTCTTAC
6601 CACACGATCC GTTGTGAATG GTTCACTCCA GATGAGATCC TCCCCCCTTC
6651 CAATCGCCCC GCTCCAGATG CTTGTTCCTC CCACTTTGCC CTGCCCACTA
6701 CCCAACTCCA GTGACTCCAC TCTCCTTCAT CTCTCCCAGT GCTCCATATT
6751 CCACCACCAC ACACCTCAAA ACCCCTCCAC CCCCAACTCC TCCACCCTCC
6801 AATCCAATCA AAATCATAAA AAAAACACTC TATTTACAAC CAAACTTTCC
6851 CAACCCGCAA TTTTTACAGC CCCGCTCGTT CTCTGCACCC CCCCCTCCCA
6901 TCCCATCACC CACCCNNNNN NNNNNNNNNN NNNNNNNNNN NNNNNNNNNN
6951 NNNNNNNNNN NNNNNNNNNN NNNNNNNNNN NNNNNNNNNN NNNNNNNNNN
7001 NNNNNNNNNN NNNNNNNNNN NNNNNNNNNN NNNNNNNNNN NNNNNNNNNN
7051 NNNNNNNNNN NNNNNNNNNN NNNNNCCGAT CCTCAAAGAA CGTCTGCCGC
7101 ATCTTGAAAA AGACCTTACA CGTCAGCAAA CTATCACTCC CTGCCTGGTG
7151 TTGGGGACCT ATTCTCGCGA TCTGGAAAAA ACGAAGTTGC AAAGAAACCG
7201 TTAATTAACG AAATTCTGGA CGGTGGGGGA TTGAAGAAAA ATGAACTCAA
7251 AAACGAAGGG CCGTTTACCT GGAGATCGTC AGCAACATCC TGTAAGCCCC
7301 CTTTGAGGTT TTTGCACGAC TTCATAAGAT ATTTGATGTC GTATATGCAA
7351 GGGAAGTAGA TTTTCAGGAG GTCAAAGAAG TCGGATTCTT CGGCGGGGAG
7401 GGGTTGGCAG GTGAGGACTT TTAGGAGGTA GCCAAAGTCG TAGCCACTGA
7451 AGGTGAAGTT TAGTGGTCAC TTCCAGAGTT GGTTCCTCAA GATACGCGTT
7501 TTGTTTATGG ATGGTACCTG TGAAATGATA TCCACTTAAC GTCGTCTAAT
7551 AACACGAAAC CAGAGGAGAT GAGCAATTCG CCAAAATGCT CCACGTCGAT
7601 TCCGAATTCC TCGTGTTTTT TGAAGTCGAT GCCGGATTTT GTGAGCAGGT
7651 CGATGGAGTC TTGGGCGTAC ATGTCATCAC TATCATAATT GATTCAAAAC
7701 GAGACAGTTG AGTCCTCTGA GGGTCCGCCG TGGAAAAAAA CAAGGAAAGA
7751 TATCCGTCCA CCATACCTGA GGTTGAACTT GAAATTGAAC TGCCAAGTGC
7801 AGACCTCGGG CATGTTCCCC TGCTCATCCG CAAACGTGAT CCCCAATTGA
7851 ATAATCTTCA ATAGGTCCAC GTTGCATCTT AGTGTCTGAT AGTGATA
```

The transcript and protein sequences of *RroRAG1L_0490* were predicted by AUGUSTUS.

```
NJGN01000490.1:9496-17392 AUGUSTUS transcript    2728 5434 0.12 +  .  ID=g2.t2;Parent=g2
NJGN01000490.1:9496-17392 AUGUSTUS start_codon   2728 2730 .    +  0  Parent=g2.t2
NJGN01000490.1:9496-17392 AUGUSTUS intron        4186 4246 0.85 +  .  Parent=g2.t2
NJGN01000490.1:9496-17392 AUGUSTUS intron        4922 5006 0.24 +  .  Parent=g2.t2
NJGN01000490.1:9496-17392 AUGUSTUS intron        5020 5069 0.4  +  .  Parent=g2.t2
```

NJGN01000490.1:9496-17392 AUGUSTUS CDS 2728 4185 0.97 + 0 ID=g2.t2.cds;Parent=g2.t2

NJGN01000490.1:9496-17392 AUGUSTUS CDS 4247 4921 0.26 + 0 ID=g2.t2.cds;Parent=g2.t2

NJGN01000490.1:9496-17392 AUGUSTUS CDS 5007 5019 0.4 + 0 ID=g2.t2.cds;Parent=g2.t2

NJGN01000490.1:9496-17392 AUGUSTUS CDS 5070 5431 0.53 + 2 ID=g2.t2.cds;Parent=g2.t2

NJGN01000490.1:9496-17392 AUGUSTUS stop_codon 5432 5434 . + 0 Parent=g2.t2

coding sequence = [atgggcaggttgggaattggacggcgcacacaccgcgactttcaaatagaacttgcctgccaccaatcattttgggga
cggccaagcccaacctggcctgcactggctccgatcttcacaggcacttccccacgttcacttccttcccccctaccattcccaaaaacattcacact
ccaaatactatgtctgaaaatattcccccaacacttttctttttcaaacctccccgggccccaaaaaaaagtccttgtcccaatctttgagcccccca
tcaacagcgccgtcggacacgctcactcaaagacatagtgcagcaatggtgccaggaaaataatgaagactaccgctccagcttgatatatctgttat
catccgatcttcgagccaacggggaatacacacaaagcgaccttcttaaatctatcttcacccacaacaacttcaatttcaaactttctcatgaacaa
agcctggccctacgcatcgcatccaatttatcttaccaggaatatgaaactgtacgtgatgttttacactcttgccttggatatgacattctttcccg
ttattctgagacggtctcagccggaagtgtgttgctgccggggaagttggaggacggtatatggaaaccatccatggtcaatttgaccgaacacatct
acgatgatgacggtggcatagcagcaattcgccattatggatggagatgggatatagaattggcggtcaagtcacatatacgggaacgactggaatta
ttggaaagtgaggggaagttgcaaactgtacgggaattggaagtcgcaatgaaggtgggggcggatgggatgggctcaacaagggaatctttggatat
ggactcatccggaaaagctctaagattcaatcaatgtttgctttgggtgggagaagatgacgggagtccattgtatcatggggattggatacttagta
aattagatagaggatggagtcccatatggcgggaagaatctcccaactcagaggcacgtatgcgaccaataatggtcgtcaacggagacgagaacgac
gatatttttctcaaacattgtctcgctcccatcgaatttgcttaccataccctacgttctactcctcttcgtttgtccataaacaatatcgacttcaa
cattaaactccgcatcatcaacacaatgtacgatgaaaagttccgcagaaagatcatcggcatgcaggcagcaggctctcactttgtctgttgtttat
gtcaagcatatcgtccggaaatgcgtgacagagtggggacttaccgttgggaacgagaactggaaagtcataaatctgtggctaaaggtcaagtagtt
gcgggggtgaaagatggggaaatggtatttctgtgcggggatttgttgaatttagtcatggatgccacacatgctgatattaacaatggaaaatggtt
tgaaaggtttttttccagattgctagcgggggtcttaaaatgggaacttacaagtcatgaggaaaagactgctgtggatgcggccaacaccatcatca
ttgacatcatacgtacagatcttaacacaccggtacctatgatgcgggatggcaaatttggtcgttgtattattagtggagattttgaagcccttttc
cttccattactacctgagtccatccaacgcaccgtcagtctcatttttcaaggatatcgtgaacttcgcacatcttggcgagcggattggccatctct
tgagctctttagagaagaagaagatggatatagtatatataaaaggacagcggtggcatgggcagttttgttacgggacaattttatatgggctgaat
ggaataactattttcattatatgatagaacatgtagatgaatatatgcgtcagtggggtaccgtgggggggtctttctgttgaaggacaagagggtggt
aacaaagtgcatagaatggcggaaaaaggacatgcaagagccaatttaattgatggcaataaggacatcctggtgtggatgtggtatcatagcagcga
gaggttacagagaatggcggttcgagcaaggaagatttatacttgctcaacatgtgggggccgtgggcatatatctagcaatcatagttgtcctattc
gcaaagttcaggtgcgtgtcgcacatcagagccactgccaggtggtgtcagatccacagcagagtgggtcaggagcaaacagggctgtcataatcaag
cctcgccaaggaggcgaatatgctgaagagttgcgtcgtgcgcgcacgacgactgacaaccagactggcacctgctgcgggggggcgtcacatgtggct
tctatcgagccctacgccaccagttttgattacgctatcaaagtaccagaaactgcgcagcattggaccaatcaacatcgattcatttcttttgatcg
gcaagccaaaaaagtctataaataccccctctccccttcaccccaccagaaatcattcacacgtcacctcttgcaaatag]

protein sequence = [MGRLGIGRRTHRDFQIELACHQSFWGTAKPNLACTGSDLHRHFPTFTSFPPTIPKNIHTPNTMSENIPPNTFLFQTSP
GPKKKSLSQSLSPHQQRRRTRSLKDIVQQWCQENNEDYRSSLIYLLSSDLRANGEYTQSDLLKSIFTHNNFNFKLSHEQSLALRIASNLSYQEYETVR
DVLHSCLGYDILSRYSETVSAGSVLLPGKLEDGIWKPSMVNLTEHIYDDDGGIAAIRHYGWRWDIELAVKSHIRERLELLESEGKLQTVRELEVAMKV
GADGMGSTRESLDMDSSGKALRFNQCLLWVGEDDGSPLYHGDWILSKLDRGWSPIWREESPNSEARMRPIMVVNGDENDDIFLKHCLAPIEFAYHTLR
STPLRLSINNIDFNIKLRIINTMYDEKFRRKIIGMQAAGSHFVCCLCQAYRPEMRDRVGTYRWERELESHKSVAKGQVVAGVKDGEMVFLCGDLLNLV
MDATHADINNGKWFERFFSRLLAGVLKWELTSHEEKTAVDAANTIIIDIIRTDLNTPVPMMRDGKFGRCIISGDFEALFLPLLPESIQRTVSLIFQGY
RELRTSWRADWPSLELFREEEDGYSIYKRTAVAWAVLLRDNFIWAEWNNYFHYMIEHVDEYMRQWGTVGGLSVEGQEGGNKVHRMAEKGHARANLIDG
NKDILVWMWYHSSERLQRMAVRARKIYTCSTCGGRGHISSNHSCPIRKVQVRVAHQSHCQVVSDPQQSGSGANRAVIIKPRQGGEYAEELRRARTTTD
NQTGTCCGGRHMWLLSSPTPPVLITLSKYQKLRSIGPINIDSFLLIGKPKKSINTPSPLHPTRNHSHVTSCK]

Query : RroRAG1L_0490

Subject: AanRAG1L

AanRAG1L

Sequence ID: Query_52693 Length: 850 Number of Matches: 1

Range 1: 450 to 841 Graphics   ▼ Next Match ▲ Previous Match

Score	Expect	Method	Identities	Positives	Gaps
94.0 bits(232)	9e-24	Compositional matrix adjust.	91/405(22%)	173/405(42%)	33/405(8%)

```
Query  329  IWREESPNSEARMRPIMVVNGDENDDIFLKHCLAPIEFAYHTLRSTPLRLSINNIDFNIK  388
            ++ E  P+S     P+++V  +EN+D      L   +  +  L  + + L + ++ F+ +
Sbjct  450  LFLEPKPSSNQVSHPLVLVYANENNDSETGLALVATKAEWAELDGSTIELDVGHVLFHAE  509

Query  389  LRIINTMYDEKFRRKIIGMQAAGSHFVCCLCQAYRPEMRD--RVG---TYRWERELESHK  443
            + +  TMYD+K  R I G     +H+ C  C   + + RD   VG   +  ++R +ES+
Sbjct  510  IDVYPTMYDKKLERSITGQMGTTAHYNCTDCTMTQDQQRDPTLVGQGESITFKRTIESNA  569

Query  444  ------------SVAKGQVVAGVKDGEMVFLCGDLLNLVMDATHADIN-NGKWFERFFSR  490
                        ++A+ ++ A     +++    DL+    D  H D+N +G W E
Sbjct  570  AAQELAVRPEPGTMAQRRMAAQGMKTDVIMAALDLVRSKTDTLHTDVNGSGSWIESIVIH  629

Query  491  LLAGVLKWELT-SHEEKTAVDAANTIIIDIIRTDLNTPVPMMRDGKFGRCIISGDFEALF  549
              A + W +T SH+E  ++  A  +  D +R  +     MM  G +GR +++ + E +
Sbjct  630  ENAQIYTWSITASHKEAYSI--AKRLFHDHLRVSIRQHSTMMIAGNYGRALVAEENERVV  687

Query  550  LPLLPESIQRTVSL-IFQGYRELRTSWRADWPSLELFREEEDGYSIYKRTAVAWAVLLRD  608
            + L+    +R   L I + +R +RT W +          +    S Y R       L+ +
Sbjct  688  VSLIANVERRQKVLEIIRCWRAMRTVWLS-------ATSDATALSSYDRHRRRLHDLITN  740

Query  609  NFIWAEWNNYFHYMIEHVDEYMRQWGTVGGLSVEGQEGGNKVHRMAEKGHARANLIDGNK  668
                  + +   Y+  H++  +     +   S E  E GNKV R   K  ++ N  +G +
Sbjct  741  PAHLGGYISIPFYVHTHIEHTLNSL-PIAPFSTESLEAGNKVLRHVLKLLSQNNR-EGIR  798

Query  669  DILVWMWYHSSERLQRMAVRARKIYTCSTCGGRGHISSNHSCPIR  713
              L  +W  S   L+ +     +   C  C   GH  +  +C +R
Sbjct  799  GALHILWLRSHPLLRELMEVETQEQECRMCHRTGH--NQRTCHLR  841
```

Figure 3 Alignment of RroRAG1L and AanRAG1L showed significant similarity.

Summary of newly identified representative Transib loci

Ascidians

Salpa thompsoni

8. SthRAG1L_2902

```
>MKHR01082902.1 Salpa thompsoni contig52158, whole genome shotgun sequence
   1 CTGAGCAATA ACTGAAAATC TCCTGGCTCA TGATTCGCAT CGCTTTGAAA
  51 CTGTCGGCAT ATATGTCATC GTCACTGAGA AAAGAATATT GCGGTGTATT
 101 TACTCTTGTC GTTTGCAGGA AACAACCGAA AGAATGACCG GATCAGTTGT
 151 AGCCCATCTT CTCGATGATG TCATGCAAGT TTTCCATCTG GGACGTTTTC
 201 CGTGCGTACA ATTTTCGAAG TTTTCTCACA ATACTATTGT TGACTTCCGC
 251 TCCAGATTCG TCCAGAGTTC CAAGACTTTC ATTATTTTCT ATCAACTCCC
 301 ACGTATGTGA AAGTACTTTA TGTACTGTGG GAGTTAAAGA TACCCGGGTG
 351 AAAATTTCTA AAAGTAGCAA ATTCGTTTCG ATACCCAAAT CTTTGAATGA
 401 TTCAACGTCA ATTTTGACAT ACCTCAAAGA CACAACTGTT AGTATTACCG
 451 CCAATTTCGC TGCTTAAGCC TTTACACCCC TTAGATCTAT TGGTGATAAT
 501 CCTTCTCCGC CAATGATGAA GTGACATTTT TCTCTTGACA GAAGCAGATC
 551 ACGAGCAACA TCACAGCCGT TGTTGCAGTT CCACCCGATA TCTATTTAAC
 601 ACCAAATTCC TTGATCATTT TTTCTTGCAA TCTCTTAGTC ACGTGCTTTC
 651 GATCTTTTGC AGCGTCACTA ATTTTATGGA CCTTCCAATC ATAATGTTGT
 701 GCTATGACGT GGACCAGCAA CTTCATGAAA TGGTCAAAGT AAAGTAAAAT
 751 GAAGTGCTGT ACCTGAACAG ACAAAATAGA TTGGCCAAGA AGAGGTTTAT
 801 GTGTCACACC AGTCCGAATT TCATAATCGC CAGGTTTATG TCTCAAGCTT
 851 CCATCTTCGT ATTACCGTTC TTCTAAACTC TTTTTGATGT TGTAAGTATT
 901 CTATTAATTA TATTTCCATG TTTGATGAAA TCGGGATCAC TGCACGTTGA
 951 TTTCGAGAAC AAAAACACAT CACAGAACGT GTCTCGAATG CCCAACATAA
1001 TAGCGGATGC TTTTCTATCG CACATTTAAT AGATTAATAT TAG
```

Query=PflRAG1LB
Subject=MKHR01082902.1

MKHR01082902.1 Salpa thompsoni contig52158, whole genome shotgun sequence

Sequence ID: **Query_555295** Length: **1043** Number of Matches: **1**

Range 1: 164 to 460 Graphics ▼ Next Match ▲ Previous Match

Score	Expect	Method	Identities	Positives	Gaps	Frame
67.4 bits(163)	3e-16	Compositional matrix adjust.	40/106(38%)	56/106(52%)	7/106(6%)	-2

```
Query  829  AKFRFLKKVYCAKLPKVDYKDDIESVKTVGIEMGMLLIDKFGYARWPNYLHKVIEHTQEL  888
            AK    +  V    +  K+D    +ES  K +GIE   +LL++ F          +HKV+ HT EL
Sbjct  460  AKLAVILTVVSLRYVKID----VESFKDLGIETNLLLLEIFTRVSLTPTVHKVLSHTWEL  293

Query  889  IEKEDSPGTIGGISGEGNEAGNKLFRQFRKLHSRKGSVMGGLRDTI  934
            IE   +S GT     +     G E   N  +  R+ RKL++RK  S  M    L  D I
Sbjct  292  IENNESLGT---LDESGAEVNNSIVRKLRKLYARKTSQMENLHDII  164
```

Figure 4 A protein fragment predicted from the genome of *Salpa thompsoni* showed significant similarity to PflRAG1LB. The protein fragment was corresponding to ZnH and CTD domain of core region of RAG1 homologs.

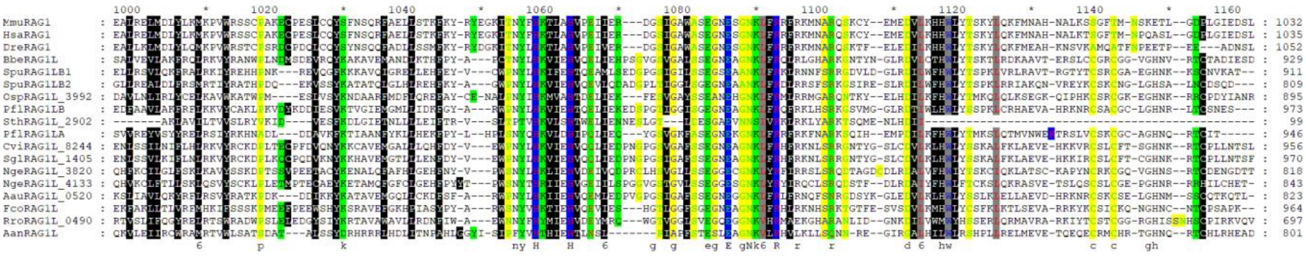

Figure 5 Alignment of SthRAG1L_2902 with other RAG1 homologs showed their conservation.

Ctenophores

Mnemiopsis leidyi

9. MleTransib_2823

>JH152823.1:422546-430223 Mnemiopsis leidyi unplaced genomic scaffold ML0180, whole genome shotgun sequence

```
   1 CTCCCTACGT CTATCTGCCC AGACCTCACC TTCTCCTGTC ACGTGATTTT
  51 TATTAATTTT AGTAACAAAA ATACAGATTT ATTTCTTTTA AGCTTCTTTT
 101 TTAACGGTTG AGTGTTTCAT TGATTTAGTA AAATCGCGAA AAATTAACAG
 151 AAACTTCAAC GTGTTTGTGA GAATCGACAT TGAGTCTTTT ACGAAAAGTT
 201 AGTGCCTAGG ATCAGATATG TATATATTCT TAGAGATTGA CATGAAGTTT
 251 CAAAATAGAT AAAAGATAAT AATCAGATAG TATTTGCTTT CAGTACAGTT
 301 TGTTTTTCTC CGCAGCTTGG TGGAATCGTC TTAAAAGCTC CTGGTAATGG
 351 GATGCTCCAG AGCTCCGTTA CCATAGGGAG GGAACATCTC CACTTTTACA
 401 GAAGAGAGCG GTACTCAATA GGTACCAATT GGCAGCAAAA TGTTTTCACG
 451 CCGACGGCGT ATGTACATTA TACATATTTA GAGATAGTAT GGTTAGAGGC
 501 CTTGGAAGTA ATAGAAAGGA CTCAGTTAAA AGGAATCAAC GAAAATCGGT
 551 AAAATGATCA TTGAAAAAAT GAGGATTATT ATCGCGAAGT ATTACTATCA
 601 AATTTTACAT GATTTTCCAT TTAATTGTAT AAGTGTTAGG GATCTCTTTC
 651 AATTGTTCCC CTAACTCCGT TAACCCGCTA ACAAAGAAAC TGAATTATAT
 701 CGCCCGTTAA TTGAGCTGAA ACACTGGCAA AATTTTATCG TTTTTATTGC
 751 CATAACCCTA GTTATCTAAC GGACGTTTAA ATAATAAAAT ACCGCATTAA
 801 CTTTCCAAAT ATAACAAAAA TATTGACGTC CAATTGGACT TCCATTGGTC
 851 GTTGCATGTC CCGATGATGG CATTTTGAAG GGACACAGGG ATAAAGAAGG
 901 CCGCAATACA TGAAAGAAAT AACAGGTTAA AAATTATAAC TACGAAAGAA
 951 TGTGCCAACG AGGATGATGC AGAAAACACA GGAAGGCTGG TAAGATCAAT
1001 TACAATTAGG GGCATAACTT TTGACCAAGT TTTAAAAAGT TGTTATATTG
1051 TTTGTTCAAT GGCCAATAAA CTGTTACAGT GGCGGAATCG TGACTTGGCT
1101 ATCAGGTTAA ATAACTGGAT AATTCAGGAA ATTATGATAG GAACTTCATC
1151 TAGTTTTACC ACCTCCACCT CAGCCACCTC CACCTCAGCC ACCTTCACCG
1201 CCATCCCCTT AGCCCAACAC AACCTCTTCG TCAAGTGCTG AAATAACCGC
1251 TGTCAACACA TTCATGAATC GTAGTAAACA AAAGGGCTAA TTTTATGAGG
1301 GCAAGACAAA TTTCCTTGCT TCTTTCGTAG TTTCCACTTT ACTTTGCTAC
1351 TTTTGCTCAA TTTTAAGTAT TAGAAGAAAA TCAAGGACAG AGAGGCTGTT
1401 GGTCGCTTTT GAATGAATTG TCATGTCAGT ATTTTGTTTT TCTTTTGTTC
1451 GTTCTCATAC AAGTAAAATG TAATGCTTAA GATGGAAGGC GAAACCACAA
1501 ATGCTTACTG CGAACAACAA AGGACGTTTT ACAGGACACA TTCCAGGTCA
1551 GATTTAAACG TCCTTTATTG TTCACAGTTA GCATAGAGTT GTTGTTGGCC
1601 ACTGTTTTCA TCGTATCATT CTGGAAATGA TTATCATAAA ATTTAATGTT
1651 TTTTATTTTT TATTCTTATA TCACTTTTGA CGAAGTTTGA AGAAGTTGTT
```

```
1701 ATGTTTTTTG CTCAAAAGCC AATACACTGT TACGGTGGCG GAATTGTAAG
1751 GGGAATCGAG ATTCGAGTAT AAAAAGTAAG TGGGACATCT TGGTTGGTAC
1801 TGTTTGTCTT AGATTTTGAT CAGTTATTTC CTTGATTTTT CATTTATAAT
1851 TGACAATTGA TAAAATGGTA ATTTCAGTGG ATCAGTGTAG AAGCTGAAAC
1901 GACGATAATC AAATTAAGAA CCAGTGTGAA GGATTTCTAA GTTTTGCAGT
1951 TACTTGATTT TCTGTCAGAC AGTCCTTTCT TGCTTAACTG TAATAAATCG
2001 AGCTCGAGAA GGGGGAATGA AAACTTATCT GTTCTATTTC AAAATGTTTT
2051 TGCTTCTACT CAGTGTCTGT ATGTAAACCA AGTTGCAGGT CAACCTCCCC
2101 CTCTTACTCT CTTATAAATC TTTTGTATAG TTCTAAAATC GATTTTTTCA
2151 GTTCCACTAT CTTTAGGCTA ACCGAGGGTA GCCGCTAACC AGTAATAATT
2201 GGGGAACGGG CAGCCCTCGG GGGGCCGGGG GAAAAAATAG ACGATATTGA
2251 TGGGATCTCA CCCCCTCCTG TTGCATGATT TTTAAAAGTT CCATGTCATG
                                   >>>predicted 3'TIR
2301 TCGTTCCCGG GGAAGGATAT CCACTACATC CCACAGTGGG GATCCATAAT
2351 GACATTAAGC TGAAAATTGA ATTTTTTTGA TTTCTTCACA TTTAATGTTT
2401 TTCATATCAG TTTATTGAGA TGAGTTTAAG GCAAAAAACC GTTTCAAAAA
2451 ATATCAAAAG CTGTTCTCGC AGTCGCGTCG CGAATTTTCT GAAATTTAAG
2501 TGGTGAAAAA AAATGCATTT TGGCAGTCAA ATTTTACTCT CTTTTTCATT
2551 TAAATCACTT CACATCATAA GAAAAACGTT TAAATGTCTT TCCTAACTTA
2601 AACAGACGAC AGAATTAGCT TTTTAGTACT TTTGGACCCA TAGAAATTCG
2651 ATTATCGACC GATTTTTGGT AAAAACTTAA AATTTTTGAT TTTTTGGCCT
2701 AATTTTTAGA AAAAAATGGG CAAAAATAGT TGTTGGCACG CCTTAGCACN
2751 NNNNNNNNNN NNNGTTTGAA AAATTGACAT ATTGGGGCCA TATTAATTTC
2801 GGGAAAATGG TGTGAACGGG AAAATGATGT GAAATTAAGA GTGAGCACAC
2851 CTAGCATGTC ATAACTCGGC TGCATTTTAG AATATATAGG ATTTAAAGAT
2901 AGATACTGTT GTGCTGTACA TGAGCTTGTT CATTGATTTA TACACAAACT
2951 TGAAACAGAT GATTGAAATC AAAGTGAAAG TTTAAGTGGG TAAATTACTT
3001 AAGAAAACAT TGAATAATAT CTTCTTCTCT AGTTAAAACT AGGACCCTTC
3051 TAGGACACTC CCTCTTTGCA TGCCCTTTTG TTTGGCACAC TTTGCACTTG
3101 GGCAATAAAT CCTGTCTTAT GGTGTTGAGT ACGGGATCAC TTCTTGTCCA
3151 TAGTCTGTGG ATGCAGTCTG TCAAATTAGT GAGCTGGTCC ACCTTTCTTG
3201 ACAGCGTGGT CCTGAATCGA CGTAGCAGTT TATTGCATGC TTCCAGTCCG
3251 CCCTCGCTTA AACCACCCAA CCCATGACCA ATGACCATCG TTTTTCTCTA
3301 TAAGTTCCCA ACCATGTCCC AGGATTTTGT GTACAGTAGG AGACATGTAA
3351 CATCTTGGTG GAGGATAATT CATCAAAATG AAGGTATACA AATCCAAACA
3401 GAAAGACTTA AATGTTGCTA GATCTGTTAC CTTCTGCTTT GAACTAATTA
3451 CCCTCATGAC AACAGAAAAG CGTTGTAGAA CTTGTGACAA AGGAGCTCTT
3501 CTGTGCTCAG GAACTCTGGA CAACAGCTCT TCCCGTACCA CTAAGTCATG
3551 GAGAAGTCGA CGGGCAACAT TTCCTGTTGT TGATGTACCT CCTCGGGAGT
3601 CAGGGTATGC CACTCTTATA CCAGTCACTT GTTTTAAGTG CTCTTGTATT
3651 TCAAGCTTGG CATTCTTGAT GAACTGGAGA TCGTAGTTGT TGGTACCTTC
3701 TCCCCAGTGA TTAACTCCTG CTACTTCATG GTACGTTAGT TTCAATACCC
3751 AATCTGTACC TCTTAACAAC ATGTGAAGTA CTTGCATACT CTCAACTTCT
3801 TTGCTTGTGG AAGGCATTCT CATGACCCCT TTACGCTGAT CATAGTCGCC
```

3851 AGGCTTGGTC TTGACATCTC CATCTGGAGT GGAAGGCAGC TTTTCACATA
3901 TGTCCACTGT AGATTGGAGC GTTCTCTCAA CTTTCATTGA GCACACTGAA
3951 GAAGCTTGAT GGGCTTCTTC TTTGGAATTG AAACAAAGGT CGCAATAAGC
4001 TCCACCAAGA CCAGTTATGC TATCAGCAGC TTTTCTGTCA AGTTCTGTCA
4051 GCCTAAACTG AACTTTTAAG TCAATTTCTT TCCCTCTTAC TGACAATCTA
4101 AAACCCTCTT GCTCTAATTT CTGAACTTCC TTGCTGAATT CAGCAAAACT
4151 TTTAAGAGAT TCAATAGTCT CTTTGCCCAG TTGCAAAGCA ACCGGGCGTT
4201 GTGTGTCTGC AGAACTAGGA TTCTTCTCGG TCCAAAGCAC ATCCTCTCCA
4251 GATTTCAATG CAAGTGGTGT TACCATAGTC ATGATAATGT TATTTGTGGC
4301 AGCACTCCCT TTTTGGTGAT GAATGTGATG ACCTCCACTT CCATCAAAAC
4351 CACCTTTAAA GACAAAGTTA AGATTCTCTT TGGTTAGGAG TTCATCAGCT
4401 TTGTCTCCCA GGGTTTCTAA AACCCTCTCA AAACACAACT GAACAACAGG
4451 TCCAAGTTCA AACATGACAC CATCGAAAGG AGGCGGAAGA GACTTGACTT
4501 TTGGTGAGAT TTCCTTGTGG AATCCGCTGA CATCCTTGTA GTTAGGCAGG
4551 ATATCAAGAC CCTCACTTTT CAGTAGTTTT CCGATTTTTA TATACTGGGA
4601 CTTGCCAATT TCAGCCTCAG CAATCAACAA GGAAGCCCCC GGTATTGATA
4651 ACTTTTGATT TGATATCGAC TCCTGAGAAA AAAGCTTCCT CCCTAATTCT
4701 GCCATATCTC GTTGGTCGTG GTAACTGGAA CGAGAGAGGA GCAGACCTGA
4751 AACAAAACAT GAGAGTACAG GAAAAGAAAA GTGATACACG CTGAATGACA
4801 AAGAACTGAT TAGTTAATTC TGAAACTGCA CTGTCAGTGA AACAGTTGCA
4851 TAATCAAAGG CAGTATAATT CAATATAAGT TTTAATTACC TGCAAGCCGA
4901 GCAGAAGATA TCTCCTCACG TCTAGCTGTT TCACAAAAGA GAGCATACAG
4951 TTCATCTAGT CTTCTGTAGC TTTGGCGCTG GCATAACTCT GAAATGTGCT
5001 GTCTTTTTCG CTTTACTCCT ATTTTTGCGG GTGCTAAGGG TTGAGACTGA
5051 CCTTCCTCTT TTTCTTCTTC TTCTTCTTCG CAATCTCCCC GGCCCCTCTG
5101 CTTTTCTTCT ATATCTTCTT CTGTCTCCTC TTCTTCCTCA CTCAGTATTA
5151 CCAGAGGACA TTCAGCCATC GAAGAGGTGT ACAAAACAGC ATCCCAATCA
5201 ATGTCAGCTG ATGATTTGTT TCGCTTGGCT GCTCGAGATT TCTCGTAGTA
5251 GCGTTTCAAT CTTACAAAAA CACTTTTGTG AAGCGATAGG GGAAAAACAA
5301 ATCCCATTTT AGCGTTTACA TTTTTGATGA ATGTTGGTTG AGAAAATGAT
5351 TTTATCGGAA AATCCCTAGA GTTTAAGAAG ATATCGTATG CTTCTTGGGT
5401 TGTGAGTGAC ATAATGCTGA ATAGTGAAAG AGTGAATGAG TGAGTGGATG
5451 AGTGACATTT TGAATTGCAA AATATTTGTA CACAATTTGT ATTGATGATA
5501 TTTATATTAT TGCATTTGTG ATACTACCAT TAAATTAAAA ACCTAAACAG
5551 GTATCCTTAC CCTGATTTTA TCTGAATTAA GACTACAACT TTTACAGCAT
5601 TGCAATGAGC TCGCTGGTGA AAGCGATTGC AGGTCACTGA ATCATCAGAC
5651 ATTGTCTTCA TCTACCGCTC TGCTGATGGA TTGGCCACTT GAGTCTTTTG
5701 CAGGTGGCCA ACCCTGTTCA TGTTAATGTT TAGTGGAAGA TATAAGTGTT
5751 GTAGGTATAA TTAAAGATCA ACAATTTTTC CTTGGGTTTT AAATTGGTAA
5801 TTTTTTTAAT TTGACCTCGA AAATCATAAA ATAAATCTGG TCTAAGCGTT
5851 CAATGATCAT CCATGCATTA TTTGGCTCCT GTTCTGTTAA ATGAATTTAA
5901 ATATGTTCTT GCATAACAGA GTGAATTAGA ATCATAACCT GTAAAAATTT
5951 TGGAATATAT GATTGATGGC GATGCGCATC AAATTTTTGA AAATTTGAGA
6001 TTTCTGAAAA ATTGAAAATT GAAAATATCT CATTATGCTT CTCTCTAATC

```
6051 GTCTTAAAAG TAGTTTTAAA CGTTTTTTTG GGCCAAAAAA CGTCTAGAAA
6101 AGTTGATTGT GAATATCAAT TCGAAAAAAA ACTTAATGTC ATAATCGCGC
6151 CTGCGATATC CCCACTGTGA GACCTTGCCT CTACCTTGCC CCAAGCTTGA
      <<<predicted 5’TIR
6201 CTCGGCCTTT TTTTGTCCAA CATTTAAATT TATTAGGCAA GAAAAAGGAG
6251 AAAACTGACC TCATTCACGT CATATAAATC GACTTGATCA TTATAATAAT
6301 AAACTAAAAT TTAAGCACTA CTTTAAAAGT GACACAGTAA AAAATTACAC
6351 TCGGACANNN NNNNNNNNNN NNNNNNNNNN NNNNNNNNNN NNNNNNNNNN
6401 NNNNNNNNNN NNNNNNNNNN NNNNNNNNNN NNNNNNNNNN NNNNNNNATT
6451 ATTTGGCTCC TGTTCTGTTA AATGAATTTA AATATGTTCT TGCATAACAG
6501 AGTGAATTAG AATCATAACC TGTAAAAATT TTGGAATATA TGATTGATGG
6551 CGATGCGCAT CAAATTTTTG AAAATTTGAG ATTTCTGAAA AATTGAAAAT
6601 TGAAAATATC TCATTATGCT TCTCTCTAAT CGTCTTAAAA GTAGTTTTAA
6651 ACGTTTTTTT GGGCCAAAAA ACGTCTAGAA AAGTTGATTG TGAATATCAA
6701 TTCGAAAAAA AACTTAATGT CATAATCGCG CCTGCGATAT CCCCACTGTG
6751 CATCCGTCTG TGTAATTAAG TAATTAGTCT GAGTTTGAAA CTTCATTAAA
6801 TGATTAAATG ATTTAATAGG TACGTTATAA ATTTCCTAAC GTTTAAGTTT
6851 TTGTAAATGA AATGTTACCT AGGTTCGATG GTAAGAAGTA CCAAGTTTAT
6901 AATGCTCATT TCTCCAAAAG ATGAAATAAA ATGAAATTTA GTATTTTTTA
6951 AAGATAAGAA GTTTTATTAG AAAGCAATAT GTGTAGCTAG TAATACATAC
7001 CAGACATATT TTCTAGATTG GCCGGATAAA ATTCTTTAAA TTCATGTCAT
7051 AATTTACACA TGAGAGTAGG AGACGTTAAC AACGAGATAA CATCATGACA
7101 GAGAACCAGG AATTTTATCC AAATTTTTTA GATAGAAGAA AGGAGTTTAG
7151 AAATACCTCT GAATCTATCC GTCCCCCACC CAGACATGTA TGTGAAAAAT
7201 GTTCATATTA ATGAACATTC CATATTAATG AAACGTCATA ATTGGAATCA
7251 TTGTACTGTA AATCAAATTG TGCAACAGAA AAAAAATCAA TAATTATAGT
7301 TATAGACAAC GGGTTAGGAA ATACTGACAA ACACACCAAC ACAATTGAAA
7351 ACACACAAAA ATACCGTGAT ACTGAGAAAT ACAAGTCTTA TCTTTCTCCA
7401 CTCGTAGTAG CGTGTTCACA TGACTTGTTA ACGGTCTGCC TAGCCCTTGT
7451 ATAAGTTAGC TGCATTCCTG AAAGATAGGG TTTTTGTTGG CAGTAAATCA
7501 GTAGGCGAGA GTTTACATGA CTGATAAATT ATAGTACAGC CCTGATTATC
7551 GTCAGACAAA AATACTATTT AGTCAGAAAC AATCAAACTC ACTTCTCTTC
7601 CTCTTTAAAT CTGTCCGATA TCGCAGCTGA AAGGACCAAT AGTGGTAGGT
7651 ATAAAACGAT AGCTGAGTGT CAAGAGAA
```

The transcript and protein sequences of *MleTransib_2823* were predicted by AUGUSTUS.

```
JH152823.1:422546-430223	AUGUSTUS	gene	2296	5661	.	-	.	ID=g2
JH152823.1:422546-430223	AUGUSTUS	transcript	2995	5652	0.12	-	.	ID=g2.t1;Parent=g2
JH152823.1:422546-430223	AUGUSTUS	stop_codon	2995	2997	.	-	0	Parent=g2.t1
JH152823.1:422546-430223	AUGUSTUS	intron	3212	3278	0.52	-	.	Parent=g2.t1
JH152823.1:422546-430223	AUGUSTUS	intron	4747	4889	1	-	.	Parent=g2.t1
JH152823.1:422546-430223	AUGUSTUS	intron	5417	5560	0.73	-	.	Parent=g2.t1
JH152823.1:422546-430223	AUGUSTUS	CDS	2998	3211	0.23	-	1	ID=g2.t1.cds;Parent=g2.t1
```

JH152823.1:422546-430223 AUGUSTUS CDS 3279 4746 0.54 - 2 ID=g2.t1.cds;Parent=g2.t1

JH152823.1:422546-430223 AUGUSTUS CDS 4890 5416 0.75 - 1 ID=g2.t1.cds;Parent=g2.t1

JH152823.1:422546-430223 AUGUSTUS CDS 5561 5652 0.73 - 0 ID=g2.t1.cds;Parent=g2.t1

JH152823.1:422546-430223 AUGUSTUS start_codon 5650 5652 . - 0 Parent=g2.t1

coding sequence = [atgtctgatgattcagtgacctgcaatcgctttcaccagcgagctcattgcaatgctgtaaaagttgtagtcttaattc
agataaaatcaggcattatgtcactcacaacccaagaagcatacgatatcttcttaaactctagggattttccgataaaatcattttctcaaccaaca
ttcatcaaaaatgtaaacgctaaaatgggatttgtttttcccctatcgcttcacaaaagtgtttttgtaagattgaaacgctactacgagaaatctcg
agcagccaagcgaaacaaatcatcagctgacattgattgggatgctgttttgtacacctcttcgatggctgaatgtcctctggtaatactgagtgagg
aagaagaggagacagaagaagatatagaagaaaagcagaggggccggggagattgcgaagaagaagaagaagaaaaagaggaaggtcagtctcaaccc
ttagcacccgcaaaaataggagtaaagcgaaaaagacagcacatttcagagttatgccagcgccaaagctacagaagactagatgaactgtatgctct
cttttgtgaaacagctagacgtgaggagatatcttctgctcggcttgcaggtctgctcctctctcgttccagttaccacgaccaacgagatatggcag
aattagggaggaagctttttttctcaggagtcgatatcaaatcaaaagttatcaataccgggggcttccttgttgattgctgaggctgaaattggcaag
tcccagtatataaaaatcggaaaactactgaaaagtgagggtcttgatatcctgcctaactacaaggatgtcagcggattccacaaggaaatctcacc
aaaagtcaagtctcttccgcctcctttcgatggtgtcatgtttgaacttggacctgttgttcagttgtgttttgagagggttttagaaaccctgggag
acaaagctgatgaactcctaaccaaagagaatcttaactttgtctttaaaggtggttttgatggaagtggaggtcatcacattcatcaccaaaaaggg
agtgctgccacaaataacattatcatgactatggtaacaccacttgcattgaaatctggagaggatgtgctttggaccgagaagaatcctagttctgc
agacacacaacgcccggttgctttgcaactgggcaaagagactattgaatctcttaaaagttttgctgaattcagcaaggaagttcagaaattagagc
aagagggttttagattgtcagtaagagggaaagaaattgacttaaaagttcagtttaggctgacagaacttgacagaaaagctgctgatagcataact
ggtcttggtggagcttattgcgacctttgtttcaattccaaagaagaagcccatcaagcttcttcagtgtgctcaatgaaagttgagagaacgctcca
atctacagtggacatatgtgaaaagctgccttccactccagatggagatgtcaagaccaagcctggcgactatgatcagcgtaaaggggtcatgagaa
tgccttccacaagcaaagaagttgagagtatgcaagtacttcacatgttgttaagaggtacagattgggtattgaaactaacgtaccatgaagtagca
ggagttaatcactggggagaaggtaccaacaactacgatctccagttcatcaagaatgccaagcttgaaatacaagagcacttaaaacaagtgactgg
tataagagtggcataccctgactcccgaggaggtacatcaacaacaggaaatgttgcccgtcgacttctccatgacttagtggtacgggaagagctgt
tgtccagagttcctgagcacagaagagctcctttgtcacaagttctacaacgcttttctgttgtcatgagggtaattagttcaaagcagaaggtaaca
gatctagcaacatttaagtctttctgtttggatttgtataccttcattttgatgaattatcctccaccaagatgttacatgtctcctactgtacacaa
aatcctgggacatggttgggaacttatagagaaaaacgatggtcattggaccacgctgtcaagaaaggtggaccagctcactaatttgacagactgca
tccacagactatggacaagaagtgatcccgtactcaacaccataagacaggatttattgcccaagtgcaaagtgtgccaaacaaaagggcatgcaaag
agggagtgtcctagaagggtcctagttttaactagagaagaagatattattcaatgttttcttaagtaa]

protein sequence = [MSDDSVTCNRFHQRAHCNAVKVVVLIQIKSGIMSLTTQEAYDIFLNSRDFPIKSFSQPTFIKNVNAKMGFVFPLSLHK
SVFVRLKRYYEKSRAAKRNKSSADIDWDAVLYTSSMAECPLVILSEEEEETEEDIEEKQRGRGDCEEEEEEKEEGQSQPLAPAKIGVKRKRQHISELC
QRQSYRRLDELYALFCETARREEISSARLAGLLLSRSSYHDQRDMAELGRKLFSQESISNQKLSIPGASLLIAEAEIGKSQYIKIGKLLKSEGLDILP
NYKDVSGFHKEISPKVKSLPPPFDGVMFELGPVVQLCFERVLETLGDKADELLTKENLNFVFKGGFDGSGGHHIHHQKGSAATNNIIMTMVTPLALKS
GEDVLWTEKNPSSADTQRPVALQLGKETIESLKSFAEFSKEVQKLEQEGFRLSVRGKEIDLKVQFRLTELDRKAADSITGLGGAYCDLCFNSKEEAHQ
ASSVCSMKVERTLQSTVDICEKLPSTPDGDVKTKPGDYDQRKGVMRMPSTSKEVESMQVLHMLLRGTDWVLKLTYHEVAGVNHWGEGTNNYDLQFIKN
AKLEIQEHLKQVTGIRVAYPDSRGGTSTTGNVARRLLHDLVVREELLSRVPEHRRAPLSQVLQRFSVVMRVISSKQKVTDLATFKSFCLDLYTFILMN
YPPPRCYMSPTVHKILGHGWELIEKNDGHWTTLSRKVDQLTNLTDCIHRLWTRSDPVLNTIRQDLLPKCKVCQTKGHAKRECPRRVLVLTREEDIIQC
FLK]

3’TIR analysis：

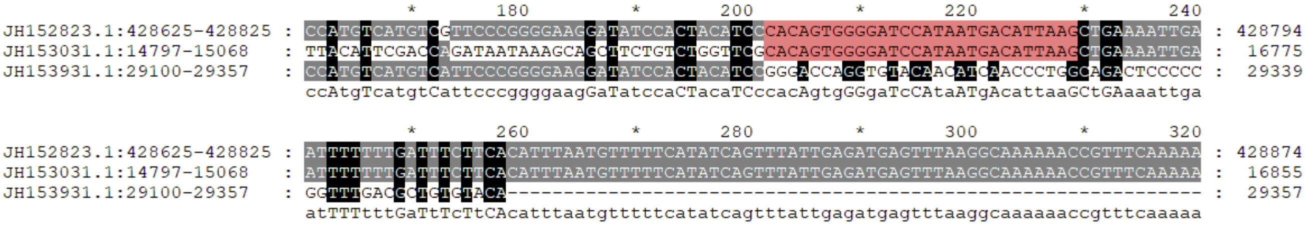

Figure 6 Alignment of 3’-flanking sequences of MleTransib_2823 showed its potential 3’TIR.

5’TIR analysis：

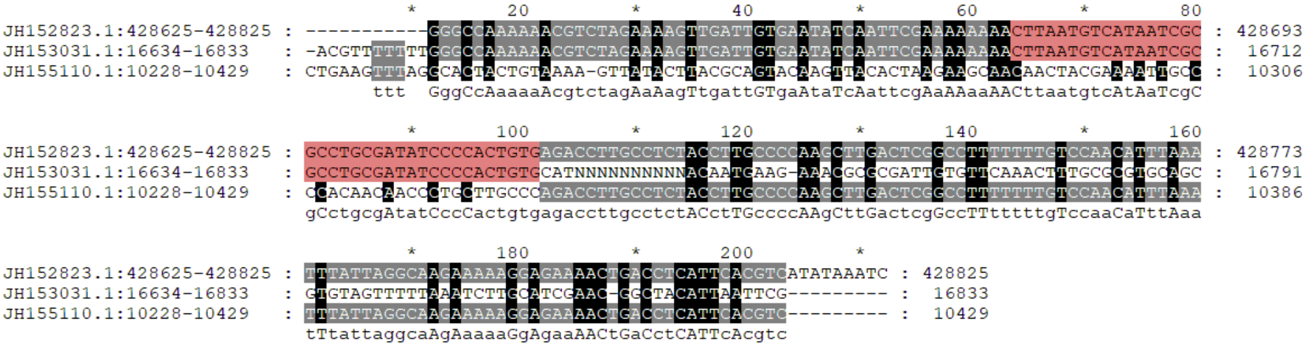

Figure 7 Alignment of 5’-flanking sequences of MleTransib_2823 showed its potential 5’TIR.

5’TIR+3’TIR analysis

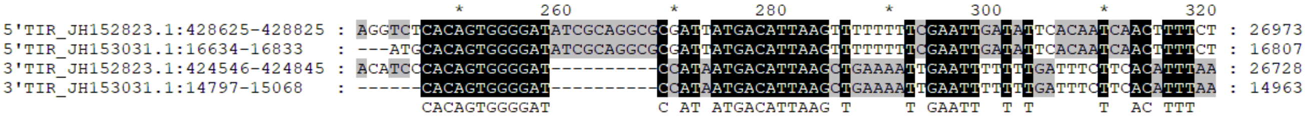

Figure 8 Alignment of 5’TIR and 3’TIR of MleTransib_2823 showed their conserved elements.

10. MleTransib_6236

>JH156236.1:146079-153902 Mnemiopsis leidyi unplaced genomic scaffold ML3593, whole genome shotgun sequence

```
  1 ACATTCTATT TTCATTCGAA TGAGAGTCGA ACTTGCTAAA GGTTGTCTAG
 51 CCCTTCTGCC GAGAAGCGGC AGCCTCTGGC GCGCACGAAG CGCGCGGCGT
101 CATGGCAGGA CAAAGGTCGG GCTCTGGTTA AAGCTAAACC TGCTAAGGCA
151 ACATAGCCCT GTGGTTCGAG CCGCGCGCAC CCACCCTAAT TCCGCTTGAC
201 GAGATTATAG CCGTCTACCG TTCTTTAGCT GCACGTGATC TGAGTCGGGT
251 ATCACCGCGA CAAGGATATA CGTGTTTAGC AAGGGTCGGA CAAAGTCAGA
301 CAATACTCAG CCACCCAGGC AATGAACAAT CGGTTTGTTT GAACATAAGA
351 CGGATATTTT CTCGCCTATA ATTTGACATG AACGTTGTCG CGGTGATACC
401 CGACTTACGC AGAACGGTAG ACGGCGTGGA TATAATTTGC CTTGCGTTGC
451 GGCAGCAGGT CAGGTCAACG GGCGATAGCG CGTGATGGCC GCGACAGTAG
501 GTTATCAACC TACAACAGGT CAGGCAACGT CTGACAAGAT ATAATCTTGT
```

```
 551 CAAATATGAA CGCTGATAAA CAATAAAGTT TCTGGTCTTC GATTGTCTGA
 601 CGGTATCCGT CCCTCATTCG ATTTTCGAAG CAAATAAATT GTTTCGTCCT
 651 TAATAATGCT TGTCCGACAT TCATTCGAAG CGAATAAAGA ATAAAATGAC
 701 GGCAGACGGG AGGGTGCGCG CTGTTATACA AAATAATGTG CGCGCGGTTG
 751 TCACGCCGCG CGTGGACAGA AGTAAGACTA AGAAATTTAT TTAGAATCGA
 801 GCGTTAAGAG CGACCTCCTC TCGCGGGTAG CGCGCACCTC GTGTTACAAT
 851 ACGAATTTTA TCGTTTAGCT GGGCGAGCTT TTGAAACCTT CTACATGCCT
 901 TCTGTTTTGA ACCCTGCCAA GGGGATGCCG AATGATTCCC CTGACGGATT
 951 GGAGAAGTTC TCTCAAGTAG TAGAAAAGAA GATCAGAAAC CTTGAGAAAC
1001 GAAAGGTCTG TATTATGTCG CTGACTGTTA CCGCAACAAA AATCGTTTCG
1051 CTGACGGTTA CTCAATTTCT TGTCGATTTC CAGCGCTTCA TTAGGTTGTT
1101 CGTGTAGTAA TTGTTTAGGA GCCACATTTT GCCAATTGTC CTTCTCTTTT
1151 GCCCATCATG CTAACACGAC TGCAGTCGTG AAGTTTACAC TGGCATACAA
1201 TCTTCGTATA GTTCTGATAT TTTTATAAGA GATTAGCTTA GGTGCATGTA
1251 ATATACAAGG GTTTGATACT AATTCTCATC GGTAAATAGC AATCACATTC
1301 TAAATAATAT ATTGTTTCAG ACCAAAATAC TCGTGTTAGT GGACCAGAAG
1351 AAAGGAGGAA AATCTCTGGA AGCGGATCAA GTGGTCAGTG TTACGTCTCG
1401 ATCTCGGAAG TTTCCTTATA AAAATATTCT TAAACAATAA ACATTTTTTT
1451 ACACATAGAG AATCCTGTCT GTGTAATCCC AGTTGAAGTA TTCGACTTAG
1501 AGCCTCCTCA TCTTTGCAGG CGTCAGTGAA CAGTCTACCG TTTGTTGAGG
1551 CTAACTTGGA GCAACTCAGG GAATTCCTTC CGCAATTCAA ATCCATTTCC
1601 AACGAAGTAA GTTTGTAAGG TCTTATTTAT ATACTGTAAA AACTGTGCGC
1651 ACAAATGATC AGAAAAGTAT TTCTTGCTGT CAGAGGCCAA TATCTCGGAA
1701 TTTTGAAGAT TTATTATTTA TTAAAGCTAT CAAAAAAGAA GATTTATTAA
1751 AGATTTATTA AAGCTATCAA GTGTCCGAGA AGCGATTCTA TCAATTCTTG
1801 ATATAAACTA GTATTTAGTA TACGAGTATT TAGTATTTGC CTTCAGAGGT
1851 TGCCTGCAAT CCCAGTCGTT CAATGCCTAA AGACACCGCC CTCTACACAG
1901 CTTCGAAATG CCAAGCCCAT TTTCATTACC CAGAACCTCA TCTTAGAATG
                                             >>>potential 3TIR
1951 TCATCCCCCA CCTACTTCCT ACGATGAGTG TAGTGTACAC AGTGGGGCAC
2001 CATTATGACA TAGGGTCAAA AATTGACTTC TGCAAATGTC TTAGATTTTG
2051 AATCCAAGTG ATCAGGAAAA TTCTGAAGAG CTTGGAACTT TTTGAATTAT
2101 GTGGATATCT TGAACATGAG AGAAGTTATG ATGATTCAAA GTTCGCCGTC
2151 TTCAATAATA TGTTGGATGA TCCATGGCTT CTATGCCATA CCTGGGAATA
2201 AGCCAAAGTT ACTGAATTAG CACAGCAGGC TTAAATATGA AGGTTTACTG
2251 AGGGTATCTT GTTACAATTG GATATCATGT AGTCTGAGGA TCACAATTTG
2301 ACTGGATTTA TTCAGTGTGG GCCATTTTCT GATTTTTGCT GGCCAGTTTT
2351 GTGACTTTCT GTCACAGTAC TATAATTTTG GGTCCAAGAT TTTTCATCTG
2401 TAGGCTCTTT ATGGCTCATA AGTTGAAATG ACAGCAAATG TCTCACATAA
2451 TATTGGCTTC TCAAGGCAGC TTATACCTTT TAGAAGGGAC CTAGAAAAAT
2501 TGTCTTGGGC TATCTTGCCA TTTTTGAGAA AAAAATTTTT TTTGAGGCGA
2551 GCCTGCGTTG ATTTTTCGCG CGCTTTTCGC GCGAGTCGCG AGACGCGACT
2601 CTAAAATTCA TATATTTTGC TGCCATAAAC ACTAGTTATT GCAAAACTGT
2651 TGATGTCTTA GGCCATTTTT TGACGAGGAA TCGAATTAAG ACATTTTCAA
```

2701 AATCAGTAAA CCTTGCCATA ATGACGTAAT TCTTAATTTT CATGATTTTT
2751 TGCAGTGAAA TTGACAATTT GACTAAAAAT GAGATGTGAA TATTTAATGA
2801 ATATGGTGAA ATCAACATGG TAAATTTTAA ATAATCAATT ATATCTCACA
2851 TTTAACCCAC CAGAATGTTT AAATAGTGCT CGACATAGTC TGCTACACTA
2901 TCAGAACTAA GTCCTAACTT CAGAGGACAT GACACAGTGT AATGCCCCTC
2951 CTTACCACAC CTGCTGCAAC AGATCTCAGG AGCCTGTCTT AGTGTCTTCG
3001 GATTTGTGGA TCACTTCTTA GCCACAATCT GGTGAAAGTG TCAGACATGT
3051 TTACAGCTTG GCTTCTTTTA CGGCTGAGAT ATTGTCGATA AAAGCGGAGG
3101 AATTTGTTGT TTGCTTCTAG ACCAGATTCA GTAAATGCAC CTAGACCGTG
3151 GTCATTGTTG GCTTGAATCA GCTCCCAGGA ATGAGACAGT AAGGCATGAA
3201 CTGTGGGGCT GATACTGATC CACTTTGTAA CTTCGTTATC AAACGAATCC
3251 AACAATATGT TGTAAGTGTC AATACAGAAA GCTTTAAACT CAGAAACATT
3301 GACCCTGTTT GTTGTGTTGT AAACAGATAT TATTATCCAA ACACGAGATA
3351 TAAGCTCTCT CATCTTCTCT TGATAACGTG TGTCGACACA GCTAACGAGC
3401 ACCTCTCTAT GGTGTGTAAG CATTCTCTTG CACACATCCC CTTTATTGGT
3451 GGTGCCTCCT TTGCCAGTCG GATCTGCAGC GTCCACTGTT ATTCCTGTTT
3501 TTGCTTTCAC GAGAGCTTTT ACCTCAAGTT TAGCAGTACA GTAGAACTGA
3551 TAGGATCTAC CCATATTAAG TGACGACTCA GTCCACTGAA GTACACCAGA
3601 TTTCAGATGG TAGATCAGAA GCAGGATGAA CTCAAAAGAA CGCATTAAAC
3651 AGTGCAGAGG GCTGACAGTA TTAAGGTCAA TGTCGACAAC TGGCTCTTGC
3701 GTCAAACCTT TCCTCTCATC ATAGTCACCA CGTTTTCTTT TGATGGTTCC
3751 ATCATCTTTC TTCAGTCGCT CAAAGTCAGC CTTGGTCTGT TGCACTGACC
3801 TGTTTATGGT GAAAAAAGAC TCAACATTAA CATCTTCTTC ACCCTCCTCT
3851 TTCCTTCCAC ATGCAGTTTC CTTCTTTACT GTACAGAGGA GACAATAGGC
3901 TCCTCCTAGA CCACTTAGCA AACCCCGCAT TTTAGAGTCA ATCATTGACA
3951 GGGTTGAATC CAAGGTGAGG TTAAATGTTT TACCACCAAC GTGTGCTACT
4001 GGCTGCATTG TACACCTCTC CCGTACAGTG TTGGCAATGT CTCTCAAGTT
4051 ACCAAGCTCT TCCTTGCCTA AGATAAGGAA CACAGGCCTT TGGGCAAAGG
4101 GAGAAGCTGG GGACATCTCC TCGAAGATTT TCCTCCCAGA TGCTACTTCC
4151 CGTATCTCCA AGACAGAGAA CATGAACAGT ATCATGTTGT GTGTGTCTTC
4201 ATTAGACATC TGCTGATAGA TAGCATGGCT GCCTGAGCCG TCAACACCGT
4251 CCTTAATAAC CACTACAAGG CTACTCTCCA AATCTGCAGT CCCTGAACTT
4301 ATCACTAGAT CAAGGATTCT CTCCACTGTG CTGACTAAAG CACTGCAGTA
4351 GGAGAATCTA ACGCCAGATA GTACAGTGTC TGTCAAGATG GGAGGGGTAA
4401 TAGACTTCTG GAACTTTCTC AGATTCTTCC AGGTTGGAAC CAGATTACCC
4451 AGGGCAGAGT GGTGTTTACT GAGAAATTTT CTCAGAGTCT CATACCCAAA
4501 TCTGCCTAAA GCAAGATTTT GTTGCAGATA GGACACCTGC TGATGAGTGA
4551 ACGACTTTAT GGGACTCCTG ACATCGTCAT CGACCAAACT GTGGGCTAAT
4601 CTGGAAACTT GTCGGTCTTT GTGGTAGTTA ACTTGTTGTA AAACAACCCC
4651 TAAAAGCTCG TTCACAGAAA CCTTGTTGAC CTCTGCCTTG ATCTCCAGGT
4701 ATTGGATAAT CTCAGCAGTT CTTCTGTACT GCTGTCTCTT GCTAACTTCC
4751 AACAGAGATT TTCTGTAATC CGTTTCATCA GAGAGATCCT CTTGCTCAAG
4801 CTCCTCCTCT GAATTTACCA ATTCAATCAG GACTGTATTG GACTGCTGCT
4851 TGTCATTCAT GTTGCGCAGA AAATTCTGGG CAGCATTGAT GAGGTCTGGT

4901 CTCCACTTGT CATCTATATG ATGCTCTCTG ATCAGTTCCT CTGCCAGGGC
4951 AGTTTTTCTG ACTACAGTGA ACTTTGATAC TTTGAGTTTT TGAATGATTG
5001 TGTGAAGTGA TCATGGGTCA ATATGTATCG TTCAGACATG ATCTAAGTGA
5051 AGTGATANNN NNNNNNNTTT AAGTAATGTA GACAGGAACG ATTCATCAAA
5101 CATGGCAAAA ATGATTTGAA AAAACTGCCT TATTCTTGTC GCACTCAATC
5151 GAGACAAATA TCATGACTGA AAACCTATTT TACTGGTGTA TTTAACCTAA
5201 AGATCATTAT ACTTCATGAG GCTTTTATAG GATTTTAATG ATAAACACTG
5251 TTTATAGATA CGATTTGATT AAAGAACAGT GAAAAATCGA TTATCCGAAA
5301 AAAATCCGAA AACCGAAACG AAAAAAACCG AAAAAAACCG AAAAAAACCC
5351 AAAACCGAAA AAAACCGAAG GCTACAGCAA ATTTCAAATT TGGGACTGGT
5401 TTTTTTCGTT TTTTTTTGGG TTTGCAACCC CCCCCCCCAA CTCTTCTTCA
5451 GTCAGCTCAG CCAAATGACC GGAAAACCGT GATTGAGGTT AGGTGAGGAG
5501 GGTCTCCGTT AAAAGATCTA GAATCTTCCA GGAGCCACTG TTTCTTAGAG
5551 ATTTAATTTA TGATATATTG TATCCCAATC TTTCATAAAA GAGAACTTCC
5601 CCCAACTCAA AACATTTTTT GACGAAAACT TTTCGTCACA GAACACATGT
5651 CTGATGACAT CTTCTTTAAC TCAGTTTCAG ATAGCACTCT ATTGAAAATG
5701 CCCAATTTGA GCAAATCTCC TCCGAACACA TAATTATTGC TATACTTGAC
5751 TTTTTTAGAG TTTCCTCAAC ACGTCTCAGC CACTCCACGT CATACATACA
5801 CGGAACCAAG TTTCCAGTGT ACAGTGTACT GGTTTTTGTC TGCCACTGTA
5851 CTGTAGGAGA CATTATCTCC CAATGTGATA TTGTCACAGT TGTGAAAGAC
5901 TGTCGAAAAA GAAGATCCGG AAAAGTGTTG CTAGTGCCTA GAGTAAGCCT
5951 GGTCTTGGAG TTGTCCATTA TCCCAGCTTG TAATCGTTAG TAGTCTTCCG
6001 GGTTTGGTCC AAATTTTGAA GCGGTGTTGT TGCTCGCTGC AGTTTGACGA
6051 CAGGAAATTG TGAAGAACAA TGTTAGAATA AAAACTTGTG ACAAACTTAA
6101 TATAGCGCGA CTGCAACAGA TGTGGCCGTA TTGGTATCGT AAAACTCGCT
6151 TGCTAAACTT TACTCACATA CGAAACTAAG ATTAAGGTCG CTCCAAACAC
6201 CGTCCTGTTG GTCAGGAAGG GGAGTACAGA TTACTACTTA TTGTTTGTTT
6251 ACTACTTATG ATCACGTGAT GGTTACTATC CCTGTCACGT GGGTTTGGAA
6301 AGTGTATGTT TTTCGAGACA ACTAAATCAT GTTCTTTGGA CGAACCTGTT
6351 GCTAAAGTTA AATCTGGCAA TTCACTCCAT TGAGTAGTAG ATCGATAAAT
6401 TCCTCGTGGA AAATTCCTTT TTCAGTTCAT TGTTGCAAAG TTATTGAGAA
6451 TTGGGCAGTA TTCGGAGCGT TAATACTAGA GAAGCTATGA AGAAAAGCTT
6501 AAATAACAAA CTTTAGGTAC AACCACATTC CTATCAAAAA TAACTGCGGA
6551 GCAAGTCTGC AGTTGAAAGT GAGACCAAAA CCCGTCCTAT TGTGTGGGTT
6601 ATATTTAGGA AATTTTCTCT TTCGGACTCT GTTTTCGGAC AAGCTTTAGT
6651 GGAGTAAGGA GTTTCTGTGC TGTGGAGTAC AACAGTTCTG CTCTGTCGAT
6701 CATTTAGTAT TTAGTTTGGA TCGTAATAAT TCGTACATGT TTATACACGA
6751 ACCCGGCTTG ATCAAAACAC ATTTTCTAAC CTGTCTCTTC TCACGATTAC
6801 TAACGTCCGG AAATATTTTA TATTGTTAGA GTGAGCTTTC TAAAGACCAC
6851 TTTCTTAAGA CCACTCAGAG GTGTGTTAGT GGTTAAAAAA GTGTTCGCGT
6901 AAAATAAACA TATTAATAGT TTAGGTGTGC AATACTAGGG TTTACGACTT
6951 GAGTTCACGA AATAAATTTC CGCTATGATT TAGTTAGTAC CGAGACTTGT
7001 TTCTTAGTTT GTTCTGGGAA CATCGGTACA TTGGAAGTTA AATCCGTGTC
7051 GTCACATCAT TATTCATCCG TGTGTTGTCA GCAGTGTGTT GTCATCAGTG

7101 TGTTTGCTGC AGTAATATAA TGCTTATTTA AACTATCCTG TTGATCAGCT
7151 ACACATATTT AATCTAACTT GTTTAATCTA ACTTGTCAGT ATTAATAATA
7201 GGGATAAACA AATTGAAGGT GACCTAAATA AGTTTTTTAG CCTGAGAAAA
7251 AACTATGGCA AATTATGGCT ATCCCCCTAA TTAAAAAGTA AAACTTTTTT
7301 ATGTTAAATT GATTATGTAT TCTTTACTGA ATTACTCTTC TACAATTTAT
7351 TTTGTCTGAA ACTTCTTTCA ATCTGTGGAC ATCCACGTGT AATCTGACAG
7401 TCAAAAAACA CTGCCATTAC ACGTTATCAA ATTTATGACC TGTTATCTAC
7451 TAGTTTGTAT ATATATATAT ATAGTCACTG TATATCACAC AATAAGTAAA
7501 GTATTACTCT TTAAACTAGA GTTGTACTAT AACCAATTGT ACTGCACTGT
7551 AGTGATCATT GTCATTACTG CACTTCAATA TAAAACATAG TCGAGGATGC
7601 AGTCAAACTT TTCCACGGAA CGCCACAGAG CGCTAAGTAT CAAGTAATCT
7651 CCTGTGACCA AGTACCTCTT TTATCGTAAA CAAGTCCTCA TTCTTCACTC
7701 TTCAGTTTAA CATAATTTCT CAGCAATATT TCAACTCAAC AATACTTCAT
7751 TGTTAACTGT CGTATTCTAT TTATCCCTGT TTTCATGGTT ATGTAGAATT
7801 TAGATATTCT TTATGTGAAA CGTT

The transcript and protein sequences of *MleTransib_6236* were predicted by AUGUSTUS.

JH156236.1:146079-153902 AUGUSTUS gene 2989 4860 . - . ID=g2

JH156236.1:146079-153902 AUGUSTUS transcript 2989 4860 1 - . ID=g2.t1;Parent=g2

JH156236.1:146079-153902 AUGUSTUS stop_codon 2989 2991 . - 0 Parent=g2.t1

JH156236.1:146079-153902 AUGUSTUS CDS 2989 4860 1 - 0 ID=g2.t1.cds;Parent=g2.t1

JH156236.1:146079-153902 AUGUSTUS start_codon 4858 4860 . - 0 Parent=g2.t1

coding sequence = [atgaatgacaagcagcagtccaatacagtcctgattgaattggtaaattcagaggaggagcttgagcaagaggatctct
ctgatgaaacggattacagaaaatctctgttggaagttagcaagagacagcagtacagaagaactgctgagattatccaatacctggagatcaaggca
gaggtcaacaaggtttctgtgaacgagcttttaggggttgttttacaacaagttaactaccacaaagaccgacaagtttccagattagcccacagttt
ggtcgatgacgatgtcaggagtcccataaagtcgttcactcatcagcaggtgtcctatctgcaacaaaatcttgctttaggcagatttgggtatgaga
ctctgagaaaatttctcagtaaacaccactctgccctgggtaatctggttccaacctggaagaatctgagaaagttccagaagtctattacccctccc
atcttgacagacactgtactatctggcgttagattctcctactgcagtgctttagtcagcacagtggagagaatccttgatctagtgataagttcagg
gactgcagatttggagagtagccttgtagtggttattaaggacggtgttgacggctcaggcagccatgctatctatcagcagatgtctaatgaagaca
cacacaacatgatactgttcatgttctctgtcttggagatacgggaagtagcatctgggaggaaaatcttcgaggagatgtccccagcttctcccttt
gcccaaaggcctgtgttccttatcttaggcaaggaagagcttggtaacttgagagacattgccaacactgtacgggagaggtgtacaatgcagccagt
agcacacgttggtggtaaaacatttaacctcaccttggattcaaccctgtcaatgattgactctaaaatgcggggtttgctaagtggtctaggaggag
cctattgtctcctctgtacagtaaagaaggaaactgcatgtggaaggaaagaggagggtgaagaagatgttaatgttgagtctttttttcaccataaac
aggtcagtgcaacagaccaaggctgactttgagcgactgaagaaagatgatggaaccatcaaaagaaaacgtggtgactatgatgagaggaaaggttt
gacgcaagagccagttgtcgacattgaccttaatactgtcagccctctgcactgtttaatgcgttcttttgagttcatcctgcttctgatctaccatc
tgaaatctggtgtacttcagtggactgagtcgtcacttaatatgggtagatcctatcagttctactgtactgctaaacttgaggtaaaagctctcgtg
aaagcaaaaacaggaataacagtggacgctgcagatccgactggcaaaggaggcaccaccaataaaggggatgtgtgcaagagaatgcttacacacca
tagagaggtgctcgttagctgtgtcgacacacgttatcaagagaagatgagagagcttatatctcgtgtttggataataatatctgtttacaacacaa
caaacagggtcaatgtttctgagtttaaagctttctgtattgacacttacaacatattgttggattcgtttgataacgaagttacaaagtggatcagt
atcagccccacagttcatgccttactgtctcattcctgggagctgattcaagccaacaatgaccacggtctaggtgcatttactgaatctggtctaga
agcaaacaacaaattcctccgcttttatcgacaatatctcagccgtaaaagaagccaagctgtaaacatgtctgacactttcaccagattgtggctaa
gaagtgatccacaaatccgaagacactaa]

protein sequence = [MNDKQQSNTVLIELVNSEEELEQEDLSDETDYRKSLLEVSKRQQYRRTAEIIQYLEIKAEVNKVSVNELLGVVLQQVN

YHKDRQVSRLAHSLVDDDVRSPIKSFTHQQVSYLQQNLALGRFGYETLRKFLSKHHSALGNLVPTWKNLRKFQKSITPPILTDTVLSGVRFSYCSALV

STVERILDLVISSGTADLESSLVVVIKDGVDGSGSHAIYQQMSNEDTHNMILFMFSVLEIREVASGRKIFEEMSPASPFAQRPVFLILGKEELGNLRD

IANTVRERCTMQPVAHVGGKTFNLTLDSTLSMIDSKMRGLLSGLGGAYCLLCTVKKETACGRKEEGEEDVNVESFFTINRSVQQTKADFERLKKDDGT

IKRKRGDYDERKGLTQEPVVDIDLNTVSPLHCLMRSFEFILLLIYHLKSGVLQWTESSLNMGRSYQFYCTAKLEVKALVKAKTGITVDAADPTGKGGT

TNKGDVCKRMLTHHREVLVSCVDTRYQEKMRELISRVWIIISVYNTTNRVNVSEFKAFCIDTYNILLDSFDNEVTKWISISPTVHALLSHSWELIQAN

NDHGLGAFTESGLEANNKFLRFYRQYLSRKRSQAVNMSDTFTRLWLRSDPQIRRH]

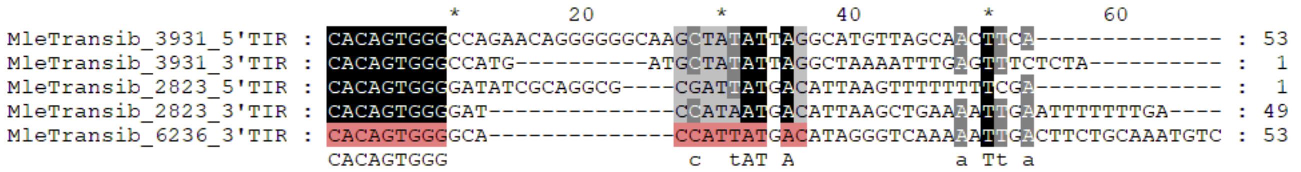

Figure 9 Alignment of 5'TIR and 3'TIR of MleTransib_3931, 2823 and 6236 showed their conservation.

11. MleTransib_3931

>JH153931.1:59145-67150 Mnemiopsis leidyi unplaced genomic scaffold ML1288, whole genome shotgun sequence

```
   1 ATTACAACTG TTGTGCCTGG GTACAGGTGC ACTAAGTAAA GTACAAGATG
  51 AAGAAAACGG AAAGGATTAC TAGTAGCTTA CCCGGGCTCT CCAATTTGTA
 101 AGGGGAGCCC TTAGGCTACT TTAATCGTCG AATGAACTAT AATTTACACA
 151 TTATATTAAC TTAGGGAAGG CAAGGGTGTT AACTTTTTCG TTATGCTAGC
 201 CTATGTTTTG TTTTTATTTT CCTTGGTTCC TAACATTTCC AATATTTTAA
 251 CTAGCCATGG TGTTGAAAAC AGTAAAAGGT GTTTATTTCG CCTTTGCCTG
 301 TTTTATATCT TCCTGGCTTG GATCCTGTTT CATCATGTTA CCCTCATACC
 351 TCCTTGGAAT CTTCGTACCT CATGTCTACA GATCCTATGT GCAGTTTATG
 401 ATGGGAACTT GGCTACTTTT TCCACCCTCA ATTTACGAGG TGACAGTGTT
 451 AAAAAATTTG ATATATTTTT AGACAGATCC ACTACAATAA CAATTTTATT
 501 AAACAATCTT CAACTTATTA ACATCATCTT GAGTTTGAGT TTAGAGTTCC
 551 AAGTTTAAAT CTTATTTAAT GATTATATTT AAATTCAGCT TGTATTTGGA
 601 ACAAAGATTA GAGTGAGTGG TGACAAGATG AGGCGAAATG AGAGTTGTAT
 651 GATAATAGCC AATCACCGAA CACGTCTCGA CTGGATGTTT TTGTGGTCTG
 701 TCACTGGACG AGCTAGATGG TCAGCTCTTA TGAAGATCAT TCTGCGGGGA
 751 GACCTAAAAC ATGTGCCTGG CTTTGGTGAG TCATCCAAGG TCTGTTAATG
 801 TTTAAATTTA AATTGTGGAA AGGCACCTTT TCCTCATATG TGGATTCGCA
 851 TTTACTTCAT TCACACACAT GGACTTAATG CTCAACTAAA TGCTATATAA
 901 ACAGTGTCGT AATGTTTGTG GCCTGGCCAA TAAGGTATCA ATTGGCAGTA
 951 AAAAGTCACC CCTATATGGC GCCCCTGTTT CCATTCATAG TACATTGGCC
1001 AAAAAATCTG AATAAATAGT GAACTTATTA AACAAAACGT GCAGTTTTAC
1051 TAGTAGTGCT GTTAAGCAAA ATTGTCTCAC ATTTTTATGA CAATTTGGTG
1101 CCCTCTTGTT GCATCATCCA CCCTCTGACA TAGCCGGGGT ATTGTTGTGC
1151 AACTGGCTAT AACTCGTACA TATTAGGATT GGAGAGCTTT AAAGTAAGGA
1201 AGAACTCTGA TAGCTCCTTC TCTAATTAGG TGATTTATTC GACAGGTTGG
```

1251 GCGATGCAGT TTGGTGGGTT TTACTTCTTA TCCAGGAAGT GGGCTTCTGA
1301 CGAGCAATAC TTAACAGATA AGATCAGGTT TGATGTGAAA ACTTTGGAAA
1351 TACTCTGTAT ATATGTACAT TGTATTCATT ACTGTCCAAA TTATGCACTT
1401 ATTTATTATC CAGTAACTTC AGAATGTTGT TTATGTGAAT AAGCTTCTGA
1451 GAATTTATTA ACTTATTATT GAGTAAGGTA TATTTAGGTA GGGCTCACAA
1501 ATCATTTACA TTATAGCGTT TTCTCGATGT TAATTAGTCC TACCTAAGTT
1551 TTCATAAGTC AATTGCTGTC ACATCGTACC AAAATTTGAG ACCGCTCCCC
1601 AAAATACCTT TCTCTATTAA ATTAGTTTCA CAATCTACAA ATTTTTTGCT
1651 TTGTACAAAT GAAAATCTAT TATATGTATA TATGTGAAGT GCACCAGTAA
1701 AACCATCACA ATTCTAATAT GTTATTATCA TTATTATTCT AATATGCATT
1751 TTCTTTCTTC AGGTATTTTA CAANNNNNNN NNNGAACTTG ACTTCAGCTA
1801 AAAAAGCATT TTGTCCAAAG TTGGTTTGTC CAAAGTTGGT TTAGTGATAC
1851 ATCATGGTTT AACAGTGATG GAATCGCTCA AAGAAAATTC AGTACAATTG
1901 GATGCAGTTA TAGCGCCCCT CAAAAGTCAG CTGACGCAAA TAAAAGTAAT
1951 GGTTTGGGCG GCAGTAAGCT CAAAAGGCGT TATTGGACCT TATTTTTTTC

>>>predicted 5’TIR

2001 ATAAAAATGT CCCTTCACAC AGTGGGCCAG AACAGGGGGG CAAGCTATAT
2051 TAGGCATGTT AGCAACTTCA GTTCTACTTC AAAGACTGTT TCTAAGGGTT
2101 TTTGGGCCTA AAATTACGTT TTAAAGTACT TTCAAGGCTC TAAAATGCTT
2151 ACTTTCGGAG AAATTTTAAA TTTCATTCAA ATTTGAGATC CTAGATTTGC
2201 GCGGCGATGC GCGTAAAATT TAAATTTTAT GATATAACTT AATTTCCCTA
2251 TTGTGCATTT ATATTTCATA ACAGTTGATG TTAAATATGC TCTATTAAAA
2301 TACTATTTCA TTCTAATATT CGTTGTATAC CATAGATGGA TTCACAAGGG
2351 AAAAATGACA CTTCTAACTA GCATGATGTC AAACACAAGT TATCATTTTT
2401 ATTCGAATAT ATGGGATCAG ATTGCACACA TTAGTTTCAA GAAATTTTTA
2451 GGGTTGACCA CCCTGTGTAT GGTGGTCAAC CCTTCAGTGG AAGTGCCTTA
2501 AGTCGGAGGC CAAAGGCATG CCGTTTGACC AATGATGACT TATGTAAGCC
2551 TAGGTTCAAC ACCGACTTCA AAACACACTG TAAAATAATT GTGTATAACC
2601 TTCAAACCAA TAAATCATGA TCATTAATTT TTCCCGGTTG AGTATCTTTG
2651 TAAATCATAA TCTTTATTAC ACAATCACTA AATTACAACA ATTTCATTTC
2701 ACTGCACTTA TCACTTTTCA TTTAGTCATG GTCATATACA AATTAATCAA
2751 GAAAGATGCC TACCAAATCT TCACTACATC ATGTTCACCA CATCAACAAC
2801 CATCTCAATC AAAATTCGCC TCATCGATTG ACAAGTTATT TGGATTGAAA
2851 TTTGATACAG CTCTTCATCA TACAAAATTT AGAACACTTC AAAAAATTTA
2901 CATCAAAGAA CGACGCCTTT CTAGCAGTCT ACGTAACAAG TTTGTAGAAC
2951 AACACTGGTC AGACACGTTT TACGACTGTG ACATGGCACA CTCTGTCTTG
3001 ATAGACGTTA ACCTTAGTGA CAATGAGGAA GAGGACAGCA AAGAGACCTC
3051 AAGCCCTGCT CCGAAGAGGT TTCGTCGACC AATCTTGTCT GTTGGAAAAC
3101 AGCAGCAGAA CAAGCGCCTC GATTCATTGT ACAATGATAT GCTGACGACT
3151 GCTGAAATTG AGGGAATCTC TCCTATTCAA ATTGCGGCCA TGTTGCTGTC
3201 TCGGTCATCA TACTCTAAAA ATAAGGAAAT AGCTGAGATT GCAGAGTCCA
3251 TAGTGCACGA TAAATTCTGT GAAAGTAAAG TAAACCAGAA ACTATCTGTA
3301 CAATCGGCTG CTTTGCTTAT GTCAACCTTG GAGATAGGGA AAATACCTTA
3351 CATCAAAATG AGGGTAATTT TTAAGTCTGA GCTTGGCTCT GCAATTGTTC

3401 CTCGTTATGA GCTCGTCTCA GATTTCAACA CGTCCATCAC ACCAACTTGC
3451 CAGGCTTTAT CTGAACAGTT CGCCGGAGTC AGGTATCCAC TTCTCCCGGC
3501 AATCACTATG ACATGCCAGA GACAGCTCAG CGTGCTGGAT TTAGAGGTAG
3551 CTGAGGTTGA GGGTCTAGAA GTTGTCTTCA AAACAGGTAT AGTAAAATAG
3601 AATAACTTTA ACTCAGCCTA AATTATCACA TATTTTCATT CATAATTTCC
3651 TTTTTCAAAT ATGTCACTCA CATGTACATC AAAACACTAC AACACCGCAG
3701 GATTTGATGG TAGTGGCTCT CACAACATCT TCCACCAAAA AGGATCTGCT
3751 GAGACAAACA ACATCACTAT GGGGATGATT TGTCCATTAA CAGTGAAATC
3801 TAAGGAGAGG ATTCTTTGGA AGCAGCCCCG ACCCCAATCT GCCAACACTC
3851 ATCGGCCAAT TGTCCTACAA CTGGGTAAAG AAACTGTTGA GTCTCTGAAA
3901 ATTTATGGGC CTATCACCCG GGAGATGGCA GACATCGAGG AGACCCCCAT
3951 TAACCTGATT GTTAAGGGGC ATGAAGTTAA GGTGTCTGTT AAAATCAGAA
4001 TAACAGCACT TGATCGAAAA GCTGCGGATG CAGTTACAGG ACTTGGGGGT
4051 GCATTTTGTG ACCTGTGTTA CATGTCTCCT GAAGAAGCTC ATGATGTTGC
4101 TGAATTGGAG GATGAGCTGA CAATGAGCAG GACATTAGAG GGAACAAAAG
4151 AACTTGTTGC AACTCTCGTT AATAGTTCAG GGGAAGTTCC TACTAAGCCA
4201 GGTGATTGGT ACACTCGTTT TGGGGTAATG AGAGCTCCCA CAGTTGAGAA
4251 AGAGGTCGAA TCAACACAAG GCCTCCATCT TCTCCTGCGT ACTACAGATT
4301 GGTTGCTAAA GCTGTGTTAT CATGAGATTG CGTGTGTCAC ACATTGGTCA
4351 GAGACAGTGA GCATGAGAGA CCTTCAATTC ATAAAGCAGG CAAAATCTCT
4401 TGTGCAAAGC CATTTGAAGG ACAAGACAGG TCTCAAAGTG GCTTTTCCTG
4451 ACTCAGCTGG TAAAGGAGGG ACCACCACAA GTGGTAATGT TTGTAGAAGG
4501 TTACTGTTTG ACAAAGAGAC CAGAGAAATC CTTCTCGAAC TAGTTCCTGA
4551 GAGAAACCGA GACAAACTGA GAGTTATTGC TGTGCGTTTA GCTGTTGCAT
4601 TAAGGGTGGT CAGTTCAAAG AACGAGTTGC AGGAGGAGAA GGTAGCTGAG
4651 TTTGAGGCTT TTAATAGGGA AACCTATAAA AAAATTCTGA CCAGTGCAGC
4701 TACCCTCCTC CCCAGGTGAA GATATCACCT TCTGTACACA AATTACTAGG
4751 TCATTCATGG GATCTCATTG CCCTCAATGA TAACTGTGGA CTAGGAACAG
4801 TGTCAGAAGG TGGTATTGAA GCCTGCAACA AGCTTCTGCG ACGGTACCGG
4851 ACCAGACTAA GCAGGAAGCG AAGCCAGCAC GATAACTTGT ATGATTGTGC
4901 AAAGCGCTTA TGGGTCAGCT CTGATCCTGT ACTTGAGAAC ATGAGGTTGA
4951 AGAATCTTCC TGTGTGTAAG AAATGCTCTG GTAGAGGACA CATAGGGAGA
5001 TACTGTCCTT CATCAATCAG CACAGTGATC CAGGAGGAGG ACGCGCTGGT
5051 ATCCAGCTTT TTTAAGTGAT ATTTTTTAGT GTGATCTTGA TTTTAAGATG
5101 AGCTTTTTCG TATTTTTTAT TGTATTTTAT CAGCACACAT TGCTTTATCC
5151 CCTTATATTC CATTTTTAAT TTTGATAGTT TAATGCAAGC ACTACACAAT
5201 AATATTTTGA TTTTTATTGC TTCAAAAATG GGAAATTAAG GTCAAAATTG
5251 AGCAGAAAAT TGCGCGTTTG GGCCTCAAGT CGAAATTTTT CAAAAATTTA
5301 TTCTGAAAAA ACCCCAACCT AGCCCAACCT GGTCTATACA GGGTCTTTTA
5351 GAGAATCTTC TCGCCTTTCA GATGATGCTA TGCATTAGTG GGCCAAATGT
5401 GGCCTTTTCA ACTATTTTTA CCTCTTAAAG CCAGAAATTT TAGCAAAAAC
5451 GCGCCCAAAA TCAATCGCGT CTCAAAAATT AGCTGAAATT GACAGATCAA
5501 AGCAGTAAAT TGCAAAATTT AGGGCTTCTA TAGATGATCC AGGTAAAGAA
5551 AACGTAATTC TGTGCATATT TAAGCATTTC AAGGCATACA AGAACGAAAA

5601 ATACCAATTT TCACTGCCAA AATAGGCAAA ATTTGGCAGT CAAATTTTGC
5651 CTCATTTCAT CAATGCGCCA TTCGCGAGTC AATCGCAATA TTCAATTTTG
5701 GTTTTTTGCA TCTTGTAGCT TTTGAATTTC TTTCAATATT TAATCAAAAT
5751 CTTTTTATTT TAGAGAAACT CAAATTTTAG CCTAATATAG CATCATGGCC
5801 CACTGTGGAC TTGCTAACAT AATATTATGG AAGCCGTATG TCGGCGGGCA
<<<predicted 3'TIR
5851 GGACGTAGAA TATAAAAATT TTCCGATAGC AGTTCGCATA TTTCACGACT
5901 TTCGACCCGA ATCCTACTAT ACTTTCACAG GGTTTAAGCT TGCACCTTGA
5951 CTAAGTTTGG TCCGGTTTGT AAGACCGACA TGAACACAAT GACAGTCAAG
6001 TTGTTAGTCG CTACTTTCAA GATTTCCGAG AGGGGGACAA ACTATATACA
6051 TCCAACATTT TGCCTATATC ATGTCATGTC AGGGTGATGC GACACAGTCA
6101 TATCGTTTCT TTATAATCAT TTTAAAAAGT AAGTTAACTT AAGCTCATCA
6151 AATCATTGAT CCGTAACTTT AAGCCACAAG CTGTCTGCAG ATACGGATAG
6201 AACGGCCTGG ACAATCAATA ACATTTCGAG AATTGGTCAA TGATGCAATT
6251 GTAGAGCTGT CATANNNNNN NNNNCTAATA AGATGATTTA TTCGACAGGT
6301 TGGGCAATGC AGTTTGGTGG GTTTTACTTC TTATCCAGGA AGTGGGCTTC
6351 TGACGAGCAA TACTTAACTG ATAAGATCAG GTTTGATGTG AAAACTTTAG
6401 AAATTCTCGG TATATACTTA GTTTGTCTCA AAATGATTGT CATCTGTAGT
6451 TTATCAGTAA AAGCAAAGGA TTTAGCCAAA AATGAAGACT TGTACGATAA
6501 TTTTTGGTCG ATTTTGAGTT AAAGCCGACC TTACGCTCTT ATCAGTGTAT
6551 AAAGAGATGG ACTCTTGGTT AAATTTTAAA GTGGTGCCTT TATCCACTGC
6601 TGAACGTTAT GCAAATGTTC AAAATTGTCA TTTCCGATCT TATGTTTATA
6651 CCGGCTTCAA ACTTGAAAAG TCCCCCACGC AACTGCATAG TGAACTTGTA
6701 CAAGTGTTTG GAGAACCATC CACGCCTAGT CTGAGGTCTG TGCAACGTTG
6751 GGTAGCCCCC ATTAGGGACG ATTCTTTTAC CTTCAGTAAG AGTGCGGCTA
6801 CAGGGCGGCC TAGGTCAGTT AGGTCACTCG TGTTGGTGAA TAAAGTCGAT
6851 TACCCGATTA CTAAGGACCC AAGACTATCG ATAAGGGATG TAGCATCATT
6901 AGTAAAGGTC GATAAAAGTG CTGTTCACCG GATAGTGACG AAAGATTTGG
6951 AGATGAAACT TGTGTGCTCA GTATGGGTTC CTGCGGCACT TCCTGAAAAA
7001 ACAAACAGGA CAGGATTCAG ATTGCATGCT GTAGGGGTAT ATTGCGTGCT
7051 GTCAGTGAAG ACAAGAATGC AGTTTACTGT GTTGAAGACG ATTTTTGTTC
7101 ACCAGATTGC AGGAGCACTG CAGGGAGATC GATTATGCAG ACGGNNNNNN
7151 NNNNNNNNNN NNNNNNNNNN NNNNNNNNNN NNNNNNNNNN NNNNNNNNNN
7201 NNNNNNNNNN NNNNNNNNNN NNNNNNNNNN NNNNNNNNNN NNNNNNNNNN
7251 NNNNNNNNNN NNNNNNNNNN NNNNNNNNNN NNNNNNNNNN NNNNNNNNNN
7301 NNNNNNNNNN NNNNNNNNNN NNNNNNNNNN NNNNNNNNNN NNNNNNNNNN
7351 NNNNNNNNNN NNNNNNNNNN NNNNNNNNNN NNNNNNNNNN NNNNNNNNNN
7401 NNNNNNNNNN NNNNNNNNNN NNNNNNNNNN NNNNNNNNNN NNNNNNNNNN
7451 NNNNNNNNNN NNNNNNNNNN NNNNNNNNNN NNNNNNNNNN NNNNNNNNNN
7501 NNNNNNNNNN NNNNNNNNNN NNNNNNNNNN NNNNNNNNNN NNNNNNNNNN
7551 NNNNNNNNNN NNNNNNNNNN NNNNNNNNNN NNNNNNNNNN NNNNNNNNNN
7601 NNNNNNNNNN NNNNNNNNNN NNNNNNNNNN NNNNNNNNNN NNNNNNNNNN
7651 NNNNNNNNNN NNNNNNNNNN NNNNNNNNNN NNAAAGGTCG ATAAAAGTGC
7701 TGTTCACCGG ATAGTGACGA AAGATTTGGA GATCGAAACT TGTGTGCTCA

7751 GTATGGGTTC CTGCGGCACT TCCTGAAAAA CAAACAGGAC AGGATTCAGA
7801 TTGCATGCTG TAGGGGTATA TTGCGTGCTG TCAGTGAAGA CAAGAATGCA
7851 GTTTACTGTG TTGAAGACGA TTTTTGTTCA CCAGATTGCA GGAGCACTGC
7901 AGGGAGATCG ATTATGCAGA CGGAGGCGAG TTGTATTTGG ACGTTCAGCG
7951 GTTTTTTCGG CGACTACCAG AAACACTATT GAAAAGAGAA CTTAGCAAGC
8001 TATTAC

The transcript and protein sequences of *MleTransib_3931* were predicted by AUGUSTUS.

```
JH153931.1:59145-67150    AUGUSTUS transcript    2728 5069 0.02 +    .    ID=g1.t7;Parent=g1
JH153931.1:59145-67150    AUGUSTUS start_codon   2728 2730 .    +    0    Parent=g1.t7
JH153931.1:59145-67150    AUGUSTUS intron    3275 3337 0.21 +    .    Parent=g1.t7
JH153931.1:59145-67150    AUGUSTUS intron    3587 3700 0.86 +    .    Parent=g1.t7
JH153931.1:59145-67150    AUGUSTUS intron    4642 4694 0.98 +    .    Parent=g1.t7
JH153931.1:59145-67150    AUGUSTUS CDS  2728 3274 0.21 +    0    ID=g1.t7.cds;Parent=g1.t7
JH153931.1:59145-67150    AUGUSTUS CDS  3338 3586 0.21 +    2    ID=g1.t7.cds;Parent=g1.t7
JH153931.1:59145-67150    AUGUSTUS CDS  3701 4641 0.56 +    2    ID=g1.t7.cds;Parent=g1.t7
JH153931.1:59145-67150    AUGUSTUS CDS  4695 5066 0.27 +    0    ID=g1.t7.cds;Parent=g1.t7
JH153931.1:59145-67150    AUGUSTUS stop_codon    5067 5069 .    +    0    Parent=g1.t7
coding sequence = [atggtcatatacaaattaatcaagaaagatgcctaccaaatcttcactacatcatgttcaccacatcaacaaccatctc
aatcaaaattcgcctcatcgattgacaagttatttggattgaaatttgatacagctcttcatcatacaaaatttagaacacttcaaaaaatttacatc
aaagaacgacgcctttctagcagtctacgtaacaagtttgtagaacaacactggtcagacacgttttacgactgtgacatggcacactctgtcttgat
agacgttaaccttagtgacaatgaggaagaggacagcaaagagacctcaagccctgctccgaagaggtttcgtcgaccaatcttgtctgttggaaaac
agcagcagaacaagcgcctcgattcattgtacaatgatatgctgacgactgctgaaattgagggaatctctcctattcaaattgcggccatgttgctg
tctcggtcatcatactctaaaaataaggaaatagctgagattgcagagtccatagtgcacgataaattctgtgaaaggaaaataccttacatcaaaat
gagggtaattttaagtctgagcttggctctgcaattgttcctcgttatgagctcgtctcagatttcaacacgtccatcacaccaacttgccaggctt
tatctgaacagttcgccggagtcaggtatccacttctcccggcaatcactatgacatgccagagacagctcagcgtgctggatttagaggtagctgag
gttgagggtctagaagttgtcttcaaaacaggatttgatggtagtggctctcacaacatcttccaccaaaaaggatctgctgagacaaacaacatcac
tatggggatgatttgtccattaacagtgaaatctaaggagaggattctttggaagcagccccgaccccaatctgccaacactcatcggccaattgtcc
tacaactgggtaaagaaactgttgagtctctgaaaatttatgggcctatcacccgggagatggcagacatcgaggagaccccattaacctgattgtt
aaggggcatgaagttaaggtgtctgttaaaatcagaataacagcacttgatcgaaaagctgcggatgcagttacaggacttgggggtgcattttgtga
cctgtgttacatgtctcctgaagaagctcatgatgttgctgaattggaggatgagctgacaatgagcaggacattagagggaacaaaagaacttgttg
caactctcgttaatagttcaggggaagttcctactaagccaggtgattggtacactcgttttggggtaatgagagctcccacagttgagaaagaggtc
gaatcaacacaaggcctccatcttctcctgcgtactacagattggttgctaaagctgtgttatcatgagattgcgtgtgtcacacattggtcagagac
agtgagcatgagagaccttcaattcataaagcaggcaaaatctcttgtgcaaagccatttgaaggacaagacaggtctcaaagtggcttttcctgact
cagctggtaaaggagggaccaccacaagtggtaatgtttgtagaaggttactgtttgacaaagagaccagagaaatccttctcgaactagttcctgag
agaaaccgagacaaactgagagttattgctgtgcgtttagctgttgcattaagggtggtcagttcaaagaacgagttgcaggaggagaagtgcagcta
ccctcctccccaggtgaagatatcaccttctgtacacaaattactaggtcattcatgggatctcattgccctcaatgataactgtggactaggaacag
tgtcagaaggtggtattgaagcctgcaacaagcttctgcgacggtaccggaccagactaagcaggaagcgaagccagcacgataacttgtatgattgt
gcaaagcgcttatgggtcagctctgatcctgtacttgagaacatgaggttgaagaatcttcctgtgtgtaagaaatgctctggtagaggacacatagg
gagatactgtccttcatcaatcagcacagtgatccaggaggaggacgcgctggtatccagctttttttaagtga]
protein sequence = [MVIYKLIKKDAYQIFTTSCSPHQQPSQSKFASSIDKLFGLKFDTALHHTKFRTLQKIYIKERRLSSSLRNKFVEQHWS
DTFYDCDMAHSVLIDVNLSDNEEEDSKETSSPAPKRFRRPILSVGKQQQNKRLDSLYNDMLTTAEIEGISPIQIAAMLLSRSSYSKNKEIAEIAESIV
HDKFCERKIPYIKMRVIFKSELGSAIVPRYELVSDFNTSITPTCQALSEQFAGVRYPLLPAITMTCQRQLSVLDLEVAEVEGLEVVFKTGFDGSGSHN
```

IFHQKGSAETNNITMGMICPLTVKSKERILWKQPRPQSANTHRPIVLQLGKETVESLKIYGPITREMADIEETPINLIVKGHEVKVSVKIRITALDRK
AADAVTGLGGAFCDLCYMSPEEAHDVAELEDELTMSRTLEGTKELVATLVNSSGEVPTKPGDWYTRFGVMRAPTVEKEVESTQGLHLLLRTTDWLLKL
CYHEIACVTHWSETVSMRDLQFIKQAKSLVQSHLKDKTGLKVAFPDSAGKGGTTTSGNVCRRLLFDKETREILLELVPERNRDKLRVIAVRLAVALRV
VSSKNELQEEKCSYPPPQVKISPSVHKLLGHSWDLIALNDNCGLGTVSEGGIEACNKLLRRYRTRLSRKRSQHDNLYDCAKRLWVSSDPVLENMRLKN
LPVCKKCSGRGHIGRYCPSSISTVIQEEDALVSSFFK]

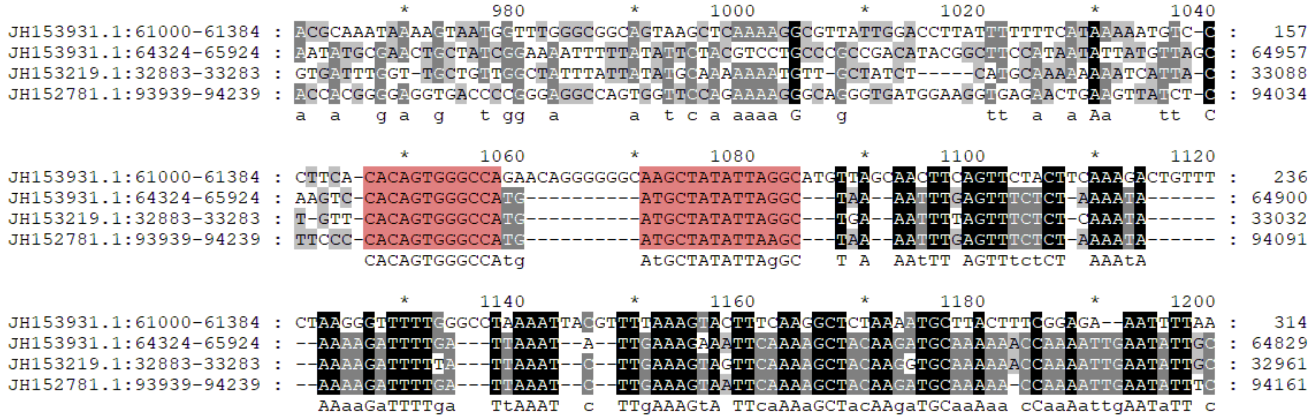

Figure 10 Alignment of 5'-and 3'-flanking sequences of MleTransib_3931 showed its potential TIRs.

Pleurobrachia bachei

12. PbaTransib_0868

>AVPN01000868.1:1-7805 Pleurobrachia bachei scaffold875.1, whole genome shotgun sequence

1 AAAATTTCAC AATGCAATTT TATTATGCCT ATTTCCGTTC TACCTGGAGT
51 TAACAATAAT GTTACAGATT CTTACACTTT TAGACCTCGC AGGCTCGGTC
101 ACGTGAGGTA GTTACACTTA GTGGCACTCG CAGGCTCGTG CACTAAAGTA
151 CAAGAATCTG CCTCGACCTA TGGTCTCGGA TCATTATTGT TGCACTTATA
201 ACACTCGACC TTCGGTCTCG TGTTATAAGT TGCCAGGTAG AACTTATAGG
251 CATAATAAAA TCTTACATTG TTGCATTTGA TAATAGTATT TTTCAACGAA
301 GAAAAGAAGA GCTGGTAACA GCGGTGATCA TGAATAAAGA CGATTACCCA
351 GGTCCCAGAT TCTATGATAA TTGTATCTTT AATTTAATAT TTATTTTAAA
401 TGACTTTACA ATTATATCAT GCAGTTCTCA GTCAATAATG AACCAAAGTA
451 TAAAGATAAA TAATTGTTGT GCTGTTGTGT TTAGTCACAT CCGTGAGAAA
501 GACACTGTTT CTGATTACCG GCGACACGGA CCGCTGAGAA AGAAGAATAT
551 CCCACGGCTG GTAACATGGG GTGGACGACC TCTTCTTCTA CGTTAACTTA
601 CATTGTGAGG GAGTGAGCAA GGTGTATTTC GACGGGCCGG TGAAGATGTC
651 TGATAATTAT ACTTTAAAAA TAATAAAATA TAGTAGACAC ATCAATTGTT
701 TAATAAATTA TGTAGCTTGG TCTTTAAAAC ACTAAACCCT TTTAAAATCC
751 TTTATATAGC TATGGCTGTT ACCGATGTTT CATAGTTTCT GCTACAGTCG
801 GACCCCGATT TACCGTTGGT AACGAAGTTA CCCCTCCCTT GCCGGCAAAC
851 GAAGGCCAAT ACAATCCGCT GAACCTGATT TTATAAACTC AACAGTTGTT
901 GAAATTAATG GTCATCATAT TCATTTACCA CAGAGACCCG GGAATCCACG
951 GGAATCCAAG GTACCCTCCC TCGTCCCAAT CTTCACCTGC TGGATCCACA
1001 ACTGGGAAAT CAAAGGTAAC CCCATTGCGA ACTGTGGGCA TATTTCCATT
1051 TGTAGTATAT CAGCTGTTAC AGCTGATAAA CTACAAATGG AAATATGCCC
1101 ATTCTCCCCG CACTAAAAAT AGAGGCCATC AGCACTGCGG CATGTATGCA
1151 ACACTGTTTC TGATTACCGG CGACAGGGAC CGCTGAGAAA GAAGAATATT
1201 CGTGCCGACC ATTATACCCC ACCTGCTGTT CTAGAGAGGA AGAGGCAATT
1251 GGATGATTGT GATCAGGGAG ACGGGAGTTA GCAAGGTGTA TTTCGGGCCG
1301 GTTAAGATGA CTGATAATTA TACTTTGAAA ATATATGTAT AAAGTGGACT
1351 TAATTCACCT TTCTAGTTCC CCCTGAATAC TCGGACTGTA CACAATCACA
1401 CTAAAAAGGA TGAAAACTGA AGGAAAAAAG AATACTTCAG AGGTAAGAAA
1451 ACCTGGTGTT CGAGTAATTA AAGAGTGGCG GAACAAATGA CATTTAAATG
1501 GAATTTAAGC GCTGCACACG GACACAAAGT TATCTTAATT TACCGAAATC
1551 TGGACCTAAT CTTGGACCTA AACATCAGAA GAGAAAACAA ACAAAAAACA
1601 TGTAGCTAAC TTTGAATTAT ATTTTATGTC TAACACCCCT TCCCCTAAAC
1651 AACAGGAGGC GTACAAAATG CCTCGGGAAA TGCCTCGAGG GTTTCGACAA
1701 TAGCTACCAA TTGGATAAGC GTTCTTGCCT CTGTGGGAAG TTAAAAAATA
1751 CTTTAACTGC TCGTTCGACG GATGCGATTT TTCCACCAAA GCGAAGGTTA
1801 GTGTGAGGGG CTATCAGAAC AAAAAACATC ATTGAGAGGG CTTGTACTCA

>>>predicted TSD+5’TIR

1851 TTCGAATAAT AAATATGGTG TGACTGTCCA CAGTGGGTAA GATTAGGGGG
1901 GGATGCTATA TTAGGGGAAA TTTCCTGTTC TAAAGAGAAA TTAATTTTCT
1951 AGGGGTTTAA AAGGCCAAGA AAACCGTTTA TGAACATATT GGAATGCATT
2001 GATATCTAAA TTTTTTAGAA AATTTGAGTT TTTATTTTTT TTTGTAAAAC
2051 ACAAGGTTTG TTTCTATTTC AAAATTCTAA AACAGTGAAA TTTATGTCCA
2101 AGATTTTTTG ATATTTTGAA TTATGTTGCT TGAATTGTGT GCCTAGTGTG
2151 ACTTGTAACA TGTGTAGTTT GACTTAATTT GTGATCACTG TGCTGGCAAG
2201 CGCAAAGGTG TACCTCTCCT CCCTCCCCCA ATTTTATACT TGCTTTACTA
2251 ACACGTAGTT CTCTGTAAAG TTATGTCAAA TGAGTATCAA CATCTCACTC
2301 TAACTGATCT CCTAAACATT GTCGTGGACG TGGGAGGATG GAAATGGATC
2351 AATGTACCAA CTCTAAGGGA TTTTGTTCAT GAGAAGACGA GTCGGAATTT
2401 TCTTTTAGAA GAACACTGTC GTGCCTATTG TTTTCTCCAG CGTGTTTATG
2451 AGCAGAGCAA GAAGAAAAAG TGCAATTTGT CAAACCTTGT TGCAACATTT
2501 GGTGACGAGG AAAAGATTTT CTGCACTGAA TCACTGGCAG TTGATGATCC
2551 ACATGCACAA GTATTAGTAA CTACAGAGGA GATAGAGATG GCTTGTGGAG
2601 ATACAGAGGA GGAGGATGCT GCAAGTGGGG ATACAGAAGA ACTGGCTAGC
2651 GTGACCGTGG ATACTACGGA GGATCAGGAT GCGAATCAAC CTGCAAAGAA
2701 GGCCAGAAAA CACCTACTGA ACATGACTAA ACAGAGCCAG TACCAGAGAA
2751 CGGAGGAGCT ATACAAGCAT CTCCTTTCTA CCGCCGAAGA GGAGGATGTT
2801 ACACCATCTT GGCTGGCACA GCTGCTGCTG GAGAGGTCAG CTTACCGTTC
2851 AGACAGAGGA GCTGCCAAGG GAAACAGGAC TACACACTCG ATGGAGGTGC
2901 CCCACGCGGC TTTTCTCCAG CAGGACCTGT TCTTGGGAAA ACAGCAGTAT
2951 GTCCGTATGA AGAAAGGCTT AGCACCCTAT GTTTCTTTAC CCAACTGGCC
3001 TGAAGTGAGG AAATTTCAAC TTTCTCTTAC CCCTAAACAC ACTCCTACTA
3051 CCAATCCAGG TACAAACAAG AATGCATCTT ATCTTTACAC TTCTCTTTGC
3101 ACATCTCCTT ACACTTCTCT ATATGTTTGC ATGCTCTTAC CAACTCTCTC
3151 TTACCCATAG TAACACTACA ACCACTGACA ACTACCAATC CAGGAATATA
3201 AACAAGAATG CATCTTTTCT TTAGGTTTGC ATGCTCATTT CTTGCTGGTT
3251 TAAAATGTAC TTATTTATTT TAGAAACGTG CTAAAGATGT ATGCTGGTTT
3301 TAACTGTGCT CAATATTTCA CGTGAGTTAT TATAACATCA AAAATAAACA
3351 TGCTGTCAAA ACAGAGGTTC CAAAAGCACA AACAGGGTTT CTCTCTCTCT
3401 ATCAACCATT ATCCCTCACT CAGGCACCAG TTTCAATTTT GAAGAGGCTC
3451 TGCTGATGAC TATTGAAGCA CTGGGAAAAT CACTTAACAT CACTTGGCCA
3501 CCCAACATAA ATGTATACAT GAAACATGGT GGGGATGGCT CGGGTTCCCA
3551 CGCCAGGTAA CATCAAGTGG ATAACTGTGA TACCCATAAT ATGTTTTTGT
3601 ATATGTTTTG CATACTCGGT GTGGAGGACA GGGAAACTGG TCAGCCACTG
3651 TATGTTAACT CCTCCTCCAA CTCCCCACAT ACCCACCGTC CTCTCTTCAT
3701 TGCCATGGGG AAGGAGGAAG TAATGGACGA TGACGTGGAG AAGGTCATGA
3751 AGGAGATTAA GGAGTTGTTA GAGGGACCAG TGAGGATGTG CGACACTAAT
3801 GTCTACATAG ACGGTAAGAT GTCTCTGGTC GATTCAAAGT TCCGGAAAAA
3851 CATGCTAGGT CTAGGGGGAG CATGGTGCAT GCTGTGCCAC GTCACATTGG
3901 AGACTGGGTG CGGTAGAGGT TTCCTGGAGC CGTATGATTT TTTCTTCATA
3951 ACTAGGTCTG GGAAGGAGGT TCAGGATACA TATGACAATC TGAAGGATGA

4001 TGAGGGGTGT GTCCCTACTA AAACAGGCGA TTATGCCGAG AGGGCTGGGA
4051 CTACCGAGGC ACCCCTACCC CAAGACTCAG AAACACTGGA CTCCCTCATA
4101 TTTGTATCTC CACTGCATGC CATCATGAGA GTGTTCAGCT GGGTTTTAAT
4151 CCTAATGTAT CATCTGATGG CGGGAATCCT CCGTTGGTCA GAGGGGAAGG
4201 TGGTTTTGGG GAGTTCCTAT CAGTTCTACG TTGCTGCCAA GAAGAAGGCG
4251 AACGAGGTGG TCAAGGCCAA GACGGGTATC CTTATGGATG CGCCTGATCC
4301 GACGGGTAAA GGGGGCAACA CTCTCAAGGG GGACATTTGC AAGCGACTTC
4351 TCGGGATAGA GGGAAATAGG GAGGTACTAG TTAGCCTGGT TCCGAGACAA
4401 TACCAGCAGT CGTTCCGTGC CCTGCTGGAG GGTCTGTGGG TCGTTATTCA
4451 TGTATACTCA TCCGACCCAG ACCCAGGCAG ATATGTCGAT GTGAATGTGT
4501 TCAAGATGGA GTGTTTGGAC TTGTACCACT TGATCCTTGA GAACTTCGGG
4551 AATCGACCTG ACCACCGCTG GGTGAACTTG TCACCGACTC TCCACTCAGT
4601 TCTAGCACAC TCATGGGAGC TCATAGAGGC CAACAACAAC CAGAGTCTGG
4651 GAGCCTTTTC CGAGAGCGGA CTTGAATGTA ACAACAAGTT TCTCCGGATA
4701 ATACGTGAGG TCAAGAGCAG AAAGATCAAT GAGACAGTGT GCCACCAGGA
4751 TACCTTGAAT AGGATGTGGG ATAAGTCCTG CCCCCTTGCT AACACATACC
4801 GTCCAATCGT GCACCCACGG CCAGGAGCTA GGAAACACCA GACGGACACC
4851 CTGAGTGAGC GAATTGCCGC TTGTTATATT GATTGATACA TTATTTTCTC
4901 CATTTTTTCA TGAATTCTTG TGTAATCGCC AAAAAACCTA AAATGCTAAC
4951 AGCATAATTA TGCAACTTTC GCGTCCAGTC GCGTCGCGAT CTCTACTTGT
5001 ACGGTGTGCA GGAGGGTCCA TATTAACATA TCCTATCAAT GAAAATAGCG
5051 AAATTGTGCG AAACAAATAC TATTTTATCT CCTTTTTCAC TAAAAAACGG
5101 GTCAATTTCA CCATATACAC GGAATGTTCC CCTGAGAAAG GCCAGTAAAA
5151 CATATTGTCA TGAGACTAAA GCTGAAAGAA TGAGAAATTT ATAGTTCTTA
5201 CTTTCCCGTA TTGAAATTTT AGTAAAAACA TGTACATAAT TCCGAAAAAA
5251 AGGTACAAAA ATGGACAATT TTCACTGTCA AAATGGATTT AGGTGTGAAT
5301 ATTTTCATAT GCGCATCATA TTTTTAGTCA TATCTTGGTC AGATCTCAAT
5351 ATTTTTTTAT GAGACTTCTT TTATTCGATA GAACTAACAA TTCTCTATAA
5401 CTAAACAAAT TTTCAGCGAA ATCTATTTTT TTCCCTAATA TAGCATCGTT
5451 ACCCACTGTG CTGTCAAGCC TGGTGATCTT CCAGCTGTTC ACAGAAATTC

<<<predicted 3’TIR+TSD

5501 CCCCATCAAT GCTCCCATCA ATGATTCTTC ATCCGATTCT GGTAGGAAGC
5551 ATCCATCTGT TACAATACGT CTGCACAATT TTAATCAGAA AGACGTGTTG
5601 CTAGACATGT TCAAAAAACG TGCTAAACGT GGCGGGGCTA AGCTAGTACA
5651 GTCCTTATGT CCTTACTACC AGGATCTGAG AAGAAGTATC AGCGACTATA
5701 ATAAACAAAA TATGCTTTGA CCATCTTTGG CAAAGGTTCT TTGCTTCTTG
5751 ACCCCATTAA TAATTTGTGA ATAATTTGAA TAACATTGAA TAAACTTCTA
5801 ATCTAATTCC ATGTTATTTT TACATTTTGA TGTTCCGCCT GATTGCAAGA
5851 ACTAGTGACG ATATATAATC CGGTGATACT ACCGCAAATG AGTATTGGAC
5901 AGACGGTCCC CATAGCGACC CCGATCCCTT CCCCTTCACA CTCCACACTA
5951 TCTGCCGATA CAAGGGAAGG ATATAAACCT GACCTTGCTT TCAAAACCGC
6001 TAAAGTCTGC CACCGACGAT TGTAAAGCTG TTTGTAGTAA ATACGGCTTT
6051 TTAGCACAGT AACTCGAGCT TGAAGAGCAT CCTGATTACG GTGATAACGA
6101 GGATAATTAA GATAAAATGG CTTCTATGAA CGATAATACT TTGGTCACTG

```
6151 GGTCGAATGA ATATCCGAAA AGCCGAATGA GTTTTCCTAA ATATCAAATG
6201 TGGGGCTAAG ATGAGAGTTA ATGGACGTGC AATACGTTTC TAAAGCAAGC
6251 TATATTCACG GACAATGCAT TCCTGTCATT CAAGAACACG ATTCGTCTTG
6301 TTGCTCTAAA TCAGTTTCGA CCGTACAGTG CGTAAAAGAA TGTTGGAACC
6351 TACAAAATAA GTGGCGTGGA GTTTGTAAAC ATAACAACGG AGACAGATTT
6401 GGCCATGATA ACGAGGGAAA CCGTGGGAGG GGAACAGACT GATAAGCAGG
6451 ATGACAAGTC CCAGGAACCA ATAAAAGCTA AACCGGCTAA ACCGGCTAAA
6501 CCGGCTAAAC TAACGCAACC CCGGGAAATC GTCCGTCCTT CCTAATATTT
6551 ATACTCGCTA ACGAGCAAAT CTTATATTTT CAAACTAACG TAACGTTCTT
6601 CACGCAGAAT AAGAAAATGT GTCCAGGGAT GCATTTCGAT ATCTCCATCG
6651 GTTGTCCGGA TGGAGACGAA AAAGTTTGAC ATTGCGAGAC AACATGGGAT
6701 ACCCACGGGG GAACTACCGG GGATTTCGAA GCTTCAGAAC CTTCGAAACA
6751 CATTTGCTGC TGCGTCATCT CGCCAGGTTC TGCCTATACT ACAGATCGCA
6801 CACACATAAC AAGCGTCATC AGAGGCAGTT TGTTTAATTT AATTTTGCCA
6851 TTTTTATTGT GTGTACCACT ATTTATACCC AAGGCCAATA GCGGCAGACC
6901 CTCAACCCCC GCCCCTCCGC TAGCAGCCGG CCAGGAGGGC AGATGTGAAG
6951 CTTCTGTACA TGCTATGAGC GATTATTTTT CGAGAGACAG TACCGAGGGT
7001 AAATTATGTG CTGATGCTTC AAATGCATTC AATATATCAA ACCGTAAAAA
7051 CATTGAATGA ACTTCTTATC ACACCCATCT CAGACACTTC ATGTTTCTTT
7101 TCACATTTTG ATGTTCCGAT TGATTACAGG GCCTAGTGAC GATATACCCC
7151 GATGATACTG CCGGGAATGA GGATTGGAAA GAAGGGGCAA GGGCCCCAGG
7201 CCCCGATCTC CCCTTTGTAT CCCACACAAT CTGCTGATAA AAGGGCAGGA
7251 TATAAACCTC ACGTCGCTTA AAAAATAGCT TAAGTCTGCC ACTGACGATT
7301 GTAAAGCTGT TTGTAGTAAG TACGGCTTTT TTTAGCACAG TAAAGGGGTA
7351 CACACGTTCT AAATCTAAAA CTGTCGTGAG CTTAACATCC GAACCAGAAG
7401 AGCCGAGGAA CCTAACTGTG ACAACAACGA TAGTAAAGAT TTTATTAACA
7451 TATGACACAT TATGATTTGA TAATTAGGAG TTTATAAATC TGACTTTATG
7501 TATAAATTGA TAGCGAAGAT GAAATGGCTT CTATGAACGA CACTACTTTG
7551 GTCAATACCA GCCTAGGTCG AATGAATATC CAAACGATTC GTCTTGTTGC
7601 TCTATGTCAG TTTCGACCGT ACAGTGCGTA AAACTGATGT CCGGCGCATA
7651 GAATGTTCGA ATGTAAAAAA TTAGTGGTGT GGAGTTTATA AACATAACAA
7701 TGGGGAAAAA GGCCAAACGG ACGAAACCAT GGGAGGGGAA CAGACTGAGA
7751 AGCAGGATGA CTCAGGAACC AATTAGAACT AACACAACCC CGTGAAATCC
7801 TCCAC
```

The transcript and protein sequences of *PbaTransib_0868* were predicted by AUGUSTUS.

AVPN01000868.1:1-7805	AUGUSTUS	transcript	2273	4886	0.12	+	.	ID=g3.t3;Parent=g3
AVPN01000868.1:1-7805	AUGUSTUS	start_codon	2273	2275	.	+	0	Parent=g3.t3
AVPN01000868.1:1-7805	AUGUSTUS	intron	3060	3423	0.82	+	.	Parent=g3.t3
AVPN01000868.1:1-7805	AUGUSTUS	intron	3557	3631	0.19	+	.	Parent=g3.t3
AVPN01000868.1:1-7805	AUGUSTUS	CDS	2273	3059	0.89	+	0	ID=g3.t3.cds;Parent=g3.t3
AVPN01000868.1:1-7805	AUGUSTUS	CDS	3424	3556	0.19	+	2	ID=g3.t3.cds;Parent=g3.t3
AVPN01000868.1:1-7805	AUGUSTUS	CDS	3632	4883	0.12	+	1	ID=g3.t3.cds;Parent=g3.t3
AVPN01000868.1:1-7805	AUGUSTUS	stop_codon	4884	4886	.	+	0	Parent=g3.t3

coding sequence = [atgtcaaatgagtatcaacatctcactctaactgatctcctaaacattgtcgtggacgtgggaggatggaaatggatca
atgtaccaactctaagggatttgttcatgagaagacgagtcggaattttcttttagaagaacactgtcgtgcctattgttttctccagcgtgtttat
gagcagagcaagaagaaaaagtgcaatttgtcaaaccttgttgcaacatttggtgacgaggaaaagattttctgcactgaatcactggcagttgatga
tccacatgcacaagtattagtaactacagaggagatagagatggcttgtggagatacagaggaggaggatgctgcaagtggggatacagaagaactgg
ctagcgtgaccgtggatactacggaggatcaggatgcgaatcaacctgcaaagaaggccagaaaacacctactgaacatgactaaacagagccagtac
cagagaacggaggagctatacaagcatctcctttctaccgccgaagaggaggatgttacaccatcttggctggcacagctgctgctggagaggtcagc
ttaccgttcagacagaggagctgccaagggaaacaggactacacactcgatggaggtgccccacgcggcttttctccagcaggacctgttcttgggaa
aacagcagtatgtccgtatgaagaaaggcttagcaccctatgtttctttacccaactggcctgaagtgaggaaatttcaactttctcttacccctaaa
cacactcctactaccaatccaggcaccagtttcaattttgaagaggctctgctgatgactattgaagcactgggaaaatcacttaacatcacttggcc
acccaacataaatgtatacatgaaacatggtggggatggctcgggttcccacgccagggaaactggtcagccactgtatgttaactcctcctccaact
ccccacatacccaccgtcctctcttcattgccatggggaaggaggaagtaatggacgatgacgtggagaaggtcatgaaggagattaaggagttgtta
gagggaccagtgaggatgtgcgacactaatgtctacatagacggtaagatgtctctggtcgattcaaagttccggaaaaacatgctaggtctaggggg
agcatggtgcatgctgtgccacgtcacattggagactgggtgcggtagaggtttcctggagccgtatgatttttttcttcataactaggtctgggaagg
aggttcaggatacatatgacaatctgaaggatgatgaggggtgtgtccctactaaaacaggcgattatgccgagagggctgggactaccgaggcaccc
ctaccccaagactcagaaacactggactccctcatatttgtatctccactgcatgccatcatgagagtgttcagctgggttttaatcctaatgtatca
tctgatggcgggaatcctccgttggtcagaggggaaggtggttttggggagttcctatcagttctacgttgctgccaagaagaaggcgaacgaggtgg
tcaaggccaagacgggtatccttatggatgcgcctgatccgacgggtaaaggggggcaacactctcaaggggggacatttgcaagcgacttctcgggata
gagggaaatagggaggtactagttagcctggttccgagacaataccagcagtcgttccgtgccctgctggagggtctgtgggtcgttattcatgtata
ctcatccgacccagacccaggcagatatgtcgatgtgaatgtgttcaagatggagtgtttggacttgtaccacttgatccttgagaacttcgggaatc
gacctgaccaccgctgggtgaacttgtcaccgactctccactcagttctagcacactcatgggagctcatagaggccaacaacaaccagagtctggga
gccttttccgagagcggacttgaatgtaacaacaagtttctccggataatacgtgaggtcaagagcagaaagatcaatgagacagtgtgccaccagga
taccttgaataggatgtgggataagtcctgccccccttgctaacacataccgtccaatcgtgcacccacggccaggagctaggaaacaccagacggaca
ccctgagtgagcgaattgccgcttgttatattgattga]

protein sequence = [MSNEYQHLTLTDLLNIVVDVGGWKWINVPTLRDFVHEKTSRNFLLEEHCRAYCFLQRVYEQSKKKKCNLSNLVATFGD
EEKIFCTESLAVDDPHAQVLVTTEEIEMACGDTEEEDAASGDTEELASVTVDTTEDQDANQPAKKARKHLLNMTKQSQYQRTEELYKHLLSTAEEEDV
TPSWLAQLLLERSAYRSDRGAAKGNRTTHSMEVPHAAFLQQDLFLGKQQYVRMKKGLAPYVSLPNWPEVRKFQLSLTPKHTPTTNPGTSFNFEEALLM
TIEALGKSLNITWPPNINVYMKHGGDGSGSHARETGQPLYVNSSSNSPHTHRPLFIAMGKEEVMDDDVEKVMKEIKELLEGPVRMCDTNVYIDGKMSL
VDSKFRKNMLGLGGAWCMLCHVTLETGCGRGFLEPYDFFFITRSGKEVQDTYDNLKDDEGCVPTKTGDYAERAGTTEAPLPQDSETLDSLIFVSPLHA
IMRVFSWVLILMYHLMAGILRWSEGKVVLGSSYQFYVAAKKKANEVVKAKTGILMDAPDPTGKGGNTLKGDICKRLLGIEGNREVLVSLVPRQYQQSF
RALLEGLWVVIHVYSSDPDPGRYVDVNVFKMECLDLYHLILENFGNRPDHRWVNLSPTLHSVLAHSWELIEANNNQSLGAFSESGLECNNKFLRIIRE
VKSRKINETVCHQDTLNRMWDKSCPLANTYRPIVHPRPGARKHQTDTLSERIAACYID]

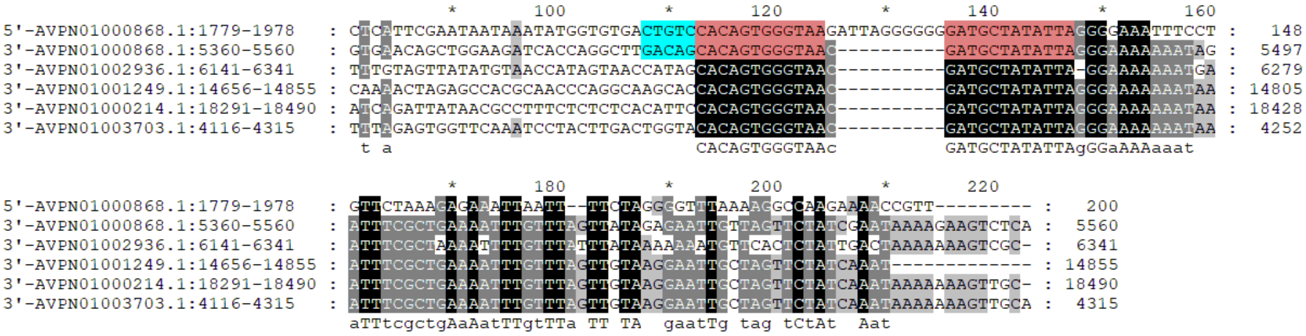

Figure 11 Alignment of 5’-and 3’-flanking sequences of PbaTransib_0868 showed its potential TIRs and TSDs

13. PbaTransib_8175

>AVPN01018175.1:1-2571 Pleurobrachia bachei scaffold00133, whole genome shotgun sequence

```
   1 GCCGGAACTA AGGAAATTCC AGCTATCACC TACACCCACC CTTACTCCAA
  51 CAAATCAGTC AGGTACAAGC AAATAGCAAT TTTCAAAAGT GTGCATGCAT
 101 CCCCTTTGCT ACTAGAACTT CTTACTCTCT ACTTATTGCT GGTTCAAATA
 151 TTTCATTCCT TTCCAGAAAA TGTGTTATTT GCTGGTTAAT TACTACACAT
 201 GCTATGAGTT ACCGAAAATA TCGATGATAC TGGTTAGTAA CGGATTTGAT
 251 TTAAGTTAGT AAAACGAGTG ACTGTGAGTT TAAAAAGGGT TCGTTTTCTC
 301 TCTCAAAATC TCTCTTATTT ATACCTCTCA GGCATCGGTG TGAAATTTCA
 351 AGATGCGATA CAAATGACTC TCAACGCACT GTTCCAGTCC CTGAATATTA
 401 CATGGCCAGC ATCTATCACT GTATATCTAA AACACGGGGG TGATGGGTCC
 451 GGTTCACATG CGATTTATCA TCAGTTAGAC AATACACCAA CACACAACAT
 501 GTTTTTATAC ATGTTTTGTA TCTTACGGGT AGAGGACAGA CAGACGGGGC
 551 AGGTTATCTA TGTCAACGAG TCACCAAACT CTCCCCACAC CCACCGCCCA
 601 ATTCTAATCG CTATGGGTAA AGAAGAGACC ATGGACACTG ATGTGGAAAG
 651 TGTGATGAAG GAGGTGAAGG AAATATTGGA GGGACCTGTC ATGATGGGAG
 701 AGACGGAGGT GTTCGTGGAT GCCAGAATGT CCCTGGTAGA CGGCAAGTTC
 751 AGAAAGAGTG TGTTGGGGCT TGGTGGAGCC TGGTGTATGC TTTGCCATGT
 801 TACACTGGAA ACAGGGTGCG GTAGGGGGGA CCTCGAGCCG TTTGATTTCT
 851 TTTTTATTAC CCGGACAGGG TCTGAGATAC AGGAAAAGTA CGACCAGTTG
 901 AAGGACGAGG AGGGCAATGT GCCAAGGAAA ACTGGGGACT ATGCCGCGAG
 951 GTCGGGTACT ACAGAGGAGC CACTGCCAAA GGATAGTGTC ACGATGGAAT
1001 CTCTTCTGTT TGTTTCACCT CTCCATGCTA TTATGCGTGT TTTTTCGTGG
1051 GTGCTGTTGC TGATGTACCA TCTAATGAGT GATACATACA AATGGTCGGA
1101 GTCAAAGACC GTGCTTGGAC GATCCAACCA GTTCTATGCA GCAGCAAAGG
1151 TGAGAGCCAA TGAGGAGGTC AAGGCTAAGA CTGGGATTGT CATGGACGCA
1201 CCGGATCCCA CAGGAAGGGG AGGGAATACA TTGAAAGGAG ACATATGCAA
1251 GAGGCTACTC AAGAACGAAG GGGCGAGGGA AGTTTTGGTG AGTCTGGTAC
1301 CTTCAATTCA CCGCCAGGCT TTTCGAGCCC TTTTGGAGGG CCTATGGGTC
1351 ATTCTCCATG TGTTCACCAT TAACGCCGAC CCTAACAGGC AGGTAAATGT
1401 AGACGTTTAT AGGATGGAAT GCCTGGATCT CTACAAATTG ATTCTTGAGA
1451 ACTTTGGCAA TAGACCTGGT CATCGTTGGT TAAACATCTC TCCAACTGTT
1501 CACTCTCTAT TGGCTCATTC ATGGGAGCTT ATCGAGGCTA ATGGCTGTCA
1551 GTCTCTTGGT GATTACTCTG AAAGTGGGCT CGAGTGCAAT AACAAGTTTC
1601 TACGTTTTAT TCGAACCAAC AAAACTAGGA AAACCAGTGA GAGAGACTGT
1651 CATAAGGACA CCTTTTATAG GATGTGGGAC AAGTCTTGTC CATTTGCCAA
1701 CATGTACCGT CCTATAGTCC ATCCCCGACC TAGGGCTCGG AAAGAGTGTA
1751 CGAGTTCCCT GACAGAGCGG ATTGAGGCTT GTTTCATTTA GTTTGGTGTC
1801 TTTGAAGTTC AAATTGAACG ATTTTATGAT GAATTTGTGA TTCACCCCCG
1851 TATACTTGTA CATTTTAGCT CTTTTTTAAC AACTTTTTAC CAAAACTAAA
1901 ATATTTGTTT GCTTAAGAAT GGTGTTTTCC GGTGGCTAGC GCTACATAAT
1951 TCTTGTTGTA GGACACTCAT AAGGGACTAT ATTAACACGT GTAACCATTG
```

```
2001 AATTTCGCGG AAAATCGCGG AATAGAAACT AATTTTACTT TTATTAAAGT
2051 AAAAAAGGGG TAATTTTACC ATGCGCACTG AAACATTCAT CACCTAAGAA
2101 TGGAAAATCC AATTATCATA AAGTAAAAAA CATTGAAAAG ACAAAATTAT
2151 GGTGAGTATG GCTAATATAT AGCTTGTCAG AGGTGACAAA CTTATAATTT
2201 AAGAAATAAT ATCTGAAAAT CGATAAATTT GACTGCCAAA AGTTGATTTA
2251 ACTGCCAAAA TTTAAATATG CGCATATCAT TTTAGTCATA ACTCGGTCAA
2301 ATCTAAGTAT TTTTTGATGC GGTTTTTTTT ACTGGCCTAA TCTAACAATT
2351 TCCTATTAGG TAGCAAATTT GCAGCAAAAT CGATTTTTTT TCCTAAACGA
2401 CCATCGTTAC CCACTGTGTG CCCATTGTAC CTGCATACTA AATAATATTT
                         <<<predicted 3’TIR
2451 ACTAATTATT AGTAAATATC ACTTGGGGAC CAGCTAATAA AAAGTACCCG
2501 GGTATGTTGG CTAAAAAGAA CTCGGGTAAG TGGTTATCAA AAGAACGTGT
2551 GGAGACGTGT GAAGAGCCAT A
```

The transcript and protein sequences of *PbaTransib_8175* were predicted by AUGUSTUS.

```
AVPN01018175.1:1-2571 AUGUSTUS transcript   1    1791 0.14 +    .    ID=g1.t2;Parent=g1
AVPN01018175.1:1-2571 AUGUSTUS intron   1    166  0.59 +    .    Parent=g1.t2
AVPN01018175.1:1-2571 AUGUSTUS intron   255  331  0.56 +    .    Parent=g1.t2
AVPN01018175.1:1-2571 AUGUSTUS CDS  167  254  0.57 +    0    ID=g1.t2.cds;Parent=g1.t2
AVPN01018175.1:1-2571 AUGUSTUS CDS  332  1788 0.18 +    2    ID=g1.t2.cds;Parent=g1.t2
AVPN01018175.1:1-2571 AUGUSTUS stop_codon   1789 1791 .    +    0    Parent=g1.t2
coding sequence = [aaaatgtgttatttgctggttaattactacacatgctatgagttaccgaaaatatcgatgatactggttagtaacggat
ttgatttaagcatcggtgtgaaatttcaagatgcgatacaaatgactctcaacgcactgttccagtccctgaatattacatggccagcatctatcact
gtatatctaaaacacgggggtgatgggtccggttcacatgcgatttatcatcagttagacaatacaccaacacacaacatgtttttatacatgttttg
tatcttacgggtagaggacagacagacggggcaggttatctatgtcaacgagtcaccaaactctccccacacccaccgcccaattctaatcgctatgg
gtaaagaagagaccatggacactgatgtggaaagtgtgatgaaggaggtgaaggaaatattggagggacctgtcatgatgggagagacggaggtgttc
gtggatgccagaatgtccctggtagacggcaagttcagaaagagtgtgttggggcttggtggagcctggtgtatgctttgccatgttacactggaaac
agggtgcggtagggggggacctcgagccgtttgatttctttttttattacccggacagggtctgagatacaggaaaagtacgaccagttgaaggacgagg
agggcaatgtgccaaggaaaactggggactatgccgcgaggtcgggtactacagaggagccactgccaaaggatagtgtcacgatggaatctcttctg
tttgtttcacctctccatgctattatgcgtgtttttttcgtgggtgctgttgctgatgtaccatctaatgagtgatacatacaaatggtcggagtcaaa
gaccgtgcttggacgatccaaccagttctatgcagcagcaaaggtgagagccaatgaggaggtcaaggctaagactgggattgtcatggacgcaccgg
atcccacaggaaggggagggaatacattgaaaggagacatatgcaagaggctactcaagaacgaaggggcgagggaagttttggtgagtctggtacct
tcaattcaccgccaggcttttcgagcccttttggagggcctatgggtcattctccatgtgttcaccattaacgccgaccctaacaggcaggtaaatgt
agacgtttataggatggaatgcctggatctctacaaattgattcttgagaactttggcaatagacctggtcatcgttggttaaacatctctccaactg
ttcactctctattggctcattcatgggagcttatcgaggctaatggctgtcagtctcttggtgattactctgaaagtgggctcgagtgcaataacaag
tttctacgttttattcgaaccaacaaaactaggaaaaccagtgagagagactgtcataaggacaccttttataggatgtgggacaagtcttgtccatt
tgccaacatgtaccgtcctatagtccatccccgacctagggctcggaaagagtgtacgagttccctgacagagcggattgaggcttgtttcatttag]
protein sequence = [KMCYLLVNYYTCYELPKISMILVSNGFDLSIGVKFQDAIQMTLNALFQSLNITWPASITVYLKHGGDGSGSHAIYHQL
DNTPTHNMFLYMFCILRVEDRQTGQVIYVNESPNSPHTHRPILIAMGKEETMDTDVESVMKEVKEILEGPVMMGETEVFVDARMSLVDGKFRKSVLGL
GGAWCMLCHVTLETGCGRGDLEPFDFFFITRTGSEIQEKYDQLKDEEGNVPRKTGDYAARSGTTEEPLPKDSVTMESLLFVSPLHAIMRVFSWVLLLM
YHLMSDTYKWSESKTVLGRSNQFYAAAKVRANEEVKAKTGIVMDAPDPTGRGGNTLKGDICKRLLKNEGAREVLVSLVPSIHRQAFRALLEGLWVILH
VFTINADPNRQVNVDVYRMECLDLYKLILENFGNRPGHRWLNISPTVHSLLAHSWELIEANGCQSLGDYSESGLECNNKFLRFIRTNKTRKTSERDCH
KDTFYRMWDKSCPFANMYRPIVHPRPRARKECTSSLTERIEACFI]
```

```
                                       *         20         *         40         *         60         *         80
AVPN01018175.1:2318-2517   : TGCGGTTTTTTTTACTGGCCTAATCTAACAATTTCCTATTAGGTAGCAAATTTGCAGCAAAATCGATTTTTTTCCTAAA : 2397
AVPN01004258.1:5768-5967   : TGCGGTTTTTTCTATTGGGCTAATCTAACAATTCTTTATCATGTGACAAAGTATCAGCAAAAATTAATTTTTTTGCTAAA : 5847
AVPN01002571.1:5484-5683   : -GCGATTTTTTCTATTGGGCTAATCTAACAATTCTTTATCATGTGACAAAGTATCAGCAAAAATTAATTTTTTTGCTAAA : 5562
AVPN01000429.1:9279-9479   : ---------TTCTATTGCGCACAGCTAACAATTCTTTATCATGTGACAAAGTATCAGCAAAAATTAATTTTTTTGCTAAA : 9349
AVPN01000039.1:76953-77152 : ----ATTTTTTCTATTGGGCTAATCTAACAATTCTTTATCATGTGACAAAGTATCAGCAAAAATTAATTTTTTTGCTAAA : 4884
                                  ttttTTcTAtTGggCtaAtCTAACAATTcttTATcAtGTgaCAAAgTatCAGCAAAAattAaTTTTTTTgCTAAA

                                       *        100         *        120         *        140         *        160
AVPN01018175.1:2318-2517   : CGACCATCGTTACCCACTGTGTGCCCATTGTACCTGCATACTAAATAATATTTACTAATTATTAGTAAATATCACTTGGG : 2477
AVPN01004258.1:5768-5967   : CGACCATCGTTACCCACTGTGCAGAGTGTCCCGACAGTATTCCCTGGAAGTTCCAGAGCTGAAACGCTTTAATTTAAGAT : 5927
AVPN01002571.1:5484-5683   : CGACCATCGTTACCCACTGTGCAGTGTGTCTCCAAGAGAATCCCGAAGTGTACCAGAAGGTTGTTAAAACTCTGCAACTG : 5642
AVPN01000429.1:9279-9479   : CGACCATCGTTACCCACTGTGCACCCATTGGGTGCGCCTCAAGTAAGGGTTTTTTCAATAACCCTCCCGGCTTCGCCGGC : 9429
AVPN01000039.1:76953-77152 : CGACCATCGTTACCCACTGTGCGCGATCTGTAGGCAGAACCTGGCAACCAAAACACTGTATAAGCCTTACCTCCCCCTTC : 4964
                             CGACCATCGTTACCCACTGTGc      T                     t     a

                                       *        180         *        200         *
AVPN01018175.1:2318-2517   : GACCAGCTAATAAAAAGTACCCGGGTATGTTGGCTAA---AAA------- : 2517
AVPN01004258.1:5768-5967   : AACCTTTTGTCCTTTCTACATGTACCCGATATCGATTAA-T--------- : 5967
AVPN01002571.1:5484-5683   : TATGTCGATGAAAAAGACCTTGTATCGTTAT---------TTGCGACATT : 5683
AVPN01000429.1:9279-9479   : TGGTTCTTTTCGCCCCTTTCAACGTCAATAACACGGTTGCTATGGCAACT : 9479
AVPN01000039.1:76953-77152 : TTCAGATATGTGTACACTCACCACTGGAGTGCAACTGA--AAGGGC---- : 5008
```

Figure 12 Alignment of 3’-flanking sequences of PbaTransib_8175 showed its potential 3’TIR.

Fungi

Massospora platypediae

14. MplTransib_1482

```
>QKRY011021482.1:1-4007 Massospora platypediae k141_1105921, whole
genome shotgun sequence
   1 ATGCGGCGGT TCAGTAACCC ACCCACTATT TAGGCAGCGA GACAGAGATA
  51 TATCCGCCTT TGTGAGAGAA GTCGCGGCCG CATGAGAGAT GTCAGCTGGC
 101 CACGTAGTCA GCCAAGTCCA GGTCCCAGTG CTACCAAAAG GAGATCTTAA
 151 TTACACCTAA TAGGAGCGAG CTGCACCGGA CCGACAGACT AGGCTGAGAC
 201 CGGTTTTGCA ATAGTGCTGT CTAAGCGGAT GCGTGCCGTC ACGTCAGGCG
          >>>Potential 3'TIR and TSD
 251 GCGCACAGTG GGGCCAAATG CCAAAAAGCT GGCGAAAACA TGAAGACGCT
 301 TTTCAATTTT TATGAAATTA TATACATAGG GGTTTCTGAG GTTGCTGATT
 351 ACGAATCTGA TGTCAAAATT ATGAAAACAA AATGGCGGAG CCAATATGGC
 401 GGACATGTTT TTTTCAAAAA ACATTTAATT TTTACGAAAT TGGGTATGTG
 451 GGTGTTTTTG AGGTCGCTGA TTACGAATCT GATGTCAAAA TTATGAAAAA
 501 CAAAATGGCG AAGCCAATAT GGCGGACATA TTTGTCAAAA AACATTTAAT
 551 TTTTACGAAA TTGGGTATGT GGGTATTTTT GAGGTCGCTG ATTACGAATC
 601 TGATGTCAAA ATTATGAAAA ACAAAATTGC GGAACCAATA TGGCGGACAT
 651 TTTTGTCAAA AAAACATTTA ATATACCTAT ATAAATTAAC ATATAATATA
 701 ACCTATATAA ATTAACAACT AGAAATGTTG AGTAAATATT GAAAATATAA
 751 CATAAGATAA CATAACATAA AAGTTTATAT ACTATCAGTA GAATCATCCG
 801 AATCATTACT GGCGTCCTCG GTCTCTTCTG GAAAACTAAT GGGGGTCGCG
 851 GGGGGTATCA AGAGACTCAA AGCATCCTTG TCAAATGTTG AAAATTTCTT
 901 TTTGGACTTT TTTCGTAAAC TGGAAATAAA TGGGTCAGAC GTGATGAGCA
 951 ATAAGTGCAG TACATCTTCA TTTGTTTTAG TGCGTGACAT TTTGCGTGAA
1001 AAGCTTTCCC TGTAATTCTT GAAATCCTTG TTTCTTGCTT CTTGGGCTTC
1051 TTCACTTAAC TGCCCTATCG GAAGTAAAGC TTGGCTTATT ATGTCTGAGC
1101 CATGGAGCAG CACCTTGTGA ACAGTTGGAG GCATTGGATA CCAGGAGTAT
1151 AGGTTTATAA AATGATTTGC TGTTTCAGTA GCATACACGC GGAATTTTTC
1201 CACGTCAATT TCGTGGTTAC TAGAAATAAC TTCCAATATT ATCTTAAATT
1251 TTCTTATTAT GTCAATATCC AATCCAGTGA TATTCGCCGA CATCTCTAAA
1301 TTATTAAAGA AACGTCTGGC TGTGTTGCCA TCATTAGTGT TTCCAAATCC
1351 CTGCTTCGGC ATGTCAACCA ACAGTCCCAT CTCAGATTTA AATCGTTCCT
1401 GAATATGCTT TTTTGTGTCA GCAACAATTT GTTTGTTTTC TGCTCCACGA
1451 ACTTGCCATT TTCGTACAGG TAGTTTGTAC GAAATATGTA GTAAACACTC
1501 AAAAAACCTT ATGCGGGCGT GAAGCACTGA CATTCCAAAT TCATGCCTGG
1551 AAGTGTCTAT TGGGTTTGTC TGCAAAACTT TTTCAACGTT ATTAAAATCT
1601 CTTGATGTAA GTCCGCAGAT GAAACATTTT TGTGTTGATG TGTTGTCGGT
```

1651 CATGGCATTA CACACTTTCC CATCCACCAT AGTTAACAAC ATTCTGTATG
1701 TAATTTTTAT TTGCGTGTCA CTGAACTCAT AAACCATAGG CCTTAAGTCC
1751 TTTATTTGAT CTTCTATGTG CTTTCGCTCA TTTTTTGATA ATGCTGTTGT
1801 TTCACGTGCA AATTGGAACC TAATAGGTCT GCAAAAGCGT GTTGAAGAAG
1851 TCACTGTATT TTGCCAAATT ACTGTTGAAC TTTCTTCTCT AGGATTATCT
1901 GAAATGAGTC TTATGGGTAC AAAAGAACAC ATGAAAATAT TCGCATCAGA
1951 AGCATTTGGA TCACTGGCGA ACTGTTGCTT GTACTCTGAT TGCGCAGATC
2001 CGTCAAACCC CCACTTGCAA AAAAGAAGCA GATTTCTCAA GGTGTCGTGT
2051 TTCTGAGTTT GCAACACTTC TTTCTGAGCT TCTAATAGGC GAGATGCGGT
2101 ATGATCAATG AGTGCCTGAA GTTTTACTTC CACGAACGTC TCCGTCACTA
2151 TCATGCTTTC TTTTGGTGGA TAACATTCTC TTTTAGCAGC TTGAATCACT
2201 TTGTAAGAGG GGAATCGAGC TTTGTCGTGG TCTCTTATAA TGTTATATTG
2251 ATGTCTACTT AGTTTTGCAT CAACATACAT TGCGAGGGAT TCCGCCCCAT
2301 TAAGCTGTAC TTTTGATGAC TCATCAATTC TTTTGTACGC GTTTCGGTAT
2351 TTCGCTGCTT GTGTTGGTGA TCCCATTTAA GTAAGAGGCA TCAACATTAC
2401 CCGCAAAACG CAAACTCATT TGAGCTGCGA AAGACAACTC ATAAGGTGAA
2451 TGTAGAGATC TTAACCGTTC GGTCTTTTGC CGCTTTGACC TGTCAGAAGA
2501 GTCTTGAAAC GTTTTTGTAG AACGACCCAT TTTTTTACTT GTTGTAGTTT
2551 CATTGACATT ACTGTAATCA ATAAAACTGA TAGAACCTCC TAACCAAGCT
2601 TCGTTTTTGG TCAGAAACCT TTCTTCTTTC CGACTAGCAT ATTGCCATCT
2651 ATTTTTAAAA TCCGATGTAA AGAAGGAAAT TTTTTTGTTT AGATCATGAA
2701 TAAAAGTTTT TGGACACGCT GTTTTTGATT CAATATATTG CTTCACGTAA
2751 GTTATTTGGT CTTCAATCTT TGTGTGGCCA GATTTCCTCA TCTTCTCATA
2801 CAGCATCTTT CTTGAGAGAC TTATCTCTCT GGTCCTCGAC CCTTCAAAGA
2851 TAAGAAAAAA AATATATATT ATATCACTTA CGTGATTGTA CTACATTTAT
2901 TAAGATAAGT AATAAAACTC AAATTGCATA AATGCAAGTA AAATTTTTAA
2951 AAATTTGTTT TTTATTTAAA CTTTAGTCAA GATTGATCTA AAAAACAAAT
3001 TTTAATAGTT TCTTAAATTA CCCGGGAGTG AAAATTGCTA GGTTAACCCA
3051 GCTACAACAA GTAAGAGTGA AACAGACTTA TGCAGGTTAT GTACGTTAAT
3101 AGGTTACCTG ATCAGGACCA GAGCAATTTT TCCTCCAGGG TACACATAAA
3151 TTTCGGGTGA TACATAATAC AGTAACAAGG GCGGGCTTCA ACCACTGCTA
3201 ACTGAGTACC TATGTAATTA CTTATTTATT ATGGTGGAAT TATAGTCCTA
3251 TATCACGAAA AGGTGCAGAA ATTCGCAGCT TTTTTTTAAT GTTTTGTTAA
3301 GAAGAACTAT TATAGAGACG TGCAAATATA ATTTCAAGCA CATGACATGC
3351 GCTAACATTT ATTAAATGTT TACGTACACT CACTAAAAAC TCGGAAATCT
3401 TTCAAAAACA GCGTGTCATT ATTCAACACA TTGTTAAGTA CACATATAAA
3451 CAGTCAATTA TAAATATCAG GGTATTATGT ACTAACCTGA TGGTAGAGAC
3501 GACATTCTAT GTAAACGTTT GTTTACCAAA CTGAAAACAA CACAATCAAC
3551 GCACAACTTA TTAATGTTTA TTTACACTCG TTTTTAATTT TAATTTTGTA
3601 TTTTTTACGT TTTTCTGAAT AAAATGCTAC ACAAATTATA ATGAACAATT
3651 ATAAATATTT TTAATGTTTG ACTCAATATA GCATTGACTT TTGTTTCCAT
3701 AGTAACGCAA AAAGTAAATA GTGTCATGTA TGTCAAATGT AAAAATACCA
3751 TACATAGAGA GCATTTTCAT CTTCCTTTAG CTGCCATCGA AACAAAGGCC
3801 CAGTCGTCTC AATGCAATAC CCGTTTATGA AATGGTATTC ACTTACTTTT

```
3851 TCTCAGATAA AAACTTTAAA AGCAGATATT AGCAGCTGTT TCTTGTATTT
3901 TTTGTTAGGT ATTATTCCAT TCTATTATCT GCAATAAGTT AGAACACCGC
3951 GCACTACAGC GCAAAACTAG TTTTACATTA ATAAAGACAT GATTTTACTC
4001 AAAAACG
```

The transcript and protein sequences of *MplTransib_1482* were predicted by AUGUSTUS.

```
QKRY011021482.1:1-4007	AUGUSTUS transcript	775	2806	0.02	-	.	ID=g1.t3;Parent=g1
QKRY011021482.1:1-4007	AUGUSTUS stop_codon	775	777	.	-	0	Parent=g1.t3
QKRY011021482.1:1-4007	AUGUSTUS intron	2355	2400	0.09	-	.	Parent=g1.t3
QKRY011021482.1:1-4007	AUGUSTUS CDS	775	2354	0.09	-	2	ID=g1.t3.cds;Parent=g1.t3
QKRY011021482.1:1-4007	AUGUSTUS CDS	2401	2806	0.02	-	0	ID=g1.t3.cds;Parent=g1.t3
QKRY011021482.1:1-4007	AUGUSTUS start_codon	2804	2806	.	-	0	Parent=g1.t3
QKRY011021482.1:1-4007	AUGUSTUS protein_match	1499	1546	3.05	+	0
    ID=pp.g1.t3.unknown_A;Target=unknown_A 1 16;Target_start=404;
QKRY011021482.1:1-4007	AUGUSTUS protein_match	1695	1775	2.47	+	0
    ID=pp.g1.t3.unknown_B;Target=unknown_B 1 27;Target_start=328;
QKRY011021482.1:1-4007	AUGUSTUS protein_match	1791	1841	3.57	+	0
    ID=pp.g1.t3.unknown_C;Target=unknown_C 1 17;Target_start=306;
QKRY011021482.1:1-4007	AUGUSTUS protein_match	1854	1934	4.09	+	0
    ID=pp.g1.t3.unknown_D;Target=unknown_D 1 27;Target_start=275;
QKRY011021482.1:1-4007	AUGUSTUS protein_match	1983	2015	6.55	+	0
    ID=pp.g1.t3.unknown_E;Target=unknown_E 1 11;Target_start=248;
QKRY011021482.1:1-4007	AUGUSTUS protein_match	2016	2039	5.85	+	0
    ID=pp.g1.t3.unknown_F;Target=unknown_F 1 8;Target_start=240;
QKRY011021482.1:1-4007	AUGUSTUS protein_match	2061	2102	3.53	+	0
    ID=pp.g1.t3.unknown_G;Target=unknown_G 1 14;Target_start=219;
QKRY011021482.1:1-4007	AUGUSTUS protein_match	2136	2270	3.91	+	0
    ID=pp.g1.t3.unknown_H;Target=unknown_H 1 45;Target_start=163;
QKRY011021482.1:1-4007	AUGUSTUS protein_match	2298	2360	4.78	+	0
    ID=pp.g1.t3.unknown_I;Target=unknown_I 1 21;Target_start=135;
QKRY011021482.1:1-4007	AUGUSTUS protein_match	2376	2399	5.66	+	0
    ID=pp.g1.t3.unknown_J;Target=unknown_J 1 8;Target_start=135;
QKRY011021482.1:1-4007	AUGUSTUS protein_match	2463	2537	5.82	+	0
    ID=pp.g1.t3.unknown_L;Target=unknown_L 1 25;Target_start=89;
QKRY011021482.1:1-4007	AUGUSTUS protein_match	2541	2570	5.07	+	0
    ID=pp.g1.t3.unknown_M;Target=unknown_M 1 10;Target_start=78;
QKRY011021482.1:1-4007	AUGUSTUS protein_match	2583	2618	4.54	+	0
    ID=pp.g1.t3.unknown_N;Target=unknown_N 1 12;Target_start=62;
QKRY011021482.1:1-4007	AUGUSTUS protein_match	2625	2654	8.05	+	0
    ID=pp.g1.t3.unknown_O;Target=unknown_O 1 10;Target_start=50;
QKRY011021482.1:1-4007	AUGUSTUS protein_match	2664	2702	10.9	+	0
    ID=pp.g1.t3.unknown_P;Target=unknown_P 1 13;Target_start=34;
```

QKRY011021482.1:1-4007 AUGUSTUS protein_match 2718 2780 3.45 + 0
ID=pp.g1.t3.unknown_Q;Target=unknown_Q 1 21;Target_start=8;
QKRY011021482.1:1-4007 AUGUSTUS protein_match 2784 2870 4.47 + 0
ID=pp.g1.t3.unknown_R;Target=unknown_R 1 29;Target_start=0;
QKRY011021482.1:1-4007 AUGUSTUS protein_match 2907 3092 5.24 + 0
ID=pp.g1.t3.unknown_S;Target=unknown_S 1 62;Target_start=0;
QKRY011021482.1:1-4007 AUGUSTUS interblock_region 775 1498 . - 0 ID=pp.g1.t3.iBR1
QKRY011021482.1:1-4007 AUGUSTUS interblock_region 1547 1694 . - 0 ID=pp.g1.t3.iBR0
QKRY011021482.1:1-4007 AUGUSTUS interblock_region 1776 1790 . - 0 ID=pp.g1.t3.iBR1
QKRY011021482.1:1-4007 AUGUSTUS interblock_region 1842 1853 . - 0 ID=pp.g1.t3.iBR2
QKRY011021482.1:1-4007 AUGUSTUS interblock_region 1935 1982 . - 0 ID=pp.g1.t3.iBR3
QKRY011021482.1:1-4007 AUGUSTUS interblock_region 2040 2060 . - 0 ID=pp.g1.t3.iBR5
QKRY011021482.1:1-4007 AUGUSTUS interblock_region 2103 2135 . - 0 ID=pp.g1.t3.iBR6
QKRY011021482.1:1-4007 AUGUSTUS interblock_region 2271 2297 . - 0 ID=pp.g1.t3.iBR7
QKRY011021482.1:1-4007 AUGUSTUS interblock_region 2400 2462 . - 0 ID=pp.g1.t3.iBR9
QKRY011021482.1:1-4007 AUGUSTUS interblock_region 2538 2540 . - 0 ID=pp.g1.t3.iBR10
QKRY011021482.1:1-4007 AUGUSTUS interblock_region 2571 2582 . - 0 ID=pp.g1.t3.iBR11
QKRY011021482.1:1-4007 AUGUSTUS interblock_region 2619 2624 . - 0 ID=pp.g1.t3.iBR12
QKRY011021482.1:1-4007 AUGUSTUS interblock_region 2655 2663 . - 0 ID=pp.g1.t3.iBR13
QKRY011021482.1:1-4007 AUGUSTUS interblock_region 2703 2717 . - 0 ID=pp.g1.t3.iBR14
QKRY011021482.1:1-4007 AUGUSTUS interblock_region 2781 2783 . - 0 ID=pp.g1.t3.iBR15
coding sequence = [atgctgtatgagaagatgaggaaatctggccacacaaagattgaagaccaaataacttacgtgaagcaatatattgaat
caaaaacagcgtgtccaaaaacttttattcatgatctaaacaaaaaaatttccttctttacatcggattttaaaaatagatggcaatatgctagtcgg
aaagaagaaaggtttctgaccaaaaacgaagcttggttaggaggttctatcagttttattgattacagtaatgtcaatgaaactacaacaagtaaaaa
aatgggtcgttctacaaaaacgtttcaagactcttctgacaggtcaaagcggcaaaagaccgaacggttaagatctctacattcaccttatgagttgt
ctttcgcagctcaaatgagtttgcgttttgcggcgaaataccgaaacgcgtacaaaagaattgatgagtcatcaaaagtacagcttaatggggcggaa
tccctcgcaatgtatgttgatgcaaaactaagtagacatcaatataacattataagagaccacgacaaagctcgattcccctcttacaaagtgattca
agctgctaaaagagaatgttatccaccaaaagaaagcatgatagtgacggagacgttcgtggaagtaaaacttcaggcactcattgatcataccgcat
ctcgcctattagaagctcagaaagaagtgttgcaaactcagaaacacgacaccttgagaaatctgcttctttttttgcaagtgggggtttgacggatct
gcgcaatcagagtacaagcaacagttcgccagtgatccaaatgcttctgatgcgaatattttcatgtgttcttttgtacccataagactcatttcaga
taatcctagagaagaaagttcaacagtaatttggcaaaatacagtgacttcttcaacacgcttttgcagacctattaggttccaatttgcacgtgaaa
caacagcattatcaaaaaatgagcgaaagcacatagaagatcaaataaaggacttaaggcctatggtttatgagttcagtgacacgcaaataaaaatt
acatacagaatgttgttaactatggtggatgggaaagtgtgtaatgccatgaccgacaacacatcaacacaaaaatgtttcatctgcggacttacatc
aagagattttaataacgttgaaaaagttttgcagacaaacccaatagacacttccaggcatgaatttggaatgtcagtgcttcacgcccgcataaggt
tttttgagtgtttactacatatttcgtacaaactacctgtacgaaaatggcaagttcgtggagcagaaaacaaacaaattgttgctgacacaaaaaag
catattcaggaacgatttaaatctgagatgggactgttggttgacatgccgaagcagggatttggaaacactaatgatggcaacacagccagacgttt
ctttaataatttagagatgtcggcgaatatcactggattggatattgacataataagaaaatttaagataatattggaagttatttctagtaaccacg
aaattgacgtggaaaaattccgcgtgtatgctactgaaacagcaaatcattttataaacctatactcctggtatccaatgcctccaactgttcacaag
gtgctgctccatggctcagacataataagccaagctttacttccgatagggcagttaagtgaagaagcccaagaagcaagaaacaaggatttcaagaa
ttacagggaaagcttttcacgcaaaatgtcacgcactaaaacaaatgaagatgtactgcacttattgctcatcacgtctgacccatttatttccagtt
tacgaaaaaagtccaaaaagaaattttcaacatttgacaaggatgctttgagtctcttgataccccccgcgacccccattagttttccagaagagacc
gaggacgccagtaatgattcggatgattctactgatagtatataa]
protein sequence = [MLYEKMRKSGHTKIEDQITYVKQYIESKTACPKTFIHDLNKKISFFTSDFKNRWQYASRKEERFLTKNEAWLGGSISF
IDYSNVNETTTSKKMGRSTKTFQDSSDRSKRQKTERLRSLHSPYELSFAAQMSLRFAAKYRNAYKRIDESSKVQLNGAESLAMYVDAKLSRHQYNIIR

DHDKARFPSYKVIQAAKRECYPPKESMIVTETFVEVKLQALIDHTASRLLEAQKEVLQTQKHDTLRNLLLFCKWGFDGSAQSEYKQQFASDPNASDAN

IFMCSFVPIRLISDNPREESSTVIWQNTVTSSTRFCRPIRFQFARETTALSKNERKHIEDQIKDLRPMVYEFSDTQIKITYRMLLTMVDGKVCNAMTD

NTSTQKCFICGLTSRDFNNVEKVLQTNPIDTSRHEFGMSVLHARIRFFECLLHISYKLPVRKWQVRGAENKQIVADTKKHIQERFKSEMGLLVDMPKQ

GFGNTNDGNTARRFFNNLEMSANITGLDIDIIRKFKIILEVISSNHEIDVEKFRVYATETANHFINLYSWYPMPPTVHKVLLHGSDIISQALLPIGQL

SEEAQEARNKDFKNYRESFSRKMSRTKTNEDVLHLLLITSDPFISSLRKKSKKKFSTFDKDALSLLIPPATPISFPEETEDASNDSDDSTDSI]

```
                                          *        20         *        40         *        60         *        80
QKRY011021482.1:154-354   : ---------CACCTAATAGGAGCGAGCTGCACCGGACCGACAGACTAGGCTGAGACCGGTTTTGCAATAGTGCTGTCTAA :  224
QKRY011691779.1:1967-2166 : TCTTAATTACACCTAATAGGAGCGAGCGGCACCGGA--------CTAGGCCGAGACCGGTTTTGCAATAGTGCTGTCTAA : 2038
QKRY011806857.1:21-220    : ----TATGTTTAAACGTACAGAATTACAAGCGCAAGCATCCAAATAAACAGTGAAGAGGATTTTCCAGTCAGTTTTCAAC :   96
QKRY011395157.1:207-406   : -----ACTTATTTATACACTTTAAAATTATTAGCCTTTCCAGCCAAACGTATAATTTAAACTCGTAATTGGTGAACTAAT :  281
QKRY011973351.1:21-220    : ----CTGGAAAATAACTACCATTACTTGTTGTTACATGGACACAAAGTTGTTTAAATAAACCAAGTTATGCAGACTTTTG :   96
                                          tA                             a       a      t    a g     t  a

                                          *       100         *       120         *       140         *       160
QKRY011021482.1:154-354   : GCGGATGCGTGCCGTCACGTCAGGCGGCG-CACAGTGGGGCCAAATGCCAAAAAGCTGGCGAAAACATGAAGACGCTTTT :  303
QKRY011691779.1:1967-2166 : GCGGATGCGTGCCGTCACGTCAGGCGGCG--TCTCCGCGTCCTCGTGGCTATTTAGGTGGAGAGAGAAATAGACGTTTCT : 2116
QKRY011806857.1:21-220    : AAATAACTCAGGAGATTTTCTACACGGCG-CACAGTGGGGCCAAATGCCAAAAAGCTGGCGAAAACATGAAGACGCTTTT :  175
QKRY011395157.1:207-406   : TTCGTTTAACTGACATGTTTGTTACGGCG-CACAGTGGGGCCAAATGCCAAAAAGCTGGCGAAAACATGAAGACGCTTGT :  360
QKRY011973351.1:21-220    : AGTTCGCTTGTATCGTTTTCCATACGCCG-CACAGTGGGGCCAAATGCCAAAAAGCTGGCGAAAACATGAAGACGCGTTT :  175
                                                  a  CGgCG caCagtGgGgCCaaaTGcCaAaaagctgGcgaAaAcAtgaAGACGctT T

                                          *       180         *       200         *
QKRY011021482.1:154-354   : CAATTTTTATGAAATTATATACATAGGGGTTTCTGAGGTTGCTGATTACGA :  354
QKRY011691779.1:1967-2166 : GGCGTGAGACAAACCTCGGTAGCCTGAAGGCCCCGGGTGTTCCAGGATAG- : 2166
QKRY011806857.1:21-220    : CAATTTTTATGAAACTATATATATAGGGGTTTCTGAGGTTGCTGA------ :  220
QKRY011395157.1:207-406   : CAATTTTTATGAAATTATATATATAGGGGTTTCTGAGGTTGCTGAT----- :  406
QKRY011973351.1:21-220    : CAATTTTTATGAAATTATATATATAGGGGTTTCTGACGTTGCTGA------ :  220
                            caatTtttAtgAAa TataTA ataGggGtttCtGaggtTgCtga
```

Figure 13 Alignment of 3’-flanking sequences of MplTransib_1482 showed its potential 3’TIRs.

Austropuccinia psidii

15. ApsTransib_1266

```
>QOUH01081266.1:1-2049  Austropuccinia  psidii  strain  Apsidii_AM
Supernova_641940, whole genome shotgun sequence
   1 TACCTCGAAG AAGTTTTGAA AGGACTTTCA AGTGAAGAGA AGGAAAATTT
  51 AAAACTTATA TCAAAATGGG GATGTGACGG ATCTCAACAG ACACGGTATA
 101 TGCAATCCTT ACAAAATACG AACGAAAGCT ATGACGATGC AAATATATTC
 151 CAAAGCTCGT TTTTGCCTTT GAGATTACTT GTTAGTCATA ACAGCCAAAG
 201 TAAAGTCATT TGGCAAAACC CCGTCCCGTC GAGTCCAAGA TTTTGTAGAC
 251 CTATTCGAAT CAGATTTGTG CATGAAACTA ACGACATCAC AAGAAATGAA
 301 ATTAGTTATA TAGAAAGTCA AATTGAAAAA TTAAACCTAT CTGAAATATT
 351 TTTGAATAGT GAAGTGATAA AAGTAAAGCA TCTGTTTTTA TTTACTATGG
 401 TGGATGGAAA AGTCTGCAAT GCGGCAACTA ATACCCTATC AACAATGCGT
 451 TGCTATATTT GCAAAGCAAC ATCAAAAGAT TTCAATAAAT TAGAAACAAG
 501 AAGAGAAGAA GACCCTGCGA CTTTGAAGTT TGGTCTTTCA ATATTACATG
 551 CGAGAATAAG ATTATTTGAA TCGATTCTAC ATTTGGCGTA CAAATTACCT
 601 ACACAAAAAT GGCAATCAAG GTCTTCTGCT GACAAAAAAA TTGTAAATGA
 651 GAATAAAATA CGTATACAGC AACAATTTAA AGAAGAAATG TCACTACTAG
 701 TAGATGTACC AAAAGCTGGT TTTGGAAATA CTAACACAGG CAATGTATCA
 751 AGACGATTTT TCTGTGATCC TGAGACAGCA TCTAGAATTA CCGGAGTAGA
 801 TTTAGATTTG ATAAAAAGGC TTAGAACTTT GTTAGAGGTC ATTTCAAGCG
 851 GTCACAGAAT AGATACCGAC AAATTATCAA CATTTTGTAA GGAAACTTCA
 901 GAAATTTATG TGAGACTTTA CGGATGGTAT CCCATGACGC CTACACTCCA
 951 CAAATTGTTG GTTCATGGAC CTACAATAAT AAAACATGCC ATAATCCCTA
1001 TAGGCCAGCT CTCTGAAGAA GCGGCAGAAG CGAGAAACAA ACACTTTCGG
1051 CAATACCGTA CTGATTTTGC TAGAAAGTTT TCAAAAATAT CTTGCAATGT
1101 GGATGTACTA AATAGGCTTT TATTGAGTTC AGACCCATTG TTATCGTGTA
1151 CGCGATCAAG ATTTATGAAG AATAAGAAGC CATTCACAAA AGAAGCATTG
1201 GAATTTCTTA TACCAGAAAG TGATGATTCT GAAGATACAG GAGCACAATA
1251 TACGACAACG ATAGATGAAT GTGATATATC TGATGATAAT GAATTTGACT
1301 AATATAGTAT TTTGATATAT AAAATCTATT GTTTTAATAC CCTCTACCCA
1351 GTAACATTTT ACTTTATAAT TTATTCTTAG ATAATATTAA AGGTACGCTA
1401 AAATTATCAA TTTTAACGTA CCTGAGGGCA GTATAGTAAT CGCCATTGAG
1451 CAAAAATAGA TAAAATATGA CCAATTTATC AGTTTAGGTA GTTAGACCAT
1501 AAGAAATAGT TTTGGCCAGC TTTTTGTTAC AAATACCCCA CCGTGCGACG
1551 GCGCGCCTCA CNNNNNNNNN NNNNNNNNNN NNNNNNNNNN NNNNNNNNNN
1601 NNNNNNNNNN NNNNNNNNNN NNNNNNNNNN NNNNNNNNNN NNNNNNNNNN
1651 NNNNNNNNNN NGTGTGCATA CATAATTGTG AATCCTACAA TGTTCAGATA
1701 ATATGATCTT AATGTTGTTC ATATAATAAT GTGCTTTTGG CTGTATACAT
1751 GTTACTGAGA AAAACCAGTC ACATATTAAT GAATCTAAAT CTTGACCACA
1801 ATTTAATGAT GCTTATAGTT GTCACAATAT TATGAATTTT TTTGACGGTC
```

1851 ATATATTTTT GAAGCCTGAA AGGGCCAATA TATTAATGAA CTCGACTGTA
1901 CAATACGATA TCAACAGTAT AGGCCAGTCA CGGACCTACA AATTGGTGCT
1951 GGTTGCTAAC CATAAACTCT TAGATAAAAT TACTCAGATA TTATTGTAAA
2001 CTTTATGGCC ACTCCATATG TAAGTCTTAA TATTTTTTTA AAACATTTA

The transcript and protein sequences of *ApsTransib_1266* were predicted by AUGUSTUS.

QOUH01081266.1:1-2049 AUGUSTUS gene 1 1302 0.81 + . ID=g1

QOUH01081266.1:1-2049 AUGUSTUS transcript 1 1302 0.52 + . ID=g1.t1;Parent=g1

QOUH01081266.1:1-2049 AUGUSTUS CDS 1 1299 0.52 + 0 ID=g1.t1.cds;Parent=g1.t1

QOUH01081266.1:1-2049 AUGUSTUS stop_codon 1300 1302 . + 0 Parent=g1.t1

coding sequence = [tacctcgaagaagttttgaaaggactttcaagtgaagagaaggaaaatttaaaacttatatcaaaatggggatgtgacg
gatctcaacagacacggtatatgcaatccttacaaaatacgaacgaaagctatgacgatgcaaatatattccaaagctcgtttttgcctttgagatta
cttgttagtcataacagccaaagtaaagtcatttggcaaaaccccgtcccgtcgagtccaagattttgtagacctattcgaatcagatttgtgcatga
aactaacgacatcacaagaaatgaaattagttatatagaaagtcaaattgaaaaattaaacctatctgaaatatttttgaatagtgaagtgataaaag
taaagcatctgtttttatttactatggtggatggaaaagtctgcaatgcggcaactaataccctatcaacaatgcgttgctatatttgcaaagcaaca
tcaaaagatttcaataaattagaaacaagaagagaagaagaccctgcgactttgaagtttggtctttcaatattacatgcgagaataagattatttga
atcgattctacatttggcgtacaaattacctacacaaaaatggcaatcaaggtcttctgctgacaaaaaaattgtaaatgagaataaaatacgtatac
agcaacaatttaaagaagaaatgtcactactagtagatgtaccaaaagctggttttggaaatactaacacaggcaatgtatcaagacgatttttctgt
gatcctgagacagcatctagaattaccggagtagatttagatttgataaaaaggcttagaactttgttagaggtcatttcaagcggtcacagaataga
taccgacaaattatcaacattttgtaaggaaacttcagaaatttatgtgagactttacggatggtatcccatgacgcctacactccacaaattgttgg
ttcatggacctacaataataaaacatgccataatccctataggccagctctctgaagaagcggcagaagcgagaaacaaacactttcggcaataccgt
actgattttgctagaaagttttcaaaaatatcttgcaatgtggatgtactaaataggcttttattgagttcagacccattgttatcgtgtacgcgatc
aagatttatgaagaataagaagccattcacaaaagaagcattggaatttcttataccagaaagtgatgattctgaagatacaggagcacaatatacga
caacgatagatgaatgtgatatatctgatgataatgaatttgactaa]

protein sequence = [YLEEVLKGLSSEEKENLKLISKWGCDGSQQTRYMQSLQNTNESYDDANIFQSSFLPLRLLVSHNSQSKVIWQNPVPSS
PRFCRPIRIRFVHETNDITRNEISYIESQIEKLNLSEIFLNSEVIKVKHLFLFTMVDGKVCNAATNTLSTMRCYICKATSKDFNKLETRREEDPATLK
FGLSILHARIRLFESILHLAYKLPTQKWQSRSSADKKIVNENKIRIQQQFKEEMSLLVDVPKAGFGNTNTGNVSRRFFCDPETASRITGVDLDLIKRL
RTLLEVISSGHRIDTDKLSTFCKETSEIYVRLYGWYPMTPTLHKLLVHGPTIIKHAIIPIGQLSEEAAEARNKHFRQYRTDFARKFSKISCNVDVLNR
LLLSSDPLLSCTRSRFMKNKKPFTKEALEFLIPESDDSEDTGAQYTTTIDECDISDDNEFD]

No potential TIRs could be reliably identified surrounding the ApsTransib_1266.

Erysiphe pulchra

16. EpuTransib_3173

>PEDP01003173.1:1-3970 Erysiphe pulchra isolate Cflorida contig_3173, whole genome shotgun sequence
1 CAGAAGGAAC GATGTTAGCA TGTAGATGAA ATAACATGAC GAAGTTTTAT
51 GGTCACAGGT TATAATCAGA GTGTGGTATT TGACCTCAAA ACCTGGCCAA
101 AATTATTTTT TGGTCTTAAC GGATCCCAAT TGCTACTAAA AGACATATTA
151 TTGATAAAAA GGGTAAAAAT AAAGACTTAT GGTGATTTAA AACTATTTAT
201 TCATCATCAC TATCATCATA TTCAGATTGT ACATTTTTTA CATTAGCAAG

251 ATCACATTCT GACAAAAGTT TTAGGACATC GGACGATAAC GGCATCTTTT
301 TTTTATGTAA TGAGGACCGC ATACCAGTGA TTAAAGGGTC TGAAGTCAAT
351 AAAAGTCTGT TAAGAACATC TTGGTTGCAG CTTACTCGTG AAAACTTGCG
401 TGAGTAATTG AGCCTGTATG ACCGAATGTG TTTGTTTCTC GCTTCTGCTG
451 CTTCTTCAGA TAACTGCCCA ATGGGGAGTA TTGCGTTCTT TATAACTTCA
501 GCTCCATGCC TTAAAATTTT ATGTAATGTA GGAGACATGG GGTAAAAATC
551 ATAAAGTGCC ACGTAGAGCT CAGCAGTCTC TTGGCAATAG TTCTCAAAAG
601 CTTCGGTGTT GATTGTGAGT CCACAGCACA AGGTGTCTAG AATTATTTTC
651 ATTCTATAAA TTAGATCTTT GTCAATGCCT GTAATAGCTG CAGAGATTTC
701 GTAGTTCTCA AAAAATCGTC TGCTGGTATT TCCATCGTTA GTGTTTCCGA
751 AATTAGCTTT AGGTGCATCT ACTAGGAGCC CGAGTTGACT TCTGAACTGT
801 TCCTGTATTT CCTTTTTTCT TTCTTTCACC ATTTCGGACT CTGCTGCAGT
851 AAGGCGGACG TTCCATTTTT TTACTGGTAG CTTATACGCT ACATGTAAAA
901 TACTCTCTAA AAATCGTATA CGGGCATGGA GTAAAGAAAG TCCAAAGCTC
951 AAATTTTCTT GTTTGCTTGG TTTTGTCTTT GACAATTTGT TGAACTCCGA
1001 TGAAGTTGCT CCGCAGATGT AGCACCTCAA TGTGGATTTT GTATGTGTGG
1051 CGGCATTGCA AACTTTACCA TCTACCATTG TAAACAACAT TTTATGCTTG
1101 ACTTTTAAGT CTCCAATTTC GGTGCTGGAA AGACTTTCAA TCTGGGATTT
1151 TATATAGTCT ATTTCTTCAT TTGTTATGTC AGTTGTCTCC TTCACAAATC
1201 GAAGTCTTAT TGGCCGACAG TAGCGGGGAG ATGACGGGGT AGGATTTTGC
1251 CACAGAATTC TTTGTTCGGG TCCGTATAAT AATTGCAATG GTACCATTGA
1301 ACTTTGGAAA ATATTTGCAT CATTGTCCGC TTCATTTTCA AACTTCATCT
1351 TGTATTGCAT TTGCTGTGAT CCGTCACATC CCCATTTACT AATTAATGTC
1401 AGGGATTGCT TCTCATCTTC ATTTAAAGAC TTTACAACCT CTTCAAGGTC
1451 CATAAGAAGT CGCCTGGCTG TGTGGTCCAT CAATTTCTGA ACTTGTATTT
1501 CAGCGCAAGT CTCAGTCACT CTGTGAATTT CAGGATAACA CTCTTTCTTC
1551 GCTTTCTGTA CCAGAGAATA ACACGGAAAG AAACGTTTGT TTGTGTTTCT
1601 TATTATTTCA TATTGGGCTT TAGTAAGACC TGCTTCTATG AACATGGATA
1651 GTGCCTGTAA AGGTGAGAGC TGACTTGGTT GTTCTTGTTC ATTTTGGCTT
1701 CTTTTCATAG CCGTTACATA TTTTGTTGCT CTTCGAGGAC TTTTTGTAAG
1751 ATCTTTTACG ACCTTTGAAG CATCAGTTTT GCCTGAATCA CGAAGTTTCA
1801 TCTGTGTTGC ATAGGTCAAT TCATCAACAT CTGCTGATCG GAGATCCTGT
1851 GTTTTTCGCC TCTTGGTTCT TTCACTTGAG TCTGAAAAAG ACTTGATGGG
1901 GCGACCAGGG CTATTTCCTG TAAAATATTA ATAAATAATT AATTAATGGT
1951 AATTACACGA AAGCAATAAT AATTATAGTA ATGAGAAATC CTTAGTAGGA
2001 GCATCGTGCT TTCCGAATTC TAAGAAATCC AATATAAAGT GCAATTTAAC
2051 ATACCTGCGG CTGGCAAAAA AATTGTGCTA TTGAGCCAAT TAGCATTTTT
2101 TTCCTCGAAT CTTGCTTTTG TATTATTAGC AGCTGTCCAT TTTTGCTTGA
2151 ATTCATGCTT TACAATTGAA AGTCTATGTC TTACATCCTT TAATATGATG
2201 TCATTGTCAG CATACTGCTT CAATATTTCA GACTCCATAA ATCTAAGTTT
2251 TCCATCAAAA GTTGGTTCGT TCGCTCTTTT TATGATACCA TACAACTCAT
2301 TTCGAGTGAT AGACCTCAAC CCAGAAGGAC CTGTAATAAT TAAAAAAAAT
2351 TAGGATTTTC AAAAAAAATT AAAATTTGGG GTGCCACCCA AAAAAGCGAA
2401 TTACAAAAAA GGGGGTGGTT TCGGGTGGCC AATTCTTTGA TGGCTGCTTC

```
2451 ATGGAAGAAC CACATTATGC CATGGAACAA GTTTCTCAAC TCAGAAATGC
2501 TCAGAAATAT AACAAAAACC TTTTTATATG TTGAAAAGTA GTATTTTTTT
2551 CACATATATG AGAGGCTTCT GTAATATTGT CGAAGTAGAC TAAATTTGGC
2601 CAAAAATATA TCTAATATAT AATAGAATAT GAAGAACATA CCTGGTTCTG
2651 CAGTATCCAT CACAAAAACA GTTCACTAAT ATCTTAGATA CGAAAAAATT
2701 ACACTTTGAA AGATAGTAAA ATTTAAGCAC ACTTATTAAT CACACAACAC
2751 TCTGAGGCGA AGGGTAACTT TCGATTGATC GTGCTGGGGA ACGATTAGTC
2801 GCAGTGACTT GCGGCTTTCA CCCGCTCATG CGCCGTAGTT TCCTGACTTA
2851 AGTTAGAGCG CTAGATAGAC AGGGGGGTTT CAGAGTACCG ACACGGCTGT
2901 CATCCCTGCA CGTTTTTGCA CGCACTTTCA CTTAAGTGTG GCTAAATCTC
2951 ATCAATTTTT TGTTGGTATT GAAATACAGT CTATTAGGCT CATTCTAAAA
3001 CAGAAGCTAA TATGACTTAA TACTAGAGCT CTAGCTAAAT AAATAAAGTT
3051 GTTGTCAATA TTTGAGTGAT AAGTTGAAAT TCATTTCTGC TTTAAACAAA
3101 TTATGCCTCA ATATTGCACA GTACCTAGCT GCAACTCAAA TTCAAGTCAG
3151 ACATTCCACT ACAGCTTCTC AACTTCAGAC GAGGATCGAC TCAAAAGCTG
3201 GATCGATATT ATTGATATTT AAAGTTCTTC TGGTCAATCT GGCTCGAAGA
3251 GAAAGTACAT TTGTGAGGTG AGTAATACTC ACGCAGTCAT GCTTGAGGGC
3301 TGTTCATATG AACACCAGAT AAAGAAGCAT TTTTTTAGAA ACATTTTTCT
3351 GACAGTCAAT TTAAATCTGG TGATTCTCAG AAAAGGAAAA CACTAAGACG
3401 AGATGCTGTA CCCGATTTAC TAATAAAACA AAAGTCCACT TTATGTGATG
3451 CTGAACAGGA AGATTAAATT AAAACAACAA ATAAGTTCTT TGAGGGAAGA
3501 AAATGAGAAT CTGAAGGTTG CAATAAAGAA TTGGAAGACA AGATGTGAAC
3551 TTGGCAAGTG TTAAAGAAAT TGGAAGATTC ACAAGGCTCA AATGCTACAA
3601 ATAAAATGAA CTTTGTTTAA AAAAGTCAAT ATTTTCAGTC GTTCTTTCAG
3651 CTGAAAATTT CTTTAAATCT CAAAGAGAAC TTATTGTAGA TAATTCACTG
3701 AAAATCAAAG AACTTACAGT ACTGTTTTTG GAAACCCTAA CTAATAATGA
3751 TGACTGCAAG AAAATTCCTG ATTGCCATAA TATGCTTCAA AAAATTATTA
3801 ATTCCTTTTA TAAGTATCAT ATTTATTTTC TTAATGGAGA AATGACATTA
3851 AATATTTTCA AAGGATCTGT AAAGTGTGGT AGTCGCTCAG TAGGCATGAG
3901 AAATGCTATT AAAAATTTGT GATAAAATAT TTAGTTTTTC GTAAAAATAT
3951 CGTTCCTTCT GTTCCTTCTG
```

The transcript and protein sequences of *EpuTransib_3173* were predicted by AUGUSTUS.

```
PEDP01003173.1:1-3970 AUGUSTUS gene 197 2660 . - . ID=g1
PEDP01003173.1:1-3970 AUGUSTUS transcript 197 2330 0.45 - . ID=g1.t1;Parent=g1
PEDP01003173.1:1-3970 AUGUSTUS stop_codon 197 199 . - 0 Parent=g1.t1
PEDP01003173.1:1-3970 AUGUSTUS intron 1918 2054 0.98 - . Parent=g1.t1
PEDP01003173.1:1-3970 AUGUSTUS intron 2331 3970 0.46 - . Parent=g1.t1
PEDP01003173.1:1-3970 AUGUSTUS CDS 197 1917 0.99 - 2 ID=g1.t1.cds;Parent=g1.t1
PEDP01003173.1:1-3970 AUGUSTUS CDS 2055 2330 0.91 - 2 ID=g1.t1.cds;Parent=g1.t1
coding sequence = [gtccttctgggttgaggtctatcactcgaaatgagttgtatggtatcataaaaagagcgaacgaaccaacttttgatgg
aaaacttagatttatggagtctgaaatattgaagcagtatgctgacaatgacatcatattaaaggatgtaagacatagactttcaattgtaaagcatg
aattcaagcaaaaatggacagctgctaataatacaaaagcaagattcgaggaaaaaaatgctaattggctcaatagcacaatttttttgccagccgca
ggaaatagccctggtcgccccatcaagtctttttcagactcaagtgaaagaaccaagaggcgaaaaacacaggatctccgatcagcagatgttgatga
```

attgacctatgcaacacagatgaaacttcgtgattcaggcaaaactgatgcttcaaaggtcgtaaaagatcttacaaaaagtcctcgaagagcaacaa
aatatgtaacggctatgaaaagaagccaaaatgaacaagaacaaccaagtcagctctcacctttacaggcactatccatgttcatagaagcaggtctt
actaaagcccaatatgaaataataagaaacacaaacaaacgtttctttccgtgttattctctggtacagaaagcgaagaaagagtgttatcctgaaat
tcacagagtgactgagacttgcgctgaaatacaagttcagaaattgatggaccacacagccaggcgacttcttatggaccttgaagaggttgtaaagt
ctttaaatgaagatgagaagcaatccctgacattaattagtaaatggggatgtgacggatcacagcaaatgcaatacaagatgaagtttgaaaatgaa
gcggacaatgatgcaaatatttccaaagttcaatggtaccattgcaattattatacggacccgaacaaagaattctgtggcaaaatcctaccccgtc
atctccccgctactgtcggccaataagacttcgatttgtgaaggagacaactgacataacaaatgaagaaatagactatataaaatcccagattgaaa
gtctttccagcaccgaaattggagacttaaaagtcaagcataaaatgttgtttacaatggtagatggtaaagtttgcaatgccgccacacatacaaaa
tccacattgaggtgctacatctgcggagcaacttcatcggagttcaacaaattgtcaaagacaaaaccaagcaaacaagaaaatttgagctttggact
ttctttactccatgcccgtatacgattttagagagtattttacatgtagcgtataagctaccagtaaaaaaatggaacgtccgccttactgcagcag
agtccgaaatggtgaaagaaagaaaaaaggaaatacaggaacagttcagaagtcaactcgggctcctagtagatgcacctaaagctaatttcggaaac
actaacgatggaaataccagcagacgatttttttgagaactacgaaatctctgcagctattacaggcattgacaaagatctaatttatagaatgaaaat
aattctagacaccttgtgctgtggactcacaatcaacaccgaagcttttgagaactattgccaagagactgctgagctctacgtggcactttatgatt
tttaccccatgtctcctacattacataaaattttaaggcatggagctgaagttataaagaacgcaatactccccattgggcagttatctgaagaagca
gcagaagcgagaaacaaacacattcggtcatacaggctcaattactcacgcaagttttcacgagtaagctgcaaccaagatgttcttaacagactttt
attgacttcagaccctttaatcactggtatgcggtcctcattacataaaaaaaagatgccgttatcgtccgatgtcctaaaacttttgtcagaatgtg
atcttgctaatgtaaaaaatgtacaatctgaatatgatgatagtgatgatgaataa]
protein sequence = [PSGLRSITRNELYGIIKRANEPTFDGKLRFMESEILKQYADNDIILKDVRHRLSIVKHEFKQKWTAANNTKARFEEKN
ANWLNSTIFLPAAGNSPGRPIKSFSDSSERTKRRKTQDLRSADVDELTYATQMKLRDSGKTDASKVVKDLTKSPRRATKYVTAMKRSQNEQEQPSQLS
PLQALSMFIEAGLTKAQYEIIRNTNKRFFPCYSLVQKAKKECYPEIHRVTETCAEIQVQKLMDHTARRLLMDLEEVVKSLNEDEKQSLTLISKWGCDG
SQQMQYKMKFENEADNDANIFQSSMVPLQLLYGPEQRILWQNPTPSSPRYCRPIRLRFVKETTDITNEEIDYIKSQIESLSSTEIGDLKVKHKMLFTM
VDGKVCNAATHTKSTLRCYICGATSSEFNKLSKTKPSKQENLSFGLSLLHARIRFLESILHVAYKLPVKKWNVRLTAAESEMVKERKKEIQEQFRSQL
GLLVDAPKANFGNTNDGNTSRRFFENYEISAAITGIDKDLIYRMKIILDTLCCGLTINTEAFENYCQETAELYVALYDFYPMSPTLHKILRHGAEVIK
NAILPIGQLSEEAAEARNKHIRSYRLNYSRKFSRVSCNQDVLNRLLLTSDPLITGMRSSLHKKKMPLSSDVLKLLSECDLANVKNVQSEYDDSDDE]

No potential TIRs could be reliably identified surrounding the EpuTransib_3173.

Bacteria

Candidatus Thioglobus

uncultured SUP05 cluster bacterium isolate

17. CthTransib_0091

>FXLE01000091.1:1-4563 uncultured SUP05 cluster bacterium isolate SPONL genome assembly, contig: NODE_91_length_4563_cov_5.43012_ID_10321, whole genome shotgun sequence

```
   1 TGTAGCTATA ACTATATTGC CCCTCTTGGA CTTCAATTTT TAAAGTTAAC
  51 GAAGCACTAG CTGTATCTTA GAGCCTGCAT GCAACTCCAT GGTGTTTACA
 101 ACTGCAAACC CCTGGTTGCA CTGCGTAGAG CAATTATTCT GGGTTCCATA
 151 ATAACTATGA TGTCTGCACA CTCACAATTA TAAGGGGATG TGTCAACATT
 201 ATAAGGGGGT TCTGCAGACC CCCCTAACGC TTCCGTTCAT CCTCGCCCTG
 251 TCAACTGTTC AAAGGGCCCA GTGAGGGAAG TTCAGTATCC TTCCCCATGC
 301 ATGAGCATGG CCGGGGTGAG TGGGAGTCAT GTTGATTTGT GTTGATTGAT
 351 GCACAATCAC TGTGTGCATT AATACTGACT GTACAGTTAT ACTCAGCTCA
                  >>>predicted 3'TIR
 401 GTGTCTACCA TACACTGTAA AAACTGTGGT TTGCACTACA TACCGCGATG
 451 GTATGACCTT CATACCAAAC AGTTTCATAC CATTGGTATG AAACAATCTA
 501 GTATGAAATA CATACCCTTG TGGTATGTAT TTCAGGTATG AAATTCGGAA
 551 ACAAAAGCAC CAGCACTGAG TTAGCCCTGA ATTTGGTTAC AAGCATTTAG
 601 TCATCTGCAG GAGTGGTCTA GAATAGCACA GGACTGTGGA CCAAGCTGCA
 651 AAGGGAGGAT TGCCCAGAGG TCATGCATGC CTAGAGTTCA TTGAAAGGTC
 701 AATCATTTGA ATTTAGCTTT GCAGCTGCTT TTAGAAGTTT GTCAGAAAGT
 751 ATTCTCCTCT TCATTTCATG AAGTCTTGAC CATTGGGTAA GTGAAATAAC
 801 TCTATAGGTT TGTTTATTTT GCCTGCTAGC TTTATGTTTG TGTGAAATGT
 851 AGAGGCAGTC AGCCTGTTTA ATAGTTTTCA TCCTTACAGG TTGGTAGTGT
 901 GAAGTTCTCT CTGCACTTCA GTTGACAATC ATTCAGCTAA GGTATAATTA
 951 AGCCATGCAT GCACTAGCTT GTGTTGATAC TGTCATTCTA TACAGATTGA
1001 GTTGGACTAA AAAGTAAAAC AAAAGTAAAA AGGTATGTGT ACTTGTTTTG
1051 TGTAGCTATA GCTAGCTATC ATTTTGCAGA TGTCACAAAG GAGCAGCATA
1101 TCAAAACCTT CCAATAGCAA ATGTCAAATC AAACCCATTA CATTTTTGCA
1151 TGGGCAATTG AAATTTTTTC TGGGCAATGA CATCATCAAA TCGGTAGGTG
1201 TGGCCTAGTG GAGAAAGCTA TGGAGGCTGA AAATCTTCCC ATCGCTCTTA
1251 GAAGAGGGAA AAGGAACCTA CGTGAAGTGA ACTACCGGGC AGCAGAGAGC
1301 CTGAAGCTGC CCAGAGCTTC TTCAAGCCTC GTGAAGGACA AACTTTATCC
1351 AGTGACTGTA CTAGAACAGG ATTCTGATAG AGTGAAGGTA CATTATGTTG
1401 GTTACTCAAG TGAGCATGAC GAATGGAAGT ATGGGGGTGA ATTAGAAGAC
1451 TTAGAGCCTG AAGAAGGTCC TGCTACATGT TCAGTGTATC AGCCTTACTC
1501 ACTCTACAGC AATTTGCGCA TTAAGGTGAA ACAGGCACTT ACTTGTGGAC
```

1551 GTAAGAGCTC ACCTCTAGTC AAGATTGTCA TGGCCTTTGA CTTGGTTCAG
1601 TTCAGTGGTG GATTGCAAAC TGTTGGTGTA CCATCAAAGA AAGTTCAAGG
1651 TGTTCAACAC TACCAAATAA AGAACTACAA AGATTTGAAC CCCTGGCTTG
1701 GAAGCTACTG GCACTTTCGA GGACTTAACG CCAATGGTGA CTATGGATTC
1751 GTGGAATTAG AAACAATTGA CTTCTGTATA CGAAGATCAA GATCACTAGT
1801 GGAATACCTT CCACCAAGTG ACCACCAGCT AACACCTTGC AAGTTGTCAA
1851 CAGACACTGG TTTTAGTCTT TCTTTTTGTT TTGTGTGTAA CTATGGAACA
1901 GCTGCTACAT TTGGAAAAGA TAAAAATATT TTTGACTAGA AAAGAAATAT
1951 AATATGGCGC CGCGAATTTA TAAGTTAAGG GTACATGAAC GCTTGTTGTG
2001 ACCAGTGAGT TTACAATTGG TACATTTCTG TTCTTTCTTT TTTCTCTGGA
2051 AGCCACCAAT TTCTAGCCCC TCAATGCGAT TTCGTTTTTC AAGCAGTTGC
2101 TTAAGAGCTT CATGTTCTCG ATGATTAGAA GATCGCTGGA AGTGAACAGT
2151 AGTAACATCG TTCAGTTTTT CGAGACCTTG CTGAATGAAC ATCACAATGT
2201 TTCCATACAA GCGTAGGAAT TCACCCACAT GCATTGCAAA GGCGTGCATG
2251 TAAGGGGTCA CATCTTTAGA CTGGTAGACA CTAGTAAAAA GTTTCACCCA
2301 TGTTTTTGCA CGGAGGTCAA AGGCCGAAGC ATCACATTCA CTCTTCCCTA
2351 GCACGTTTAC CAGTTTAAAG AACTCACTCC ATAACTCCTG AAGTTGCGTC
2401 TTGGTTTGCA GAGCAGGGAA CAAAGTTGGG ATATTCATTT TCTTGAATAG
2451 CTTTGTCTTC TCAGGCCCAG TCAAATCACG CCAGGCAAGC TTTTTCGATT
2501 CTTTGTCAAC GTACCATTGA AATCTAATTT TGCATGTATC GTTGAGAAAC
2551 TGAACATAAG CGTCAACATT CTGGCCAGTA TGCTCTGACG TAGCCTTGTC
2601 CATGCCATCC AAAACTCTTA TATCACGGAT AAGGAGATTA ATAAGAACAT
2651 CCGATATTCG GAGAAAGAGA TGAAGTGAGT CAATTACAAC ACGTTGAAGT
2701 GGAATAAATG GAAACATTGG TACTCTGCAA CAGTTAAATC GGTTCTTACT
2751 AGATTTAGCT AGCTTCGATT TGTCAGTGAT TTCCTTCACC GTCCTCCCCC
2801 CCTTTTCAGG ATCAACAAGC GACCAAGTTA AACTCATATC CCACCGTTTC
2851 CTTTTCGGAC ATTTGCACCA TATACATGCA TATTCAGACG TGGCAGACTC
2901 CAAGCCACAT ATCAAAGCTA AAAACTTCCA GTCGCCTCCG AGGAAGAACT
2951 GAATTCTATA CACCCTTTCT TCAACTGTTA CCACCTCTAA ATCTTTAGCT
3001 TCACTGATGA TATCTTCTAG TGCAGAAGCA AGGTCCTCAT ACTTCTCAGA
3051 TACCCTCAAA ATCGCAATGC TATGATTTCC TGACACCGAT GTTGCTTGTT
3101 GACCCTCTTC TAGTATAGTG AACGCAACGT TCACAACACC CAACCCTCTG
3151 GCTATTTGAG TACCATCTCC GGTAAGTTTT ACTCTAATAA TACTGGGAAT
3201 GGGTCTATTC TCAGCAGTGT ATTGATGAGC AAGCTGGGTC AAATGGACAG
3251 TGAGACGTTT GCGTAAGCTT TGTTGTACTC CTGTTCGATT AGGGGTGCTA
3301 CGTATTTCAA ATTCAGAATT CAAGGCAGAT TTCAATCTCT TAACCTGAGA
3351 CGAGTTAGGT AGATCTGATA GCATGCTTAA CTCATGGAAT GCTTCATTAG
3401 ATACACAAAA CTTATCTTTG ACGTAGAGAG CAGAATGGAG CTTGTCATTC
3451 GAGCCAGGAG ATGATTCCTT TCTATGAAAT GTACCACAGC TAACATCAAC
3501 AACTTCCCGA TTTCCTGTGT CGATGTTCTC AATCTCAACC ATGCATGGTT
3551 TAAACCCCTC TTCTTCGCAA AAACTCAGTG CACCTTGAAT GCCAGCAGCA
3601 ACGTTCCTCT TCTTGTTGTA CTGCTGCTGC CTGCTGTATT CTGACCATGG
3651 CTTCGATGAC TTTCTGCTAT TCTCCGAAAG ACCAGTCTTA AGCCGTGCAA
3701 TGACTTTCGC TTGCTGTTTA GTTGATTTCC GTAGCTTTCC CACCTCAGCT

3751 TCAAACTTTC TCCTCTTGGC GCTCTCCTCC TCTAGCTTGG TTTCAGCATT
3801 ACGCTTCATC TTTAAGAGTT GCCGGTTTAC TTCATGTTCT TCAATTTCAA
3851 GGTTCCACAC AGAAGCAATA CCCTCCTTCC ATTGTGCCAA TTTCTGCTGG
3901 CGTTTCGTAC CCCCAATCTT CGAATAGAGC TTCCCCATTC TCCATAGTTT
3951 TCCCTTCACA GCTGCTCTAA TCTTGTCCGT AATCTCAAGG GGAACTTCGT
4001 AATGCTTAAA AGCTTTGGTG ATTATCCTTT CATACAGCAA TTGAACAGGG
4051 GTGAACTTGT CAGAGGACAC ACATGCCTCA TCGTCCTGAG ACAAGTCATA
4101 ATTGGGAGTT CCATTTTTCC CTGGGTGAGA AATGCTCCAG AGATAGTAGC
4151 TGTGAAATAT GCATACACAT TATTCAATCT ATAGGCCAAG GAAGTCAAAA
4201 GAAAATCCAC TTACTTGTTT ATCTCAATGA TTTGAGCCAT CCATCATACT
4251 TCTCCAAAGT GCGAGATGAA GCGCTATGCC TACAGCGAAT CACTTGGTAT
4301 CAAACTGCTG CTAAACCCCT GCTCTATTTG ATGCCACCCT TACTGGAAGG
4351 GGTTCTCCCT TCAGAGTGAT GATGTAAACT GTTTTTATGA ACAAGTTGCT
4401 GAGTAAGCAA TTAGTCAATA CAAAAAGGAT AGGGGGTGGA GTGAGATTGT
4451 CCTGCTAAGA AGCTAAATAG CGGCCACTTC CGGTATGATG CTGGTCGCTT
4501 GAGGGATATT ATCTTGAAGA GGGAGCCCTG AAGAAGAAGA ATCTGGCCAT
4551 ACTGAGTTTA GCC

The transcript and protein sequences of *CchTransib_0091* were predicted by AUGUSTUS.

FXLE01000091.1:1-4563 AUGUSTUS gene 1968 3966 . - . ID=g2

FXLE01000091.1:1-4563 AUGUSTUS transcript 1968 3966 0.76 - . ID=g2.t1;Parent=g2

FXLE01000091.1:1-4563 AUGUSTUS stop_codon 1968 1970 . - 0 Parent=g2.t1

FXLE01000091.1:1-4563 AUGUSTUS intron 3967 4563 0.76 - . Parent=g2.t1

FXLE01000091.1:1-4563 AUGUSTUS CDS 1971 3966 0.82 - 1 ID=g2.t1.cds;Parent=g2.t1

coding sequence = [agcagctgtgaagggaaaactatggagaatggggaagctctattcgaagattgggggtacgaaacgccagcagaaattg
gcacaatggaaggagggtattgcttctgtgtggaaccttgaaattgaagaacatgaagtaaaccggcaactcttaaagatgaagcgtaatgctgaaac
caagctagaggaggagagcgccaagaggagaaagtttgaagctgaggtgggaaagctacggaaatcaactaaacagcaagcgaaagtcattgcacggc
ttaagactggtctttcggagaatagcagaaagtcatcgaagccatggtcagaatacagcaggcagcagcagtacaacaagaagaggaacgttgctgct
ggcattcaaggtgcactgagtttttgcgaagaagaggggtttaaaccatgcatggttgagattgagaacatcgacacaggaaatcgggaagttgttga
tgttagctgtggtacatttcatagaaaggaatcatctcctggctcgaatgacaagctccattctgctctctacgtcaaagataagttttgtgtatcta
atgaagcattccatgagttaagcatgctatcagatctacctaactcgtctcaggttaagagattgaaatctgccttgaattctgaatttgaaatacgt
agcacccctaatcgaacaggagtacaacaaagcttacgcaaacgtctcactgtccatttgacccagcttgctcatcaatacactgctgagaatagacc
cattcccagtattattagagtaaaacttaccggagatggtactcaaatagccagagggttgggtgttgtgaacgttgcgttcactatactagaagagg
gtcaacaagcaacatcggtgtcaggaaatcatagcattgcgattttgagggtatctgagaagtatgaggaccttgcttctgcactagaagatatcatc
agtgaagctaaagatttagaggtggtaacagttgaagaaagggtgtatagaattcagttcttcctcggaggcgactggaagtttttagctttgatatg
tggcttggagtctgccacgtctgaatatgcatgtatatggtgcaaatgtccgaaaaggaaacggtgggatatgagtttaacttggtcgcttgttgatc
ctgaaaagggggggaggacggtgaaggaaatcactgacaaatcgaagctagctaaatctagtaagaaccgatttaactgttgcagagtaccaatgttt
ccatttattccacttcaacgtgttgtaattgactcacttcatctctttctccgaatatcggatgttcttattaatctccttatccgtgatataagagt
tttggatggcatggacaaggctacgtcagagcatactggccagaatgttgacgcttatgttcagtttctcaacgatacatgcaaaattagatttcaat
ggtacgttgacaaagaatcgaaaaagcttgcctggcgtgatttgactgggcctgagaagacaaagctattcaagaaaatgaatatcccaactttgttc
cctgctctgcaaaccaagacgcaacttcaggagttatggagtgagttctttaaactggtaaacgtgctagggaagagtgaatgtgatgcttcggcctt
tgacctccgtgcaaaaacatgggtgaaactttttactagtgtctaccagtctaaagatgtgaccccttacatgcacgcctttgcaatgcatgtgggtg
aattcctacgcttgtatggaaacattgtgatgttcattcagcaaggtctcgaaaaactgaacgatgttactactgttcacttccagcgatcttctaat

catcgagaacatgaagctcttaagcaactgcttgaaaaacgaaatcgcattgaggggctagaaattggtggcttccagagaaaaaagaaagaacagaa

atgtaccaattgtaaactcactggtcacaacaagcgttcatgtacccttaacttataa]

protein sequence = [AAVKGKLWRMGKLYSKIGGTKRQQKLAQWKEGIASVWNLEIEEHEVNRQLLKMKRNAETKLEEESAKRRKFEAEVGKL

RKSTKQQAKVIARLKTGLSENSRKSSKPWSEYSRQQQYNKKRNVAAGIQGALSFCEEEGFKPCMVEIENIDTGNREVVDVSCGTFHRKESSPGSNDKL

HSALYVKDKFCVSNEAFHELSMLSDLPNSSQVKRLKSALNSEFEIRSTPNRTGVQQSLRKRLTVHLTQLAHQYTAENRPIPSIIRVKLTGDGTQIARG

LGVVNVAFTILEEGQQATSVSGNHSIAILRVSEKYEDLASALEDIISEAKDLEVVTVEERVYRIQFFLGGDWKFLALICGLESATSEYACIWCKCPKR

KRWDMSLTWSLVDPEKGGRTVKEITDKSKLAKSSKNRFNCCRVPMFPFIPLQRVVIDSLHLFLRISDVLINLLIRDIRVLDGMDKATSEHTGQNVDAY

VQFLNDTCKIRFQWYVDKESKKLAWRDLTGPEKTKLFKKMNIPTLFPALQTKTQLQELWSEFFKLVNVLGKSECDASAFDLRAKTWVKLFTSVYQSKD

VTPYMHAFAMHVGEFLRLYGNIVMFIQQGLEKLNDVTTVHFQRSSNHREHEALKQLLEKRNRIEGLEIGGFQRKKKEQKCTNCKLTGHNKRSCTLNL]

```
                                    *        20         *        40         *        60         *        80
FXLE01000091.1:313-512   : -GGGGTGAGTGGGAGTCATGTTGATTTGTGTTGATTGATGCACAATCACTGTGTGCATTAATACTGACTGTACAGTTATA :  391
FXLF01000102.1:2805-3004 : GATGTGATTCACCCATTAGTGTGGCAATGCATTGAACACCTGGAGTTAATTGGCCTCAAAGTTTTAGCAGTGGTGTGTGA : 2884
FXLE01000109.1:3136-3335 : CACAGGTGGGGAGAGGAGCACTCTTCACAGGTGGGGGGATGAGCACTATTGGAGTCTGCACTAAAAACCAGTGATTTCAG : 3215
                                                T         T               A T         A T     C       T

                                    *       100         *       120         *       140         *       160
FXLE01000091.1:313-512   : CTCAGCTCAGTGTCTACCATACACTGTAAAAACTGTGGTTTGCACTACATACCGCGATGGTATGACCTTCATACCAAACA :  471
FXLF01000102.1:2805-3004 : TGGTGCTACTCCCAATCGCAACACTGTAAAAACTGTGGTTTGCACTACATACCGCGATGGTATGACCTTCATACCAAACT : 2964
FXLE01000109.1:3136-3335 : TACTTTGTGTGACTGTACCCACACTGTAAACACTGTGGTTTGCACTACATACCGCGATGGTATGATCTTCATACCAAATA : 3295
                                               ACACTGTAAA ACTGTGGTTTGCACTACATACCGCGATGGTATGA CTTCATACCAAA

                                    *       180         *       200
FXLE01000091.1:313-512   : GTTTCATACCATTGGTATGAAACAATCTAGTATGAAATACA- :  512
FXLF01000102.1:2805-3004 : GTTTCATACCATTGGTATGAAACAATCTAGTATGAAATAC-- : 3004
FXLE01000109.1:3136-3335 : GTTTCATACCATTGGTATGAAACAAGATAGTATGAAATAC-- : 3335
                           GTTTCATACCATTGGTATGAAACAA  TAGTATGAAATAC
```

Figure 14 Alignment of 3'-flanking sequences of CthTransib_0091 shows its potential 3'TIR.

18. CthTransib_0066

>FXLX01000066.1:1-3240 uncultured SUP05 cluster bacterium isolate CRYPA genome assembly, contig: NODE_66_length_3240_cov_0.19332, whole genome shotgun sequence

```
  1 AAACAATTTA CTAATTTAAT TGTTAGATAA ACAAATTTTC AAAAAATATA
 51 ACTTTCCGTG AAATTGGGTG CGTTATGACG TCATAATGAC ATCATAATTA
101 GACGGATAAT GCTAAAAATG GACTATAAAC GACATATTTA ATGTACATTT
151 TAGCTATTTT ATATACCTCC ACAGTCTTCC ACCAACACCA AATTGAGCAG
201 TTGTTTTGAG TATAGAGGGT TAATGATTGT ATCAAAATAT TTTGTTGGTG
251 GAGGATTGTG GAGGTTGTTA AATATTAATT TATTTATAAC CTCAGCCAGA
301 CCCTTAAAAT CAATATTTTC ACTCTTCTGA TTGTATATAT GAGACCATTA
351 TATACACAAT CTGTACATTT CATGATTCAT TTCAAGTAAT GAAATTCATC
401 AGTAAATAAC TTATGTTTCT GTAAATAATT GTATTTATTT TAAATACAGT
451 TTGTGTACAT TGAAACCAAG AGAAAAACTC AGTGCATGCA ATCCGGCGTC
501 AATGCCATAA AAAGAACTGG GGACTATAAG TGCCATATAT GGCCCCATAT
551 TTAATGAATC ATTATCTTAG AAAATACTTG ATTAAAATTT GATGTGTTTT
601 ATTTTATAGA CGACATCATA CTTTTGATGT TAAAACTTGT TTCCAACAAA
651 ATCGATACGT ATTTGACGTC ATTGTGACGT CATGAGCGTT ATTTGTTTTC
701 TGTATTTCGT GGAATTTTGC CCGCGATATT TCGACGGTTT TCGAAGAAAT
751 TAAGCACCTC CGCAGTCGTG CACCAGAAAA ATATTTTACC AGATTATTAA
801 AATAAATAAA ACACAAATAC TGTTAAAGTA TTTTTTTGGT GCACGAGGTG
```

```
851 TTAAATTTCT TGAAAAACGA CGGGAAAATG TTCAAATATT ACAAGCGCTT
901 AGATGACGTT TTGATATTTG AGAAGCAATC TTTGCCGTAA AAAACGATGG
951 TATGTCGGCA CGTAACAGTT ATATACAAAT GTAAGATAAA AATAAAATAA
1001 ACACCACTTC ATCCAAAATA GTTTTTTTTC ATAAGCCCCC AATTATTGAT
1051 TATATATTTT CTTCCGGGAT ACTGAAATAG TTATTACTAC CGGGATACTG
1101 AGGTAGGTTT AATGGTCTAT CGATGCTACT TCTAAAATGA AGTAAAATAG
1151 TTTGATCCAA AAGGAGTAGA TATTTTTTGG GCACAATCCA ACAGATAGCT
1201 CTCACATTTT CAATTACAAG CTCCTTTTTC AAGGTGCTCT CCAAAGCAAA
1251 GGACAAATAC CCCGATTTTA AATATATAGA TATTTTAAAT TCCCAATTAC
1301 AATCATTTCA GTTAAAATTC ACAGAGAGAC TTGAAAACAG ACTTTCAAGT
1351 AAGACTATTA AAGTTTTGTT ATCAAATAGA AAATTGACAG GGAGGAAGCG
1401 AATTGCTTTT GATAGTAATG TGCATAAGAT CGCAATATTC TCAGATGAGT
1451 TAGAAAATGT AAAACAATTA AAATTTAGCT TACAAAAGTT TGAAAGCGAC
1501 AACAAAAAAA AAAAAAAAAG TGTTCTGATT TACATAATAG TTTACAGGAA
1551 GAAATATGTC TTAAGGGGGT TTTGCTAGCA AAAAACCAGA ATCTGTCAAA
1601 TCAACTTGAC AGTCTGAAAG ATACGAACAG TCGCCTTATA GATTATGTAA
1651 AATCAATAAA TAAAAAAGTT GGAACCGCGA ATAGTGAAAA AGAGTTTTCG
1701 GAAATTACCG AAAATAACAA ATCAAGGAAA ATAAAAGAAT TTAAATCGAA
1751 GGCTGAAACT GCGCTTTGGT TTGTAGAGTC ATATGGCCTT GTACCCCAGT
1801 ACTTAAAATT AGAAAGTACT GATGGGCAGA CTGTTAAAGT CGATTTCAAT
1851 CCGTCGTCCA GCAAATCTTC ATACCAAGAT TTGCCTGAAG AAGAAAGACA
1901 AAAAATTAAA GATTTGTTAT TTATTTTGGA AAAATTTAAT GTTAGCGAGA
1951 GCGCTAAAAG GGAACTGACA GTATTTTGTG ATGGGCTACC ACGAACATAT
2001 CTGGTTTCAT CAATGCCAGG AAGATATTAA CAGTTTATAT CATTTTGAAA
2051 GAATTCCGGG AAATATCCCT GGCGCATATG TTAGTCTAGA AAGCGAAATT
2101 ATCCGGTACA TCAAGTATCA TGTAAATCCT GAAACAGACA AGATAAAAAT
2151 TAAAATTTCG GGGGACGGGT CAAAAGTTAG TAGAATTTCA AACTTTGTCG
2201 TGTTGTCCTT TTCTGTAATT ACCGATGATT TGACATTATC ATCAAAAGAT
2251 CAAAATGTTT TTTGCATTGT AAACTGCAAA GAAGATTATG ATCATTTAAA
2301 ACTTGCTTGC AAACCCATTT TTCAGAAAAT AAACACATTA TACGAAAAAG
2351 CAAGTATTGA AGTTGAGGGG AAACATTTTG ATTTAGATAT TTTGATGGGT
2401 GGTGATATGA AATTTTTGCA ACTTGTGCTT GGGTTGGGTG GATCTCTATG
2451 TAATTATTCC TGTCCATGGT ATAGGGTCCA TAAAAACCAG AGGGACGACA
2501 TGACAAAGCC ATTGGATTTT TACCATACTC GTGGTATGCA GAGAACATCA
2551 CAGAATCTGA AGGAAGATGT CGTAAAGAAT GACTTTGGTG TTCGTGCTCA
2601 ACCTCTTGTT AGTATTGAGC CTGAACACAT AATCATTGAT GAACTTCATC
2651 TTCTTCTTCG GATATGTGAT AAACTGTTAA GGAACTTAAT ATTAGACACA
2701 AAAACGCTCG ATGACAAAAA TGCCGTTCAT GGTGAGAAGT CAGATTTCTT
2751 AGGACAGCTT ACTGAGAAAA TAAGAGGATG CGGCGTTTCG TTTTACATTT
2801 GGACAAAAAA GGGTACTCAA GGTGAGTTGG ACTGGTCGTC ATTGACGGGG
2851 TCTGACTATT AAAAGCTTTT GGAAAATCTT CCTTCAAAAT TATGCTTTTT
2901 GATCCATCAT GATACCCATG ATCATACTGT CAAAATTTGG AGTTCATTTT
2951 TGAATTTATA TCGTTTCGTG ACGTTATAAA TACACGAGTT CTCTAACATT
3001 GAAGATATTT TTGAAAAATG TAAAGGGTGG GTAAATGACT TTCTTGCTTT
```

3051 AGGATCATTG GGGAGAAAAG GTTTTGATAG CGTTACTCCT TATATGCATT
3101 GTCTTGTATA CCATGTTCCG TTTTTCACTA AAAAGTATGG AAAACTTTTA
3151 CGTTTCAGTG GGCAGGGCGT AGAAAAGATC AATGATGATA TAAAAAAAAA
3201 TCATCATTCA AAAAACAAAC AAATGGAAAG CAACGGTCGA

The transcript and protein sequences of *CthTransib_0066* were predicted by AUGUSTUS.

```
FXLX01000066.1:1-3240 AUGUSTUS   gene       1     3240   0.31   +   .   ID=g1
FXLX01000066.1:1-3240 AUGUSTUS   transcript 1     3240   0.31   +   .
          ID=g1.t1;Parent=g1
FXLX01000066.1:1-3240 AUGUSTUS   intron     1     1668   0.57   +   .   Parent=g1.t1
FXLX01000066.1:1-3240 AUGUSTUS   intron     1799  2051   0.59   +   .   Parent=g1.t1
FXLX01000066.1:1-3240 AUGUSTUS   CDS        1669  1798   0.52   +   2
          ID=g1.t1.cds;Parent=g1.t1
FXLX01000066.1:1-3240 AUGUSTUS   CDS        2052  3240   0.62   +   1
          ID=g1.t1.cds;Parent=g1.t1
coding sequence = [ttggaaccgcgaatagtgaaaaagagttttcggaaattaccgaaaataacaaatcaaggaaaataaaagaatttaaatc
gaaggctgaaactgcgctttggtttgtagagtcatatggccttgtaccccaaattccgggaaatatccctggcgcatatgttagtctagaaagcgaaa
ttatccggtacatcaagtatcatgtaaatcctgaaacagacaagataaaaattaaaatttcgggggacgggtcaaaagttagtagaatttcaaacttt
gtcgtgttgtccttttctgtaattaccgatgatttgacattatcatcaaaagatcaaaatgtttttttgcattgtaaactgcaaagaagattatgatca
tttaaaacttgcttgcaaacccatttttcagaaaataaacacattatacgaaaaagcaagtattgaagttgaggggaaacattttgatttagatattt
tgatgggtggtgatatgaaatttttgcaacttgtgcttgggttgggtggatctctatgtaattattcctgtccatggtatagggtccataaaaaccag
agggacgacatgacaaagccattggatttttaccatactcgtggtatgcagagaacatcacagaatctgaaggaagatgtcgtaaagaatgactttgg
tgttcgtgctcaacctcttgttagtattgagcctgaacacataatcattgatgaacttcatcttcttcttcggatatgtgataaactgttaaggaact
taatattagacacaaaaacgctcgatgacaaaaatgccgttcatggtgagaagtcagatttcttaggacagcttactgagaaaataagaggatgcggc
gtttcgttttacatttggacaaaaaagggtactcaaggtgagttggactggtcgtcattgacggggtctgactattaaaagcttttggaaaatcttcc
ttcaaaattatgcttttgatccatcatgatacccatgatcatactgtcaaaatttggagttcatttttgaatttatatcgtttcgtgacgttataaa
tacacgagttctctaacattgaagatatttttgaaaaatgtaaagggtgggtaaatgactttcttgctttaggatcattggggagaaaaggttttgat
agcgttactccttatatgcattgtcttgtataccatgttccgtttttcactaaaaagtatggaaaacttttacgtttcagtgggcagggcgtagaaaa
gatcaatgatgatataaaaaaaaatcatcattcaaaaaacaaacaaatggaaagcaacggtcga]
protein sequence = [GTANSEKEFSEITENNKSRKIKEFKSKAETALWFVESYGLVPQIPGNIPGAYVSLESEIIRYIKYHVNPETDKIKIKI
SGDGSKVSRISNFVVLSFSVITDDLTLSSKDQNVFCIVNCKEDYDHLKLACKPIFQKINTLYEKASIEVEGKHFDLDILMGGDMKFLQLVLGLGGSLC
NYSCPWYRVHKNQRDDMTKPLDFYHTRGMQRTSQNLKEDVVKNDFGVRAQPLVSIEPEHIIIDELHLLLRICDKLLRNLILDTKTLDDKNAVHGEKSD
FLGQLTEKIRGCGVSFYIWTKKGTQGELDWSSLTGSDYQKLLENLPSKLCFLIHHDTHDHTVKIWSSFLNLYRFVTLQIHEFSNIEDIFEKCKGWVND
FLALGSLGRKGFDSVTPYMHCLVYHVPFFTKKYGKLLRFSGQGVEKINDDIKKNHHSKNKQMESNGR]
```

No potential TIRs could be reliably identified surrounding the CthTransib_0066.

Entomoplasmatales bacterium

19. EbaTransib_0044

>MVYV02000044.1:1-2606 Entomoplasmatales bacterium EntAcro10 NODE_44_length_2606_cov_2.76038_ID_87, whole genome shotgun sequence

1 CCGTAGGGCA CAGCAAAAAC ACTTTGGGGG GGGAAGAGAG CCGGGCGACA
51 GATCAAAGTT GATCCACCCG TCCCACACCC TTCCCCCCGT TGCCACCAAC
101 AAGCTTATGG CGTCGTCGGA GAGACACCAC AAGCGCAGGA GCCCCGAGGG
151 GTTCCTGCGT CCTTCGAATG CCTCAACGGG GCGGCCAGTC TCCCGGGCCG
201 CAACCCGTGC GATTGCCTCC GGGCCACAGT CCGGGGAGGC CCCCACAATC
251 CGAAACGCCA TCGGGGCGAT CGGGACGCTG AAGAGAACCT CCCAATTACA
301 TGGGAGAACA AAAATTCAGT AACAAAAATC TTCCTCTTTG TCGGATTCGT
351 ACATTGCCGC AGATTCACTT TCCGAAGCAG ATGAATCTGA TACGTATGAG
401 GCCGTTTGCT GTGGCAACAT TTCCGGTGGC TTAAGAAGTT ATACCGTTTT
451 CGCTATCAAC GACTTCAGTG GTTTTTGCGG GAGTTTTCTG ACGCTTGAAA
501 TGTATGGATC AGAGGTGACC AGTAGTCTAT TAAAGACATC ATGTAAATTT
551 GAGGTTCTTG AACATTTCCT TGAGAAGTCT TCACGGAAAC GTTTGATATC
601 TTTATTCCGC GAATCCTGTG CATCCTCAGA AAGTTGACCA ATCGGCAATA
651 ACGCTTTTTC AATAATTTCT GGTCCGTGTA TTAGCAATTT ATGTATGGAT
701 GTTGGCATGT AGTACCATGG GTATAGGTTT ACAAATAGAC GTGCAGTTTC
751 CACAGAGTAT TCTCTAAATT TGTCGTTATC TACATCGTAA CCACTTGAGA
801 TAGCTTGCAA AATGGTATGA AATCGATGAA TTAAGCCTTC ATTCACTCCT
851 GTTATTCTTG CGGCCGAAAT CGCATTTTCG AAATATCGAC GTGCCGTATT
901 GCCATCATTC GTACTACCAA ACCCTGGCTT TGGGCGATCG ACGATAAGTC
951 CGAGTTGGGA AAAAAATGCT TTTTGTATAG TAGCTTTTCT GTCTGCAATT
1001 ATCTTCTTTT CATCATCTTT GCGCGATTGC CATTTTTGAG TGTTAAGTTT
1051 ATAAATTAAA TGTAGCAGGC ATTCAAAACA GCGAATCCAG GCGTGCAGAG
1101 TTGACAAACC AAATCTCAGA TTGTCTACAT TTATTTCATT AGCAAGCATC
1151 TTGTCAATAT CATTGAATTC TTTTGACGTT GCTTTACACA AGAAGCAACG
1201 CTGCGCCGAG GTGGTGCCAG TTACAGAATT GCACACTTTT CCATCGATCA
1251 TTGTGAAGGA CATGTTGTAT GTAACTGATA CTGACTTTCC GTTCATGACA
1301 ATTGACATCG GTTGAAGGGC TTCGATCTGA TTTTCAATCC CTGTAGCTTC
1351 ATTCACCGTG AGTTGTACAT TTTCATGAGC AAACTGTACT TTGATAGGCC
1401 GACAAAACCG TGCAGAAGAT GGTCTCGGGT TTTTCCAAAC GATGACTTGG
1451 TTTTTTGTTC GGTCGTCTGT GCCTAACAGC TGTAAAGGTA CCATTGATGT
1501 AAAAAATATA TTATCGTCGC ATTTTCCATC TTCGCTGAAC TTTTGTTTAT
1551 ACACACTTTG GCCGGAACTA CCGTCACAAC CCCACTTGCA GTACAATGTT
1601 AAATTATTGG CAATTTCAGG TGGTAAGGTT GCAAATACAT TTTCCTGTAG
1651 CAGCAAAATT CGATTTGCAG TGTGGTTCAG TAGAGCTTGT AACTTGACTT
1701 CAGTACAGCT CTCGCTGACA GAAAAAGTGT TTGTTGGTGG ATAGCAGCGA
1751 TGTTTTGCAG TCATTACCTC TTTATAAGAC GGATATAATT TACAATTATT
1801 TTCTTTAGCG ATATTTCGTA TACCCTGATA AGTACGCTTT GGAAATCGAT
1851 TTTCCACAAG TACCGAAAGT GCTTGATCCG GAGACAAAGT ATTTTCACTT
1901 GTCGACTTGA GAGCGCTAAT ATACTGAGAT GCTTTTGATG GGCTCGTAGA
1951 AGTTATATCT TGGATGACTT TAGCAGCTTG TGATTGACCT GATGACCTAA
2001 GACTCATTTG AGTCGCGTAA GCCAATTCCG CTAAAGAAAC GGTATTGCGC
2051 AGTTCTTGAG TTTTTCTCCT CTTTGATCTT TCACTCGAAG ACGAAAATGA
2101 CAGCGAAGGG CGACCAATCT TCATATTGTC TTTTAACGGA ATTGATGTTC
2151 TACATTCACT GCTAGGTAAG GTACTGAATT CCACGACAGC TTTTAACCAT

```
2201 TCTTCGTTGT TTTTTAAAAA CGCTGCTTCG TTCCTAAACG CCGAAGCCCA
2251 TCTGGTTCTG ATGCCGGAAA ATAGCAGATT CATGGTGTGC TTCATATCAG
2301 GTCCTATATC GATGAAAAGT CCAGTATTTT GTTTTACAGA TGTTATCAAG
2351 ACATCGACTA TGTTATCATA ACTGTCTTTC ATATGGTTTT TGATTATGAA
2401 GAAAATATCC TTCCTCGAGT AAGACGGATT TTTGTCAGAC GATCCTAAAA
2451 ATCAATAAAT ATAAGATTAA AATTTAATGC GACTTTTAAA AATATAATGA
2501 AATGATAAAA TTTAATCGTT ATTTCGTTAC AGGAAACAAG AATTATTGAT
2551 AGCAGTCGCA GTCCCTTCGC ATTACTCGCT TTGTTTACTT CGAAGGCCGG
2601 CAGAGA
```

The transcript and protein sequences of *EbaTransib_0044* were predicted by AUGUSTUS.

```
MVYV02000044.1:1-2606   AUGUSTUS   transcript   316   2572   0.01   -   .   ID=g1.t8;Parent=g1
MVYV02000044.1:1-2606   AUGUSTUS   stop_codon   316   318   .   -   0   Parent=g1.t8
MVYV02000044.1:1-2606   AUGUSTUS   intron   2358   2425   0.01   -   .   Parent=g1.t8
MVYV02000044.1:1-2606   AUGUSTUS   intron   2445   2530   0.01   -   .   Parent=g1.t8
MVYV02000044.1:1-2606   AUGUSTUS   CDS   316   2357   0.01   -   2   ID=g1.t8.cds;Parent=g1.t8
MVYV02000044.1:1-2606   AUGUSTUS   CDS   2426   2444   0.01   -   0   ID=g1.t8.cds;Parent=g1.t8
MVYV02000044.1:1-2606   AUGUSTUS   CDS   2531   2572   0.01   -   0   ID=g1.t8.cds;Parent=g1.t8
MVYV02000044.1:1-2606   AUGUSTUS   start_codon   2570   2572   .   -   0   Parent=g1.t8
MVYV02000044.1:1-2606   AUGUSTUS   protein_match   510   557   3.48   +   0
ID=pp.g1.t8.unknown_A;Target=unknown_A 1 16;Target_start=620;
MVYV02000044.1:1-2606   AUGUSTUS   protein_match   711   791   2.16   +   0
ID=pp.g1.t8.unknown_B;Target=unknown_B 1 27;Target_start=542;
MVYV02000044.1:1-2606   AUGUSTUS   protein_match   819   869   3.93   +   0
ID=pp.g1.t8.unknown_C;Target=unknown_C 1 17;Target_start=516;
MVYV02000044.1:1-2606   AUGUSTUS   protein_match   882   962   3.91   +   0
ID=pp.g1.t8.unknown_D;Target=unknown_D 1 27;Target_start=485;
MVYV02000044.1:1-2606   AUGUSTUS   protein_match   1011   1043   7.76   +   0
ID=pp.g1.t8.unknown_E;Target=unknown_E 1 11;Target_start=458;
MVYV02000044.1:1-2606   AUGUSTUS   protein_match   1047   1070   8.03   +   0
ID=pp.g1.t8.unknown_F;Target=unknown_F 1 8;Target_start=449;
MVYV02000044.1:1-2606   AUGUSTUS   protein_match   1089   1130   4.79   +   0
ID=pp.g1.t8.unknown_G;Target=unknown_G 1 14;Target_start=429;
MVYV02000044.1:1-2606   AUGUSTUS   protein_match   1164   1298   3.39   +   0
ID=pp.g1.t8.unknown_H;Target=unknown_H 1 45;Target_start=373;
MVYV02000044.1:1-2606   AUGUSTUS   protein_match   1326   1388   4.09   +   0
ID=pp.g1.t8.unknown_I;Target=unknown_I 1 21;Target_start=343;
MVYV02000044.1:1-2606   AUGUSTUS   protein_match   1404   1427   4.99   +   0
ID=pp.g1.t8.unknown_J;Target=unknown_J 1 8;Target_start=330;
MVYV02000044.1:1-2606   AUGUSTUS   protein_match   1488   1562   7.03   +   0
ID=pp.g1.t8.unknown_L;Target=unknown_L 1 25;Target_start=285;
MVYV02000044.1:1-2606   AUGUSTUS   protein_match   1566   1595   3.71   +   0
ID=pp.g1.t8.unknown_M;Target=unknown_M 1 10;Target_start=274;
```

MVYV02000044.1:1-2606 AUGUSTUS protein_match 1608 1643 3.45 + 0
ID=pp.g1.t8.unknown_N;Target=unknown_N 1 12;Target_start=258;
MVYV02000044.1:1-2606 AUGUSTUS protein_match 1650 1679 5.09 + 0
ID=pp.g1.t8.unknown_O;Target=unknown_O 1 10;Target_start=246;
MVYV02000044.1:1-2606 AUGUSTUS protein_match 1689 1727 8.19 + 0
ID=pp.g1.t8.unknown_P;Target=unknown_P 1 13;Target_start=230;
MVYV02000044.1:1-2606 AUGUSTUS protein_match 1743 1805 4.33 + 0
ID=pp.g1.t8.unknown_Q;Target=unknown_Q 1 21;Target_start=204;
MVYV02000044.1:1-2606 AUGUSTUS protein_match 1809 1895 4.24 + 0
ID=pp.g1.t8.unknown_R;Target=unknown_R 1 29;Target_start=174;
MVYV02000044.1:1-2606 AUGUSTUS protein_match 1932 2117 4.73 + 0
ID=pp.g1.t8.unknown_S;Target=unknown_S 1 62;Target_start=100;
MVYV02000044.1:1-2606 AUGUSTUS interblock_region 316 509 . - 0
ID=pp.g1.t8.iBR1
MVYV02000044.1:1-2606 AUGUSTUS interblock_region 558 710 . - 0
ID=pp.g1.t8.iBR0
MVYV02000044.1:1-2606 AUGUSTUS interblock_region 792 818 . - 0
ID=pp.g1.t8.iBR1
MVYV02000044.1:1-2606 AUGUSTUS interblock_region 870 881 . - 0
ID=pp.g1.t8.iBR2
MVYV02000044.1:1-2606 AUGUSTUS interblock_region 963 1010 . - 0
ID=pp.g1.t8.iBR3
MVYV02000044.1:1-2606 AUGUSTUS interblock_region 1044 1046 . - 0
ID=pp.g1.t8.iBR4
MVYV02000044.1:1-2606 AUGUSTUS interblock_region 1071 1088 . - 0
ID=pp.g1.t8.iBR5
MVYV02000044.1:1-2606 AUGUSTUS interblock_region 1131 1163 . - 0
ID=pp.g1.t8.iBR6
MVYV02000044.1:1-2606 AUGUSTUS interblock_region 1299 1325 . - 0
ID=pp.g1.t8.iBR7
MVYV02000044.1:1-2606 AUGUSTUS interblock_region 1389 1403 . - 0
ID=pp.g1.t8.iBR8
MVYV02000044.1:1-2606 AUGUSTUS interblock_region 1428 1487 . - 0
ID=pp.g1.t8.iBR9
MVYV02000044.1:1-2606 AUGUSTUS interblock_region 1563 1565 . - 0
ID=pp.g1.t8.iBR10
MVYV02000044.1:1-2606 AUGUSTUS interblock_region 1596 1607 . - 0
ID=pp.g1.t8.iBR11
MVYV02000044.1:1-2606 AUGUSTUS interblock_region 1644 1649 . - 0
ID=pp.g1.t8.iBR12
MVYV02000044.1:1-2606 AUGUSTUS interblock_region 1680 1688 . - 0
ID=pp.g1.t8.iBR13
MVYV02000044.1:1-2606 AUGUSTUS interblock_region 1728 1742 . - 0
ID=pp.g1.t8.iBR14

MVYV02000044.1:1-2606   AUGUSTUS   interblock_region   1806   1808   .   -   0
ID=pp.g1.t8.iBR15
MVYV02000044.1:1-2606   AUGUSTUS   interblock_region   1896   1931   .   -   0
ID=pp.g1.t8.iBR16
MVYV02000044.1:1-2606   AUGUSTUS   interblock_region   2118   2357   .   -   2
ID=pp.g1.t8.iBR17
MVYV02000044.1:1-2606   AUGUSTUS   interblock_region   2426   2444   .   -   0
ID=pp.g1.t8.iBR17
MVYV02000044.1:1-2606   AUGUSTUS   interblock_region   2531   2572   .   -   0
ID=pp.g1.t8.iBR17

coding sequence = [atgcgaagggactgcgactgctatcaataattcttgtttcctgatcgtctgacaaaaatcctcgatgtcttgataacat
ctgtaaaacaaaatactggacttttcatcgatataggacctgatatgaagcacaccatgaatctgctattttccggcatcagaaccagatgggcttcg
gcgtttaggaacgaagcagcgtttttaaaaaacaacgaagaatggttaaaagctgtcgtggaattcagtaccttacctagcagtgaatgtagaacatc
aattccgttaaaagacaatatgaagattggtcgcccttcgctgtcattttcgtcttcgagtgaaagatcaaagaggagaaaaactcaagaactgcgca
ataccgtttctttagcggaattggcttacgcgactcaaatgagtcttaggtcatcaggtcaatcacaagctgctaaagtcatccaagatataacttct
acgagcccatcaaaagcatctcagtatattagcgctctcaagtcgacaagtgaaaatactttgtctccggatcaagcactttcggtacttgtggaaaa
tcgatttccaaagcgtacttatcagggtatacgaaatatcgctaaagaaaataattgtaaattatatccgtcttataaagaggtaatgactgcaaaac
atcgctgctatccaccaacaaacacttttctgtcagcgagagctgtactgaagtcaagttacaagctctactgaaccacactgcaaatcgaattttg
ctgctacaggaaaatgtatttgcaaccttaccacctgaaattgccaataatttaacattgtactgcaagtggggttgtgacggtagttccggccaaag
tgtgtataaacaaaagttcagcgaagatggaaaatgcgacgataatatatttttacatcaatggtacctttacagctgttaggcacagacgaccgaa
caaaaaaccaagtcatcgtttggaaaaacccgagaccatcttctgcacggttttgtcggcctatcaaagtacagtttgctcatgaaaatgtacaactc
acggtgaatgaagctacagggattgaaaatcagatcgaagcccttcaaccgatgtcaattgtcatgaacggaaagtcagtatcagttacatacaacat
gtccttcacaatgatcgatggaaaagtgtgcaattctgtaactggcaccacctcggcgcagcgttgcttcttgtgtaaagcaacgtcaaaagaattca
atgatattgacaagatgcttgctaatgaaataaatgtagacaatctgagatttggtttgtcaactctgcacgcctggattcgctgttttgaatgcctg
ctacatttaatttataaacttaacactcaaaaatggcaatcgcgcaaagatgatgaaaagaagataattgcagacagaaaagctactatacaaaaagc
atttttttcccaactcggacttatcgtcgatcgcccaaagccagggtttggtagtacgaatgatggcaatacggcacgtcgatatttcgaaaatgcga
tttcggccgcaagaataacaggagtgaatgaaggcttaattcatcgatttcataccattttgcaagctatctcaagtggttacgatgtagataacgac
aaatttagagaatactctgtggaaactgcacgtctatttgtaaacctatacccatggtactacatgccaacatccatacataaattgctaatacacgg
accagaaattattgaaaaagcgttattgccgattggtcaactttctgaggatgcacaggattcgcggaataaagatatcaaacgtttccgtgaagact
tctcaaggaaatgttcaagaacctcaaatttacatgatgtctttaatagactactggtcacctctgatccatacatttcaagcgtcagaaaactcccg
caaaaaccactgaagtcgttgatagcgaaaacggtataacttcttaagccaccggaaatgttgccacagcaaacggcctcatacgtatcagattcatc
tgcttcggaaagtgaatctgcggcaatgtacgaatccgacaaagaggaagattttgttactga]

protein sequence = [MRRDCDCYQQFLFPDRLTKILDVLITSVKQNTGLFIDIGPDMKHTMNLLFSGIRTRWASAFRNEAAFLKNNEEWLKAV
VEFSTLPSSECRTSIPLKDNMKIGRPSLSFSSSSERSKRRKTQELRNTVSLAELAYATQMSLRSSGQSQAAKVIQDITSTSPSKASQYISALKSTSEN
TLSPDQALSVLVENRFPKRTYQGIRNIAKENNCKLYPSYKEVMTAKHRCYPPTNTFSVSESCTEVKLQALLNHTANRILLLQENVFATLPPEIANNLT
LYCKWGCDGSSGQSVYKQKFSEDGKCDDNIFFTSMVPLQLLGTDDRTKNQVIVWKNPRPSSARFCRPIKVQFAHENVQLTVNEATGIENQIEALQPMS
IVMNGKSVSVTYNMSFTMIDGKVCNSVTGTTSAQRCFLCKATSKEFNDIDKMLANEINVDNLRFGLSTLHAWIRCFECLLHLIYKLNTQKWQSRKDDE
KKIIADRKATIQKAFFSQLGLIVDRPKPGFGSTNDGNTARRYFENAISAARITGVNEGLIHRFHTILQAISSGYDVDNDKFREYSVETARLFVNLYPW
YYMPTSIHKLLIHGPEIIEKALLPIGQLSEDAQDSRNKDIKRFREDFSRKCSRTSNLHDVFNRLLVTSDPYISSVRKLPQKPLKSLIAKTVQLLKPPE
MLPQQTASYVSDSSASESESAAMYESDKEEDFCY]

No potential TIRs could be reliably identified surrounding the EbaTransib_0044.

Plants

Capsicum chinense

20. CchTransib_3309

>MCIT02003309.1:1-3586 Capsicum chinense isolate PI159236 CCv1.2.scaffold4857, whole genome shotgun sequence

1 AATTCATCAA TCTCATGGTA ATTTAAAAAG GTAGAAATTC GTTATTTTAA
51 CTCGCTACGA ACTACATGTC ACTCTCATAT ATGTGGACTC CCTTCCCCAC
101 CATCCCCATC ACAAACACAA ATGTTGCATA TTTTAAGAAA ACATTTTGTA
151 TAAGGTAACA GAAAGCACAC TTTACTGAAA AAATAAATCA CATACTGAGA
201 TCGAAAAAAT GACTGAGATC ATAATTTGCT TTAGGAAAAA ATGTATAAAA
251 CAAAACTTAA CGGATAAAGT TAAAAAAAAA TTAATCACAT TTCAATGAGA
301 AAACAAACAA AAACAATTAA AAATACACGA TCCTAGCTTT GATTCAATCT
351 TCCATATCCA TATTCTCGTC ACTCTCCTCG TCCATTTCTT CATCTATCCA
401 TTCGTCTATT TCTTCGTCTA TGTCTTCATC CATTTCTTCG TCTTCATCGT
451 CATCGCTCTT CCCTTCTTCC TCGACTTTCT CTTCTTCATC TTCTTCTTTA
501 TCTTCCTCCA ATTTCAAATG ATCAAAACAA GGATTTGGCT CTAAAAGATC
551 CAAAGCGGCA CGTGGCAATT TTGTAGCTTT CTTTATTACG TGGGACTTCA
601 GGATTACAGG ATCTGACGAG CACATCATCC GACACAAAAT ATCTATATTT
651 GTTGCCTCTC TTGATCTTTT CCTCGAAAAA CATTTTCTAA AAAGTCTATA
701 TACCTTATTT TGAGCTTCCA AAGCCTCCTC AGTGTATTGC CCAATTGGCA
751 GCTCCATTGA TTTGACGATT TCAGCTCCGT GAATCAGAAC TTTATGCGCT
801 GTAGGAGACA TTGGATACCA TGGATAAATT TCTACGTAGA ATTCTGCTGT
851 TTCTTTGGAA TACTCTTTGA AAACGTCATA TTTAATCGCG TAGCCACTTG
901 CTAGAACTGC GAGAATTAGG TGGAATCGGT GTATTAATCC AGGAACGATC
951 TGTGTGATTT TTGATGACAC GGGAGGATTT TTGAAAAACC TTCTAGCAGT
1001 GTTTCCACAG TTGGAATTGC CTCCGCCTTG TTTCGGTTGG TCCACGATTA
1051 ATCCAATTTC CTTAAACTCG CGTTGAATTC TTTTCTTCTC CTCATTTTTG
1101 ATAACTTTTT CGGCTGGAAT AATAATTCGC CATTTTTTCA GTTTTAGGCG
1151 ATACGCAATA TGGAGCAGTA GCTCAAAAAT TTTGATCCAC GCATGTAAGG
1201 TCGATAGACC AAGGCAATAC AAGTCGGTTC GAGGGGTTTT AGAGAATGCT
1251 AATTTTAGAT CGTTTGCCTT AGAGGGTAGA ATACCGCACA GATAACATGT
1301 GCTTGCGCTT TTGGTGCCTG ATAAACAATT ACACATCTTA GTGTCCAGCA
1351 TTGTAAGCTT TAAGGTATAG GAAACTTCAA TATTCACCCC GTCTTTGATC
1401 ACCTTTGTTG GCACTAAGTT TGCAATCTCA CTTTCTAAAC TAGCAAGCTG
1451 GTCTAACACA AAGTCATCCT CCTCTTTTTT GAAAATAAAC TTGATCGGTC
1501 GACAGTATTG GGGTGATGCG GGTTTAGGAT TTTGCCAAAA AATTCCACCT
1551 GGTTGCCCAT TTGCAGCTCT TAAAATTAAT GGCACTGCGC ATATGACAAA
1601 TAAGTTTTTA TCGGACGAAT CTTTGAATTC CTCGGAAAAT TTTTGGTTAT
1651 ACTCACTTTG ATTGCTTGAT CCGTCAAAGC CGTATTTCCA GAGGAAATGT

```
1701 AAAGTATGGG GATTATCTGT ACGGTGGTTA ATCAGCGAAG ATTCATATTT
1751 CGTCCATTCG GCCTCTAAAA GACGGTTTGT GGTGATGTCA AGTAACGCTT
1801 GCAATGAAAT TTCGCCGGAG ATCTCTGTAA TTTTTATTGC ATCATTTCCG
1851 GGCATGCATT TTTTCTTTTC TTCTAAAACA TAATTATAAG GAGGAAAGAT
1901 GTCAGCCTTC CTGAGTTTGG ACTCTTTTCT TGTGTTGACA TATTGGGATT
1951 TTGTAAAGTT ATTTTTGATC ATGAATGCAA GAGCTTCGCT GGGTAAAAGA
2001 GGCACGGGCT CTTCGTTCTT TCGCCCTAAC ACTACGTTTT TCAGATCTAC
2051 AACTTTTTCA GGTGTTGCTG AAATAGCCTT AACAACCTGC GATTTGGACC
2101 TTTCCCCTTC CTTCCAAAGT GCAGTTGTAT ACGCTACTCC GAGCTGTGAA
2151 GAGGAATGAC TGGTCACCAA ATCATGGACT TTTCGCTGCT GCACTTTTAT
2201 GCTGCAGCTC TCAAAGTCTT TAGAGGCTCG ACTCGTGCTT GGATGAGTAT
2251 TGAGAGAGGG ATCTTCAGAG AGAGGAAATA CTACCTTGCA AAGCCAATCT
2301 CCATGCAGTT TCCTCAGCCT CTCTTCGCAC CTATGACATT CCTTCCACTT
2351 CTGTCTGAAC ATCATAATCA GCGCATATAA TTTTTTCTCT CCATCTTCAG
2401 AAATGATTGC AGCGGGTATA TTGCGTAACA AAAAAGCCTT CAGAAGATCC
2451 ATTAACCTGC TGATTGAGGT GCTGGTCAAC CCAACTTTCT TTGCCTCTTG
2501 AAATAATTCC TCTCTTGTCA CAGAAACGAC TGCATGATCT GAAAATTAAG
2551 TAAAACTACT ATACAACCGG TAAAAATAAT TACAAACTTC AGACCTAATG
2601 AAAAAATTAA ATAATATTTC TTGACTTCGA TACATCTGAC ATTACAGGGA
2651 TAATCAGCCT TTCCTAGAGG CCCTTGTGGA GGAAAGATCA ATTTTTAACG
2701 AAATTTGGCA ACACAGTTGA CAATTTAAAT TTGTCATACT TAGTCGAAAA
2751 TGTCATAATA ATCTACGACT CCGCATAAAT TTGTCATTTC AGCGATAATC
2801 GGCCTTCCCT GGCGGGTCTT ATGAAAACGT TATATTTTCG GCCAAATTTG
2851 GCAGCAATGG ATTCTGATGC AATTTGACAT TATTTAAAAT TGCAACAATA
2901 TAGGCTAAAC TTGGCAACAC CGCAAAATAT TTGGCCGTGC TCTCTAGCTG
2951 GAAGAGACAA GCGACCTTAT CTATCGTGTT ATTGAATCAA AACACTGAGT
3001 CACATTTCAA ATCGCAGAGA TGCGCTCCGA AAAAAATTGA TTTCGACCGT
3051 TAGTTGCAAA AAGGCGCAAT TAAACTTAGC ATCATTTTAA ACTTAAGGAT
3101 ATGTGGAACA AGGTTTTTCA ATTCCCAAAG CATTAACTAG AATTAAAAAA
3151 AAGTTAGAGC TTCCTAAAGA TTATAAATTT CACTGATTTC TAGCCTTTTT
3201 CGGTGTATGT TTGCAGCGTT AAAAAGCATA CAAATTTCGG CTTAAAATTA
3251 ATGATACTGG GAAAAACGGA CGTCACTTAC CATCAGGTGG ATCCATATTG
3301 CCCAAAATTT CACATTGATG AGGCGTAACT CGGAAAAACA AACACTTTGT
3351 AACGCACTAC TGAATGCGGC GCACAATCAG CTAATGAAGA CCTGTTGCGC
3401 GCTCTGTTGT TAAGGCCTGT CCCTGTCTTA TCAATCATTA TTAATCGATG
3451 AAAACTCAGG TGATGATCTC TGAGAGTATG TTTGATAAAA CTGTGGTGCC
3501 AAACTTTCAT TTTTTGCACC TTCAAAATTT GCTTAAAGTT CCGTTAAATC
3551 CGGTCTGTGG ACCATAGTGC GGCGCGGCGT GAGCTA
```

The transcript and protein sequences of *CchTransib_3309* were predicted by AUGUSTUS.

```
MCIT02003309.1:1-3586 AUGUSTUS gene 344 3296 . - . ID=g1
MCIT02003309.1:1-3586 AUGUSTUS transcript 344 2452 0.96 - . ID=g1.t1;Parent=g1
MCIT02003309.1:1-3586 AUGUSTUS stop_codon 344 346 . - 0 Parent=g1.t1
MCIT02003309.1:1-3586 AUGUSTUS CDS 347 2452 0.96 - 0 ID=g1.t1.cds;Parent=g1.t1
```

MCIT02003309.1:1-3586 AUGUSTUS start_codon 2450 2452 . - 0 Parent=g1.t1

coding sequence = [atggatcttctgaaggctttttgttacgcaatatacccgctgcaatcatttctgaagatggagagaaaaaattatatg
cgctgattatgatgttcagacagaagtggaaggaatgtcataggtgcgaagagaggctgaggaaactgcatggagattggctttgcaaggtagtattt
cctctctctgaagatccctctctcaatactcatccaagcacgagtcgagcctctaaagactttgagagctgcagcataaaagtgcagcagcgaaaagt
ccatgatttggtgaccagtcattcctcttcacagctcggagtagcgtatacaactgcactttggaaggaaggggaaaggtccaaatcgcaggttgtta
aggctatttcagcaacacctgaaaaagttgtagatctgaaaaacgtagtgttagggcgaaagaacgaagagcccgtgcctcttttacccagcgaagct
cttgcattcatgatcaaaaataactttacaaaatcccaatatgtcaacacaagaaaagagtccaaactcaggaaggctgacatctttcctccttataa
ttatgttttagaagaaaagaaaaaatgcatgcccggaaatgatgcaataaaaattacagagatctccggcgaaatttcattgcaagcgttacttgaca
tcaccacaaaccgtcttttagaggccgaatggacgaaatatgaatcttcgctgattaaccaccgtacagataatccccatactttacatttcctctgg
aaatacggctttgacggatcaagcaatcaaagtgagtataaccaaaaattttccgaggaattcaaagattcgtccgataaaaacttatttgtcatatg
cgcagtgccattaattttaagagctgcaaatgggcaaccaggtggaatttttggcaaaatcctaaacccgcatcaccccaatactgtcgaccgatca
agtttattttcaaaaaagaggaggatgactttgtgttagaccagcttgctagtttagaaagtgagattgcaaacttagtgccaacaaaggtgatcaaa
gacggggtgaatattgaagtttcctataccttaaagcttacaatgctggacactaagatgtgtaattgtttatcaggcaccaaaagcgcaagcacatg
ttatctgtgcggtattctaccctctaaggcaaacgatctaaaattagcattctctaaaacccctcgaaccgacttgtattgccttggtctatcgacct
tacatgcgtggatcaaaatttttgagctactgctccatattgcgtatcgcctaaaactgaaaaaatggcgaattattattccagccgaaaaagttatc
aaaaatgaggagaagaaaagaattcaacgcgagtttaaggaaattggattaatcgtggaccaaccgaaacaaggcggaggcaattccaactgtggaaa
cactgctagaaggtttttcaaaaatcctcccgtgtcatcaaaaatcacacagatcgttcctggattaatacaccgattccacctaattctcgcagttc
tagcaagtggctacgcgattaaatatgacgttttcaaagagtattccaaagaaacagcagaattctacgtagaaatttatccatggtatccaatgtct
cctacagcgcataaagttctgattcacggagctgaaatcgtcaaatcaatggagctgccaattgggcaatacactgaggaggctttggaagctcaaaa
taaggtatatagacttttttagaaaatgtttttcgaggaaaagatcaagagaggcaacaaatatagatattttgtgtcggatgatgtgctcgtcagatc
ctgtaatcctgaagtcccacgtaataaagaaagctacaaaattgccacgtgccgctttggatcttttagagccaaatccttgttttgatcatttgaaa
ttggaggaagataaagaagaagatgaagaagagaaagtcgaggaagaagggaagagcgatgacgatgaagacgaagaaatggatgaagacatagacga
agaaatagacgaatggatagatgaagaaatggacgaggagagtgacgagaatatggatatggaagattga]

protein sequence = [MDLLKAFLLRNIPAAIISEDGEKKLYALIMMFRQKWKECHRCEERLRKLHGDWLCKVVFPLSEDPSLNTHPSTSRASK
DFESCSIKVQQRKVHDLVTSHSSSQLGVAYTTALWKEGERSKSQVVKAISATPEKVVDLKNVVLGRKNEEPVPLLPSEALAFMIKNNFTKSQYVNTRK
ESKLRKADIFPPYNYVLEEKKKCMPGNDAIKITEISGEISLQALLDITTNRLLEAEWTKYESSLINHRTDNPHTLHFLWKYGFDGSSNQSEYNQKFSE
EFKDSSDKNLFVICAVPLILRAANGQPGGIFWQNPKPASPQYCRPIKFIFKKEEDDFVLDQLASLESEIANLVPTKVIKDGVNIEVSYTLKLTMLDTK
MCNCLSGTKSASTCYLCGILPSKANDLKLAFSKTPRTDLYCLGLSTLHAWIKIFELLLHIAYRLKLKKWRIIIPAEKVIKNEEKKRIQREFKEIGLIV
DQPKQGGGNSNCGNTARRFFKNPPVSSKITQIVPGLIHRFHLILAVLASGYAIKYDVFKEYSKETAEFYVEIYPWYPMSPTAHKVLIHGAEIVKSMEL
PIGQYTEEALEAQNKVYRLFRKCFSRKRSREATNIDILCRMMCSSDPVILKSHVIKKATKLPRAALDLLEPNPCFDHLKLEEDKEEDEEEKVEEEGKS
DDDEDEEMDEDIDEEIDEWIDEEMDEESDENMDMED]

No potential TIRs could be reliably identified surrounding the CchTransib_3309.

21. CchTransib_2435

>MCIT02002435.1:1-5081 Capsicum chinense isolate PI159236 CCv1.2.scaffold3982, whole genome shotgun sequence

1 TTTCCGAATA AAATTGCATC GGAAATTTTT AAGTTAATTT TATTCATGTA
51 TGTCTAACAA AAATCAACAT ATTCAACCGC ATCTTATAAA TTTAGCTTTA
101 GCGACTCATT AAAATTAATT TCTTATTTAT TATTATTTAG ACATGAAAGA
151 AAAATTCCAA CTGGCTGATA AATAAAATAT TTTTTATGAT TTCTTTTGTA
201 AAATTCAATG CTAAATTTTT AACCTCACAT TCTTTTTTTC CCTACAGAAC
251 TTGTTGAAAT AAATCGCAAG GATCTCTTTG ATGAATGGAG GAATCCTAAT

301 GAACTACAGG ACAAAAACAA AGCTGTCTCC ACTTTTGTCC TGAATAAGAT
351 AAGATCTGAT GCAGAATCTT TAAATTTTGA TTCAGCATTG GATGTTCTCA
401 CTACAGAAGA TGCTGAGGCA AATCATGCAA GTATCAAAAA CTTCAATTCA
451 AAACTTTCTG CCAAGTGGAA AAAACATAAA AAAAATGAGC GTTTTCTCAT
501 GAATGAAGAA TCTTGGTTGG AGAAGAATGA GGTGTTCATC GTTGCTAGTT
551 GCTCACCATC TAAGAAGGAG GTTGGCAAGG AATTAAAAAC TAAAATGGGT
601 CGGCCGTCGA AAGAATTTTC CGACCTAAGT GTGGATGCAA AACGCCGGAG
651 AATTTCTAGT CTTATGGACC AAGCGACTGC GGAGGAGCTT AGTTTTGCGG
701 CCTCTTCTAG CTTATACATT GAGGGTAAAA GAGATGCGGC CAGTCTCGCT
751 CGTAAAATTT CTGAAACTCC TATTGAAACA GCGTTGTCAG TGAAAGATAC
801 AGAGAATCAA TTAATAAAGT CAGAGCTTCA TCCCTACACT CCTGATGAAG
851 CTTTGGCTTT ATTTATTGAC GGGCGATTTT CACGTGTCAC TTATGAGCGA
901 GTTCAGCAAT CAGCACGACG ACACAACGCA AATATTTACC CAATTTATGA
951 GCAAGTAAAG GTCGCAAAAA AAAGGTGCTA CCCTTCAAAT GAGTCAATTC
1001 ATATTACCGA AACCAGTGCC GAATCTACGT TACAGGGTAT CGTGGATCAC
1051 ACAACCAGCC GAATCTTTCA GATGATCGGA TATGAAAATG AGGCTTCGGA
1101 CCTCAAATTG CTGTACAAAT GGGGTGCGGA TGGTAGCTCT GGCTACTCTA
1151 TGTACAAGCA AAAGTGGTCC GAAAAAAACC AGGATGCCTC CGATTCTTCG
1201 CTGTTTTCCA TTTCTTTAGT CCCTATACAA CTAAGAAATA GAAGCAGCGA
1251 AGAAATTTTG TGGACAAATC CTCGTCCTAA TTCGGTCCGA TTTTGCCGGC
1301 CAATAAAAAT TTTACGCATC TCAGAAAGTA AGGAGATCAC TCAAGTCGAA
1351 ATAGGGAAGG TTAAAGACCA GATAAAAGCG ATTAATCCCA CCAGAATAAG
1401 CAGAGGCACA GCTCCTGATT GTGTTGTTTA TCCCGAATTT TTTTGTACCA
1451 TGATCGATGG CAAAATTTTA TGCGACTTAA CAAATACGTC TACACAAAGT
1501 TGCCCTATTT GCGGCGCTAC GCCAAAAGAT ATGAATGACA TTGATAAAGT
1551 GATGAAGAGG CCTTGCAACA AAAAAAATTA TGAGTACGGA TTGTCGACGT
1601 TGCACGCATA TATTCGATCC CTTGAATATA TGCTGCATGT AGCTTATCGC
1651 CTAGGACTAG AGAGAGAAAA TAGGAAATGG CGAATTTGTA GTAAAGAAGA
1701 TAAGTTAATT GTGAGAAATA AGAAAAAAAA CATACAATCA GAAATCAAAC
1751 AGGAGCTGGG CCTCTTGCTC GATGTCCCTA AAGTGAATAG TGGGAACACT
1801 AATGATGGTA ACTCCGCTAG GAGATTTTTC AAAAATGCAA AGAGAGTAGC
1851 ACAGATTACA GGCTTGGATG AAAACATCCT ACATCGGTTT GAAACCATCC
1901 TAATTGTACT TTCCAGCGGA TTTGCCATCA ATGTCGAGTC TTTCAGATCA
1951 TTTGCTATCG AAACTGCAAG GCTATGTGTC AAAGTATACC CGTGGTACTA
2001 TATGCCCCCT GCTGTCCATC GTGTGCTAAT TCACGGAGCA GACATAATTC
2051 AAGTAGCTCC TGTCCCGATT GGGCTATTAG GTGAAGATGC CAGCGAGTCT
2101 AAAAATAAAG ATATTAGGAG ATTTCAGGTG CATAGCACGA GAAAAACTAA
2151 CAGGATTGCA AACATGACGG ACCTTTACAA CGCTTTGCTA TATTCGTCCG
2201 ATCCGAAAAT TTCCGCCCTC TCAATGATAG ATAAACCTAA GAAACGTAAC
2251 TTTAGTAAAG CAGTAAAGAG CCTTTTGAAA GATGTTCCTG AGGCTGATGA
2301 TGATGATGAT GGTGATGNNN NNNNNNNNNN NNNNNNNNNN NNNNNNNNNN
2351 NNNNNNNNNN NNNNNNNNNN NNNNNNNNNN NNNNNNNNNN NNNNNNNNNN
2401 NNNNNNNNNN NCAACCTATG AACGTCTGGA GAAAAATACA TTCCCCTAAC
2451 TACTTCCCAG ATTTTTTTAG TCACCTGAAA GCTTATTTTG TGCAAGATGA

2501 AATCAATACA TTTAAATTAT TTTTACGCAA TTTCGTGTGA TTATTTTACC
2551 TTCATTTAGA TTTTAACGGC TCACTGTGAT AACTCCTTAG TTTCTGAGAT
2601 GACGTAATTT TTAAATTAGC TGAAAATATA TTGTTTTCAT TGTTCTCAAA
2651 AACGTATGCA ATCAAATCGA TCTCTGAGGC ATTTTGACAA GTTTTAGAAT
2701 GTAAGTTAGA CATGTGATCT TTTTGTCCAG AAGAAAATTT TGTTTCTAGT
2751 CTTCCTCTAT TTTTTATTTA ATTGCCAATT TGTATTTCTA TGTAGCTCAA
2801 ACATTTTTAA AATTATGCAT TTTGTCATTA GCGTTACTAA GATTTTGGAA
2851 GATCCGTTTT TTTATGTAGG TATGCCGTTG ACTCTTTTAT TATTCTACAT
2901 TTATTTCACC TATCTCATTA AATTTTGAAA CCAAGTGCCT ATTAATTACT
2951 TTTAAGGGTT CGTCAACTTT AAGTTGAACT TAATTGTCCC TTTTTATTAC
3001 ACGAATTCTT ACGTTGATCC ATTGTTAACG GTTTTTTGTT CCTTTCATAT
3051 CGATATTTTT GTGAAATGCA TCTCATTCCT CCACTATCAT TTTTTAATTT
3101 TTCCCCTTTC ATGCTCTCAC ACTCTCACGC ACTGTAGCCA TTTAACTAGT
3151 TTCCAGAATG TTTCAGACAT TTGGATGGCT GCAATCAATA ACATCATTGA
3201 TAGTTAAAAT TTGCCTCATT CATTAGAGAT CTACTAGTTC ATTGAAATTA
3251 TACAACCTTT TTTTTCTCCT GTAAAATTGT GAATTTTCAA TATTTTTCAG
3301 GTCTGATAAA AAGTGATTTT AATAATTGCC TAATTCTAGT TTTTTTCAAT
3351 AATGTTATAC CTAGTTTCAT TCTTACATCG AACTTGGCTA TTTGAAGTAA
3401 ATTTCATTTT GTTGTTATTT TTTGTTGGGA TTTTTTTATT TTCCTTTGTT
3451 TTTGTCTTTG ATTTATTTTC TTTAAATTTA CCAAGTTTTA TTGTTCCTAC
3501 GGAGTATGTT CCTTTTTTCC CCTTTAAATG TATGTTCCTT TTTTTCTTTA
3551 AATATCCTTT CTTTCGGTGA AAAAATGTGA TTCATTTGTT ATTATTGTAT
3601 TTCTGTGATT TTTGAATATC ATTCAGCCCT TTTGATTTGA AATTGTCGTC
3651 TACTTTAGTA GGAGTTTTGA AACAATTGTC TTCTTTTTTT CAAATTTTTT
3701 TTGGTATCGT CACAACTTAC TTCATTTGCT AAAAAAATCG TCACCTATTT
3751 AATACGGTGC TGCAATTGAG TGTTAAGTAC CTAAAACTAT CATGTGAGGT
3801 TCCTAATTTA TGTTGATTTC TTGACTTTTC CATGTTTGGA AGGTGCTTAC
3851 AGAAATGTCA TGTTGGTACT CTATTTTTTT TATTCTACGT ATTATCGGTT
3901 CGTTATATTT CAAGATTGTG GTTTTATAAT GTACTATTTC AAAGATTCCT
3951 GCCTTATGTC CATCCTCTTT TCGACGAATC AATAATTTCT CGGTTAAATT
4001 TTTTTGTTCC TAAAATTTTC ATTACCTCTT TTCTTATTGT TATTTAATGA
4051 TTTTTGTATT CTCTTAGGAA TTGATTATTT AATGCGTCAA CTTTTTTGTA
4101 TGAGAAGTTT TTCTTGGAGT ATTCATATGA TTACTCATAG TGCATTGAAG
4151 TAAAATAAGA AGAATGAAAA GGTTAAAACA AGACTGCCAG AATTTGTTTT
4201 TGAAAAATCT AAAATACACA TTGAACGCGG TATTTTTTTA TCCATGCGTA
4251 TTTAACGATC GATCCATCTT TATGTACCTG TTATGACCAC TTTGAAGTCA
4301 AACTCGATTC CTTAAAATTA AAATTTTCAT AGCCAATTTT AATTCAATGC
4351 ACAAATTAGG AAATACTGAC CTTTTGTACG ACAAATTTGT TTTCTCTGTT
4401 ATATTTCTTT TTGTGTGTTT TCAAATGTTC CTGCATTGTG ACATTATTAT
4451 TAAAGAATGA TTTTAATTTC TTGAATAGTA GGTCCAATTA TTTTCCTATC
4501 TACCGATTGC TTTGAAAGTA TCTAAATGAC CTAATAGTCG TAAGAATGCA
4551 AAATGTTCGT TGCAGCACTT CAAAAATGGT TTATTTCACT TTAAGTTTTC
4601 AGAAAAAACA TTAGATGCAT TGAGCGAAAG GTATTGCCAA AATTCCCGAG
4651 TATTAGAAAT ACTTCACTTT CCTTTCATGA CAATAAAGTG TGTTAATAAA

4701 CTAATGACAC GGATTTAAAC GATTAAATTT CCTGTCTGAC ATCACTTCAT
4751 GATAAATTAA ACGTTCGCAC TTTAAATGTT ATCTTAACAA GATTTTGAAA
4801 AAAAATAGAA CGCGTAAATA TCCATGGATT AGTTATAGCT ATTCCATTGT
4851 TCGACGGTTA ATTTTGAACT TTAAAATTTC AAGCATTTTT CTCCTCTTTG
4901 TGTACTGTTA TTATTAAATG CTTGCCAATA TTAATATGAC CTTTGTATCT
4951 ATCTACATCG ATGGTAATGA GCTGCGGATT ACCTACATTC ATCCTTTGTC
5001 AGAACCTCAT CTTATCCATG GATTACGCAT AACTGTTCCA CTGTGCGACG
5051 GTCATTTCTT GGGAAATTTA AAATTTCAAG C

The transcript and protein sequences of *CchTransib_2435* were predicted by AUGUSTUS.

MCIT02002435.1:1-5081 AUGUSTUS gene 1 2607 0.12 + . ID=g1
MCIT02002435.1:1-5081 AUGUSTUS transcript 1 2607 0.12 + . ID=g1.t1;Parent=g1
MCIT02002435.1:1-5081 AUGUSTUS intron 1 247 0.74 + . Parent=g1.t1
MCIT02002435.1:1-5081 AUGUSTUS intron 2312 2590 0.33 + . Parent=g1.t1
MCIT02002435.1:1-5081 AUGUSTUS CDS 248 2311 0.21 + 2 ID=g1.t1.cds;Parent=g1.t1
MCIT02002435.1:1-5081 AUGUSTUS CDS 2591 2604 0.34 + 2 ID=g1.t1.cds;Parent=g1.t1
MCIT02002435.1:1-5081 AUGUSTUS stop_codon 2605 2607 . + 0 Parent=g1.t1

coding sequence = [aacttgttgaaataaatcgcaaggatctctttgatgaatggaggaatcctaatgaactacaggacaaaaacaaagctgt
ctccacttttgtcctgaataagataagatctgatgcagaatctttaaattttgattcagcattggatgttctcactacagaagatgctgaggcaaatc
atgcaagtatcaaaaacttcaattcaaaactttctgccaagtggaaaaaacataaaaaaaatgagcgttttctcatgaatgaagaatcttggttggag
aagaatgaggtgttcatcgttgctagttgctcaccatctaagaaggaggttggcaaggaattaaaaactaaaatgggtcggccgtcgaaagaattttc
cgacctaagtgtggatgcaaaacgccggagaatttctagtcttatggaccaagcgactgcggaggagcttagttttgcggcctcttctagcttataca
ttgagggtaaaagagatgcggccagtctcgctcgtaaaatttctgaaactcctattgaaacagcgttgtcagtgaaagatacagagaatcaattaata
aagtcagagcttcatccctacactcctgatgaagctttggctttatttattgacgggcgattttcacgtgtcacttatgagcgagttcagcaatcagc
acgacgacacaacgcaaatatttacccaatttatgagcaagtaaaggtcgcaaaaaaaaggtgctacccttcaaatgagtcaattcatattaccgaaa
ccagtgccgaatctacgttacagggtatcgtggatcacacaaccagccgaatctttcagatgatcggatatgaaaatgaggcttcggacctcaaattg
ctgtacaaatggggtgcggatggtagctctggctactctatgtacaagcaaaagtggtccgaaaaaaaccaggatgcctccgattcttcgctgttttc
catttctttagtccctatacaactaagaaatagaagcagcgaagaaattttgtggacaaatcctcgtcctaattcggtccgattttgccggccaataa
aaattttacgcatctcagaaagtaaggagatcactcaagtcgaaatagggaaggttaaagaccagataaaagcgattaatcccaccagaataagcaga
ggcacagctcctgattgtgttgtttatcccgaatttttttgtaccatgatcgatggcaaaattttatgcgacttaacaaatacgtctacacaaagttg
ccctatttgcggcgctacgccaaaagatatgaatgacattgataaagtgatgaagaggccttgcaacaaaaaaaattatgagtacggattgtcgacgt
tgcacgcatatattcgatcccttgaatatatgctgcatgtagcttatcgcctaggactagagagagaaaataggaaatggcgaatttgtagtaaagaa
gataagttaattgtgagaaataagaaaaaaaacatacaatcagaaatcaaacaggagctgggcctcttgctcgatgtccctaaagtgaatagtgggaa
cactaatgatggtaactccgctaggagattttcaaaaatgcaaagagagtagcacagattacaggcttggatgaaaacatcctacatcggtttgaaa
ccatcctaattgtactttccagcggatttgccatcaatgtcgagtctttcagatcatttgctatcgaaactgcaaggctatgtgtcaaagtatacccg
tggtactatatgccccctgctgtccatcgtgtgctaattcacggagcagacataattcaagtagctcctgtcccgattgggctattaggtgaagatgc
cagcgagtctaaaaataaagatattaggagatttcaggtgcatagcacgagaaaaactaacaggattgcaaacatgacggacctttacaacgctttgc
tatattcgtccgatccgaaaatttccgccctctcaatgatagataaacctaagaaacgtaactttagtaaagcagtaaagagccttttgaaagatgtt
cctgaggctgatgatgatgatgatgtttctgagatgacgtaa]

protein sequence = [LVEINRKDLFDEWRNPNELQDKNKAVSTFVLNKIRSDAESLNFDSALDVLTTEDAEANHASIKNFNSKLSAKWKKHKK
NERFLMNEESWLEKNEVFIVASCSPSKKEVGKELKTKMGRPSKEFSDLSVDAKRRRISSLMDQATAEELSFAASSSLYIEGKRDAASLARKISETPIE
TALSVKDTENQLIKSELHPYTPDEALALFIDGRFSRVTYERVQQSARRHNANIYPIYEQVKVAKKRCYPSNESIHITETSAESTLQGIVDHTTSRIFQ
MIGYENEASDLKLLYKWGADGSSGYSMYKQKWSEKNQDASDSSLFSISLVPIQLRNRSSEEILWTNPRPNSVRFCRPIKILRISESKEITQVEIGKVK

DQIKAINPTRISRGTAPDCVVYPEFFCTMIDGKILCDLTNTSTQSCPICGATPKDMNDIDKVMKRPCNKKNYEYGLSTLHAYIRSLEYMLHVAYRLGL
ERENRKWRICSKEDKLIVRNKKKNIQSEIKQELGLLLDVPKVNSGNTNDGNSARRFFKNAKRVAQITGLDENILHRFETILIVLSSGFAINVESFRSF
AIETARLCVKVYPWYYMPPAVHRVLIHGADIIQVAPVPIGLLGEDASESKNKDIRRFQVHSTRKTNRIANMTDLYNALLYSSDPKISALSMIDKPKKR
NFSKAVKSLLKDVPEADDDDDDVSEMT]

No potential TIRs could be reliably identified surrounding the CchTransib_2435.

Hordeum vulgare

22. HovTransib_9086

>CAJV010199086.1:1-4539 Hordeum vulgare subsp. vulgare WGS project CAJV00000000 data, cultivar Barke, contig barke_contig_285576, whole genome shotgun sequence

>>>TSD + predicted 3'TIR

```
   1 CGCACAGTGG GCCAGAATTC AATTCTAAGT GGCCAAAAAT ATTTTTTTTC
  51 CTTATCACTT AACAACAGTA CTTTGAAACA TAGGACCATG ACCAAATTTG
 101 TTTTTAGTTA TAAATATTTA CATTTAACTT ATTTTTTATG AAGATTTTTT
 151 TCATTATAAA ATAAATTTTA ATAAATAATT TTAATTTTAT AATTTATAGG
 201 TGATTTTTCG TAATCATTTT TACTAATTTA TTTATATCCA ATATAACATA
 251 CAAAAAACAT AATACTTAAT ACTTTAAACA TAATACATAG GTCTTCACTA
 301 ATCAGAATTT GAAATTGTGT CATCTTCAGA TGACTGTATA TCTTGATTAT
 351 CCAGGTTCAA TAAATACAAT ACTTCTGGAG CAAATTTTTC TTTTAAATAT
 401 TTTTTTGGCG GGCTTAATGA TGTTATAAGT GGATCTGAAG ATATAAGTAG
 451 AAAGTGTATT AAGTCCGAAT TGGTATTTAT ACGGGAGGAT TTTCTAGAAT
 501 GATGTTCTCG ATATCTTTTG TAGTCCTTAT TTCGTGATTC TTGAGCTTCT
 551 TCTGACAGTT GTCCAATTGG AACAAGCGCA TATTTTATGG CTATAGAACC
 601 GTGAATTAAT ATTTTGTGGA CACTAGGAGG CATCCGGTAC CAGTTATATA
 651 AATGTACATA AAGTTTTGCG GTCTCTTTTG TATATTTATC GAATCTATCA
 701 AAATCAATGT CATGGCCACT AGAAATTGCT GTTAAAATAG TAGAAAATCT
 751 CTTTAATAAG TTAATATTTA CTCCAGTTAT TTCTGAAACC ATTTCGGGGT
 801 CTTTAAAAAA TCTTCTAGCA ACATTTCCGG TATTTGATGT TCCCATACCC
 851 TGAACTACTC TGTCTACTTT TAATCCCATC TCATTCCAAA GTCGATTTTG
 901 AATATCTTTT TTTCTATTGG CAACCAATAA TTTTTGTTCT TCAGTAAGTC
 951 TTTTCGTAGT TGGAGTTGAT AATTTGTATG ATATGTGTAA AGTACATTCT
1001 AGAAATTTAA TCCATGCATG AAGTGTAGAT AAACCGTATT TTAAATTTTC
1051 TATATTTACA TCAAAGTTAC ATAATTTGTC AAGATTGTTC ATTGCTGTAG
1101 GATTACATTT ACATATATAA CATTTTTGAC TCGATGTGTT TGTTAATACA
1151 TTAATAACTT TACCGTCAAT CATAGTCATA ATTAGAGTGT AATTTACAGT
1201 TAGTGACTTT TCCTTGCCAA TTACTATGTC TATGGTTTTC AAATTCTCAA
1251 TATCTTTTTC AACTTTTTGT ACTTCTGTTC TTGTTGTTTC TGGTGTCTCT
1301 TTTGTATATA TAAACTTAAT TGGTCTACAG TAGCGAGTGG ACGATGGTTT
1351 TGGATTTTCC CATAAAATTA TTTGTCTACC ATTATTTTTG TTAGTTGTCG
1401 TTAGTCGTAT AGGAACTAAA GAAAATAAAA AAACACTTGT TTCTGTAATT
1451 TCATTATGAT CGGTTTCATT GCTATCTGAA AAACGTTGGT GATATTCATT
1501 ATGGCCTGAG CTCCCATCAC AGCCCCACTT ACAAATAAAA TTAATTTTTC
1551 TATCGAGTTC ATCAAAGTTT TTATCGAAAT CGTAGTCTAA TGTTTGCATT
1601 AGTCTATTGA TTGTATGGTC TAATAGATTT TGAAGTGGAA CAGAAACACC
1651 AACTTCACTT ACTTCAATTC CATCAGGATA ACATCTTAAT TTAGCTGAAC
1701 GAACTTTATT GTATGATGGG TAAATATTAG CTCCCCGTTG TTTCGCCCCT
```

```
1751 AAACGTGTTA TATTATATGA ATCTTTAGTC ATTCGGGTAT TTATTAAAAA
1801 AGCGAGGGCT TCATCTTCCG AATACGGAGT TATATTATTT TTTTCAAGAG
1851 ACTTGTATAA TTTTCTTATT TTTATTGGAC GATTCGGAGA AAACTGTGTC
1901 ACATGCGTGA GCAAATCTGC AGCAGCATGT TTACCATCTT TGTATAAAGC
1951 AGACTTTGTG GCATAAACTA ATTCTTGAGA AGAATGGGAT TCCCTTAATT
2001 CTATTATAGC TCTTCTCTTA GAACGATCAC TTTTTTCAGA AAATAACTTT
2051 TTAGGTCTAC CTCGACTTGT TGTTGGTTGA TCTAATATTG TTTCTTCTTT
2101 ATTAGTCAAA TGAGGAATGT TAAATTCTAA TTTTAGCCAT TTTTGATTTT
2151 TATTTAAAAA TTTAGTGAAA TCTCGGTTTG AATCACGCCA TTTTTTGTTT
2201 GCGGAACTAC AATAACCCTT AACTCGGCTC ATCATTATAT TTTGGAATTC
2251 TTCGTTTTTT GAATCCCATT TTTCGCCTAT CTTGTTTTGT ATATACGTGC
2301 ATATAAGGTT TATTGTATCC ATTGAACATA TCTTATTAGT TAAACCGTGC
2351 AATTTTAAAA ATTCAAATAC TTCAATATTT TGAATATTAT TTTCGGTATT
2401 CATTTTTCTA AAAACAAATA TTCATAACTT TAAAAGGGGC AAAGGGGGGC
2451 AGACAGGACC AAAAATATTT TATAAAATAA AAGTTAAATT ATGAAATAGA
2501 GTACATGCCA AAGGTCTAAA AACCCCTAAA AATAATTTAT ATAATTTTAA
2551 AATATGATAA ACGTATTTTG TAATAAAAAT TACAATTATT TATTTATAAA
2601 ATACTTCTTA TATAAGTTTT AAAACATACC TGCAGTCCTT AGTCTAGTCT
2651 TCATAAAAGT AAAAGCAATT TGCAAAACTA AAATACCTAC GTACGATCAC
2701 GGTCGAGCTA CGTATTCATA CAGAAACGTT CAAATTCAAA CCTACTCGGC
2751 TTTCAATGGT ACAGCCAAAT CATAGATAAT GAAATATAAT CTATATACTT
2801 ATAATAGGAT TATAATGGGT TATATTGGCA GTTTACAAGC AGAAGTATAA
2851 ATAATGCTTA TCTACAACTA ATATTGAATG TAATCATATA AGTTTTATAT
2901 CTTATTGTGA GTATGAAAAA ACCCTATTTT TGACCGCTTA GAAATGATAT
2951 CTGGCACACT GTGAGCCGTA GCGCACGACA CAATTTTATT TTGACTATCA
                   <<<predicted TSD+5'TIR
3001 GGTAGATTTT TTTTTGATAA AATTAGACAA AAACAATTTT TTATGAATTT
3051 CTAACACAAA ATTATTTGCA CATTTTCGTG ATTTTTAAAA TTGCTAAAAT
3101 TCGAACCTTA AATGCGTTGA CGGAGTGTTT ATGTTGGGAT ATAATATGTT
3151 AACAGACATT TTTTATTAAA TGAATAATTA AAACTCAACG TCAATTATTT
3201 TAGACTTTGT ATTATTTAAT TATTAATTAT TATTGATATT TTATAGTAAA
3251 CGAGTTTAAA GAAAAATGGA AGAACCTCCG TTCCGTATTT GTCCATCATA
3301 TGAAACCCCC TGCCAGTGGT TCCAGTTCTA AAAGTAAAAT CCTTATTATT
3351 TGACAGAATT CGTGCAATTT GCTCTTCCAT TCATAAGAGC AGTGGGTACG
3401 CCAGTTGGAA ATTTATTTGA TTGCCCGCCA CAAAGTCCAA CTGAGCTAGC
3451 ACAAGATTCT GAAGATCAGA CCCAAAACGA AATATACAAT GACGAAGAAA
3501 ATGAAGATTT TCAACTATCA CATGTAGATA ACGATCTGTC ACCATCAAAC
3551 TGTCCTACCC AACTACCTTC ATATCCAATA CAAGAACAAC AAGACAATGT
3601 TTCTAATGAA AAATCGTTAA GTGACACATA TAAAATACCT CTAACTCCGA
3651 GGAAAGGAAA ATTTCAGAAG CGAGAAAATA CTGATGTGGA CCAATCCTTT
3701 ATGGAATACT TAAATATGAA ACAAAATAGA AACAACTCCT AATGAGTACA
3751 ACCAACAAAA AAAATGTTTT TTCTGAGTTT GCTACCCGAG ATTGAACCCA
3801 TGACAAACGC ACAAATGAGT TCACTTAGGC GTAGAGTTAT GCAGCTTATT
3851 GATGATATAA TGAATCCATT TGTACAAAAC ATGTTTAGTA GGTACTCTTT
```

3901 AATTTAATTT TTTTTCAAAA AAAAATGGCA ATGAAAACTG GCAATTCAAT
3951 GATGATACTA TCCCCCAGCA TATGTCTGGT GCTTTTTTTG TATGTGTAAG
4001 AAAATTTAAT TCGAAAACCT GCATGTTAGA GAGTCCGGCA AGTCAGTCAT
4051 TTTACCTGTC TAAGACAATA CAAAAATAAG CAATCCACCT ACGGCGGATA
4101 TGTGCCGTTG CATGTCCGGT GCTTTTTCTT AGGTAAAAAA TGTCAAAAAC
4151 CATGTTGGAG TATCTAGCAA GTCAGTCATC TCAAGTCAAC ATCGATTAAC
4201 TTCCAATAAG CCACCTTGGA CAATGTGCTA CAGGATATCA GGTAGGCAAA
4251 GCAGACCCGA CGTTGAGCAG CTTTAAATGA ACATGTTGAG AAATGTGCAA
4301 TTATAATTCC CCCAAAAAAA TAACATATAG ATACATAATG ATATAATATC
4351 CAATACAATA CATATAAACA TGTACCTGTA GTGTATATGT TACGGTGAAA
4401 GACGAAAATT GATAAATGTC GTCATTCCCC GGTCAATGAC GTCATTAACC
4451 AAAATGATTC AATTATGTAG GTAAATGTTG TAAAAACACA TATTTTAAAC
4501 TCAAATGTAC ATCTTTCACC GGTCATTGAC GTCATTTAC

The transcript and protein sequences of *HovTransib_9086* were predicted by AUGUSTUS.

CAJV010199086.1:1-4539 AUGUSTUS gene 298 2322 . - . ID=g1

CAJV010199086.1:1-4539 AUGUSTUS transcript 298 2322 0.68 - . ID=g1.t1;Parent=g1

CAJV010199086.1:1-4539 AUGUSTUS stop_codon 298 300 . - 0 Parent=g1.t1

CAJV010199086.1:1-4539 AUGUSTUS CDS 301 2322 0.68 - 0 ID=g1.t1.cds;Parent=g1.t1

CAJV010199086.1:1-4539 AUGUSTUS start_codon 2320 2322 . - 0 Parent=g1.t1

coding sequence = [atggatacaataaaccttatatgcacgtatatacaaaacaagataggcgaaaaatgggattcaaaaaacgaagaattcc
aaaatataatgatgagccgagttaagggttattgtagttccgcaaacaaaaaatggcgtgattcaaaccgagatttcactaaatttttaaataaaaat
caaaaatggctaaaattagaatttaacattcctcatttgactaataaagaagaaacaatattagatcaaccaacaacaagtcgaggtagacctaaaaa
gttattttctgaaaaaagtgatcgttctaagagaagagctataatagaattaagggaatcccattcttctcaagaattagtttatgccacaaagtctg
ctttatacaaagatggtaaacatgctgctgcagatttgctcacgcatgtgacacagttttctccgaatcgtccaataaaaataagaaaattatacaag
tctcttgaaaaaaataatataactccgtattcggaagatgaagccctcgctttttttaataaatacccgaatgactaaagattcatataatataacacg
tttaggggcgaaacaacggggagctaatatttacccatcatacaataaagttcgttcagctaaattaagatgttatcctgatggaattgaagtaagtg
aagttggtgtttctgttccacttcaaaatctattagaccatacaatcaatagactaatgcaaacattagactacgatttcgataaaaactttgatgaa
ctcgatagaaaaattaattttatttgtaagtggggctgtgatgggagctcaggccataatgaatatcaccaacgtttttcagatagcaatgaaaccga
tcataatgaaattacagaaacaagtgtttttttattttctttagttcctatacgactaacgacaactaacaaaaataatggtagacaaataattttat
gggaaaatccaaaaccatcgtccactcgctactgtagaccaattaagtttatatatacaaaagagacaccagaaacaacaagaacagaagtacaaaaa
gttgaaaaagatattgagaatttgaaaaccatagacatagtaattggcaaggaaaagtcactaactgtaaattacactctaattatgactatgattga
cggtaaagttattaatgtattaacaaacacatcgagtcaaaaatgttatatatgtaaatgtaatcctacagcaatgaacaatcttgacaaattatgta
actttgatgtaaatatagaaaatttaaaatacggtttatctacacttcatgcatggattaaatttctagaatgtactttacacatatcatacaaatta
tcaactccaactacgaaaagacttactgaagaacaaaaattattggttgccaatagaaaaaaagatattcaaaatcgactttggaatgagatgggatt
aaaagtagacagagtagttcagggtatgggaacatcaaataccggaaatgttgctagaagatttttaaagaccccgaaatggtttcagaaataactg
gagtaaatattaacttattaaagagattttctactattttaacagcaatttctagtggccatgacattgattttgatagattcgataaatatacaaaa
gagaccgcaaaactttatgtacatttatataactggtaccggatgcctcctagtgtccacaaaatattaattcacggttctatagccataaaatatgc
gcttgttccaattggacaactgtcagaagaagctcaagaatcacgaaataaggactacaaaagatatcgagaacatcattctagaaaatcctcccgta
taaataccaattcggacttaatacactttctacttatatcttcagatccacttataacatcattaagcccgccaaaaaaatatttaaaagaaaaattt
gctccagaagtattgtatttattgaacctggataatcaagatatacagtcatctgaagatgacacaatttcaaattctgattag]

protein sequence = [MDTINLICTYIQNKIGEKWDSKNEEFQNIMMSRVKGYCSSANKKWRDSNRDFTKFLNKNQKWLKLEFNIPHLTNKEET
ILDQPTTSRGRPKKLFSEKSDRSKRRAIIELRESHSSQELVYATKSALYKDGKHAAADLLTHVTQFSPNRPIKIRKLYKSLEKNNITPYSEDEALAFL
INTRMTKDSYNITRLGAKQRGANIYPSYNKVRSAKLRCYPDGIEVSEVGVSVPLQNLLDHTINRLMQTLDYDFDKNFDELDRKINFICKWGCDGSSGH

NEYHQRFSDSNETDHNEITETSVFLFSLVPIRLTTTNKNNGRQIILWENPKPSSTRYCRPIKFIYTKETPETTRTEVQKVEKDIENLKTIDIVIGKEK

SLTVNYTLIMTMIDGKVINVLTNTSSQKCYICKCNPTAMNNLDKLCNFDVNIENLKYGLSTLHAWIKFLECTLHISYKLSTPTTKRLTEEQKLLVANR

KKDIQNRLWNEMGLKVDRVVQGMGTSNTGNVARRFFKDPEMVSEITGVNINLLKRFSTILTAISSGHDIDFDRFDKYTKETAKLYVHLYNWYRMPPSV

HKILIHGSIAIKYALVPIGQLSEEAQESRNKDYKRYREHHSRKSSRINTNSDLIHFLLISSDPLITSLSPPKKYLKEKFAPEVLYLLNLDNQDIQSSE

DDTISNSD]

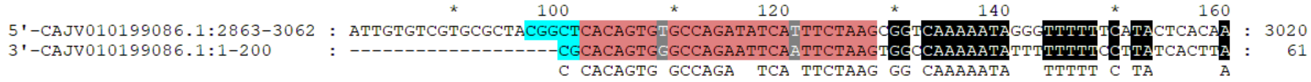

Figure 15 Alignment of 5’-and 3’-flanking sequences of HovTransib_9086 shows its potential TIRs

Corrigiola litoralis

23. CliTransib_2264

```
>CAAKMY010062264.1:1-3168 Corrigiola litoralis genome assembly,
contig: contig_39194, whole genome shotgun sequence
   1 GTGATTTTTT TAATTCTCAG TCCACATGTT CTTGCCTTAG TTGAAGCGTT
  51 GGTTTTTTCA CTTTTTAAAA ATTAACTTAG GCCTAAAATT TCAAACTGAA
 101 TGAATTTGTG ATTTATTTTC AGGTGCTATT ATTCCGTTGC AATCACTTTT
 151 GGATAACACC GCGATGAGAA TTGCTGAGAA TGTGCAATAT AATTTGCAAG
 201 ATGATGTTGA TCATCTTACT TTGATTTGTA AATACGGCTG TGATGGCAGT
 251 GGTGGGCACT CACAATATAT GCAGAAGCCT CCTCAAGAAG ACATTGAAGA
 301 ATTCAGCGAA GACGAAATGA TTGAAAGTGA AAGTGAGGAA AATAGGCAGG
 351 AAGTTGACGA TACCTGTTTA CTACTCATGT CTATGGCTCC ACTATGTCTG
 401 AAAACAAACC TTGGAAACCT GATCTGGGAA AATAAAACTC CCAACTCTCC
 451 TCGATTTTGT CGGCCTATTG GATTTGTCTT GAAGAAAGAA ACGAGTCACC
 501 TGGTTCTTTC AAAGATGGGC AGGCTGAAAA GTGAAATAAG GGCCCTCCAG
 551 GAGGTAATTC TCAGTTTCAA CGATAAAATG GTAGCCTGTT CATTCGAGTT
 601 GCATTGTACC ATGTTAGATG TTAAAACCAT CAACACTCTA ACAGGTAATG
 651 CTTCCACTCA GACCTGCTAC CTTTGCCACA GCAAGCCAAC CGAGATGAAC
 701 CAATTACTGT CTGCCAAACA TCAATCCATC GAAGAAAATA ATCTACAATA
 751 CGGTTTATCA TCTTTACACG GCTGGGTGAA GATGATGGAA TACATCCTTC
 801 ATATTTCGTA TATGCTGGAA GTGAAGCAGC CAACTATTCG GGGACTTGAT
 851 GCGAAGCAAA GGGCAGAACT GGCTGAAAGA AAATCACTAA TACAGAAGAA
 901 ATTGGCTGCA CTTGGAATGC CGATCGACAG GGTGGTACTG GGAAAAGGTA
 951 CCAGCAATAC GGGCAATGTG GCCCGTAAAT TTTTGCGACA TTATTCCGAA
1001 GTTTCCAAAA TCACAGGTGT TGATCAGAAC TGTATCCAGC GTCTTTATTT
1051 CATCATGGTG GCCCTGACCT GTAGCAAGCC ACTCAACATT CCAGCTTTTC
1101 AAATTTTCTG CGAAGAGACA GCTAAGAAGT TTGTGGACCT ATACCACTGG
1151 TATAGAATGC CAGTGACCGT CCACAAACTT CTGGTACATG GAGCTCAGGT
1201 GGCTCAATTC ATGCCACTTC CAATAGGCAT GTTATCTGAG GAGGCTTCTG
1251 AGGCGGCTAA TAAAATCTAC AGGGCTGTCC GCGAACATCA CACCCGAAAA
1301 GCAGCTAGAG AACAGACAAT TTTGGATCTC ATCTGCTATA TGTTAGCTTT
1351 CAGTGATCCA AAATTGTCTG AACTCAGAAG ACCGGCCAGA GAAACTCTGC
1401 ATCTTCCAGA GGAGGTGCTA CCTCTTTTGC TCCATTATGA CGAAGTTCCT
1451 TCACATTCTG ATAATGGGGA TTCTGAAGCA GTGATCGAAG TAGAATGTAT
1501 ATGCGACATT GACAATTTCT GTGATTAAAT TTGATAATTA TGGTGTTTTC
1551 ACTTCATAAT ACTTTTGATT TTGTGTTCGA ATTAAATTTG TCTTTAATTG
1601 TGAGTTTTAA TGAAGTCTTT TCTTTAGAAA CTTATAAAAA CCCTGATTAA
1651 AACTTGAATG AAAGAAAGAT ACAAGCTATG CCTACATGAG ATTTTTTGAA
1701 GCTGGTGCTG GTCATTGCAA ATATTGTGAA CTGAAAGAAA TTTGTTTGCC
1751 ACCAGTTTTT GTTTCAATTT TCTAAATAAT ATTCCATAGG AAAGGAAATT
1801 GATTCACTTT TCAACCTATA TTTTCTAATA ATTCTGCTCC CAATTATTCA
```

1851 CTTCCCGTTT TTAGTCTCAA TGAGTTCCCC CAGACTTTGC TCTTCAATCC
1901 ATTCGTTTGA ATCAACCAAA TCTATAAAAA AAAATTGACT GAGTTATGTG
1951 CGCTGATTGA GAAACTGTTG ATTATTTAAT TTGCACCAAA ATTCAATTGT
2001 GAACAATGTT TTTAATGAAT GCCTAGTTTG TCTAGGACCA ATAATTATCT
2051 ATATACACAT CTACAAGCAG TCATCTATCA CCATACGTTT TTTGGATATC
2101 CATCAATGAA TCACCCTGTA TATTACTTGC GTGAGGACCG AAAGGACACC
2151 CTGTATGTAC GGATAAACTA TTGGAGAGAG AAAAAATAGT CCTGCATGAA
2201 ATTCAATGCA AAAGTGTGTC TTGGAATGCG AAAGAAATGG ACGAAACTAT
2251 AAATGGGGAT TTTGGCCGCC TAAAATCACG ATTTCGCCCA GTGTGCGGCG
2301 TATACATTTA AACTCTACAA ATTTCTTATA TGACCCAAAA TTTTGGGTTT
2351 ATTTTGATTT CAAGGAGTAA CTGTGCCAAA GTGAGAAAAT TAAATTGAAA
2401 AATTTTGCTC AGAAAGCTCT CCTTTTTTGT ACGTTTTAAA ATTCAGTGTA
2451 TTAAATTTGA AATAAATTTA ATTCTTCTTT CTGTCATCCC TTATGAAAGA
2501 GAAGAAAGAA AATGTTGAGT TTATTTTGCA TCGAGGCTAT CATTTAAAAT
2551 ACCATCTCAC CTCTTTTTGG CTAAATATTA CACTTCAATA ATTGATGAGT
2601 AGCATTCTTG CATTATAATG CACCTAGTTT TAATATAATA AACTGCAAGA
2651 ATAAAATTTT CAACTTTTTC TCACTTATAG AAAAAAATCA GGCAGAGTAT
2701 ACTCTCAAGA AATTTTTCGG GGACACCACT GTTCTAATGT CCATATATAA
2751 ATCGCAGTGT GAAAACAAAT TTTTCTGATG ATACATGGAA AAAAAAATCA
2801 ATTCATTCGA CAGAGAAGTT TTGTTGTAAT AGACGATACT AACTTAGAGC
2851 TCGAGATTCG CCTCAAATAA ATGAACTGGT AATTTCAGAA AGGGACTCAT
2901 TCCACTTTCT TGAGGGATAT GAGTTGAAGA AAGTAGTAAT ACTATTTGAG
2951 GGTACGACTA CTCTACAGTA GTTTTATTGG AGATTCAAAT TTATGTTTAC
3001 TGCAGAATAT CAATGAAAAT CTATCTCCTC ATTGCCCTCA TCTATGGAAA
3051 ATTTATATGT TTGTGTCGCC ATTTATTTCC TTTAAAAAAA AGGAATTCAC
3101 TCTTGTCCTG CTCAGAGAGG TCTAATGTTC AATTTCATTG AGGGGGATCA
3151 TGGTAGGGTA CCCACTAG

The transcript and protein sequences of *CliTransib_2264* were predicted by AUGUSTUS.

CAAKMY010062264.1:1-3168 AUGUSTUS gene 1 3168 0.8 + . ID=g1

CAAKMY010062264.1:1-3168 AUGUSTUS transcript 1 3168 0.53 + . ID=g1.t1;Parent=g1

CAAKMY010062264.1:1-3168 AUGUSTUS intron 1 122 1 + . Parent=g1.t1

CAAKMY010062264.1:1-3168 AUGUSTUS intron 1490 3168 0.55 + . Parent=g1.t1

CAAKMY010062264.1:1-3168 AUGUSTUS CDS 123 1489 0.56 + 2 ID=g1.t1.cds;Parent=g1.t1

coding sequence = [gtgctattattccgttgcaatcacttttggataacaccgcgatgagaattgctgagaatgtgcaatataatttgcaaga
tgatgttgatcatcttactttgatttgtaaatacggctgtgatggcagtggtgggcactcacaatatatgcagaagcctcctcaagaagacattgaag
aattcagcgaagacgaaatgattgaaagtgaaagtgaggaaaataggcaggaagttgacgatacctgtttactactcatgtctatggctccactatgt
ctgaaaacaaaccttggaaacctgatctgggaaaataaaactcccaactctcctcgattttgtcggcctattggatttgtcttgaagaaagaaacgag
tcacctggttctttcaaagatgggcaggctgaaaagtgaaataagggccctccaggaggtaattctcagtttcaacgataaaatggtagcctgttcat
tcgagttgcattgtaccatgttagatgttaaaaccatcaacactctaacaggtaatgcttccactcagacctgctacctttgccacagcaagccaacc
gagatgaaccaattactgtctgccaaacatcaatccatcgaagaaaataatctacaatacggtttatcatctttacacggctgggtgaagatgatgga
atacatccttcatatttcgtatatgctggaagtgaagcagccaactattcggggacttgatgcgaagcaaagggcagaactggctgaaagaaaatcac
taatacagaagaaattggctgcacttggaatgccgatcgacagggtggtactgggaaaaggtaccagcaatacgggcaatgtggcccgtaaattttg
cgacattattccgaagtttccaaaatcacaggtgttgatcagaactgtatccagcgtctttatttcatcatggtggccctgacctgtagcaagccact

caacattccagcttttcaaattttctgcgaagagacagctaagaagtttgtggacctataccactggtatagaatgccagtgaccgtccacaaacttc
tggtacatggagctcaggtggctcaattcatgccacttccaataggcatgttatctgaggaggcttctgaggcggctaataaaatctacagggctgtc
cgcgaacatcacacccgaaaagcagctagagaacagacaattttggatctcatctgctatatgttagctttcagtgatccaaaattgtctgaactcag
aagaccggccagagaaactctgcatcttccagaggaggtgctacctcttttgctccattatgacgaagttccttcacattctgataatggggattctg
aagcagtgatcgaa]

protein sequence = [AIIPLQSLLDNTAMRIAENVQYNLQDDVDHLTLICKYGCDGSGGHSQYMQKPPQEDIEEFSEDEMIESESEENRQEVD
DTCLLLMSMAPLCLKTNLGNLIWENKTPNSPRFCRPIGFVLKKETSHLVLSKMGRLKSEIRALQEVILSFNDKMVACSFELHCTMLDVKTINTLTGNA
STQTCYLCHSKPTEMNQLLSAKHQSIEENNLQYGLSSLHGWVKMMEYILHISYMLEVKQPTIRGLDAKQRAELAERKSLIQKKLAALGMPIDRVVLGK
GTSNTGNVARKFLRHYSEVSKITGVDQNCIQRLYFIMVALTCSKPLNIPAFQIFCEETAKKFVDLYHWYRMPVTVHKLLVHGAQVAQFMPLPIGMLSE
EASEAANKIYRAVREHHTRKAAREQTILDLICYMLAFSDPKLSELRRPARETLHLPEEVLPLLLHYDEVPSHSDNGDSEAVIE]

No potential TIRs could be reliably identified surrounding the CliTransib_2264.

Juglans sigillata

24. JsiTransib_2954

>QEOY01132954.1:1-3127 Juglans sigillata isolate DJUG951.04 C633597, whole genome shotgun sequence

```
   1 GCACAGTGGG CGGAACACCC GAAAAAGCAA GAAAAATTCG GAATTGACCG
  51 TGTGAGTACA TTTTTCGACT CCTCTTGACG AGGCAAATCG AAAGTGTTCT
 101 TACATGTTTT CCGATCAGAG TCGAAATAAC AAATTTATTA GCGATTATTA
 151 GCATTTAAGG TCTTAGAAAT TGTTAAGATA CCTATAAAAT CCTCTAATTC
 201 ATCATAATTT CTTGATTTTG ACCTCGCTTT CATGTAAAAG ATCAGTTTTT
 251 ATTGCCAAGG TGAAGAAACA CACAACATTG GGCACAGTAA AATTATTTTT
 301 ATTGGATCAA CTAGCTCCAA GGAGCAATGA TATAAAATTA TGAGGGTTCA
 351 TCGATTTCAC TGTCAGATTC TTCAATGATT TCCATGGGCA AAGGCTCATT
 401 TGAAGTTGGA TCACTAGCTC CTAGGAGCAT CTCAGCAACC CTTCTGTCAA
 451 GGCAACCTTT CGGCTTAGGA GTTCTCCGCA GAGAAGCAAT TAAGGGATCC
 501 GTTTGGACAA GGAGTCCCTT GTAAAGGTCA GTCATAGTTG CCTCTCGACT
 551 GGATTTTCTT GTATGGTGCA GTCGCACGTA CCGGATTATC TTATGGTCAC
 601 TCTCAAGAGG CTCTTCTGCA AATTAAATAT TTTCTTTTCA AACTGATATT
 651 ACGTTAAGTA GGCAATGGAT GTTAGAAAAT TATTTACCTG ACAGCATTCC
 701 AATTGGAACA GGAAGTATCT CAATCATAGC TGCTCCATGC ATGAGGGCAA
 751 GATGTATAGC TGATGGCATA TACCAGAATC CATATAAACG TACTAGGTCC
 801 CTTCCTAATT GGATACAATA AGCACCGAAG ACCTGTGTAT TGATGGCATA
 851 CCCACATGAG TAAGTTTTTA GGACGACTTG AATTTTTTTC AGCACATCTA
 901 AGTCAAGTCC AGTGATAGAT GCAACCTAAA ACAGAGATAA ACAGAGGTAA
 951 ATTATAAAAA TTGTACTATT GCATCATTTT GAATGACAAC ACATTCCAGA
1001 ACTCACATCT AATGGCTTAT CAAAGAAACG GCGAGCGACA TTGCCTGTGT
1051 TGGTGTTTCC ACCCTTGCTT GTAGGTTTAT CAACAATCAA GCCCAACTCT
1101 GCTTTAAGCC TTTTACAAAT CTTCGAGTGT CTTTCTTTCA TCTCTGCAGT
1151 TTCCTTTTCT GAGTATTTTC CATAACACTT CTCTGCAACT TCAAGTCTGA
1201 TCGCTATATT CAAACAATAC TCTAAAAATC TAATATGCAA GTGAAGAACA
1251 GACAACCCGT ATTGAAGTGC TGTGGCATCA CATTTCCTTT TTAAAACTTC
1301 ATCAATATTG TTCATTTGGA CTGGAGTCGC CTTGCAAACA TTACATGTGG
1351 CGCTGCCTGT CTTAGTCATC GCACTTGAAA CTTTACCATC CACCATGGTG
1401 AACTGAATTC GATGAGAGAT TTCATAGACA ACTCCATCAA CAGTAACTAT
1451 AGAAACTTCT AAATTATTTA TTGCATCTTG AAGTTCTGAA GCCTTCAACT
1501 TAATGAAATT TGTTGTTTCT TTGGTAAATT CTAGCTCAAG TGGGCGACAA
1551 TGAACAATCG ATGAAGGTTT TTGATTTATC CAAAGAACTT TACCTGTAAA
1601 CCAAAAAAAA AAAATTATTT GCAAACAGAA AAAAATTAAT CAATGAGGGA
1651 TAGGGAAGAA AGGAACAAAA ATCAAAATAA ACATACTTGA ATCATCAATT
1701 CTTTCATGCA GTAACAGAGG GACAAGTGCA GTTAACACCA TTTGACTATC
1751 ATCCGCTGGT CCATCCTTCC ATAGTTGATG ATACAAGGAT TGATTTGATG
1801 ACCCGTCAAA TCCAATTTTA GAAACAAGTT CTAGTTTATT TCCTTCAACA
```

1851 TGACCACTTT TAAAAACTTC AGCCTGGGCC TCCACTATTC TTTTTGCAGT
1901 GTGGTTGAGG AGGTCTTGCA GACTAACACT TGCCTTTCTT TCAGTAATTT
1951 TCAAATTATT GGGGAGGCAT TTTTTCTGAA AGAAGTAAGC TTGATTCAGT
2001 AACAAAAATT TTGGTCTGTA TCAGGTTAGG AAATAAAAAT GTTTGCTCTT
2051 CATACCTTAT ACTCTCTTAT CTTCTCATAG CTGGGAAATA CTGGGCAATT
2101 TTTAGAGAGA AGCCTAGTTC TATTTAAAAT ATACTGCTCC TTCGTGAAGT
2151 CACACTCAAT CTGGTGGACT ACTGCTTCCT CTAGACTAAA CGGCTTTGGG
2201 GACCCATCAT CTAGATTACC ACTCTCGGTC CTCTTTATTT TACCTATTAC
2251 TAAGCAAATC ATGCTCACTG TTCGAGGACT TGCTTCAGTA ACTTTCTTCA
2301 CCACTGTTGC CTCTTTTTTC TTCCCTGCAC CTCGCAATTT CATTTGAGCT
2351 GCATATGTAA CTTCATCTAC ATCCTTCTCA TCTCTCAAAG ATTTTGTCCT
2401 TTTCCGTTTA CTATCCTCAC TCAAGTCATT GAAGTCTTCC TCCTAAAACA
2451 CGAATCAAAA TTGTAAACAC TGCAATATGA TCAAACTAGA GAATTTATTA
2501 ATTAGCCTCA CCTTAGGGCG CCCTCTCTTG CTGCTGTCAC CACTTGTACT
2551 AGTACTTGGC TGAGGCTCAG TTTCAGTAAA ACATTCAGCT CCAGCAAGCC
2601 AGGTTTCATT ATCATGTTGA AAACGGTCTG TGTGGTATGC CGCTTTCCTC
2651 CATCTAGTGT TCACTTTATC CACGAATCGT CTCAGTACTA CTAAGGCTGT
2701 CGTTTTCCAC TCTGGAGCAA TATCAGACAA ACCCAGTTTT CCTGAAACCA
2751 ATAGCAAGTT GGGAAAGGAC AGTCATAACC CTACAAACAA TGATGCGACT
2801 TCAAAAATGC TCACCTGCCA CAAAGTCCAC GAATAAGTCC ATACATTGAT
2851 CACGTCCTTT AATCTTAACA TATTCATTCC ACAACTCAAT ACGACCGATA
2901 CTTTGACTTG CCATCTTGAT AGGACAAGAT TCCTTTAAAT TGCACACGCA
2951 ACATCGTTTT GGACTTCGTA AACAAACCAC CGGCTCTGGC GGTGCAGCCC
3001 GCAGCGCCCC TGGCGGCGGG ATCACGAACC GCCCGTCCAC CCGTCTCGGC
3051 AGCCGAGCTG AGAGCGGCTG AGGGTGTCCA ACCGCACTTA CCGAGGTACT
3101 TAGTTCAAAA GTGCGGACGA GTGCGGA

The transcript and protein sequences of *JsiTransib_2954* were predicted by AUGUSTUS.

QEOY01132954.1:1-3127 AUGUSTUS transcript 338 2914 0.01 - . ID=g1.t9;Parent=g1
QEOY01132954.1:1-3127 AUGUSTUS CDS 338 615 0.98 - 2 ID=g1.t9.cds;Parent=g1.t9
QEOY01132954.1:1-3127 AUGUSTUS CDS 688 925 0.96 - 0 ID=g1.t9.cds;Parent=g1.t9
QEOY01132954.1:1-3127 AUGUSTUS CDS 1007 1593 0.9 - 2 ID=g1.t9.cds;Parent=g1.t9
QEOY01132954.1:1-3127 AUGUSTUS CDS 1687 1975 0.87 - 0 ID=g1.t9.cds;Parent=g1.t9
QEOY01132954.1:1-3127 AUGUSTUS CDS 2251 2442 0.12 - 0 ID=g1.t9.cds;Parent=g1.t9
QEOY01132954.1:1-3127 AUGUSTUS CDS 2512 2741 0.99 - 2 ID=g1.t9.cds;Parent=g1.t9
QEOY01132954.1:1-3127 AUGUSTUS CDS 2815 2914 0.18 - 0 ID=g1.t9.cds;Parent=g1.t9
coding sequence = [atggcaagtcaaagtatcggtcgtattgagttgtggaatgaatatgttaagattaaaggacgtgatcaatgtatggact
tattcgtggactttgtggcaggaaaactgggtttgtctgatattgctccagagtggaaaacgacagccttagtagtactgagacgattcgtggataaa
gtgaacactagatggaggaaagcggcataccacacagaccgttttcaacatgataatgaaacctggcttgctggagctgaatgttttactgaaactga
gcctcagccaagtactagtacaagtggtgacagcagcaagagagggcgccctaaggaggaagacttcaatgacttgagtgaggatagtaaacggaaaa
ggacaaaatctttgagagatgagaaggatgtagatgaagttacatatgcagctcaaatgaaattgcgaggtgcagggaagaaaaaagaggcaacagtg
gtgaagaaagttactgaagcaagtcctcgaacagtgagcatgatttgcttaaaaaaatgcctccccaataatttgaaaattactgaaagaaaggcaag
tgttagtctgcaagacctcctcaaccacactgcaaaaagaatagtggaggcccaggctgaagtttttaaaagtggtcatgttgaaggaaataaactag

aacttgtttctaaaattggatttgacgggtcatcaaatcaatccttgtatcatcaactatggaaggatggaccagcggatgatagtcaaatggtgtta
actgcacttgtccctctgttactgcatgaaagaattgatgattcaagtaaagttctttggataaatcaaaaccttcatcgattgttcattgtcgccc
acttgagctagaatttaccaaagaaacaacaaatttcattaagttgaaggcttcagaacttcaagatgcaataaataatttagaagtttctatagtta
ctgttgatggagttgtctatgaaatctctcatcgaattcagttcaccatggtggatggtaaagtttcaagtgcgatgactaagacaggcagcgccaca
tgtaatgtttgcaaggcgactccagtccaaatgaacaatattgatgaagttttaaaaaggaaatgtgatgccacagcacttcaatacgggttgtctgt
tcttcacttgcatattagattttagagtattgtttgaatatagcgatcagacttgaagttgcagagaagtgttatggaaaatactcagaaaaggaaa
ctgcagagatgaaagaaagacactcgaagatttgtaaaaggcttaaagcagagttgggcttgattgttgataaacctacaagcaagggtggaaacacc
aacacaggcaatgtcgctcgccgtttctttgataagccattagatgttgcatctatcactggacttgacttagatgtgctgaaaaaaattcaagtcgt
cctaaaaacttactcatgtgggtatgccatcaatacacaggtcttcggtgcttattgtatccaattaggaagggacctagtacgtttatatggattct
ggtatatgccatcagctatacatcttgccctcatgcatggagcagctatgattgagatacttcctgttccaattggaatgctgtcagaagagcctctt
gagagtgaccataagataatccggtacgtgcgactgcaccatacaagaaaatccagtcgagaggcaactatgactgacctttacaagggactccttgt
ccaaacggatcccttaattgcttctctgcggagaactcctaagccgaaaggttgccttgacagaagggttgctgagatgctcctaggagctagtgatc
caacttcaaatgagcctttgcccatggaaatcattgaagaatctgacagtgaaatcgatgaaccctcataa]
protein sequence = [MASQSIGRIELWNEYVKIKGRDQCMDLFVDFVAGKLGLSDIAPEWKTTALVVLRRFVDKVNTRWRKAAYHTDRFQHDN
ETWLAGAECFTETEPQPSTSTSGDSSKRGRPKEEDFNDLSEDSKRKRTKSLRDEKDVDEVTYAAQMKLRGAGKKKEATVVKKVTEASPRTVSMICLKK
CLPNNLKITERKASVSLQDLLNHTAKRIVEAQAEVFKSGHVEGNKLELVSKIGFDGSSNQSLYHQLWKDGPADDSQMVLTALVPLLLHERIDDSSKVL
WINQKPSSIVHCRPLELEFTKETTNFIKLKASELQDAINNLEVSIVTVDGVVYEISHRIQFTMVDGKVSSAMTKTGSATCNVCKATPVQMNNIDEVLK
RKCDATALQYGLSVLHLHIRFLEYCLNIAIRLEVAEKCYGKYSEKETAEMKERHSKICKRLKAELGLIVDKPTSKGGNTNTGNVARRFFDKPLDVASI
TGLDLDVLKKIQVVLKTYSCGYAINTQVFGAYCIQLGRDLVRLYGFWYMPSAIHLALMHGAAMIEILPVPIGMLSEEPLESDHKIIRYVRLHHTRKSS
REATMTDLYKGLLVQTDPLIASLRRTPKPKGCLDRRVAEMLLGASDPTSNEPLPMEIIEESDSEIDEPS]

No potential TIRs could be reliably identified surrounding the JliTransib_2954.

25. JsiTransib_3411

>QEOY01133411.1:1-5769 Juglans sigillata isolate DJUG951.04 C636353, whole genome shotgun sequence

```
  1 TTTTACGCGG ACTGCACCAC GAAATTCGGT TGAATCTCGC AAAAAATCCG
 51 TCAAGTTGCT GCTATTGCTG GCAGCGTTTC GAACACGTTC GACAATTTCA
101 ATGAGCTCGT CCGCCGTCTC ATGCTTGCTG ATATGAATTG ATTTTTCTTT
151 GTGTTGAGAG TGCATCTGAA ACAAAGGAAA AGCAACTTTA GTAATGTGGT
201 TGGCAGTGTC TGTCAATGAA GCTACAATTC CTTTAAGTTT TGGCAGATAC
251 TTATCTTTTT CGTGCCTAGG GCTCCACTTT TTTGTATGAT TTAAGTTGTG
301 AACTTCCACA TGACTTTAAT TCTTTCGAAA TTTCGCTCTG GATTTCTTCT
351 TTTTCGTTAC GTGTGATTTC ACTTCTTAAG CTTTTGTTAG AAACTTAGAT
401 CAGCTTGGTT GCATGGCAGT GGGGAGTTTT TTTCGAAAGG ACGGGTTGCC
451 CTNTTTCCAC TTAAGTGGTT TTTTTTTCTG ATCTCTTGAA AGTCGGGCAA
501 CTTAAGTGGT TTTTTTTTCT GATCTCTTGA AAGTCGGGCA GTTTGTGTGT
551 CTTGGGAAAT GTAGTTTTCC TGATGCAGGA GAGATGCACT GCGGGCTTGA
601 ATGCGGGTCA CGTCGTTAAT GTGCTCATAT TTGTTGCTTG AATTGCGATT
651 GCAAGAGCGT TTGCGCATTA CTTTTTCCTG AAGGTCCCGA AAGTGTAGAA
701 ACAATGAGCA TTCATTGTTT CTGGAAGAGT TACTTGCGAT AGTTGTTGGT
751 TCGTAACACG GAATGATTGT CTTGAGATTC TTGCGGTCGA CGGAGAAAAT
```

801 TCCTGATACA GTAGCAGCTC GACGGATCCT AGGTAATCGT GGCCCTTGGC
851 GCATCATCGA ACATATAAAC AATTTTCTTG CTAATTAGGA GCAGCTACGA
901 AGTTTTCAAA TAAATTTGTA CAGAGTTGCA TTCGTAATGC GAATTGAGAA
951 CAGTTAATAC TACATAATAC TTCATTTTGA TAGATGTTTG ACTTAGTTGA
1001 CCTTGAGAAC CTGGGGAAGG AAATATTAAA CTCGTTAACA TCATCTCTGT
1051 ATGCATCAAA TCTCCGTATT CAAGAAACGG AAATAACAAA CCTGCGATCA
1101 ACGAAGTAAT TGATTTGCTA TTAATTAAAT CATAATAAGA AATCTTAAAT
1151 TCCATAAATT GATCTTCCGA AACAAATGTA AAGTTAGTGC GTCTTTTGCG
1201 TCCATAAATT TCATTCATTC ATTATGCATA TTTCATAATA TTCATGATAT
1251 TCAGTATTGT TCATAAATTT CATCTCGTTG AAATAAAATA ACTAATTAAA
1301 CTTGGGCTTT CTTGATAGCC CACGACAAAA TTGAAAACTA AAGTTTAGAC
1351 AAGGAGAGAG TTAACCTCGT CATCAACTGC TCTTAGCTTC TTTGGGATAG
1401 ATCTGCAAAA GAATGACACC GCTTTATAAT GGAAAGGAAC GACTTTTAAA
1451 AATGCGTTGT TTGAGAACGA TACTTACGTC AGAGAACTTA TGTAGGGATC
1501 GGATCTGACA AGCAGACGCT TGAAGATGTC CTCATTCGTG AATTCCCTAC
1551 TGCACTGGCG GGCGTATGAG GACCTGTACA GTCTAAGAAG CTTGTTGATA
1601 GATTCCTGCG CATCCTCTGA CATAAGACCA AGTGGGAGAG GAGACCTTTC
1651 AGCTATTTGG GCTCCGTGGC CCAGGACTCT ATGCACTGTT GGTGACATCT
1701 TTGCCCAGGG ATACAGGTCT CTGTACAATT GTCCTGTCCC TATAGCGTAT
1751 GCATTGTATT TGGAAGAGTT GATCTCTCTT CCGGACGCGA TAGCCTTTAG
1801 GACGACAGCA AATCGTCTGA TAAGCTGCGG GTCAAGTTCC ATGACCGCTG
1851 CGGCCTTTTC CGGATTGTCA AATACCTTAC GTGCTGTGTT CCCTAAGTAA
1901 ACGATGTGAA TTGACAATAG GATCAATAAT GTAAAGATAC TCGCGTAATG
1951 AACGATTGAA TTGCATAGAG GAGAGGAAAA CCATAAACCA ACCGTCCGTT
2001 GTGTTTCCAC CTGCTATGGG AATATCAACG AGAAGGTTCA TCTCTTTCTT
2051 AAACCTATCC TGAATAAGGA TTTTCCTAGC CTTAATCGTT GTGAGTTCAC
2101 CGGGGTCTTT GGTGCTCCAT TGTTGGATAT CTTGATGACA AGCTATTTTG
2151 AATAGGTTTA AAAAGCAGTT GATGGTTGCA TGTAAAGGGG ACATACCAAA
2201 ATCAGCCGCA TCTGTTTGCA ATGGCCTCTC TAGGCAAGCT TCTATATCAT
2251 TGAGCTGATT TGATGGGGCC TTGCACAAAG GGCATGCATT GCTGCAGCAC
2301 CTTAAAATCA AGTTGCCTAC TTTACCATCG ATCATCGTCA TGACAAAATT
2351 AGAAGAAACC GATACCACTT TTCCAGTTTT CAGAGTCAAC TCCAGTGGCT
2401 GCAGCAACTG GATTTCAGTT TTCATTTGTT GATGGATATC CCGAACAAAG
2451 TCCCCGGTTT CCTTGCTTCC AAATATTAAC TTAATAGGAC GGCAATATCT
2501 TGTTGAAGAG GGCGTTAAAT TTTGCCATAA GACTTTCTTT GACTCAGCTT
2551 CTCTCAACAT TAGGGGGACG CAGGTGACTA GGAAGCAGTT GCTATCCGAA
2601 CCGAGTTCCA CTGATTCGTC AGAGAAAGCT TGCTTCCTTA AAGCATGGCA
2651 ACCACTTCCA TCCATGCCGT ACTTTACGAC GAATTCCACC AAAGTTACGG
2701 CTTCAGAGGT GAGGTCTAGG TTCCCTAGTA GTATCTCTAC GATTCTGACA
2751 ATGGTGTGAT TGACCAGCTC TTGCAGATTT ACCTACAAAT GGAAGTTTAA
2801 TGTCAACATT TTAATCTTAT TTTCTCTTTA TGATATTATT TGAAATTTGT
2851 TGTGGTATTA CAGCTTCACG TGCACGGGCG TAGAAAGGAT ACTAGATATC
2901 ATTGAATCTA TTCTTACCTG AGCGGTCGTA TCACTGATGG TGATTCTGTC
2951 AGGGTAGCAA GCAGACTTAG CAGCTTGAAC AGCCTTATAT GAAGGGAAAA

3001 GTTTAGAATT GTGAGAGAGT GCAATATTGC GCAGTGCTTG ATAATCGTGC
3051 TTGGAGTCCT CGGTCTCGAT TAAGAAACTA AGAGCCTCCT CCGGGGTCAA
3101 TTGTGTTTCT TGCACTGAGT TGGTTGGCAC TTCACACTGT TCCGGACAAC
3151 TAAGGATAGA TGACACAGTT TTAGCTGCAG CATGATAGCC TTGCTGCCGA
3201 AGGACACTCT CGACGGCAAA AAATAGCTGC TCATTTGGAA CCTCGAGCCT
3251 CAGCGATTCT GTTTTTTGGC GCTTGGATCT GTCAGTACAC AAACTGAAGT
3301 CCTTCTTGGG ACGGCCGCGC TTTCTGTGCT CCACTTCAGA AGAGGATTGA
3351 CCTTCTAAAA AATGGAAATC ATGGATACAT GAAACTGTGA GTAGTGTTAC
3401 GCAATAAGAA AATCTGTAAG TAGCCTCATG GAGGTGGTAC CATCAGCAGC
3451 CGCATTTACG TCACTACCCT CAGGAGCAAA GGAACAGGTT GTTCCATGAC
3501 TGACCTCTCG TAGGAGTGAA ATGGGAGCAG AACCTGTATG AAGACATCAA
3551 GGTGACTTTA ATAATGATGA TAATCGAGGA ATCATTGAAA TGGCCTAGAA
3601 GATTCAATGG ATTACCCCCT AAAGCTGGAG CTGTAATTTC AAGCGCAACA
3651 TTCGCATCAT TGTCAGCACA GACTTGGGCT TCCATTGTGA GAGCATCGTC
3701 AGCATGATCG GTCTCAAGCA TGTCAAGAGC TGAATCACTG GAATCTGGAG
3751 GACCTGTGCA CATCCTGTCA TGGGATGCTC GATCATCAGG TACGTCCATG
3801 TTGATTGGAG CTGGTTCATA GGGTAATTTC TCTAACGCAC CGCTGGCTTC
3851 TAAACTAGAA ATGGATTCTG AAAATCAATT GTGCCATAAA ATATTAAAAA
3901 CTTCTGATTT CCTTTCCAAA AGCCTGTGAG AGCAGAAACG ATGTTAAGTA
3951 TACTTGCCAT GATTTGAGTA GTCCTTCGCA GACATGCCTT TTGGTGTGAC
4001 AGCGAGCCAC TTAGAATGTC TTTTCTCAAA TCTCGAAAAT TCATGACGGC
4051 ATGCTTTCAA ATGACTTTGA TACTCAGACA CGAACCTTGA GGCGCACCTC
4101 CTTACTTCGA CCTTTTCAGT GTCCGGCAAA TTAAGGCTTT GAAGGAGAAA
4151 CAATGAAAGA GCGTCACTTT GCTCATAGTT CTTCAAATCC CTGTCCCTGA
4201 TTTCCTGGTA CAAATCCCAA CGCGTGAATC CCTGATGCGC TGTTTGAAAG
4251 TGACAAATAC GAGATAATAA TGAGTAAAAA TGCTTCGACT AATGATACTT
4301 TGTTTACCGT TAAATGACTC CCTTGCCAAA CCTACCTTGC ATCATGATAA
4351 CTTACGAAAC TAAAAAGACA AACGATAGAA AGAAAATCTA TACCGTGAAT
4401 TGAATGCAAA AAGAAATGTA GAATTGTCGA TCATCAGAAT CAACTATTAA
4451 ATTGCATAAA AACGCAAAGT TCGCACCCAA ATGGGCAAGG CAATGTCAGC
4501 TAGCACAACA GTAGCTAAGG AGAACTTGCC AGTAAACAAT CTGTTAGAAA
4551 ATGAGACGGA ATTGATTAAA ATTGAGCAGA CAGAACAGCA TGTAAAAGCA
4601 TGTCAAAGTC AATTAATGCT AGTTTAAAAC TTTCACCAAA ACCCCGTCTG
4651 GAGAACGAAA CCATTCTTTT GTTCAGAAAA TCCACCATAG CTTCAAAAAA
4701 TTAAAAAGAA AACTTACTTT CACGCCAGTA GTTAAAACAA GAAAGGCGAA
4751 ACAAAATTCT AAGAGGGGAA AAAATGTTGA AGGGCTTACA AACAGTCCCC
4801 GATTTTAATG TAGGTATAAG CAAAATGTCT CTTTTCATTG CAGGGCCTCT
4851 AATATGAACA TCATTTAAGT AGGCTACTAG TTTGCAATGC CTTTGCCCTG
4901 GCTAGAGATC TTCCTCCTAC CTCGCTCTCT CAAGCTTGAT GTGTCACGTG
4951 TTTTGATTTT GGTAATTTCC TCAGAAAATT TGAATTTCAT TAGTGTATAT
5001 CCCTACGGCC AAGGGTATTG GGTGAGTGCA GTCCAGCACA CTCGCTTTAC
5051 TATCCTGGCA TTTTTTTTAT GTTTAATATT TCTGCATCGT GATTATAAAG
5101 GGATTGTCAT ATTCTGAAAA GCTGTCCACA TTGTTTTGAT ATTGTCACTT
5151 GATGGAAGTA TAACTTGACG TGTGTATGTA ATGAAGATGA ATCTCAAATT

5201 GTAGTAGCCT ACAGTTGAAT GGACTAAATA AATGTTCTTC TCTGAATAAT
5251 AATCCATAGG TCATTTTATA TACCAATACA TCAAAGTGTT TACGAAACGC
5301 TATCCATGTC TTTGCAAGGT TGATGCCCTG GAGTGAGAGT TACTTTTATA
5351 CTGCAAAATA CTATGATTTC TGCATATATT TGTGAAAAAG CATGTGAAGT
5401 GTCCTTGATG TCAAAGAATC GAAAAAGACA CATAGAGACT TGAGTGTGAT
5451 AAATAAGTGA CAACTTAAAC GCCAACAAAA CAAGTCATAA TTTGTGAAGT
5501 GGCCCCCCAG ACTAGAATTC ATGCGACATT TTTAACCTCA TTCAATTGCG
5551 AACTATGTAA AAATCTTCAA AAGGTGAACG TTTCGATGCT GGCGAGGACA
5601 CAGCATACCT GTAAAAGATG TTTCCTTTCT ACTTTAAGCC CCCCTCATCC
5651 CCAAGGAACT GGAGAATTTT TCTGGGATTT GACCGAATTT CGTGGTGCAG
5701 TCCGCTTAAA AGCCCCGGAG GGGTAATTTT TTATGGTTGG CAACTATCAA
5751 GCACTCGGTC AGACGTCAT

The transcript and protein sequences of *JsiTransib_3411* were predicted by AUGUSTUS.

QEOY01133411.1:1-5769 AUGUSTUS gene 51 4345 . - . ID=g1
QEOY01133411.1:1-5769 AUGUSTUS transcript 1345 4345 0.02 - . ID=g1.t1;Parent=g1
QEOY01133411.1:1-5769 AUGUSTUS stop_codon 1345 1347 . - 0 Parent=g1.t1
QEOY01133411.1:1-5769 AUGUSTUS intron 1403 1477 0.62 - . Parent=g1.t1
QEOY01133411.1:1-5769 AUGUSTUS intron 1893 1992 0.66 - . Parent=g1.t1
QEOY01133411.1:1-5769 AUGUSTUS intron 2783 2917 0.91 - . Parent=g1.t1
QEOY01133411.1:1-5769 AUGUSTUS intron 3355 3440 0.52 - . Parent=g1.t1
QEOY01133411.1:1-5769 AUGUSTUS intron 3534 3615 0.79 - . Parent=g1.t1
QEOY01133411.1:1-5769 AUGUSTUS intron 3855 4085 0.43 - . Parent=g1.t1
QEOY01133411.1:1-5769 AUGUSTUS intron 4240 4335 0.35 - . Parent=g1.t1
QEOY01133411.1:1-5769 AUGUSTUS CDS 1348 1402 0.36 - 1 ID=g1.t1.cds;Parent=g1.t1
QEOY01133411.1:1-5769 AUGUSTUS CDS 1478 1892 0.48 - 2 ID=g1.t1.cds;Parent=g1.t1
QEOY01133411.1:1-5769 AUGUSTUS CDS 1993 2782 0.79 - 0 ID=g1.t1.cds;Parent=g1.t1
QEOY01133411.1:1-5769 AUGUSTUS CDS 2918 3354 0.69 - 2 ID=g1.t1.cds;Parent=g1.t1
QEOY01133411.1:1-5769 AUGUSTUS CDS 3441 3533 0.53 - 2 ID=g1.t1.cds;Parent=g1.t1
QEOY01133411.1:1-5769 AUGUSTUS CDS 3616 3854 0.55 - 1 ID=g1.t1.cds;Parent=g1.t1
QEOY01133411.1:1-5769 AUGUSTUS CDS 4086 4239 0.31 - 2 ID=g1.t1.cds;Parent=g1.t1
QEOY01133411.1:1-5769 AUGUSTUS CDS 4336 4345 0.26 - 0 ID=g1.t1.cds;Parent=g1.t1
QEOY01133411.1:1-5769 AUGUSTUS start_codon 4343 4345 . - 0 Parent=g1.t1

coding sequence = [atgatgcaagcgcatcagggattcacgcgttgggatttgtaccaggaaatcagggacagggatttgaagaactatgagc
aaagtgacgctctttcattgtttctccttcaaagccttaatttgccggacactgaaaaggtcgaagtaaggaggtgcgcctcaagtttagaagccagc
ggtgcgttagagaaattaccctatgaaccagctccaatcaacatggacgtacctgatgatcgagcatcccatgacaggatgtgcacaggtcctccaga
ttccagtgattcagctcttgacatgcttgagaccgatcatgctgacgatgctctcacaatggaagcccaagtctgtgctgacaatgatgcgaatgttg
cgcttgaaattacagctccagctttaggggttctgctcccatttcactcctacgagaggtcagtcatggaacaacctgttcctttgctcctgagggt
agtgacgtaaatgcggctgctgatgaaggtcaatcctcttctgaagtggagcacagaaagcgcggccgtcccaagaaggacttcagtttgtgtactga
cagatccaagcgccaaaaaacagaatcgctgaggctcgaggttccaaatgagcagctatttttgccgtcgagagtgtccttcggcagcaaggctatc
atgctgcagctaaaactgtgtcatctatccttagttgtccggaacagtgtgaagtgccaaccaactcagtgcaagaaacacaattgaccccggaggag
gctcttagtttcttaatcgagaccgaggactccaagcacgattatcaagcactgcgcaatattgcactctctcacaattctaaacttttcccttcata
taaggctgttcaagctgctaagtctgcttgctaccctgacagaatcaccatcagtgatacgaccgctcaggtaaatctgcaagagctggtcaatcaca

ccattgtcagaatcgtagagatactactagggaacctagacctcacctctgaagccgtaactttggtggaattcgtcgtaaagtacggcatggatgga
agtggttgccatgctttaaggaagcaagctttctctgacgaatcagtggaactcggttcggatagcaactgcttcctagtcacctgcgtccccctaat
gttgagagaagctgagtcaaagaaagtcttatggcaaaatttaacgccctcttcaacaagatattgccgtcctattaagttaatatttggaagcaagg
aaaccggggactttgttcgggatatccatcaacaaatgaaaactgaaatccagttgctgcagccactggagttgactctgaaaactggaaaagtggta
tcggtttcttctaattttgtcatgacgatgatcgatggtaaagtaggcaacttgattttaaggtgctgcagcaatgcatgccctttgtgcaaggcccc
atcaaatcagctcaatgatatagaagcttgcctagagaggccattgcaaacagatgcggctgattttggtatgtcccctttacatgcaaccatcaact
gctttttaaacctattcaaaatagcttgtcatcaagatatccaacaatggagcaccaaagaccccggtgaactcacaacgattaaggctaggaaaatc
cttattcaggataggtttaagaaagagatgaaccttctcgttgatattcccatagcaggtggaaacacaacggacgggaacacagcacgtaaggtatt
tgacaatccggaaaaggccgcagcggtcatggaacttgacccgcagcttatcagacgatttgctgtcgtcctaaaggctatcgcgtccggaagagaga
tcaactcttccaaatacaatgcatacgctatagggacaggacaattgtacagagacctgtatccctgggcaaagatgtcaccaacagtgcatagagtc
ctgggccacggagcccaaatagctgaaaggtctcctctcccacttggtcttatgtcagaggatgcgcaggaatctatcaacaagcttcttagactgta
caggtcctcatacgcccgccagtgcagtagggaattcacgaatgaggacatcttcaagcgtctgcttgtcagatccgatccctacataagttctctga
catctatcccaaagaagctaagagcagttgatgacgaggttaactctctccttgtctaa]
protein sequence = [MMQAHQGFTRWDLYQEIRDRDLKNYEQSDALSLFLLQSLNLPDTEKVEVRRCASSLEASGALEKLPYEPAPINMDVPD
DRASHDRMCTGPPDSSDSALDMLETDHADDALTMEAQVCADNDANVALEITAPALGGSAPISLLREVSHGTTCSFAPEGSDVNAAADEGQSSSEVEHR
KRGRPKKDFSLCTDRSKRQKTESLRLEVPNEQLFFAVESVLRQQGYHAAAKTVSSILSCPEQCEVPTNSVQETQLTPEEALSFLIETEDSKHDYQALR
NIALSHNSKLFPSYKAVQAAKSACYPDRITISDTTAQVNLQELVNHTIVRIVEILLGNLDLTSEAVTLVEFVVKYGMDGSGCHALRKQAFSDESVELG
SDSNCFLVTCVPLMLREAESKKVLWQNLTPSSTRYCRPIKLIFGSKETGDFVRDIHQQMKTEIQLLQPLELTLKTGKVVSVSSNFVMTMIDGKVGNLI
LRCCSNACPLCKAPSNQLNDIEACLERPLQTDAADFGMSPLHATINCFLNLFKIACHQDIQQWSTKDPGELTTIKARKILIQDRFKKEMNLLVDIPIA
GGNTTDGNTARKVFDNPEKAAAVMELDPQLIRRFAVVLKAIASGREINSSKYNAYAIGTGQLYRDLYPWAKMSPTVHRVLGHGAQIAERSPLPLGLMS
EDAQESINKLLRLYRSSYARQCSREFTNEDIFKRLLVRSDPYISSLTSIPKKLRAVDDEVNSLLV]

No potential TIRs could be reliably identified surrounding the JliTransib_3411.

Silene latifolia

26. SlaTransib_8058

```
>QBIE01058058.1:1-4021 Silene latifolia isolate Sa984 Scaffold_58373,
whole genome shotgun sequence
   1 AATTTGTACC ATCAGCATGA AATGGATGAT TTTTGTATAT TAGGAAACAA
  51 ACTTTAGAAA AGTACCCTAA TATAGGTCAA AGAAGTTTAC CAACTAGTGG
 101 CATGAGGTTA CTTTGGTTCA AATTTTCTAG AAAAATAACA ATAAAAGTCA
 151 AACTTTCATT GTATTACAAT GATCGGTTCT TGATTATGTA CTGTTGAAGG
 201 ATCCTAGATG AACCTTGGAT GTCAAAAAAG CCCTTCTCAG GGTGGATAAT
 251 ATATTTTAAA AGTTGTTGGA TTTAATTATA CCTACATAAT TATATAGTTT
 301 GCCTAAATTA CTTCACTTAC AGTTTCTTTT TTTTCACAAT ATTTGAAATG
 351 ATATTACCAA CCATAAATTA TGAGCACTGG ATGTTAGTGT ATATTCAGCA
 401 TTTTCTACGT CTAGGTATGT GGAATATTCA ATACTTTCAG CAGCATACAC
 451 ACAGATTTAC TTAAAATTTA GCTACCGAAT TTAAGTGAAC TATTTCAACT
 501 TCAATATTGA CAATTTATCC GAATAAAATA AATATGTGAA AACCTGCTGA
 551 AAATGTACTT TTGAACAATT TCACTGCAAA CTATCACTTC ACTACAGTAT
 601 AGTTAAAGCT GAAGAACGAT TTTTTTTCAC GAATAGGTAT ACGAAAGCAC
 651 GAGTTAAGAG TTGAAAATGG TGGCACATAC AACAGTTTCC GTTTGCGGTT
 701 TCAACAAAGG GTTGGCCCGA AGTATAAGAC CGAGGACAGA ACTACGTGTG
 751 CTCACAAAAA CCTTCTTTTT TCTCCAATTC CTCCATCAAC CTACGACATA
 801 AAAACTCTGC CCACAGCAAT ACGGTGCACA GTAGCCATTT GTTGAGGGTG
 851 GAAAAGAGTC ATTCGAAAAT CCACCCATCT TTTTTTTTTT GCTTTTTCTC
 901 TTTTTTTCGT CCTCTTTCTA GCTTGAAAAA GGCTCGATCA ATATGTTCTA
 951 CGAGACAGAA GCACCAATAT ATTTGTACAG ACACGGTCAC GTGATTTAGG
1001 GTCTGAAATT TGTTCGCTCG AATGACCGTG TGACAGATTG ACCACAAGCC
1051 GACTAGTAAT AATGGACGAT GTTGATATAC GACCACCTTC TCCCGTGTAC
1101 CCCTAAATCC ACCTTCATTT CGATTTTTTC CCTCGTCAGT GTACACAAAT
                           >>>potential 3'TIR
1151 TCCTTTCAAC TGGTTACTGC ACAGTGGGAC GAATCGCGAA TCTAGGTGGA
1201 CAAAAATCAT ATTGTTGGAA AAACGGCGTG AAAAAGGNTA GTTTGAGCAG
1251 TAAAATCTAG ACTTAGTTTT TTATATTAAT AATCTTCATT ATCGCTGCTA
1301 TCGCTGCCAT TTAATGTGTT GATGTCAGTA TCGTCATCGC TAGTATCTGT
1351 AGATTCAGTA TGTAATAACA TCATCTTCGT CTCTGGTAAA ATGGTTTTTT
1401 TCTTCACTCG ATCTCGTCGC AGATGTGAAA TAACTGGATC GCTTGTAATC
1451 AACAGGTTGT TTAATATGTC TCTGGTAGTT GCTTCTCTTT TCGATTTACG
1501 AGTATGATGC TCTCGAATCC TGGCAAGGTG CAATAATTGT ATACCTATTT
1551 GTATCATATT GCCTTTGAAT TGAAAATATT AAAAATTACG TACCTACCTT
1601 TTATAGTCTT TGTTTCTTGC TTCTTGAGCT TCTTCAGAGA GGTCGCCTAT
1651 TGGTAAACGT AAATGTCGCA TCACTTGAGC TCCATGATGG AGCACTTTGT
1701 GCACAGTTGG AGGTAAATTG TGCCAACTGT ACAACATTAA ATATCGCTGC
1751 TCCGTCATTC GTGTATATTC TTCAAATTTT GGAATGTCGA CATTTTCACC
```

1801 ACAAGTTATT ACATTTATAA TCACCGAAAA CCTTTCAATC AACTCGTGAT
1851 CAACTCCAGT AATCTCACTA GTTTTTTTAG GATTTCGAAA AAATCTTCGG
1901 GCAGTATTGC CAGTATTAGT GGTCCCACCA CCTGGAAGAA CTTTATCTAC
1951 CTGTAAATGA ACGCAATATG CTCTGGTTAT TTCAGCTACG AGCATTTTAT
2001 TCCCCTAAAC TGACAATTTA TAATAGTAAG GTTTGAATTT ATACTTAGGC
2051 CTACCTTGAT TCCAAGTTCT TGATGAAGTT TTTTCCGAAT CTCTTTCGTT
2101 CGTTCTTCGA TTTCTTTTTT TTCTTCGATC GAAGCTCCAC GTTTAGTACG
2151 TTTTGCATAT CCCATTTGAT AACCCAGGGT AAGGACCAAT TCCATGAACT
2201 TGATCATCGC ATGTAAAACA CTTAAACCAT AGTTTAAATT GTCTTCTTTC
2251 ACTTCTAATT TAGCCAGCTG ACTTTTATCG TTCATCTGTG ACGGCTTCGC
2301 ACCACATATG AAGCAGCTCT GTAAAGATTC ACAAGATTAG AAAAAAAGAA
2351 TCGATTTCCA GATTACGGTA ATAGTCTTTT TTGGAATACG TAGGCTAATT
2401 TTTCGTTTCC TTCTCGAGAA TTTTAAATAC ATACTTGCGA CGATGATGAA
2451 GTTAAAGCGT TTATTACTTT TCCATCAACC ATTGACATAA CCATTTGGTA
2501 ATCGATCGTA AAATCCTTGC AGGTGAATAA TGTAAGGTTG GAAATTTCGT
2551 TTCGCATCTT CTCTACCTGG ATTTTAATAT CCTGTATAGT TTCTTTTAAA
2601 TACAGAAATT TGATAGGCCG ACAATATTTG GTAGAAGATG GTCGCGGATT
2651 TTTCCACAGC ACAATTTCTT GGCCATCATC TGTTTCCGCA ATTAGTCGAA
2701 GAGGTACTGT AGTAAAAAGA AATAAGTAAG AATCGCTCGG TGGTACTTGA
2751 TTTGAATCGT TGTGTTCTGC TGGTGCAGCA TCATTAAAAC TGGAATCATT
2801 TGACTGGTAA TATTCTGGAT GCCCACTACA TCCATCGCAG CCCCATTTAA
2851 GAACTAATGT GATCTTGCGA TACGTTCGCG ACTCCATATT CACGACTTCT
2901 GAAATAAATC TGCCAGCTGT ATGGTTCAAC AGTTGTTGAA GAGGAATTGA
2951 GGCACCTGAA TATAAAAATT TTTGCATGAA TAGAATTTAC TCATAGGTAC
3001 TTGAATAAAA CATTTTTCTT AATGAGCTTC GATCAGAAAG AAAAAACAAA
3051 AAAAAATCAA AGAACGAACA AAAGAGATTT GAAAAAAACT TCATCAAGAA
3101 GAATCAAGGT AGGCCTAAGT ACAAATTCAA ACTTTACTAC CTATTATAAA
3151 TTGTCAGTTT CGGGGAATAA AATGCTCGTA GCTGAAATAA CCAGGGCATA
3201 TTGCGTTCAT TCACAGGTAG ATAAAGCTTT TCCAGGGGGT AGGACCACTA
3251 ATCGATAAAT AATAAGGTAA TTTTCTAAGT AAACATAGAT AGGTAATTCT
3301 TTCTAGCAAA ATTTTCGTTC TTTTGTAGAC GCACAATCAT TGAAAAATTA
3351 ATTGAGAAAT TCTAATAGGC AATTGCATAA ACTCTGAATA AGGTGCAGTG
3401 AACCTTACCA TAATTATCGA TACATATATC AGGAGGGTAA CAACGTTTTT
3451 TGGCTTCTCC AATTTTGTGG TACGACGGAT AAATATCTGC TCCATGTTTT
3501 TTGGCGCCTA CACGCACCAT GATATAACTT TCTTTTGTGA GTCGAGCATC
3551 GATCATATGC GCAAGAGCTT CATCATCGGA GAACTGAATG ATCTTGTCCC
3601 CTGCATTAGC TTTTCGAACT GCTTTCGTTA TTTTCAACGC GCGCCCCGGT
3651 GAAAACTCAG TTGCTTGAGA TATTAATTCA GCCGCAGACC GTTTTCCTGA
3701 TTTGAGTAGT GATGTTTTCG TAGCAAACGC CAATTCTTCT TGAGAAGATG
3751 ATTCCAATAG GTTTGTGATT CGTCTTTTCT TCTCTCTTAC GCCTACTTCT
3801 GTAAATAATT GTTTCGGGCG ACCGCGAGGC CTGGGTTCAG GAGGTATGGA
3851 GGATGTTGAT GGTTTGCTAG GTGAAGGTGT AATTACTATT CCTGAAATAT
3901 CATGTTGGGT ATAATGTTTT TAGACTGCAA AGTTCAAATG AGTAAGTACC
3951 TAACCTAAGC AGTAAGTTCA ATTGAATAGC AGGCTGCAAA AAGGGCCTCC

4001 AAGAGTGGGA GGCCCTTTTT C

The transcript and protein sequences of *SlaTransib_8058* were predicted by AUGUSTUS.

QBIE01058058.1:1-4021 AUGUSTUS transcript 1276 3798 0.09 - . ID=g1.t3;Parent=g1

QBIE01058058.1:1-4021 AUGUSTUS stop_codon 1276 1278 . - 0 Parent=g1.t3

QBIE01058058.1:1-4021 AUGUSTUS CDS 1276 1519 1 - 1 ID=g1.t3.cds;Parent=g1.t3

QBIE01058058.1:1-4021 AUGUSTUS CDS 1598 1950 1 - 0 ID=g1.t3.cds;Parent=g1.t3

QBIE01058058.1:1-4021 AUGUSTUS CDS 2055 2318 0.99 - 0 ID=g1.t3.cds;Parent=g1.t3

QBIE01058058.1:1-4021 AUGUSTUS CDS 2435 2955 0.97 - 2 ID=g1.t3.cds;Parent=g1.t3

QBIE01058058.1:1-4021 AUGUSTUS CDS 3409 3798 0.09 - 2 ID=g1.t3.cds;Parent=g1.t3

coding sequence = [aagtaggcgtaagagagaagaaaagacgaatcacaaacctattggaatcatcttctcaagaagaattggcgtttgctac
gaaaacatcactactcaaatcaggaaaacggtctgcggctgaattaatatctcaagcaactgagttttcaccggggcgcgcgttgaaaataacgaaag
cagttcgaaaagctaatgcaggggacaagatcattcagttctccgatgatgaagctcttgcgcatatgatcgatgctcgactcacaaaagaaagttat
atcatggtgcgtgtaggcgccaaaaaacatggagcagatatttatccgtcgtaccacaaaattggagaagccaaaaaacgttgttaccctcctgatat
atgtatcgataattatggtgcctcaattcctcttcaacaactgttgaaccatacagctggcagatttatttcagaagtcgtgaatatggagtcgcgaa
cgtatcgcaagatcacattagttcttaaatggggctgcgatggatgtagtgggcatccagaatattaccagtcaaatgattccagttttaatgatgct
gcaccagcagaacacaacgattcaaatcaagtaccaccgagcgattcttacttatttctttttactacagtacctcttcgactaattgcggaaacaga
tgatggccaagaaattgtgctgtggaaaaatccgcgaccatcttctaccaaatattgtcggcctatcaaatttctgtatttaaaagaaactatacagg
atattaaaatccaggtagagaagatgcgaaacgaaatttccaaccttacattattcacctgcaaggattttacgatcgattaccaaatggttatgtca
atggttgatggaaaagtaataaacgctttaacttcatcatcgtcgcaaagctgcttcatatgtggtgcgaagccgtcacagatgaacgataaaagtca
gctggctaaattagaagtgaaagaagacaatttaaactatggtttaagtgttttacatgcgatgatcaagttcatggaattggtccttaccctgggtt
atcaaatgggatatgcaaaacgtactaaacgtggagcttcgatcgaagaaaaaaaagaaatcgaagaacgaacgaaagagattcggaaaaaacttcat
caagaacttggaatcaaggtagataaagttcttccaggtggtgggaccactaatactggcaatactgcccgaagatttttttcgaaatcctaaaaaaac
tagtgagattactggagttgatcacgagttgattgaaaggttttcggtgattataaatgtaataacttgtggtgaaaatgtcgacattccaaaatttg
aagaatatacacgaatgacggagcagcgatatttaatgttgtacagttggcacaatttacctccaactgtgcacaaagtgctccatcatggagctcaa
gtgatgcgacatttacgtttaccaataggcgacctctctgaagaagctcaagaagcaagaaacaaagactataaaaggattcgagagcatcatactcg
taaatcgaaaagagaagcaactaccagagacatattaaacaacctgttgattacaagcgatccagttatttcacatctgcgacgagatcgagtgaaga
aaaaaaccattttaccagagacgaagatgatgttattacatactgaatctacagatactagcgatgacgatactgacatcaacacattaaatggcagc
gatagcagcgataatgaagattattaa]

protein sequence = [VGVREKKRRITNLLESSSQEELAFATKTSLLKSGKRSAAELISQATEFSPGRALKITKAVRKANAGDKIIQFSDDEAL
AHMIDARLTKESYIMVRVGAKKHGADIYPSYHKIGEAKKRCYPPDICIDNYGASIPLQQLLNHTAGRFISEVVNMESRTYRKITLVLKWGCDGCSGHP
EYYQSNDSSFNDAAPAEHNDSNQVPPSDSYLFLFTTVPLRLIAETDDGQEIVLWKNPRPSSTKYCRPIKFLYLKETIQDIKIQVEKMRNEISNLTLFT
CKDFTIDYQMVMSMVDGKVINALTSSSSQSCFICGAKPSQMNDKSQLAKLEVKEDNLNYGLSVLHAMIKFMELVLTLGYQMGYAKRTKRGASIEEKKE
IEERTKEIRKKLHQELGIKVDKVLPGGGTTNTGNTARRFFRNPKKTSEITGVDHELIERFSVIINVITCGENVDIPKFEEYTRMTEQRYLMLYSWHNL
PPTVHKVLHHGAQVMRHLRLPIGDLSEEAQEARNKDYKRIREHHTRKSKREATTRDILNNLLITSDPVISHLRRDRVKKKTILPETKMMLLHTESTDT
SDDDTDINTLNGSDSSDNEDY]

```
                                          *        20         *        40         *        60         *        80
QBIE01058058.1:1068-1268 : -GATGTTGATATACGACCACCTTCTCCCGTGTACCCCTAAATCCACCTTCATTTCGATTTTTTCCCTCGTCAGTGTACAC : 1146
QBIE01141909.1:1-200     : -TAGCAGCCAGTCAGCACAACTGAAGACCTGCGATTGCCTCAAAGTTTCCGTCTCGAGCCACTCTTCTGACCTCTGGCGT :  122
QBIE01040561.1:1-207     : AAATAATGGAAATCGTATTCTTCGATTTTCTAGATAATCTAAAATCAATGTTTTTGATTTTTCAGAA-ACTGGAAAATCG :  129
HvuTransib_9086_3'TIR    : -------------------------------------------------------------------------------- :    -
HvuTransib_9086_5'TIR    : -------------------------------------------------------------------------------- :    -

                                          *       100         *       120         *       140         *       160
QBIE01058058.1:1068-1268 : AAATTCCTTTCAACTGGTTACTGCACAGTGGACGAATCGCGAATCTAGGTGGACAAAAATCATATTGTTGGAAAAACGG : 1226
QBIE01141909.1:1-200     : GCTACTTTCACATACTCGCAGTGCACAGTGGACGAATCGCGAATCTAGGTGGACAAAAATCATATTGTTGGAAAAACGG :   42
QBIE01040561.1:1-207     : AAATTTTCCAATTTTAGACATTGCACAGTGGACGAATCGCGAATCTAGGTGGACAAAAATCATAAAAATGGAATATTTC :   49
HvuTransib_9086_3'TIR    : -----------------------CACAGTGGCCAGAATTCAATTCTAAGTGGCCAAAAATATTTTTTTCCTTATCACT :   57
HvuTransib_9086_5'TIR    : -----------------------CACAGTGTGCCAGATATCATTTCTAAGCGGTCAAAAATAGGGTTTTTTCATACTCAC :    7
                                                   CACAGTGgG C  At  C a TCTA GtGG CAAAAAT  t tt tT  a a
```

Figure 16 Sequence alignment of potential TIRs of SlaTransib_8058 and HovTransib_9086 showed their conservation.

Arabis nordmanniana

27. AnoTransib_1776

>LNCG01061776.1:1-2801 Arabis nordmanniana contig_116069, whole genome shotgun sequence

```
   1 GGGGCAATTG ACATCGATTA GGCATTAAAT CATAGGGTAA AAAAATATAT
  51 TCCTAAAATT AATATTATTT TTATATTTAT TCATCAGACA TATACAAAGT
 101 CGTTTACATA GTATTAACAT AAGGTATAAA GTATGTACGG TATTTATTTA
 151 TTTAATTTAT TAACAAAATT ATAAAAATGT ATTATTTTAA GTTTTAAGTT
 201 TAAAACTGGT CATGGTTATT GTCTTCATTG TCTTCGTTGC TGTCATTATC
 251 TTCGTCATAA TCATCATCAT TATCGTTGTC GTTATCATCA TCATCATTAT
 301 CGTTGTCGTC ATCATCATCA TCATCGGTAT CTTTGGCATA TTGAAGAGAG
 351 ACTCGGATAT CTGGTGATTT TAGTAATGCG AGTGCTTCAA TTGGCATTGA
 401 TTGTGTTTTT TTCTTCAGTA ATTTTCTTTT ACTTGAAATT ACTGGATCTG
 451 AACTTAATAA AAATAAATTT AATATATCTA AATTAGTTTT TTCACGACTA
 501 CATTTCCTAG AGTTATGCTC TCGATATCTT TTGAAATCTT TATTTCGCGC
 551 TTCTTGGGCT TCTTCAGTTA ACTGTCCTAT TGGCAACAAA GCCGATTCAA
 601 TTATCTCTGG TCCATGTATA AAAAACTTGT GCATCGTGGG TGACATATTA
 651 TACCACGGAT ACTTCTCAAC AAAATATTTT GCAGTTTTAT ACGTGTATTC
 701 CCGAAACTCA TTGATTTTTA TTTCGTGTCC ACTTGCTACT ACTATCATTA
 751 CTATATGTAT TTTTTCTATT ATTTCTACGT CTAATTTTGT GATTTCAGCA
 801 GATATTTCTG AATTTTGGAA AAAACGTCTG GCTGTGTTCC CATCATTAGA
 851 ATTTCCAAAT CCTGGTTTGG GTTTATCTAC AAGTAATCCT AATTTGTCTT
 901 TGAATTCTTT TTGAATTCTT AGCTTATTTT CTGAAACAAT TTTTTTTTCC
 951 ACATCTCCTC TAGCTTGCCA TTTTTTAAGT GGTAATTTGT AAGACAAATG
1001 AAGGAGACAT TCGAAAAAAC GAATCCACCC GTGTAAAACT GATATCCCAA
1051 AAGATAAATT ATCAGTTTTT ACCTCTCGTT TAACCATTTC ATCAATTAAA
1101 TTAAACTGTT TCGATGTTGC ACCACACAAG AAACATTTAA GTGTTGATGA
1151 CGTATCAGTG AGTGCATTAC ATATTTTCCC ATCAACCATT GCTAAAATCA
1201 AATTGTGAGT TACAATAAAT GATTTGTTAT CAAAAGTCAC AATACTATTT
1251 ATTAACTTAT TTATTTGACT CTGAACTTTT TCTTTCTCAG CTATAGATGT
1301 ATCTGTAGAT TCTTTTATAA ACTCAATTTT TATAGGCCTA CAATATCTAG
1351 TTGATGCAGG ACGTGGGTTT TGCCATATAA TTTGGTCACC GTTTACCAAG
1401 CGAAGTGGAA CTATACTTGT AATAAATACA GCCGAATCAC TGGCTTCGGG
1451 ACTACAAAAA GCTTGTTTAT ATGAACTATG TCCAGAGCTG CCATCAAAGC
1501 CCCATTTTGA ATACAAAACG AACTTATCTA ATTCATTTTC TTGGAGTAAG
1551 ACAATAACAG GCTTTTGCAC TAAAAATAAT CGTTCTACTG TGTGATCAAG
1601 TAATGCTTGT AAAGAAACTT CTGCGTTTGT AGAAGTTACT TGTATGTTTT
1651 CTGGTTTTGG ATAACACTGT TTTTTACTTT CTTGTACGAC TTTGTATGAT
1701 GGAAATTTAT CTGGGGCACT CATGCGAATA ATATTGTACT GATGTCTAGA
1751 TAGCTTTGCA TCTACTATAA CAGCTAGAGC ATCTTCACCC GATAGTTGTT
1801 TAGGTGCTTG TGCAGACTGC TTCTTAAATA CTTTTCTATA TCTACTTGCA
```

1851 CGTGTGGGGG TGGATGTAGT TATTTCCTTC AGTAACTTTG CGGCTTCAAC
2001 CTACTCATCT CAAATGCCAC TACTGGACGA CCACGATTAT TCTGGTTATT

1901 TTGTCCCGAT GCTCTTAAGC TCATCTGTGT TGCATGACTT AACACATTAA
1951 CAGACGTATT ATTTCTCAGT TCTTGAGTTA TTTTCCGCTT GTTTCGTTCA
2051 TAAAGATATA AATTTAATAG AAGCATTTAA CCAGTTTTTA TTTTCTATAA
2101 TAAATCGTTC TTCCACTCTA TGTGCCTTTT CCCAACGAGA TTTATATTCA
2151 GATTTGAAAT AAGACAATTT TTTTTTCAAA TCATTTTGCT CACAGTTTTT
2201 CACTCGTTTT TCTATTTCGA TTTTTATCAA ATTAAATTTT TCATCAATAG
2251 TCTCACATCC AGAACTTTTA ATTATATCAT ATAATTTTTG TCTGGATATC
2301 AAAACAGTGC TCGAATTCAT TCCTATACAA ATATTTTTAA ACTAATTATG
2351 ACGTTTGTCT TCATCAACAT AATATTTCTA TAGGTACCTA CTATCAAAAT
2401 TAGCAATAAA TAATACAGAC ATGAGATAGT ACATAAATAA TGTGTTCAAA
2451 AAGTTTGGAA ATGCTTTAAT TACTATTTGT GTATTTTAAT GCATACACAG
2501 AAAAATAACA TATAACACTG CTACACCTTT GTAAGGTAAC ATTATTAATA
2551 AACTCATTAA TTATAGTAAA TAAAGCAATT TTTTTCATGT GAAATTGGTA
2601 TAGGTCGTCA CAGTTAGAAT TTATTTACAC GAAATGGGGC AAATATGGGC
2651 AACATTTTTT TTAACAAGGT AATAGAATAC TCTATTAAGT AATATATTTT
2701 GCAATTTGTA TTGCTTGGAA AGCCTTGGAA GTGAACTTAA ATTATATATC
2751 ATTTTCGTGT TACAAATATA TTATCATTAT TATTAGTTAT CTAGGTAAAT
2801 A

The transcript and protein sequences of *AnoTransib_1776* were predicted by AUGUSTUS.

LNCG01061776.1:1-2801 AUGUSTUS gene 200 2320 . - . ID=g1

LNCG01061776.1:1-2801 AUGUSTUS transcript 200 2320 0.64 - . ID=g1.t1;Parent=g1

LNCG01061776.1:1-2801 AUGUSTUS stop_codon 200 202 . - 0 Parent=g1.t1

LNCG01061776.1:1-2801 AUGUSTUS CDS 203 2320 0.64 - 0 ID=g1.t1.cds;Parent=g1.t1

LNCG01061776.1:1-2801 AUGUSTUS start_codon 2318 2320 . - 0 Parent=g1.t1

coding sequence = [atgaattcgagcactgttttgatatccagacaaaaattatatgatataattaaaagttctggatgtgagactattgatg
aaaaatttaatttgataaaaatcgaaatagaaaaacgagtgaaaaactgtgagcaaaatgatttgaaaaaaaaattgtcttatttcaaatctgaatat
aaatctcgttgggaaaaggcacatagagtggaagaacgatttattatagaaaataaaaactggttaaatgcttctattaaatttatatctttaaataa
ccagaataatcgtggtcgtccagtagtggcatttgagatgagtagtgaacgaaacaagcggaaaataactcaagaactgagaaataatacgtctgtta
atgtgttaagtcatgcaacacagatgagcttaagagcatcgggacaagttgaagccgcaaagttactgaaggaaataactacatccacccccacacgt
gcaagtagatatagaaaagtatttaagaagcagtctgcacaagcacctaaacaactatcgggtgaagatgctctagctgttatagtagatgcaaagct
atctagacatcagtacaatattattcgcatgagtgccccagataaatttccatcatacaaagtcgtacaagaaagtaaaaaacagtgttatccaaaac
cagaaaacatacaagtaacttctacaaacgcagaagtttctttacaagcattacttgatcacacagtagaacgattatttttagtgcaaaagcctgtt
attgtcttactccaagaaaatgaattagataagttcgttttgtattcaaaatggggctttgatggcagctctggacatagttcatataaacaagcttt
ttgtagtcccgaagccagtgattcggctgtatttattacaagtatagttccacttcgcttggtaaacggtgaccaaattatatggcaaaacccacgtc
ctgcatcaactagatattgtaggcctataaaaattgagtttataaaagaatctacagatacatctatagctgagaaagaaaaagttcagagtcaaata
aataagttaataaatagtattgtgacttttgataacaaatcatttattgtaactcacaatttgattttagcaatggttgatgggaaaatatgtaatgc
actcactgatacgtcatcaacacttaaatgtttcttgtgtggtgcaacatcgaaacagtttaatttaattgatgaaatggttaaacgagaggtaaaaa
ctgataatttatcttttgggatatcagttttacacgggtggattcgtttttttcgaatgtctccttcatttgtcttacaaattaccacttaaaaaatgg
caagctagaggagatgtggaaaaaaaaattgtttcagaaaataagctaagaattcaaaaagaattcaaagacaaattaggattacttgtagataaacc
caaaccaggatttggaaattctaatgatgggaacacagccagacgtttttttccaaaattcagaaatatctgctgaaatcacaaaattagacgtagaaa
taatagaaaaaatacatatagtaatgatagtagtagcaagtggacacgaaataaaaatcaatgagtttcgggaatacacgtataaaactgcaaaatat

tttgttgagaagtatccgtggtataatatgtcacccacgatgcacaagtttttatacatggaccagagataattgaatcggctttgttgccaatagg
acagttaactgaagaagcccaagaagcgcgaaataaagatttcaaaagatatcgagagcataactctaggaaatgtagtcgtgaaaaaactaatttag
atatattaaatttatttttattaagttcagatccagtaatttcaagtaaaagaaaattactgaagaaaaaaacacaatcaatgccaattgaagcactc
gcattactaaaatcaccagatatccgagtctctcttcaatatgccaaagataccgatgatgatgatgatgacgacaacgataatgatgatgatgataa
cgacaacgataatgatgatgattatgacgaagataatgacagcaacgaagacaatgaagacaataaccatgaccagttttaa]
protein sequence = [MNSSTVLISRQKLYDIIKSSGCETIDEKFNLIKIEIEKRVKNCEQNDLKKKLSYFKSEYKSRWEKAHRVEERFIIENK
NWLNASIKFISLNNQNNRGRPVVAFEMSSERNKRKITQELRNNTSVNVLSHATQMSLRASGQVEAAKLLKEITTSTPTRASRYRKVFKKQSAQAPKQL
SGEDALAVIVDAKLSRHQYNIIRMSAPDKFPSYKVVQESKKQCYPKPENIQVTSTNAEVSLQALLDHTVERLFLVQKPVIVLLQENELDKFVLYSKWG
FDGSSGHSSYKQAFCSPEASDSAVFITSIVPLRLVNGDQIIWQNPRPASTRYCRPIKIEFIKESTDTSIAEKEKVQSQINKLINSIVTFDNKSFIVTH
NLILAMVDGKICNALTDTSSTLKCFLCGATSKQFNLIDEMVKREVKTDNLSFGISVLHGWIRFFECLLHLSYKLPLKKWQARGDVEKKIVSENKLRIQ
KEFKDKLGLLVDKPKPGFGNSNDGNTARRFFQNSEISAEITKLDVEIIEKIHIVMIVVASGHEIKINEFREYTYKTAKYFVEKYPWYNMSPTMHKFFI
HGPEIIESALLPIGQLTEEAQEARNKDFKRYREHNSRKCSREKTNLDILNLFLLSSDPVISSKRKLLKKKTQSMPIEALALLKSPDIRVSLQYAKDTD
DDDDDDNDNDDDDNDNDNDDDYDEDNDSNEDNEDNNHDQF]

No potential TIRs could be reliably identified surrounding the AraTransib_1776.
