# Supplementary material for: The RAG key to vertebrate adaptive immunity descended directly from a bacterial ancestor
Source: Natl Sci Rev. 2022 Apr 18;9(8):nwac073. doi: 10.1093/nsr/nwac073 (PMC9435367; doi:10.1093/nsr/nwac073)
Supplement: nwac073_Supplemental_Files [file nwac073_supplemental_files.zip › Supplementary Data_taoxin-20220402-V5.pdf]

SUPPLEMENTARY DATA 1

The RAG key to vertebrate adaptive immunity descended directly from a bacterial ancestor

Xin Tao[1, 3], Ziwen Huang[1], Fan Chen[1], Xinli Wang[1], Tingting Zheng[5], Shaochun Yuan[1, 4, *], Anlong Xu[1, 2, *]

[1] State Key Laboratory of Biocontrol, Guangdong Key Laboratory of Pharmaceutical Functional Genes, Southern Marine Science and Engineering Guangdong Laboratory (Zhuhai), School of Life Sciences, Sun Yat-sen University, Guangzhou, 510275, China

[2] School of Life Sciences, Beijing University of Chinese Medicine, Dong San Huan Road, Chao-yang District, Beijing, 100029, China

[3] Center for Infection and Immunity, School of Medicine, Sun Yat-sen University, Guangzhou, 510275, China

[4] Laboratory for Marine Biology and Biotechnology, Qingdao National Laboratory for Marine Science and Technology, Qingdao, People's Republic of China

[5] Shanghai Institute of Immunology, College of Basic Medical Sciences, Shanghai Jiao Tong University School of Medicine, 200025 Shanghai, China

[*] Correspondence to be sent to: Anlong Xu, School of Life Sciences, Sun Yat-sen University, No. 135, Xingang Xi Road, Guangzhou, 510275, P. R. China; E-mail: lssxal@mail.sysu.edu.cn; Shaochun Yuan, School of Life Sciences, Sun Yat-sen University, No. 135, Xingang Xi Road, Guangzhou, 510275, P. R. China; E-mail: yuanshch@mail.sysu.edu.cn

MATERIALS AND METHODS

Cell Culture of *Aureococcus anophagefferens*

The axenic strain of *Aureococcus anophagefferens* (CCMP strain number 1984) was purchased from the Provasoli-Guillard National Center for Culture of Marine Phytoplankton, Bigelow Laboratory for Ocean Science, Maine. The cultures were grown in sterile L1 media at 19±1°C on a 13/11 h light/dark cycle at a luminous intensity of 120 mmol photons m^{-2} s^{-1}. Algal growth was monitored on a daily basis via *in vivo* chlorophyll a fluorescence using flow cytometry (Beckman CytoFLEX) with a 485 nm excitation laser and a 690±50 nm optical filter.

Sources of Corals and Ribbon Worms

Coral samples (*F. costulata and F. tenuis*) were a gift from Researcher Hui Huang at the South China Sea Institute of Oceanology, Chinese Academy of Sciences. These corals were collected from the sea near the San Ya city, Hai Nan Province of China. *N. geniculatus* was a gift from Professor Shichun Sun at the Institute of Evolution & Marine Biodiversity, Ocean University of China. These animals were collected from the beach of Qingdao city, Shandong Province of China.

Extraction of Total RNA and Genomic DNA

A. anophagefferens was inoculated at an initial cell density of 1×10^5 cells/ml. After 5-7 d of continuous culture, the cell density reached to 5×10^6~1×10^7 cells/ml. The cultures were then aliquoted into small samples, each with approximately 1.5×10^8 cells. The cells were harvested via centrifugation at 8000 g for 10 min, and immediately stored in liquid nitrogen until extraction. Total RNA was extracted using the RNeasy mini kit (Qiagen) according to the

manufacturer's instructions. An additional step of treatment with a DNase I stock solution (Qiagen) on the membrane was adopted to reduce the contamination of genomic DNA. The purified RNA was further treated with the TURBO DNA-free Kit (Thermo Scientific) to eliminate traces of genomic DNA. The RNA concentrations were quantified with the NanoDrop 8000 spectrophotometer (Thermo Scientific). The integrity of the total RNA was assessed by 1% (wt/vol) agarose gel electrophoresis. Genomic DNA was extracted using the DNeasy Blood & Tissue kit (Qiagen) according to the manufacturer's instructions.

Synthesis of First Strand of Normal cDNA and RACE cDNA

The mRNA was first purified from the total RNA with the Oligotex mRNA mini kit (Qiagen) according to the manufacturer's instructions. With the purified mRNA, the first strand of normal cDNA was synthesized with SuperScript IV Reverse Transcriptase according to the manufacturer's instructions. The RACE cDNA was prepared with the Clontech SMARTer RACE 5'/3' Kit.

Splinkerette PCR to Reveal the Loci of *RAGL* Transposons

Genomic DNA from different samples was prepared with the DNeasy kit (Qiagen). The purified genomic DNA was digested with BstYI restriction endonuclease (NEB) for 2.5 h. After purification, the fragmented genomic DNA was ligated to the adapter, which came from the annealing of two primers: SPLNK-TOP and SPLNK-BOT. The product of ligation was used as the template for two rounds of nested PCR. To clone the flanking sequence of 5'TIR, the primers for the first round of PCR were SPLNK_S1 and 5TIR_S2, and those for the second round of PCR were SPLNK_S2 and 5TIR_S1. To clone the flanking sequence of 3'TIR, the

primers for the first round of PCR were SPLNK_S1 and 3TIR_S2, and those for the second round of PCR were SPLNK_S2 and 3TIR_S1. The PCR products were separated on a 1.2% agarose gel, and the typical bands were extracted, ligated into pGEM-T Easy vector (Promega), and transformed into *E. coli* DH5α for Sanger sequencing. Sequences were mapped to the species reference genome through BLAST. The primers used in Splinkerette PCR were listed in the Supplementary Table S2.

Identification of RAGL and Transib Homologs and Phylogenetic Analysis

As of April 18, 2020, the number of organism genomes in NCBI had grown nearly to 870,000, which provided valuable material for the identification of RAGL and Transib homologs. Excluding the jawed vertebrate genomes, the rest of the genomes were downloaded from the NCBI Genome database with Python package. All the latest RefSeq assemblies were selected when there were alternative assemblies for one organism. In addition, several coral genomes were downloaded from the Reefgenomics website (http://reefgenomics.org/) [1]. Several ascidian genomes were downloaded from the Aniseed website (https://www.aniseed.cnrs.fr/aniseed/) [2]. In total, we collected over 680,000 genomes, as shown in Supplementary Table S1. All these genomes were then combined and built into BLAST databases. The protein sequences of the RAG1 core and RAG2 core from lancelet, hemichordate, and pelagophyte were selected as the query sequences and were used to search the databases with the tblastn program. The threshold of the E-value was set to 0.001. The BLAST result with an E-valve in the range of e^{-5}~0.001 was inspected manually with regard to the bits score (>50) and blast length (>50 aa) [3]. The filtered subject DNA sequences were extracted from the genomes with an expanded range of 3000 nt at each

terminus when allowable. The profiles of vertebrate RAG1 and RAG2 and invertebrate RAG1L and RAG2L were built, respectively, through multiple sequence alignment. Under the instruction of these profiles, the Augustus program was used to predict the genes in the subject genome sequences. The best prediction of RAG homologs was selected based on the bit scores when querying with the previously cloned RAG homologs. The Transib homologs were identified in a similar way using 67 Transib proteins from the Repbase as the query sequences [4]. For TIR detection, extended RAGL or Transib was queried to its host genome. Alignment between the polymorphic copies would reveal a shift in the flanking sequences and a pair of conserved TIR region. Surrounding the 5'- and 3'TIRs, a pair of 5 bp TSDs with identical sequences were always found.

All newly predicted RAGL and Transib homologs were aligned, respectively, in T-coffee using the psi mode. The abnormal sequences were polished out, and the remainder were sent for alignment. In the phylogenetic analysis of RAG1 and Transibs homologs, the core regions of RAG1 were extracted from the alignment and used to construct an ML tree with IQ-tree. In the phylogenetic analysis of RAG2 homologs, the core domains including 6*Kelch repeats, were extracted and used to construct an ML tree with IQ-tree [5-7].

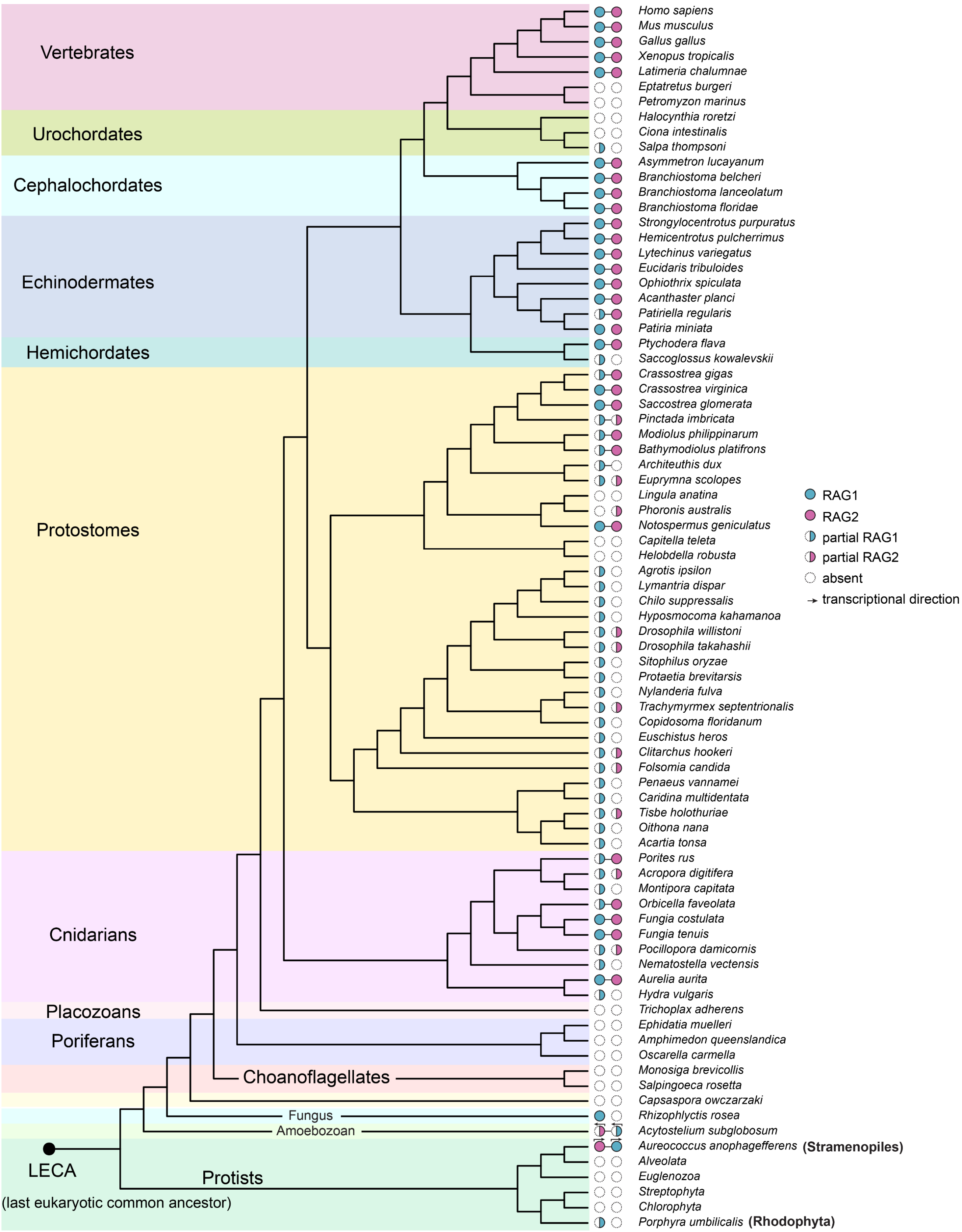

Supplementary Figure S1. Distribution of *RAG* and *RAGL* homologs across major phyla of eukaryotes. The color-filled circles show the existence of nearly complete *RAG* or *RAGL*

genes in species genomes, and the color-filled half-cycles represent the existence of partial fragments of *RAG* or *RAGL* genes in species genomes, and the empty circles represent the absence. The lines linking the circles show the joining of *RAG1-RAG2* or *RAG1L-RAG2L* genes in genome loci, and the paired *RAGL* genes transcribed in the same direction were marked with arrows. The branch length and relationship between species were drawn by referring to previously published studies [8, 9].

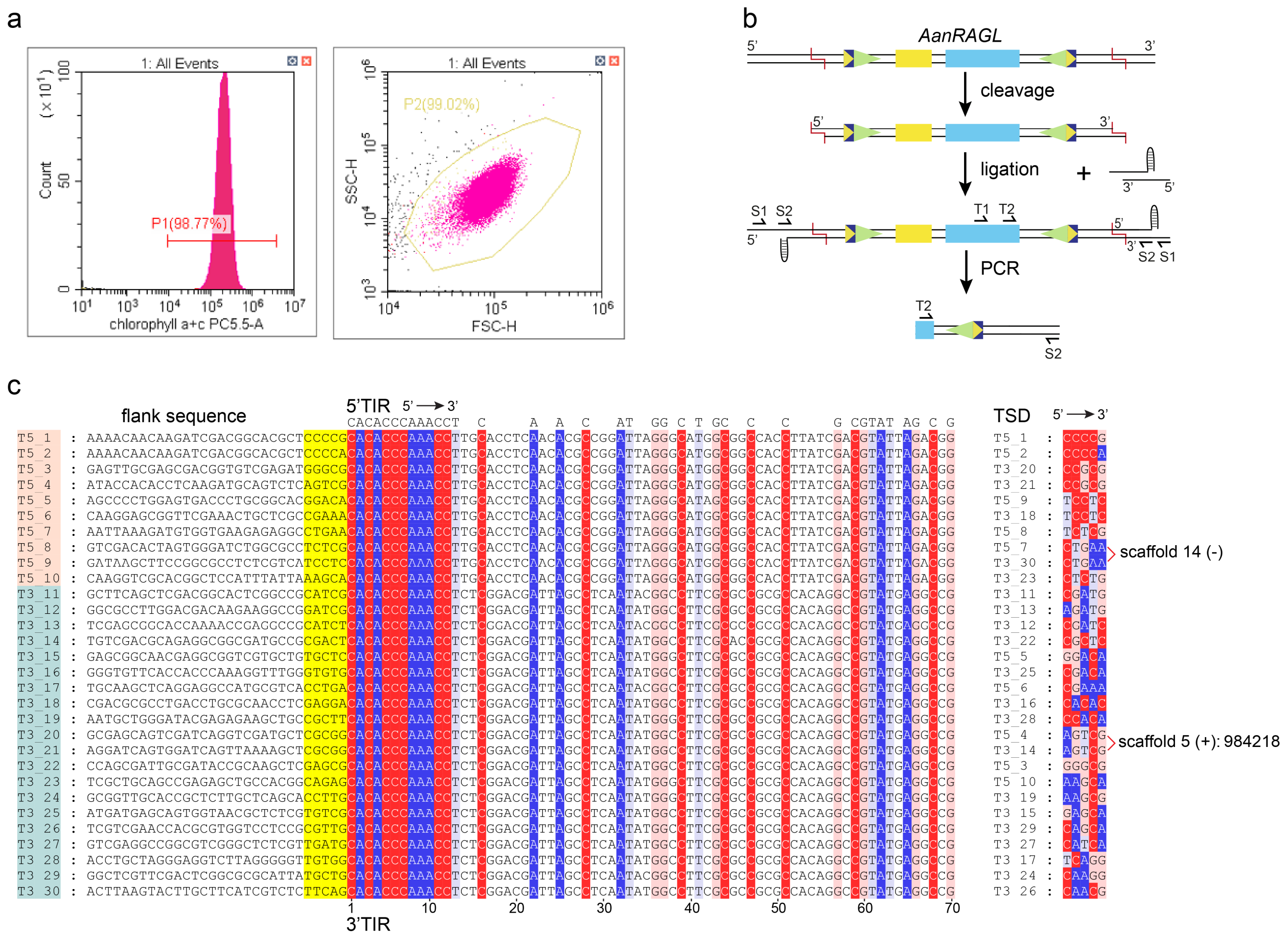

Supplementary Figure S2. Analysis of the TIRs and flanking sequences of *AanRAGL*.

a) Cell counting and viability analysis of cultured *A. anophagefferens* with flow cytometry.

b) Schematic diagram of Splinkerette PCR to reveal flanking sequences of the *AanRAGL* transposon. The adapter primers and genome specific primers were designed as shown.

c) Multiple sequence alignment of flanking sequence+5'-/3'-TIR sourced from Splinkerette PCR. 5'-/3'-TIRs were grouped, and colored with salmon pink and pale bluish green, respectively. The unspaced consensus sequences between 5'-/3'-TIR were shown above, and the putative TSD region were colored with yellow. The list of TSD sequences were reordered to clearly show the paired TSDs from the same *AanRAGL* loci.

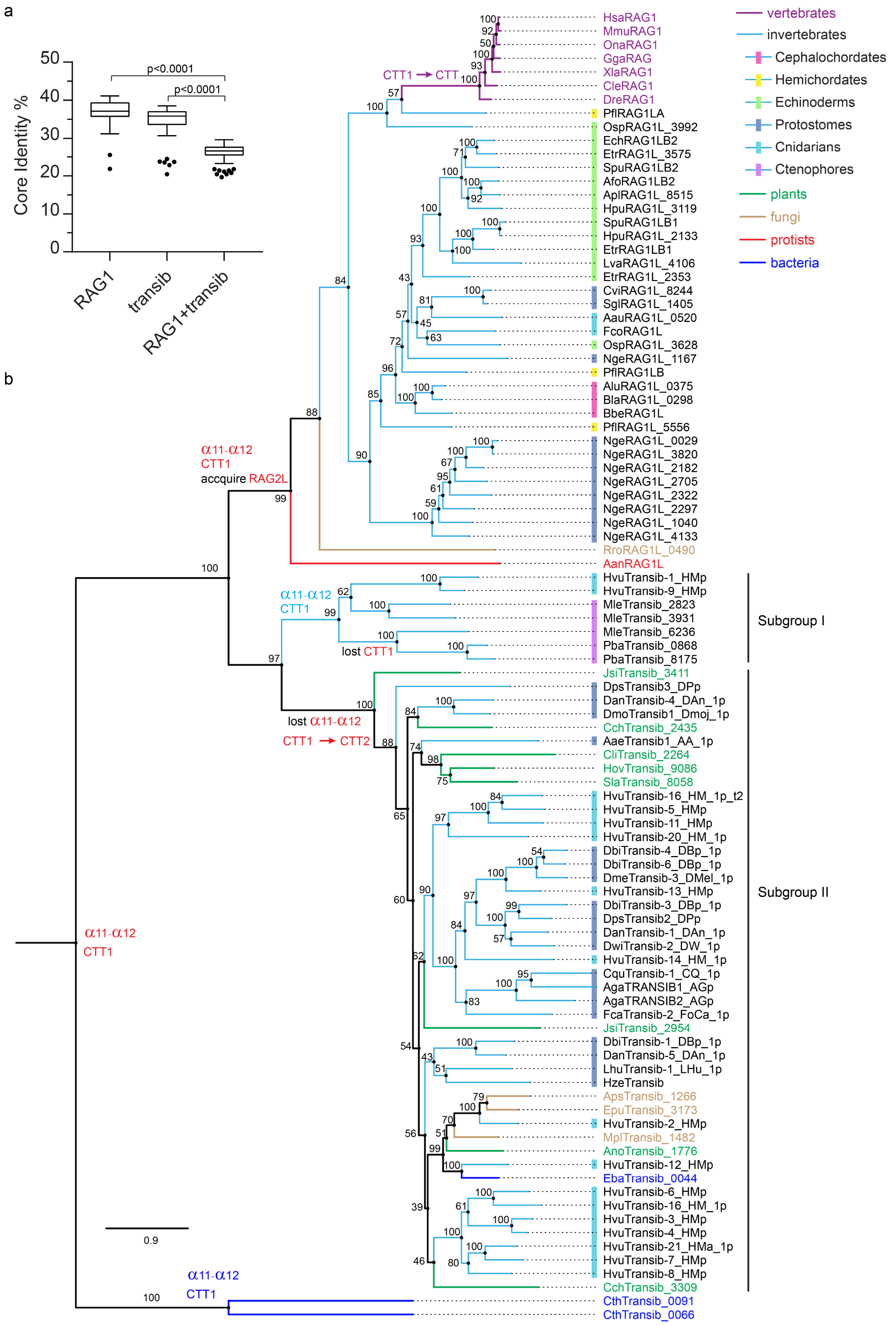
a
Core Identity %
50
40
30
20
10
0
p<0.0001
p<0.0001
RAG1
transib
RAG1+transib
b
vertebrates
invertebrates
Cephalochordates
Hemichordates
Echinoderms
Protostomes
Cnidarians
Ctenophores
plants
fungi
protists
bacteria
CTT1 → CTT
α11-α12
CTT1
accquire RAG2L
α11-α12
CTT1
lost CTT1
lost α11-α12
CTT1 → CTT2
α11-α12
CTT1
α11-α12
CTT1
Subgroup I
Subgroup II
0.9
HsaRAG1
MmuRAG1
OnaRAG1
GgaRAG
XlaRAG1
CleRAG1
DreRAG1
PflRAG1LA
OspRAG1L_3992
EchRAG1LB2
EtrRAG1L_3575
SpuRAG1LB2
AfoRAG1LB2
AplRAG1L_8515
HpuRAG1L_3119
SpuRAG1LB1
HpuRAG1L_2133
EtrRAG1LB1
LvaRAG1L_4106
EtrRAG1L_2353
CviRAG1L_8244
SglRAG1L_1405
AauRAG1L_0520
FcoRAG1L
OspRAG1L_3628
NgeRAG1L_1167
PflRAG1LB
AluRAG1L_0375
BlaRAG1L_0298
BbeRAG1L
PflRAG1L_5556
NgeRAG1L_0029
NgeRAG1L_3820
NgeRAG1L_2182
NgeRAG1L_2705
NgeRAG1L_2322
NgeRAG1L_2297
NgeRAG1L_1040
NgeRAG1L_4133
RroRAG1L_0490
AanRAG1L
HvuTransib-1_HMp
HvuTransib-9_HMp
MleTransib_2823
MleTransib_3931
MleTransib_6236
PbaTransib_0868
PbaTransib_8175
JsiTransib_3411
DpsTransib3_DPp
DanTransib-4_DAn_1p
DmoTransib1_Dmoj_1p
CchTransib_2435
AaeTransib1_AA_1p
CliTransib_2264
HovTransib_9086
SlaTransib_8058
HvuTransib-16_HM_1p_t2
HvuTransib-5_HMp
HvuTransib-11_HMp
HvuTransib-20_HM_1p
DbiTransib-4_DBp_1p
DbiTransib-6_DBp_1p
DmeTransib-3_DMel_1p
HvuTransib-13_HMp
DbiTransib-3_DBp_1p
DpsTransib2_DPp
DanTransib-1_DAn_1p
DwiTransib-2_DW_1p
HvuTransib-14_HM_1p
CquTransib-1_CQ_1p
AgaTRANSIB1_AGp
AgaTRANSIB2_AGp
FcaTransib-2_FoCa_1p
JsiTransib_2954
DbiTransib-1_DBp_1p
DanTransib-5_DAn_1p
LhuTransib-1_LHu_1p
HzeTransib
ApsTransib_1266
EpuTransib_3173
HvuTransib-2_HMp
MplTransib_1482
AnoTransib_1776
HvuTransib-12_HMp
EbaTransib_0044
HvuTransib-6_HMp
HvuTransib-16_HM_1p
HvuTransib-3_HMp
HvuTransib-4_HMp
HvuTransib-21_HMa_1p
HvuTransib-7_HMp
HvuTransib-8_HMp
CchTransib_3309
CthTransib_0091
CthTransib_0066

Supplementary Figure S3. Phylogenetic Analysis of RAG1 and Transib Homologs

a) Statistics of the average sequence identity between the core domains of RAG1Ls and Transibs. The RAG1 group contained 42 protein sequences of RAG1s and RAG1Ls. The Transib group contained 53 protein sequences of Transibs. The RAG1 and Transib group was the combination of the former two groups. Significant differences were analyzed with a two-tailed Student's t-test and the p values were shown.

b) Phylogenetic analysis of RAG1 and Transib homologs. The maximum-likelihood phylogenetic tree was constructed with IQ-TREE based on the core region of RAG1 and Transib homologs. The optimum LG+F+R6 model was tested and selected, and the ultrafast bootstrap (%) support was shown near the branch. The newly identified Transib proteins were marked with a prefix composed of three-letters abbreviation of the species name, like that of RAG1 homologs. The primary diversification of protein domains was shown near the branches of protein clans. Transib family were subdivided into two subgroups according to proteins phylogenetic relationship. Representative species abbreviation: Osp, *Ophiothrix spiculata*; Rro, *Rhizophlyctis rosea*; Mle, *Mnemiopsis leidyi*; Pba, *Pleurobrachia bachei*; Jsi, *Juglans sigillata*; Cch, *Capsicum chinense*; Cli, *Corrigiola litoralis*; Hov, *Hordeum vulgare*; Hvu, *Hydra vulgaris*; Sla, *Silene latifolia*; Aps, *Austropuccinia psidii*; Epu, *Erysiphe pulchra*; Mpl, *Massospora platypediae*; Cth, *Candidatus Thioglobus*. (The complete list of species abbreviations was shown in Supplementary Table S3.)

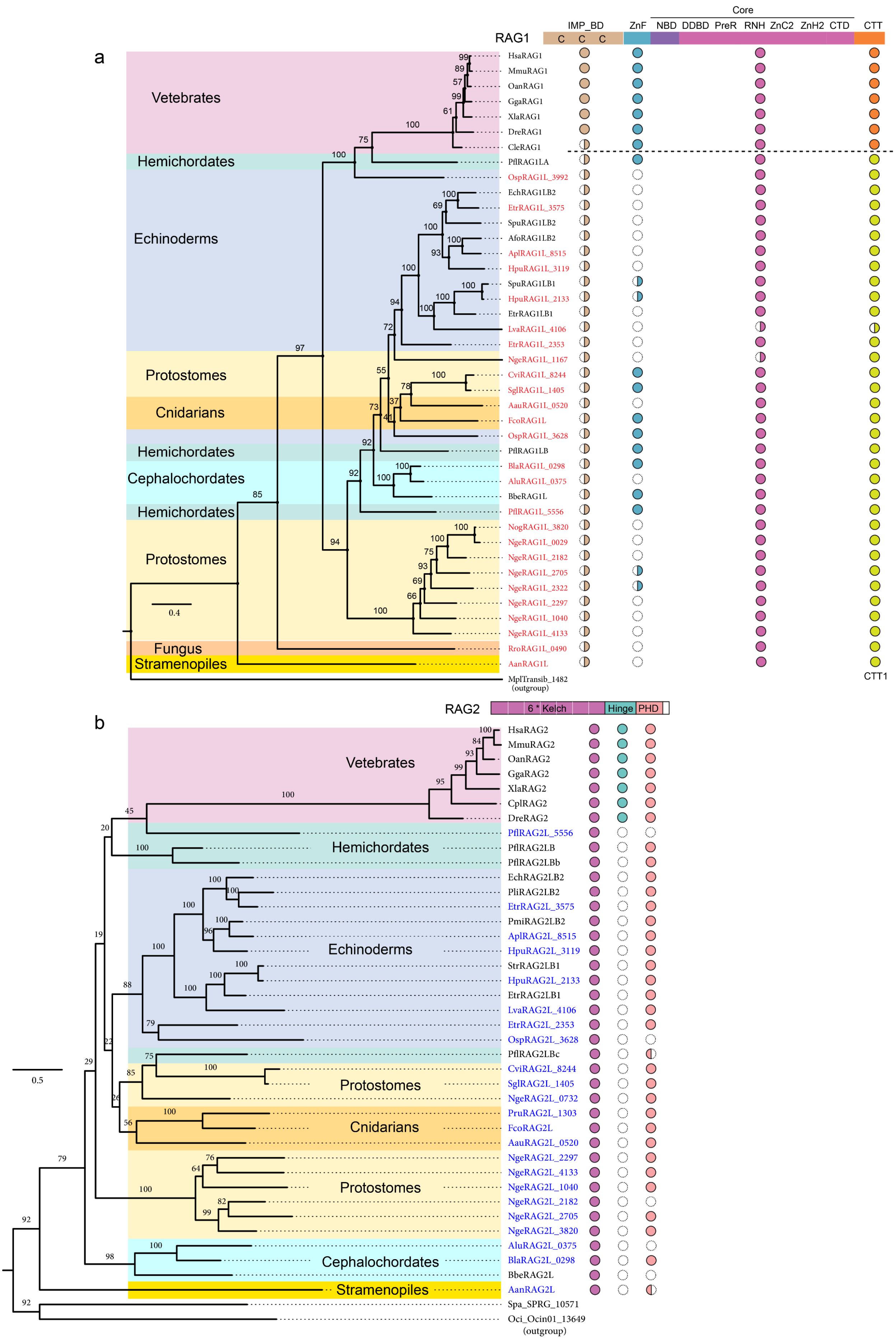
a
RAG1
Core
IMP_BD
ZnF
NBD
DDBD
PreR
RNH
ZnC2
ZnH2
CTD
CTT
C
C
C
Vetebrates
Hemichordates
Echinoderms
Protostomes
Cnidarians
Hemichordates
Cephalochordates
Hemichordates
Protostomes
Fungus
Stramenopiles
0.4
HsaRAG1
MmuRAG1
OanRAG1
GgaRAG1
XlaRAG1
DreRAG1
CleRAG1
PflRAG1LA
OspRAG1L_3992
EchRAG1LB2
EtrRAG1L_3575
SpuRAG1LB2
AfoRAG1LB2
AplRAG1L_8515
HpuRAG1L_3119
SpuRAG1LB1
HpuRAG1L_2133
EtrRAG1LB1
LvaRAG1L_4106
EtrRAG1L_2353
NgeRAG1L_1167
CviRAG1L_8244
SglRAG1L_1405
AauRAG1L_0520
FcoRAG1L
OspRAG1L_3628
PflRAG1LB
BlaRAG1L_0298
AluRAG1L_0375
BbeRAG1L
PflRAG1L_5556
NogRAG1L_3820
NgeRAG1L_0029
NgeRAG1L_2182
NgeRAG1L_2705
NgeRAG1L_2322
NgeRAG1L_2297
NgeRAG1L_1040
NgeRAG1L_4133
RroRAG1L_0490
AanRAG1L
MplTransib_1482
(outgroup)
CTT1
b
RAG2
6 * Kelch
Hinge
PHD
Vetebrates
Hemichordates
Echinoderms
Protostomes
Cnidarians
Protostomes
Cephalochordates
Stramenopiles
0.5
HsaRAG2
MmuRAG2
OanRAG2
GgaRAG2
XlaRAG2
CplRAG2
DreRAG2
PflRAG2L_5556
PflRAG2LB
PflRAG2LBb
EchRAG2LB2
PliRAG2LB2
EtrRAG2L_3575
PmiRAG2LB2
AplRAG2L_8515
HpuRAG2L_3119
StrRAG2LB1
HpuRAG2L_2133
EtrRAG2LB1
LvaRAG2L_4106
EtrRAG2L_2353
OspRAG2L_3628
PflRAG2LBc
CviRAG2L_8244
SglRAG2L_1405
NgeRAG2L_0732
PruRAG2L_1303
FcoRAG2L
AauRAG2L_0520
NgeRAG2L_2297
NgeRAG2L_4133
NgeRAG2L_1040
NgeRAG2L_2182
NgeRAG2L_2705
NgeRAG2L_3820
AluRAG2L_0375
BlaRAG2L_0298
BbeRAG2L
AanRAG2L
Spa_SPRG_10571
Oci_Ocin01_13649
(outgroup)

Supplementary Figure S4. Phylogenetic Analysis and Domain Composition of RAG Homologs across Eukaryotes.

a) The maximum-likelihood phylogenetic tree was construct with IQ-TREE based on the core region of RAG1 homologs. Most redundant RAG1Ls generated by duplications have been omitted in phylogenetic tree. The optimum LG+F+R5 model was tested and selected, and the ultrafast bootstrap (%) support was shown near the branch. Core domain of a Transib homolog in *Massospora platypediae* (fungus) was accepted as the outgroup. A diagram of the vertebrates RAG1s domains was shown on the top [10, 11]. The essential catalytic core of MmuRAG1 (387-1008 aa) consists of seven domains and the regulatory N-terminus of mRAG1 is composed of the importin binding domain (IMP_BD, 1-291 aa) and the zinc finger domain (ZnF, 292-383 aa) [12, 13], which were annotated through Pfam, and others in RAG1Ls were defined through multiple sequence alignment with vertebrate homologs. NBD, nonamer binding domain; DDBD, the dimerization and DNA binding domain; PreR, pre–RNase H domain; RNH, ribonuclease H-like domain; Zn^{2+}-binding domain coordinates Zn^{2+} through the two Cys (of ZnC2) and two His (of ZnH2); CTD, C-terminal domain; CTT, C-terminal tail; CTT1, type I C-terminal tail. Colored circle means existence, and half cycle means existence with partial sequences, and empty cycle means absence or unidentified.

b) Phylogenetic analysis and domain composition RAG2 homologs across eukaryotes. The maximum-likelihood phylogenetic tree was constructed with IQ-TREE based on the core region of RAG2 homologs. Most of the redundant RAG2Ls generated by duplications have been omitted in phylogenetic tree. The optimum LG+F+R4 model was tested and

selected, and the ultrafast bootstrap (%) support was shown near the branch. Core domain of two 6*kelch domain containing proteins in *Orchesella cincta* (Ecdysozoa) and *Saprolegnia parasitica* (Oomycete) was accepted as the outgroup. A diagram of the MmuRAG2s domains was shown [10, 11], and the essential core of MmuRAG2 is composed of a six-bladed β-propeller, which contacts with central regions of MmuRAG1 core. The plant homeodomain (PHD) (416-484 aa) and Hinge region (351-387 aa) in MmuRAG2 have been reported to be important for inhibition of the transposition activity of MmuRAG1/2 complex [14, 15]. Colored cycle means existence, and half cycle means absence of partial sequence, and blank cycle means not identified. Species abbreviation: Hsa, *Homo sapiens*; Mmu, *Mus musculus*; Oan, *Ornithorhynchus anatinus*; Gga, *Gallus gallus*; Xla, *Xenopus laevis*; Dre, *Danio rerio*; Cpl, Carcharhinus plumbeus; Cle, *Carcharhinus leucas*; Pfl, *Ptychodera flava*; Osp, *Ophiothrix spiculata*; Ech, *Evechinus chloroticus* ; Pli, *Paracentrotus lividus*; Etr, *Eucidaris tribuloides*; Lva, *Lytechinus variegatus*; Pmi, *Patiria miniata*; Spu, *Strongylocentrotus purpuratus*; Afo, *Asterias forbesi*; Apl, *Acanthaster planci*; Hpu, *Hemicentrotus pulcherrimus*; Nge, *Notospermus geniculatus*; Cvi, *Crassostrea virginica*; Sgl, *Saccostrea glomerata*; Aau, *Aurelia aurita*; Pru, *Porites rus*; Fco, *Fungia costulata*; Bla, *Branchiostoma lanceolatum*; Alu, *Asymmetron lucayanum*; Bbe, *Branchiostoma belcheri*; Nge, *Notospermus geniculatus*; Rro, *Rhizophlyctis rosea*; Aan, *Aureococcus anophagefferens*; Mpl, *Massospora platypediae*; Oci, *Orchesella cincta*; Spa, *Saprolegnia parasitica*

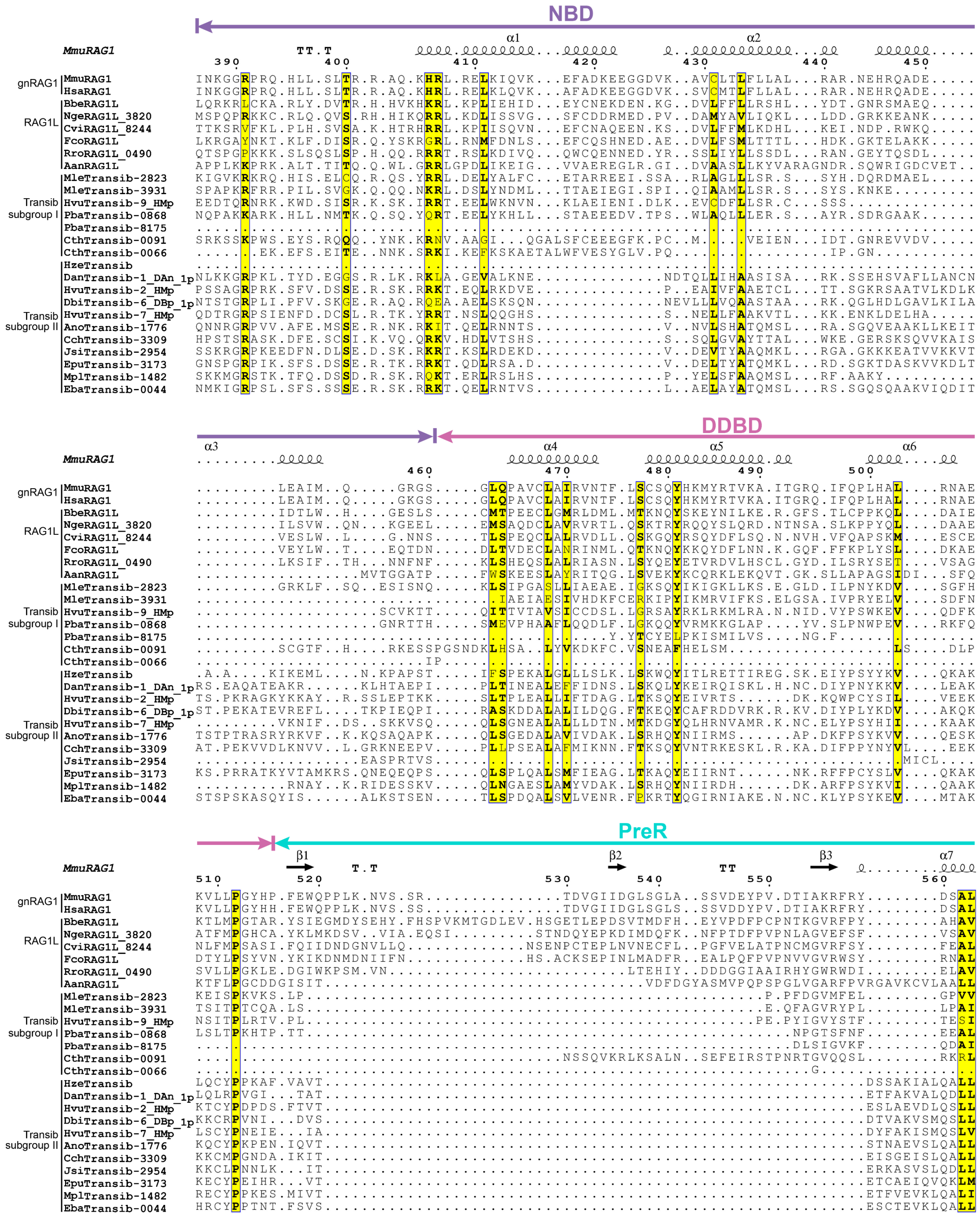
NBD
DDBD
PreR
MmuRAG1
gnRAG1
RAG1L
Transib subgroup I
Transib subgroup II
MmuRAG1
HsaRAG1
BbeRAG1L
NgeRAG1L_3820
CviRAG1L_8244
FcoRAG1L
RroRAG1L_0490
AanRAG1L
MleTransib-2823
MleTransib-3931
HvuTransib-9_HMp
PbaTransib-0868
PbaTransib-8175
CthTransib-0091
CthTransib-0066
HzeTransib
DanTransib-1_DAn_1p
HvuTransib-2_HMp
DbiTransib-6_DBp_1p
HvuTransib-7_HMp
AnoTransib-1776
CchTransib-3309
JsiTransib-2954
EpuTransib-3173
MplTransib-1482
EbaTransib-0044

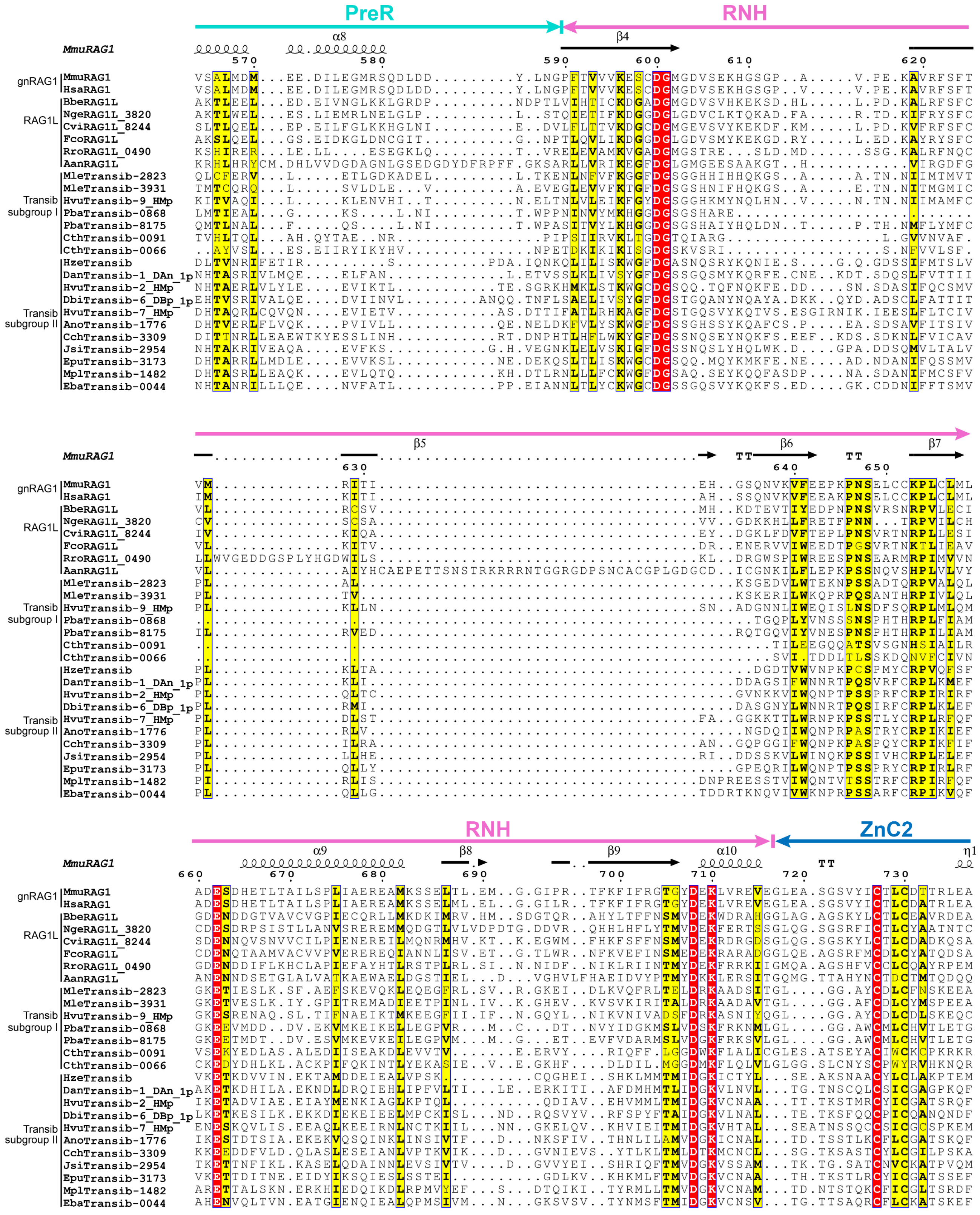
PreR
RNH
ZnC2
MmuRAG1
gnRAG1
RAG1L
Transib subgroup I
Transib subgroup II
MmuRAG1
HsaRAG1
BbeRAG1L
NgeRAG1L_3820
CviRAG1L_8244
FcoRAG1L
RroRAG1L_0490
AanRAG1L
MleTransib-2823
MleTransib-3931
HvuTransib-9_HMp
PbaTransib-0868
PbaTransib-8175
CthTransib-0091
CthTransib-0066
HzeTransib
DanTransib-1_DAn_1p
HvuTransib-2_HMp
DbiTransib-6_DBp_1p
HvuTransib-7_HMp
AnoTransib-1776
CchTransib-3309
JsiTransib-2954
EpuTransib-3173
MplTransib-1482
EbaTransib-0044

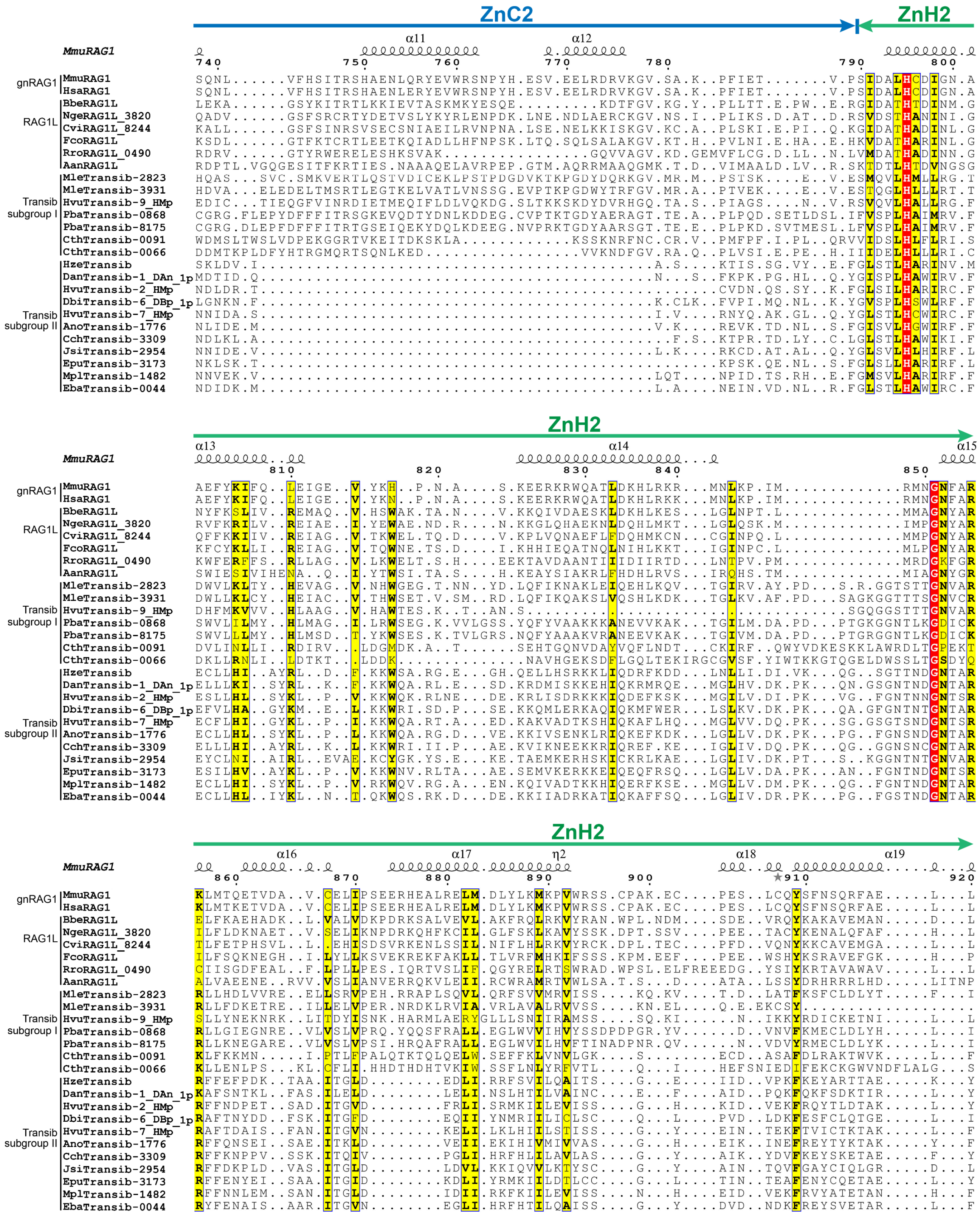

ZnC2
ZnH2
MmuRAG1
gnRAG1
RAG1L
Transib subgroup I
Transib subgroup II
MmuRAG1
HsaRAG1
BbeRAG1L
NgeRAG1L_3820
CviRAG1L_8244
FcoRAG1L
RroRAG1L_0490
AanRAG1L
MleTransib-2823
MleTransib-3931
HvuTransib-9_HMp
PbaTransib-0868
PbaTransib-8175
CthTransib-0091
CthTransib-0066
HzeTransib
DanTransib-1_DAn_1p
HvuTransib-2_HMp
DbiTransib-6_DBp_1p
HvuTransib-7_HMp
AnoTransib-1776
CchTransib-3309
JsiTransib-2954
EpuTransib-3173
MplTransib-1482
EbaTransib-0044

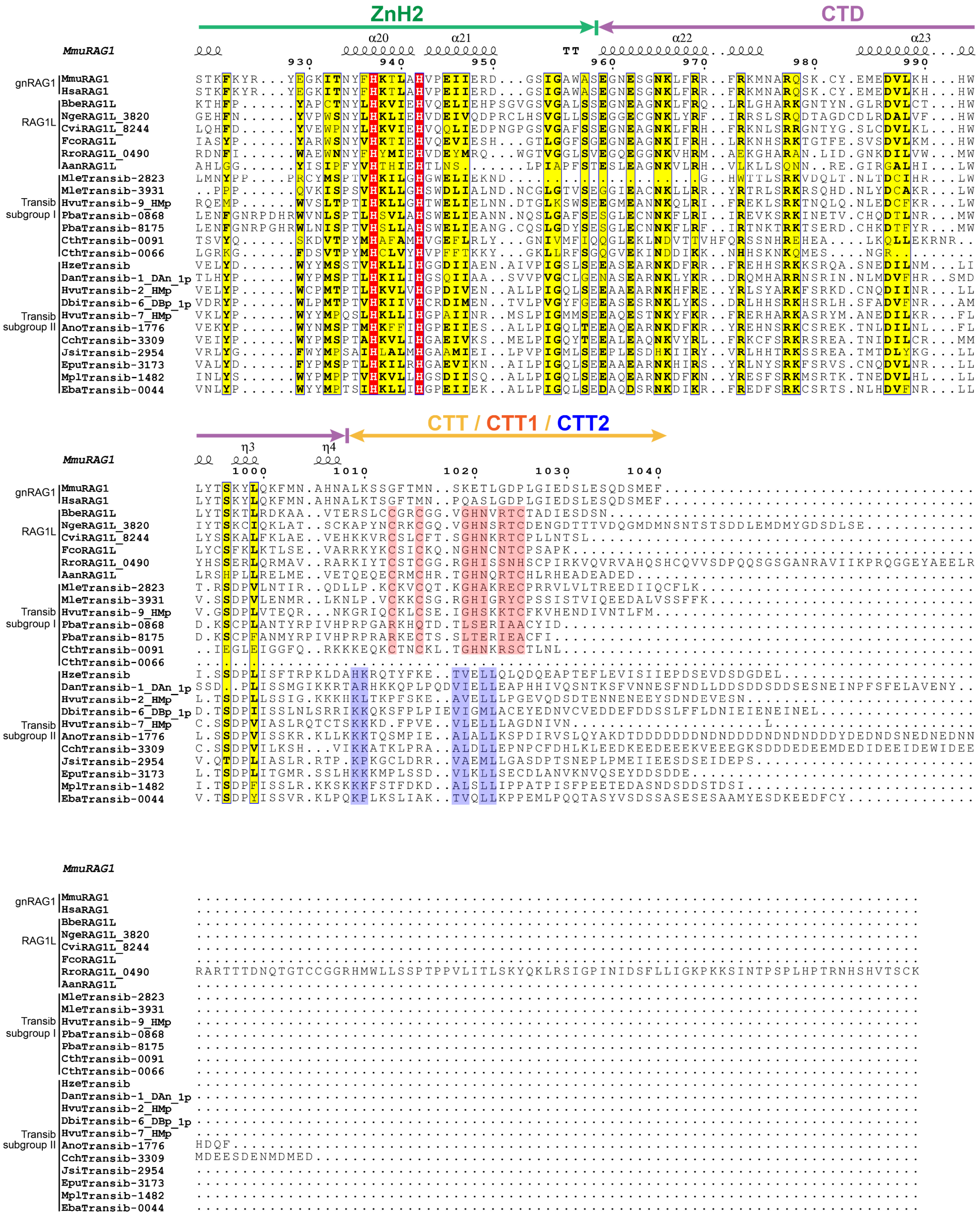

Supplementary Figure S5. Multiple sequence alignment of RAG1 and Transib homologs. The core domain of RAG1 and Transib homologs were displayed, and the secondary structure was assigned according to the MmuRAG1 cryo-EM structure (PDB: 6oet) using the

Esprit. The abbreviations of these domains have been listed in Supplementary Fig. S4. gnRAG1: gnathostome RAG1

Supplementary Table S1. Statistics of collected genomes for searching RAGL and Transib homologs

Taxon	Genomes
**Viruses**	35851
**Bacteria**	636399
**Archaea**	4868
**Fungi**	6172
**Plants**	1398
**Protozoans**	920
**Animals (invertebrates)**	1427
**In total**	687035

Supplementary Table S2. Sequences of primers for splinkerette PCR and genome cloning

**SPLNK-TOP**	5’-GATCCCACTAGTGTCGACACCAGTCTCTAATTTTTTTTTTCAAAAAAA-3’
**SPLNK-BOT**	5’-CGAAGAGTAACCGTTGCTAGGAGAGACCGTGGCTGAATGAGACTGGTGTCGACACTAGTGG-3’
**SPLNK_S1**	5’-CGAAGA-GTAACCGTTGCTAGGAGAGACC-3’
**SPLNK_S2**	5’-GTGGCTGAATGAGACTGGTGTCGAC-3’
**Transposon specific primers to clone the flanking sequences of *AanRAGL* from *A. anophagefferens***	
**3TIR_S1**	5’-CAGACACTGCTGGG-TATAGCGTAAGT-3’
**3TIR_S2**	5’-CGCGAAAATTCAAGATGGCGACCAGACA-3’
**5TIR_S1**	5’-CGCCGACCTTATGCACACGTAAGT-3’
**5TIR_S2**	5’-GGTCGAGTTACCCTGG-TCACG-3’
**Confirmation of *AanRAGL* insertion in Scaffold 5 from *A. anophagefferens***	
**AanRAG_Sca5_U1**	5’-AGATGGATACCACACCTC-3’
**AanRAG_Sca5_L1**	5’-CAGGCTGATGATCTTCTC-3’

Supplementary Table S3. Abbreviation and major taxa of species appeared in main figure and context

Abbreviations	Species Names	Major Taxa		
Aae	*Aedes aegypti*	Bilateria	Protostomia	Ecdysozoa
Aan	*Aureococcus anophagefferens*	Stramenopiles	Ochrophyta	Pelagophyceae
Aau	*Aurelia aurita*	Cnidaria	Scyphozoa	Semaeostomeae
Afo	*Asterias forbesi*	Bilateria	Deuterostomia	Echinodermata
Aga	*Anopheles gambiae*	Bilateria	Protostomia	Ecdysozoa
Alu	*Asymmetron lucayanum*	Bilateria	Deuterostomia	Chordata
Ano	*Arabis nordmanniana*	Viridiplantae	Streptophyta	Streptophytina
Apl	*Acanthaster planci*	Bilateria	Deuterostomia	Echinodermata
Aps	*Austropuccinia psidii*	Fungi	Dikarya	Basidiomycota
Bbe	*Branchiostoma belcheri*	Bilateria	Deuterostomia	Chordata
Bfl	*Branchiostoma floridae*	Bilateria	Deuterostomia	Chordata
Bla	*Branchiostoma lanceolatum*	Bilateria	Deuterostomia	Chordata
Cch	*Capsicum chinense*	Viridiplantae	Streptophyta	Streptophytina
Cle	*Carcharhinus leucas*	Bilateria	Deuterostomia	Chordata
Cli	*Corrigiola litoralis*	Viridiplantae	Streptophyta	Streptophytina
Cqu	*Culex quinquefasciatus*	Bilateria	Protostomia	Ecdysozoa
Cth	*Candidatus Thioglobus*	Bacteria	Proteobacteria	Gammaproteobacteria
Cvi	*Crassostrea virginica*	Bilateria	Protostomia	Lophotrochozoa
Dan	*Drosophila ananassae*	Bilateria	Protostomia	Ecdysozoa
Dbi	*Drosophila bipectinata*	Bilateria	Protostomia	Ecdysozoa
Dme	*Drosophila melanogaster*	Bilateria	Protostomia	Ecdysozoa
Dmo	*Drosophila mojavensis*	Bilateria	Protostomia	Ecdysozoa
Dps	*Drosophila pseudoobscura*	Bilateria	Protostomia	Ecdysozoa
Dre	*Danio rerio*	Bilateria	Deuterostomia	Chordata
Dwi	*Drosophila willistoni*	Bilateria	Protostomia	Ecdysozoa
Eba	*Entomoplasmatales bacterium*	Bacteria	Terrabacteria group	Tenericutes
Ech	*Evechinus chloroticus*	Bilateria	Deuterostomia	Echinodermata
Epu	*Erysiphe pulchra*	Fungi	Dikarya	Ascomycota
Etr	*Eucidaris tribuloides*	Bilateria	Deuterostomia	Echinodermata
Fca	*Folsomia candida*	Bilateria	Protostomia	Ecdysozoa
Fco	*Fungia costulata*	Cnidaria	Anthozoa	Hexacorallia
Gga	*Gallus gallus*	Bilateria	Deuterostomia	Chordata
Has	*Homo sapiens*	Bilateria	Deuterostomia	Chordata
Hma	*Hydra magnipapillata*	Cnidaria	Hydrozoa	Hydroidolina
Hov	*Hordeum vulgare*	Viridiplantae	Streptophyta	Streptophytina
Hpu	*Hemicentrotus pulcherrimus*	Bilateria	Deuterostomia	Echinodermata
Hvu	*Hydra vulgaris*	Cnidaria	Hydrozoa	Hydroidolina
Hze	*Helicoverpa zea*	Bilateria	Protostomia	Ecdysozoa
Jsi	*Juglans sigillata*	Viridiplantae	Streptophyta	Streptophytina
Lhu	*Linepithema humile*	Bilateria	Protostomia	Ecdysozoa
Lva	*Lytechinus variegatus*	Bilateria	Deuterostomia	Echinodermata
Mle	*Mnemiopsis leidyi*	Ctenophora	Tentaculata	Lobata
Mmu	*Mus musculus*	Bilateria	Deuterostomia	Chordata
Mpl	*Massospora platypediae*	Fungi	Fungi incertae sedis	Zoopagomycota
Nge	*Notospermus geniculatus*	Bilateria	Protostomia	Lophotrochozoa

Ona	*Ornithorhynchus anatinus*	Bilateria	Deuterostomia	Chordata
Osp	*Ophiothrix spiculata*	Bilateria	Deuterostomia	Echinodermata
Pba	*Pleurobrachia bachei*	Ctenophora	Tentaculata	Cydippida
Pfl	*Ptychodera flava*	Bilateria	Deuterostomia	Hemichordata
Pmi	*Patiria miniata*	Bilateria	Deuterostomia	Echinodermata
Rro	*Rhizophlyctis rosea*	Fungi	Fungi incertae sedis	Chytridiomycota
Sgl	*Saccostrea glomerata*	Bilateria	Protostomia	Lophotrochozoa
Sla	*Silene latifolia*	Viridiplantae	Streptophyta	Streptophytina
Spu	*Strongylocentrotus purpuratus*	Bilateria	Deuterostomia	Echinodermata
Xla	*Xenopus laevis*	Bilateria	Deuterostomia	Chordata

References

1. Liew YJ, Aranda M, Voolstra CR. Reefgenomics.Org - a repository for marine genomics data. *Database (Oxford)*. 2016; **2016**.
2. Brozovic M, Dantec C, Dardaillon J *et al.* ANISEED 2017: extending the integrated ascidian database to the exploration and evolutionary comparison of genome-scale datasets. *Nucleic Acids Res*. 2018; **46**: D718-D25.
3. Pearson WR. An introduction to sequence similarity ("homology") searching. *Curr Protoc Bioinformatics*. 2013; **Chapter 3**: Unit3 1.
4. Bao W, Kojima KK, Kohany O. Repbase Update, a database of repetitive elements in eukaryotic genomes. *Mob DNA*. 2015; **6**: 11.
5. Hoang DT, Chernomor O, von Haeseler A *et al.* UFBoot2: Improving the Ultrafast Bootstrap Approximation. *Mol Biol Evol*. 2018; **35**: 518-22.
6. Chernomor O, von Haeseler A, Minh BQ. Terrace Aware Data Structure for Phylogenomic Inference from Supermatrices. *Syst Biol*. 2016; **65**: 997-1008.
7. Nguyen LT, Schmidt HA, von Haeseler A *et al.* IQ-TREE: a fast and effective stochastic algorithm for estimating maximum-likelihood phylogenies. *Mol Biol Evol*. 2015; **32**: 268-74.
8. Simakov O, Kawashima T, Marletaz F *et al.* Hemichordate genomes and deuterostome origins. *Nature*. 2015; **527**: 459-65.
9. Hedges SB, Marin J, Suleski M *et al.* Tree of life reveals clock-like speciation and diversification. *Mol Biol Evol*. 2015; **32**: 835-45.
10. Kim MS, Lapkouski M, Yang W *et al.* Crystal structure of the V(D)J recombinase RAG1-RAG2. *Nature*. 2015; **518**: 507-11.
11. Ru H, Chambers MG, Fu TM *et al.* Molecular Mechanism of V(D)J Recombination from Synaptic RAG1-RAG2 Complex Structures. *Cell*. 2015; **163**: 1138-52.
12. Cortes P, Ye ZS, Baltimore D. RAG-1 interacts with the repeated amino acid motif of the human homologue of the yeast protein SRP1. *Proc Natl Acad Sci U S A*. 1994; **91**: 7633-7.
13. Brecht RM, Liu CC, Beilinson HA *et al.* Nucleolar localization of RAG1 modulates V(D)J recombination activity. *Proc Natl Acad Sci U S A*. 2020; **117**: 4300-9.
14. Zhang Y, Cheng TC, Huang G *et al.* Transposon molecular domestication and the evolution of the RAG recombinase. *Nature*. 2019; **569**: 79-84.
15. Grundy GJ, Yang W, Gellert M. Autoinhibition of DNA cleavage mediated by RAG1 and RAG2 is overcome by an epigenetic signal in V(D)J recombination. *Proc Natl Acad Sci U S A*. 2010; **107**: 22487-92.
